# Development and Qualification of a Nipah Virus Glycoprotein-Specific IgG ELISA for the Assessment of Human Antibody Responses

**DOI:** 10.3390/vaccines14060534

**Published:** 2026-06-16

**Authors:** Mohammad Mamun Alam, Tahsin Tabassum Anonto, Sinthia Karim, Gathoni Kamuyu, Ali Azizi, Ayesha Siddika, Shadman Sakib Choudhury, Md Wasik Rahman, Anika Farzin, Dewan Imtiaz Rahman, Rubhana Raqib, Mustafizur Rahman, Sharmin Sultana, Trevor Shoemaker, Michael K. Lo, Sayera Banu, Tahmina Shirin, Christina F. Spiropoulou, Joel M. Montgomery, Syed Moinuddin Satter, Mohammed Ziaur Rahman

**Affiliations:** 1 One Health Laboratory, Infectious Diseases Division, International Centre for Diarrhoeal Disease Research, Mohakhali, Dhaka 1212, Bangladesh; mamun.alam@icddrb.org (M.M.A.); anontotahsin@gmail.com (T.T.A.); sinthiakarim39@gmail.com (S.K.); ayesha.siddika@icddrb.org (A.S.); shadman.choudhury@icddrb.org (S.S.C.); wasik.rahman@icddrb.org (M.W.R.); anika.farzin@icddrb.org (A.F.); dewan.rahman@icddrb.org (D.I.R.); rubhana@icddrb.org (R.R.); mustafizur@icddrb.org (M.R.); sbanu@icddrb.org (S.B.); dr.satter@icddrb.org (S.M.S.); 2 Coalition for Epidemic Preparedness Innovations (CEPI), 6th Floor, Henry Wood House, 4-5 Langham Place, London W1B 3DG, UK; gathoni.kamuyu@cepi.net; 3 Coalition for Epidemic Preparedness Innovations (CEPI), 1899 Pennsylvania Avenue NW, Washington, DC 20006, USA; ali.azizi@cepi.net; 4 Institute of Epidemiology, Disease Control and Research (IEDCR), Mohakhali, Dhaka 1212, Bangladesh; dr.sharmin1579@yahoo.com (S.S.); tahmina.shirin14@gmail.com (T.S.); 5 Viral Special Pathogens Branch, Division of High-Consequence Pathogens and Pathology, National Center for Emerging and Zoonotic Infectious Diseases, Centers for Disease Control and Prevention, 1600 Clifton Rd. NE, Atlanta, GA 30333, USA; tis8@cdc.gov (T.S.); mko2@cdc.gov (M.K.L.); ccs8@cdc.gov (C.F.S.); ztq9@cdc.gov (J.M.M.)

**Keywords:** Nipah virus, IgG ELISA, glycoprotein G, assay validation, serosurveillance, vaccine evaluation, BSL-2 diagnostic platform, convalescent sera, henipavirus, outbreak response

## Abstract

**Background/Objectives:** Nipah virus (NiV) is a highly pathogenic zoonotic virus with fatality rates exceeding 70% and causes recurring outbreaks in South and Southeast Asia. Reliable serological assays are critical for outbreak surveillance, diagnosis, and evaluation of vaccine-induced immune responses. This study aimed to develop and qualify an indirect enzyme-linked immunosorbent assay (ELISA) based on recombinant NiV glycoprotein G for the detection of virus-specific IgG antibodies in human serum. **Methods:** An indirect ELISA was developed and optimized for antigen concentration, blocking conditions, and serum dilution. The assay performance was evaluated using convalescent human sera from Bangladesh, along with the World Health Organization (WHO) International Standard for anti-Nipah virus antibodies, maintained and distributed by the National Institute for Biological Standards and Control (NIBSC). Analytical validation was conducted in accordance with ICH Q2 (R2) guidelines, including assessments of sensitivity, specificity, Precision, Linearity, and detection limits. **Results:** The assay demonstrated 100% sensitivity and specificity relative to reference sera. Intra-assay coefficients of variation ranged from 0.36% to 5.73%, and inter-assay variation was 4.16%, indicating high precision. The ELISA showed excellent Linearity (R^2^ > 0.995). The lower limit of detection was 0.51 IU/mL, and the lower limit of quantification was 0.98 IU/mL. **Conclusions:** The developed ELISA is a BSL-2-compatible, robust, and scalable platform suitable for serosurveillance and the assessment of vaccine-induced immunity in endemic regions. Calibration against an international standard supports its applicability for standardized antibody measurement. This assay provides a practical tool for NiV outbreak response and vaccine evaluation.

## 1. Introduction

Nipah virus (NiV) is a highly pathogenic zoonotic paramyxovirus of the Henipavirus genus, with human fatality rates ranging from 40% to over 70% depending on the outbreak strain and epidemiological context [1,2,3]. Since its first identification in 1998–1999 during a large outbreak in Malaysia and Singapore, NiV has emerged as a recurrent public health threat in South and Southeast Asia [1,3,4]. Bangladesh has experienced multiple documented outbreaks and sporadic cases since 2001, with case fatality rates frequently exceeding 70%, representing a significant epidemiological burden [2,5]. The initial Malaysia–Singapore outbreak was associated with exposure to infected pigs, whereas subsequent outbreaks in Bangladesh have been linked mainly to bat-to-human and human-to-human transmission [3,4,5].

Transmission to humans occurs through multiple pathways, including direct contact with infected animals, particularly fruit bats (*Pteropus* species), which serve as the natural reservoir, consumption of contaminated foods such as date palm sap, or exposure to bat body fluids and feces [2,4,5]. Human-to-human transmission occurs through direct contact with infected individuals’ body fluids, and healthcare-associated transmission in facilities lacking appropriate infection control measures has amplified outbreak severity in clinical settings [2,5]. The clinical manifestations of NiV infection are highly variable and include asymptomatic seroconversion, acute respiratory infection with potential progression to acute respiratory distress syndrome, and encephalitis presenting with fever, altered mental status, seizures, and neurological complications [2,5]. This pathogenic heterogeneity presents significant diagnostic challenges, as initial clinical presentation may resemble other endemic febrile illnesses or respiratory infections, delaying timely recognition and appropriate infection control measures. The absence of licensed vaccines and limited therapeutic options, combined with rapid transmission potential in healthcare settings, underscores the critical need for sensitive, rapid, and reliable diagnostic tools [6].

Several serological and virological methods are available for NiV diagnosis, each with distinct technical requirements and practical applicability. The plaque reduction neutralization test remains the reference standard for evaluating neutralizing antibody responses and is particularly valuable for vaccine studies; however, it requires biosafety level 4 containment facilities equipped for handling live virus, rendering this approach impractical for routine diagnostics in endemic regions [7,8]. Direct virus isolation through tissue culture is another reference method but similarly demands high-containment facilities, specialized equipment, and extended result turnaround times, making it unsuitable for high-volume outbreak response testing [1,6].

Indirect fluorescent antibody assays targeting NiV-specific IgM and IgG have been employed historically and offer moderate sensitivity and specificity; however, interpretation can be subjective and dependent on operator expertise and microscope availability, resulting in reproducibility challenges across different laboratories [7,8]. Western blotting provides high specificity but requires significant technical expertise and produces results too slowly for acute outbreak response [1,6]. Reverse-transcription polymerase chain reaction assays detect viral nucleic acid rapidly but are most useful during the acute infection phase and require expensive equipment and trained personnel [2,6].

In contrast, enzyme-linked immunosorbent assays employing recombinant antigens provide practical advantages for endemic-region deployment [7,8,9]: (1) they require only BSL-2 containment when using recombinant antigens; (2) they provide objective, quantitative results less dependent on operator interpretation; (3) they can be readily scaled to high-throughput applications; (4) they generate minimal biological hazard waste; and (5) they can be combined with automation for large-scale applications [9]. However, reliability depends critically on proper assay development and analytical qualification [10,11].

The envelope glycoproteins of Nipah virus exist in two distinct forms: the attachment glycoprotein G and the fusion glycoprotein F. The G protein is the viral attachment protein responsible for cellular recognition through binding to ephrin B2 and B3 receptors and serves as the primary determinant of viral cell tropism [12]. More significantly, the G protein is a major target of virus-specific neutralizing antibodies in both naturally infected and vaccinated individuals [12,13]. This critical role makes it an appropriate antigen choice for detecting and quantifying antibody responses relevant to vaccine-induced immunity [13,14].

The F protein is involved in membrane fusion, whereas the attachment G protein is more directly tied to receptor binding and has been widely used as an immunogen and antibody target in henipavirus vaccine and serological studies [12,13]. Using G protein as the target antigen, therefore, allows the assay to measure an immunologically relevant antibody response. Furthermore, G protein-based assays align with current NiV vaccine-development programs, including CEPI-supported candidates based on soluble Hendra virus G glycoprotein, where binding and neutralizing antibody responses are used for immunogenicity and correlate-of-protection assessment [15,16].

Critically, the G protein of NiV shares structural and antigenic relationships with Hendra virus G protein, raising the potential for cross-reactive antibody responses between NiV and HeV infection [12,17]. This structural homology is essential to consider in serological diagnosis in endemic regions where related henipaviruses may circulate. Our assay development strategy incorporated systematic evaluation for cross-reactivity, which is essential for diagnostic specificity and accurate disease attribution.

High-throughput multiplex platforms, such as Luminex or electrochemiluminescence systems, are often impractical in low-resource endemic-region settings due to high equipment costs and complex infrastructure requirements [10,18]. Additionally, these platforms may be susceptible to matrix interference, reducing reliability in heterogeneous human sera [10]. Therefore, simple, robust ELISA-based assays that require minimal specialized equipment remain particularly valuable for endemic regions.

To address this diagnostic gap, this study developed and rigorously validated an indirect IgG ELISA targeting NiV glycoprotein G. The assay was systematically optimized and validated in accordance with ICH Q2 (R2) guideline [19]. The recombinant antigen-based design ensures BSL-2 compatibility, reducing biosafety risks while maintaining high analytical performance [20]. The assay was calibrated against the WHO/NIBSC International Standard, providing a common reference for standardizing antibody measurements across different laboratories [21]. The resulting platform provides a cost-effective, reproducible, high-throughput method suitable for serosurveillance, outbreak investigations, occupational health surveillance, and vaccine immunogenicity assessment.

## 2. Materials and Methods

### 2.1. Clinical Samples

Samples used for assay qualification included the NV-1, WHO International Standard (NIBSC code 22/130_BA), and additional serum samples provided by NIBSC. These included reference panel sera from Bangladesh and Malaysia (NV-2, NV-10) and collaborative study samples from Bangladesh and Malaysia (NV-4, NV-6), as labeled by NIBSC. The assigned antibody concentrations for each sample are shown in Table 1. Assay qualification was also included using archived positive samples, determined as Nipah Human Positive (NHP), NHP1-NHP7, and negative samples of healthy individuals—Negative Control (NC), NC1 to NC8, and negative serum commercially obtained (CNC), which had been previously tested with the IgM and IgG ELISA protocols in line with the CDC protocol [22]. Additional details regarding these sera and their roles in assay validation are provided in Table 1.

### 2.2. Ethical Statement

This investigation did not necessitate the primary collection of clinical samples. All serum samples from individuals (NHP-1, NHP-3, NHP-5, NHP-6, and NHP-7) who had recovered from NiV exposure in Bangladesh were approved by the Institutional Review Committee (IRB) of icddr,b. These samples were characterized, pooled, and a control was prepared using commercially available control human serum (Sigma Aldrich, H4522, Germany). And the NV-1, NV-2, NV-4, NV-6, and NV-10 were supplied by the Medicines and Healthcare products Regulatory Agency (MHRA), with collections handled by Universiti Malaya (Kuala Lumpur, Malaysia) and icddr,b (Dhaka, Bangladesh). The collections were approved by the Institutional Review Committee (IRB) and the Medical Research and Ethics Committee (MREC) of the Universiti Malaya Medical Centre (UMMC).

### 2.3. Development of ELISA

#### 2.3.1. Evaluation of Coating Antigen and Determination of Optimal Concentration

During assay development, the Nipah Virus Glycoprotein G Mouse Fc-Tag (The Native Antigen Company, Kidlington, UK, Strain: Malaysia 2008, CAT# REC31631) was selected as the coating antigen due to its commercial availability. Two coating concentrations, 0.2 μg/mL and 0.5 μg/mL, were evaluated for their impact on assay performance. Although the 0.2 μg/mL coating concentration yielded a higher optical density (OD), it failed to meet the validation criteria because the OD of the known negative sample exceeded the predetermined cut-off value.

To optimize the assay, a coating concentration of 0.5 μg/mL was employed, which effectively minimized nonspecific interactions, thereby enhancing assay specificity and reducing the risk of false-positive results. However, some known negative serum samples, such as commercially available Human Serum Type AB (Sigma-Aldrich, St. Louis, MO, USA, CAT# H4522), exhibited false-positive reactions for the Nipah Virus Glycoprotein G Mouse Fc-Tag.

Subsequently, the assay was further optimized by introducing a new coating antigen, Recombinant Nipah Virus Glycoprotein G (Creative Diagnostics, Shirley, NY, USA, DAGA-1004). Given that the previous coating concentration of 0.5 μg/mL yielded more reliable results, it was retained in the optimization phase, along with testing additional concentrations. For the optimization of the ELISA using the new coating, various concentrations of Recombinant Nipah Virus Glycoprotein G (Creative Diagnostics, Strain: Malaysia and Singapore, DAGA-1004) were evaluated, ranging from 0.2 μg/mL, 0.5 μg/mL, 1.0 μg/mL, 2.0 μg/mL, to 3.0 μg/mL, to identify the optimal concentration for improved assay performance.

#### 2.3.2. Dilution Factor Determination of Serum for ELISA

The Nipah virus, being a BSL-4 organism, imposes significant restrictions. The CDC-recommended protocol advises heat inactivation of serum, which requires diluting it by adding 21 μL of serum to 500 μL of skim milk (1:23.8) [22] Therefore, the assay dilution was established at 1:24.

#### 2.3.3. Selection of Blocking Buffer and Substrate

Blocking Buffer: Two blocking solutions, 1% Bovine Serum Albumin (BSA, Sigma-Aldrich, Cat# A39454) and 5% skim milk (BD BBL™/Difco™, Cat# 11792994, Nippon Becton Dickinson Company, Ltd., Franklin Lakes, NJ, USA), were evaluated for their effectiveness in blocking nonspecific binding. Both solutions provided comparable results; however, 1% BSA was selected for the assay due to its equivalent performance and convenient availability in the laboratory setting.

Substrate: During assay development, two substrates were tested: TMB (Bio-Rad, Hercules, CA, USA, Cat# 1721072) and o-phenylenediamine dihydrochloride (OPD, Sigma-Aldrich, Cat# P1526).

#### 2.3.4. Determining the Secondary Antibody Conjugate Dilutions

Initial testing was conducted using various dilutions of the secondary antibody, Rabbit Anti-Human IgG-Fc Secondary Antibody (HRP, Rabbit PAb, Antigen Affinity Purified, Sino Biological, Cat# 10702-T16-H, Beijing, China), including 1:5000, 1:10,000, and 1:20,000. The optimal performance was achieved with the 1:5000 dilution.

#### 2.3.5. Evaluation and Identification of Optimum Time for Adding Stop Solution

TMB is a highly sensitive substrate commonly used to produce soluble reaction products with HRP. Due to its rapid reaction rate, the optimal time for adding the stop solution (1 M H_2_SO_4_) was evaluated at 10, 15, and 20 min.

### 2.4. ELISA Procedure

The indirect ELISA was developed to detect anti-Nipah IgG specific to the NiV glycoprotein G antigen. Briefly, 96-well high-binding microtiter plates were coated with 100 μL/well of Recombinant Nipah Virus Glycoprotein G (Creative Diagnostics, Strain: Malaysia and Singapore, Cat# DAGA-1004) at 0.5 µg/mL concentration in carbonate-bicarbonate buffer, and incubated overnight at 4 °C. The next day, wells were washed three times with 300 μL/well of 0.01M Phosphate Buffered Saline (PBS) (Sigma Aldrich, Cat# P3813-10PAK) + 0.1% Tween^20^ (Sigma Aldrich, Cat# P1379) (PBS-T) with a holding time of 3 min between each wash and blocked for 2 h at 37 °C with 200 μL/well of 1% Bovine serum albumin. Serum samples were prediluted in 5% Skim Milk at a 1:24 ratio and heat-inactivated at 56 °C for 30 min before adding to the plates. After blocking, the plates were washed as in the previous step. After the washing step, 100 µL of 0.1% BSA diluent was added to the wells of rows B-H. Next, 200 µL of heat-inactivated serum was added to row A according to the plate layout, then a two-fold serial dilution was carried out. Then, 0.1% BSA was added to the designated Blank column. After that, the plates were incubated at 37 °C for one hour. Following incubation, each plate was washed three times, with a 5 min hold between washes. Rabbit Anti-Human IgG-Fc Secondary Antibody (HRP), Rabbit PAb, Antigen Affinity Purified (Sino Biological, Cat#10702-T16-H), was used in the assay for the detection of IgG. The HRP-conjugated secondary antibody was diluted in 0.1% BSA to a 1:500 ratio. 100 µL of diluted secondary antibodies was added to each well and incubated for 1 h at 37 °C. Plates were then washed using the same procedure, and 100 μL/well of substrate TMB (3,3′, 5,5′–Tetramethylbenzidine Liquid Substrate, Bio-Rad) was added. The plates were incubated for 15 min. The reaction was stopped with 100 μL/well of 1 M H_2_SO_4_, and the sample was immediately read at 450 nm (Victor Nivo, Victor Nivo control software 5.0.1, Cat# HH35000000, Revvity, PerkinElmer, Shelton, CT, USA).

Determination of cut-off values and assay validation: The Cut-off value for the assay to determine the positive or negative results using the formula:

Cut-Off Value = 2 × (Average of Blank + SD)

The sample will be considered positive if the optical density (OD) is greater than or equal to the cut-off value and negative if the OD is less than the cut-off value.

### 2.5. Statistical Analysis

Optical density (OD) values were measured using Gen5 Microplate Reader software Gen-5 3.16 (BioTek, Winooski, VT, USA). Four-parameter logistic (4PL) curves were generated in GraphPad Prism v9 to optimize the ELISA conditions. For assay qualification, sample concentrations were calculated in international units per milliliter (IU/mL) using SoftMax Pro software (version 7.1.1, GxP edition, Molecular Devices, San Jose, CA, USA).

To quantify samples relative to the WHO International Standard for anti-Nipah virus antibodies (custodied by NIBSC) (NV-1, NIBSC code 22/130_BA), a parallel-line calibration was performed. The NV-1 reference was prediluted at 1:24, followed by two-fold serial dilutions across 8 wells (rows A–H) on each plate. Sample sera were diluted in parallel. The dose–response curves of the reference and samples were fitted using SoftMax Pro software (version 7.1.1, GxP edition), and sample concentrations were calculated in IU/mL relative to the standard. This approach ensured that all reported concentrations, as well as LLOD and LLOQ values, are fully traceable to the international standard.

### 2.6. Qualification of ELISA

The assay qualification was conducted in accordance with ICH Q2 (R2) guidelines [19].

#### 2.6.1. Sensitivity and Specificity

The sensitivity and specificity of the in-house indirect ELISA were assessed using the following criteria: 10 positive samples (5 reference panel sera, including NV-1 WHO IS as the standard, and 5 archived positive convalescent sera) were tested 3 times over 3 days. These tests ensured the assay’s reliability in distinguishing true positives and true negatives, as required by ICH Q2 (R2) guidelines [19].

#### 2.6.2. Precision

The precision of the assay was tested both within (intra-assay precision) and between (inter-assay precision) over several days:

Intra-assay Precision (Repeatability): 10 serum samples (3 NIBSC reference serum NV-1 WHO IS as the standard, 3 archived positive convalescent serum, and 4 negative samples) were tested once in response to one ELISA. Both samples were done twice each. The coefficient of variation (CV %) was obtained with [23]:

CV (%) = (Standard Deviation/Mean) × 100%

Inter-assay Precision (Reproducibility): Tests with the same serum were repeated across 3 different assay runs (over a series of days, across different assay runs, or among assessors). The overall geometric coefficient of variation was calculated, and the forecast overall maximum was below or equal to 30 percent in terms of ICH Q2 (R2) [19].

#### 2.6.3. Linearity

The Linearity of the indirect ELISA was determined by performing fourfold dilutions of three NIBSC references, and WHO IS NV-1 as the standard. OD values were recorded at each dilution, and a log10 plot was drawn to investigate linearity. The resulting calibration curve was expected to be approximately linear, with the **correlation coefficient (R^2^) approaching 1** [24]. The 95% confidence and prediction intervals were calculated from the fitted linear model using the standard error of the mean response and residual variance, respectively.

#### 2.6.4. Accuracy

By comparing measured and expected concentrations with NIBSC samples, the assay’s accuracy was validated. It was expected that the findings would range from 80% to 120% of the reference values. Five serum samples were tested: one negative control, three NIBSC reference samples, and NV-1 as a standard, in a single run submitted in compliance with ICH Q2 (R2) [19,25].

#### 2.6.5. Limits of Detection and Quantification

By evaluating repeated dilutions and reference standards within the assay range, the lower limit of detection (LLOD), lower limit of quantification (LLOQ), upper limit of detection (ULOD), and upper limit of quantification (ULOQ) were determined [26]. These parameters were established to provide acceptable precision and accuracy at the lowest and highest concentrations [25,26]. A four-parameter logistic regression was used to model the relationship between concentration and response in ELISA, accounting for the assay’s sigmoidal nature. The ICH Guideline Q2 (R2) [19] was used for determining the lower limits of detection (LLOD) and quantification (LLOQ). On the other hand, a rule-based method was used to establish the upper limits of detection (ULOD) and quantification (ULOQ).

## 3. Results

Optimization of Anti-Nipah IgG ELISA

**Figure 1:**
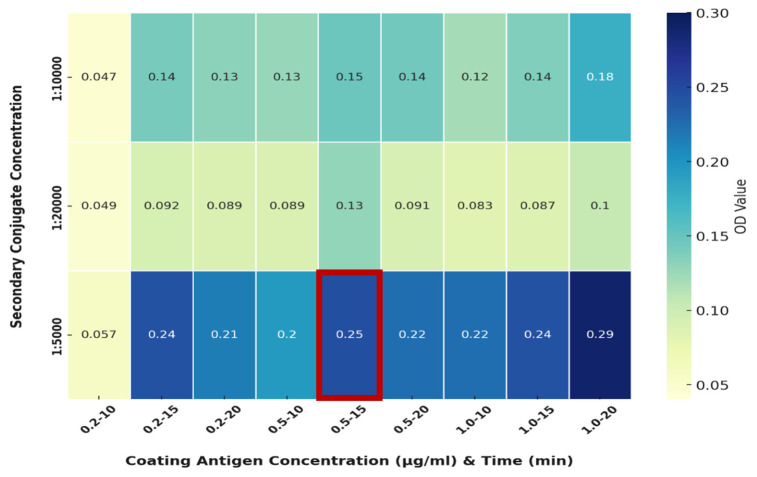
Coating Antigen Concentration vs Secondary Conjugate Concentration (Substrate Incubation Period). This heatmap illustrates the effect of Coating Antigen Concentration, Secondary Conjugate Concentration, Substrate Incubation Time, and serum dilution on OD values in an ELISA. The serum was diluted at a 1:24 ratio as per CDC protocol. The optimal conditions for maximum performance are 0.5 µg/mL Coating Antigen, 1:5000 Secondary Conjugate dilution, and a 15 min incubation time, highlighted in red. Lower Coating Antigen concentrations 0.2 µg/mL result in weak antigen–antibody interaction, while higher concentrations 1.0 µg/mL cause over-saturation, reducing assay sensitivity. Secondary Conjugate dilutions of 1:10,000 or 1:20,000 provide insufficient signal amplification, and incubation times shorter or longer than 15 min result in weak or oversaturated signals, respectively.

The heatmap analysis in Figure 1 reveals that the optimal conditions for the ELISA are achieved with a Coating Antigen concentration of 0.5 µg/mL, a Secondary Conjugate concentration of 1:5000, and a Substrate Incubation Time of 15 min, as indicated by the highest OD value. The 0.5 µg/mL coating concentration provides the optimal antigen density, ensuring effective antigen–antibody interaction without antigen-binding site saturation, which would otherwise reduce binding efficiency. The 0.2 µg/mL coating concentration is suboptimal because it is too low to generate a sufficiently strong antigen-antibody interaction. At this concentration, the antigen density on the plate is insufficient, resulting in weak antigen–antibody binding. This weak interaction leads to lower signal intensity, reducing the overall sensitivity of the assay. While lower concentrations may reduce oversaturation, they fail to provide sufficient antigen to facilitate efficient binding, and thus the OD values remain low. Higher concentrations, such as 1.0 µg/mL, lead to saturation of antigen sites, impeding overall assay performance. The 1:5000 dilution (concentration 1 ng/mL) of the secondary conjugate offers the ideal balance of signal amplification while avoiding the risk of nonspecific binding or oversaturation. Lower dilutions (e.g., 1:10,000, 1:20,000) result in insufficient secondary antibody for adequate signal amplification. The 15 min incubation period provides sufficient enzyme-substrate interaction to generate a signal, maximizing sensitivity and avoiding oversaturation or background noise. In contrast, a 10 min incubation is insufficient, yielding weak signals, whereas a 20 min incubation introduces signal oversaturation and background interference, reducing assay precision. This combination of conditions maximizes sensitivity and specificity, yielding the highest detectable signal and optimal assay performance.

Optimizing the indirect ELISA for detecting anti-Nipah virus IgG antibodies resulted in a highly reliable and reproducible assay. The optimal conditions, 0.5 µg/mL coating antigen, 1:5000 conjugate dilution, 1:24 serum dilution, and 15 min stop solution addition, provided the highest OD values while maintaining reagent efficiency and assay consistency.

These findings demonstrate that the optimized assay provides a robust, reproducible, and efficient platform for detecting Nipah virus IgG antibodies, which has significant potential for diagnostic and research applications.

*Qualification of the Assay*

To determine the performance criteria of the NiV IgG ELISA, qualification parameters were tested against the target acceptance criteria. Every requirement was fulfilled, thereby determining the reliability and accuracy of the test under the test conditions. The qualification outcomes are summarized in Table 2.

Following the optimization phase, the Anti-Nipah IgG ELISA was qualified across several key performance parameters: sensitivity, specificity, accuracy, Precision, Linearity, and detection limits. The qualification process confirmed that the assay meets the necessary standards for clinical diagnostics and future research applications [19].

### 3.1. Sensitivity and Specificity

The IgG-ELISA demonstrated 100% sensitivity (CI 94.96–100%) for positive samples and 100% specificity (CI 87.51–100%) for negative samples, highlighting the assay’s ability to distinguish true positives from true negatives, with minimal cross-reactivity and false positives (Table 3).

### 3.2. Intra- and Inter-Assay Precision

Overall, the assay method shows good precision for both intra- and inter-assay testing, with minor differences between analysts. For intra-assay precision, the geometric mean CV% for both analysts was 1.73 (0.36–3.97%) and 4.31 (1.54–9.25%), respectively, confirming reliability across runs and analysts (Table 4).

The precision results from both analysts demonstrate good reproducibility, with Inter-Assay Precision of 4.16 (1.03–8.98 CV %), which was within the acceptable range of up to 25% (Table 5).

### 3.3. Linearity

**Figure 2:**
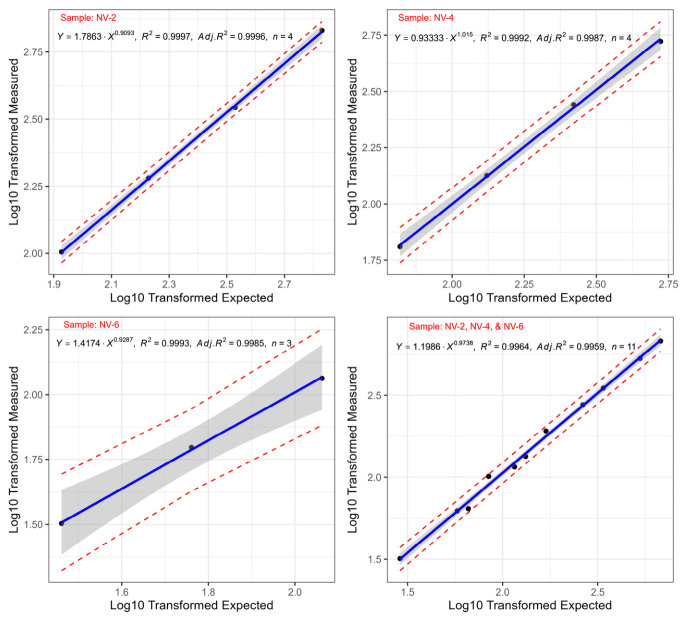
Transformed values (log10) of measured and expected values of NV-2, NV-4, and NV-6. The resulting plots demonstrate strong linear correlations among the variables, with an adjusted R^2^ of 0.9996, 0.9987, 0.9985, and 0.9959 for NV-2, NV-4, NV-6, and merged data, respectively. The shaded band around the blue line indicates the 95% confidence intervals, and the red-dashed lines indicate the 95% prediction intervals.

Linearity is a crucial attribute of the assay, as it demonstrates the potential to yield results proportional to antibody concentration over a given range [27]. The data proved to be highly linear, and the overall R^2^ value was 0.9964 (in the entire dataset) with a slope of 0.9738 y. These high R^2^ values support a strong linear association between the target antibody concentration and the measured OD values, indicating that the assay can quantitatively measure antibodies over a wide concentration range (31.9 to 676.3 IU/mL) in samples. The fitted regression lines, along with their equations and adjusted R^2^ values, also verify a very high level of linearity, thus confirming the accuracy of the assay performance over the entire measured concentration range (Figure 2).

### 3.4. Accuracy

The assay was shown to be reproducible, with accuracy between 82–100% across all test samples, and it has a wide dynamic range, confirming its ability to provide consistent and reliable measurements over a broad concentration range (Table 6).

### 3.5. Limit of Detection and Quantitation

**Figure 3:**
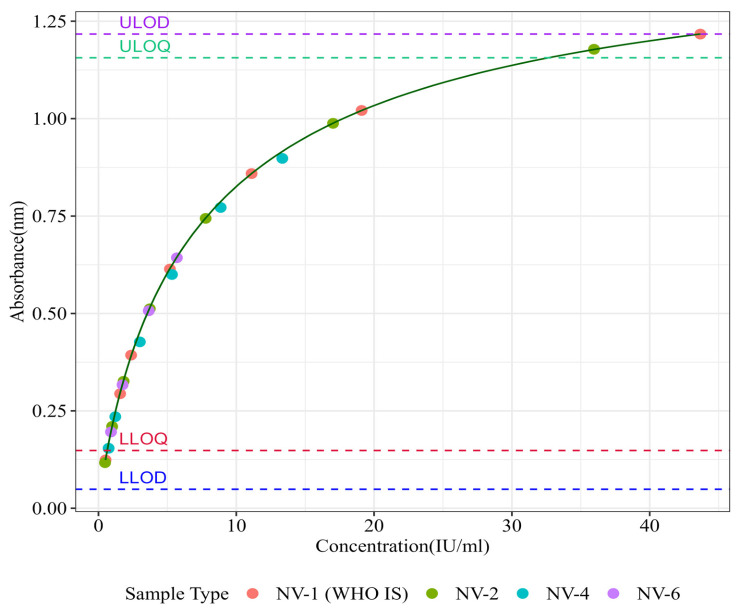
This figure illustrates the correlation between concentration (IU/mL) and absorbance (nm) during an ELISA assay, with the data fitted by a four-parameter logistic regression. The value represents the calculated lower limits of detection (LLOD) and lower limits of quantification (LLOQ), as well as the upper limits of detection (ULOD) and upper limits of quantification (ULOQ). The lower dashed blue and red lines indicate the LLOD and LLOQ, respectively, and the higher purple and green lines indicate the ULOD and ULOQ, respectively. The points in the data set represent specific sample types: NV-1 (WHO IS) (salmon), NV-2 (olive green), NV-4 (turquoise), and NV-6 (light purple), and indicate the response of each type at the given concentrations. Such values were developed in accordance with rules and ICH Q2 (R2) guidelines to ensure reasonable detection and quantification at specified concentrations.

The LLOQ and ULOQ were 0.98 IU/mL and 32.65 IU/mL, respectively, while the LLOD and ULOD were calculated to be 0.51 IU/mL and 43.65 IU/mL, respectively (Figure 3). With upper limits considering a maximum 24-fold sample dilution, these values indicate the concentrations at which accurate detection and quantification are feasible [20,21].

## 4. Discussion

This study describes the development and qualification of a colorimetric indirect ELISA for detecting IgG antibodies against the Nipah virus glycoprotein G. The assay demonstrated strong analytical performance, including 100% sensitivity and specificity with the tested serum panel, intra-assay CV values of 1.73% and 4.31%, inter-assay CV of 4.16%, good linearity, and low limits of detection and quantification. Together, these findings indicate that the assay is reproducible and suitable for measuring NiV-specific IgG responses under the tested conditions.

The selection of NiV glycoprotein G as the target antigen is appropriate because the G protein is involved in receptor binding and is an important target of neutralizing antibody responses in henipavirus infection and vaccine studies [12,13]. Previous studies have also used henipavirus glycoprotein-based serological assays for antibody detection, supporting the relevance of this antigenic target [7,8,28,29]. In the present assay, the use of recombinant glycoprotein G provides an important practical advantage because it avoids the need to work with live virus during routine antibody detection. This makes the platform more suitable for BSL-2 laboratory settings, especially in regions where high-containment facilities are not widely available [19].

Comparative sequence analyses of Nipah virus isolates have demonstrated high amino acid identity across major structural proteins between the Bangladesh and Malaysia lineages, including the attachment glycoprotein G. Specifically, the G protein shows approximately 95.5% amino acid identity between the two strains, with conserved structural domains relevant for antigenicity and receptor binding. This high sequence similarity supports the use of a recombinant G protein from one strain for broad serological detection without significantly affecting ELISA sensitivity across geographic variants [11].

The diagnostic performance observed in this study supports the potential usefulness of this ELISA for identifying previous NiV exposure and measuring antibody responses. The 100% sensitivity and specificity values should, however, be interpreted within the context of the sample panel used in this qualification study. Larger studies using more diverse serum panels will be necessary to confirm the assay’s performance across broader populations and field settings. Calibration against the WHO/NIBSC International Standard is an important strength because it supports standardized reporting of antibody concentrations and may allow comparison of results across laboratories and studies [21].

Precision is a key requirement for serological assays intended for surveillance or vaccine evaluation. The low intra-assay and inter-assay CV values observed in this study indicate that the assay produced consistent results across repeated measurements and testing conditions. The linearity results also suggest that the assay can provide proportional measurements of antibody concentration across the evaluated range. These findings are consistent with the principles of analytical qualification described in ICH guidelines and with general expectations for bioanalytical method validation [19,27].

The use of four-parameter logistic regression is appropriate for ELISA analysis because antibody-binding assays typically produce sigmoidal dose–response relationships rather than purely linear responses across the full analytical range [25]. In this study, the 4PL model enabled estimation of the lower and upper limits of detection and quantification. The calculated LLOD of 0.51 IU/mL and LLOQ of 0.98 IU/mL indicate that the assay can detect low levels of NiV-specific IgG under the tested conditions. These limits may be particularly useful when evaluating low antibody responses in convalescent individuals or in vaccine immunogenicity studies.

A major practical advantage of this assay is its compatibility with BSL-2 laboratory workflows. Nipah virus is a high-consequence pathogen, and live-virus assays such as virus isolation or plaque reduction neutralization testing require high-containment facilities. Although neutralization assays remain important for measuring functional antibody activity, they are less practical for routine surveillance in many endemic settings. By using recombinant antigen and heat-inactivated serum, the present ELISA provides a safer and more scalable alternative for antibody detection. Heat inactivation at 56 °C for 30 min has been reported as an effective approach for safer processing of NiV serum samples in lower-containment laboratories [20].

The assay may have several practical applications. First, it may support serosurveillance studies by helping identify previous NiV exposure in populations living in endemic or high-risk areas [22]. Second, it may be useful for outbreak investigations, particularly for identifying convalescent cases or estimating the extent of past exposure after an outbreak [5,22]. Third, the assay may support vaccine studies by providing a standardized method for measuring NiV G-specific IgG responses. Because candidate NiV vaccines often aim to generate antibody responses against henipavirus glycoproteins, a reproducible G-specific IgG ELISA may help evaluate vaccine-induced immune responses alongside functional neutralization assays [13,16].

However, important limitations should be recognized. An IgG-specific ELISA cannot reliably distinguish between acute infection, previous infection, or vaccine-induced antibody responses when used as a single test. In acute outbreak settings, IgM testing, RT-PCR, virus detection, or paired acute and convalescent sera may be required to support diagnosis and determine recent infection [8]. Therefore, this assay should be viewed primarily as a tool for serosurveillance, convalescent case confirmation, and vaccine immunogenicity assessment rather than as a stand-alone diagnostic test for acute NiV infection.

Another limitation is that binding IgG responses measured by ELISA do not always directly represent neutralizing antibody activity. Some antibodies may bind to glycoprotein G without fully preventing viral entry. For this reason, vaccine studies and detailed immunological investigations should use this ELISA together with neutralization assays when functional antibody activity is required [13,14]. Future studies should also evaluate possible cross-reactivity with related henipaviruses, including Hendra virus, because NiV and HeV glycoproteins share structural and antigenic relationships [12,17,29].

Further validation is needed before broad implementation. Future work should include larger panels of positive and negative sera, samples from different geographic regions, longitudinal samples from convalescent individuals, and specimens from vaccinated participants once such samples become available. Field validation in endemic settings will be especially important to assess real-world assay performance, reproducibility, and operational feasibility. Additional studies should also examine inter-laboratory comparability using the NV-1, WHO International Standard for anti-Nipah virus antibodies, provided by NIBSC, and evaluate how well ELISA-derived IgG concentrations correlate with neutralizing antibody titers [14,21].

Previous studies have described ELISAs targeting the Nipah virus glycoprotein G [7,8]; however, our assay offers several distinct advantages. It is calibrated to the WHO International Standard for anti-Nipah virus antibodies (NV-1), provided by NIBSC, enabling reporting of antibody concentrations in IU/mL and facilitating standardized comparisons across laboratories. The assay employs recombinant G protein in a BSL-2 compatible workflow, allowing safe and practical use without live viruses. Furthermore, it integrates parallel-line calibration and comprehensive analytical qualification, including sensitivity, specificity, precision, linearity, and detection limits. Designed for serosurveillance, outbreak investigations, and vaccine immunogenicity studies, this ELISA provides a reproducible, scalable, and standardized platform that expands the capabilities of previous NiV serological assays.

Overall, the developed NiV G-specific IgG ELISA provides a reproducible, BSL-2-compatible, and scalable platform for detecting human anti-NiV IgG antibodies. Its calibration against an international standard and its analytical performance support its potential use in serosurveillance, outbreak investigation, and vaccine immunogenicity assessment. With further field validation and cross-reactivity testing, this assay may contribute to strengthening NiV diagnostic and surveillance capacity in endemic regions.

## 5. Conclusions

The Anti-Nipah IgG ELISA has demonstrated excellent performance in sensitivity, specificity, accuracy, Precision, and Linearity, as established during qualification. The assay has high sensitivity (100%), specificity (100%), and precision (inter-assay CV of 4.16%), which account for its reliability and strength in Nipah virus antibody detection for clinical and research purposes. The NiV G IgG ELISA is an optimized assay that will contribute to the diagnosis of human henipavirus. It has high diagnostic accuracy, reproducibility, and scalability, making it appropriate for inclusion in public health systems in endemic areas. Future studies will focus on confirming the assay in the field, where it will be useful for large-scale human serosurveillance and for assessing the immunogenicity of candidate vaccines. Together, these efforts will enhance global capacity to identify, track, and treat NiV infections in humans.

## Figures and Tables

**Table 1:** Summary of serum sample used in the evaluation of in-house ELISA for Nipah virus. The table includes sample ID, description, expected result, and reference value (if available). NV = Nipah Virus, NHP = Nipah Human Positive, NC = Negative Control.

Sl. No.	Serum ID	Serum Description	Expected Category	Reference Value (IU/mL)
1	NV-1	WHO IS	POS	1000
2	NV-2	Reference Panel: High from Bangladesh	POS	687 (516–913)
3	NV-4	Collaborative Study Sample: High from Bangladesh	POS	541 (417–701)
4	NV-10	Reference Panel: Malaysia Pool	POS	256 (166–396)
5	NV-6	Collaborative Study Sample: Low from Malaysia	POS	140 (91–215)
6	NHP-1	Positive from Bangladesh	POS	-
7	NHP-3	Positive from Bangladesh	POS	-
8	NHP-5	Positive from Bangladesh	POS	-
9	NHP-6	Positive from Bangladesh	POS	-
10	NHP-7	Positive from Bangladesh	POS	-
11	NC-1	Negative Serum	NEG	N/A
12	NC-2	Negative Serum	NEG	N/A
13	NC-3	Negative Serum	NEG	N/A
14	NC-4	Negative Serum	NEG	N/A
15	NC-5	Negative Serum	NEG	N/A
16	NC-6	Negative Serum	NEG	N/A
17	NC-7	Negative Serum	NEG	N/A
18	NC-8	Negative Serum	NEG	N/A
19	CNC	Commercially purchased negative serum	NEG	N/A

**Table 2:** The acceptance standards of each parameter of the qualification of the Nipah IgG ELISA.

Parameter	Acceptance Criteria	Qualification	
		Outcome	Passed/Failed
Sensitivity	≥80%	100%	Passed
Specificity	≥80%	100%	Passed
Dilutional Linearity	Linear regression slope (observed geomean vs. expected) range 0.80–1.25 and R^2^ value ≥ 0.95	Slope = 1.1986 R^2^ = 0.964	Passed
Relative accuracy	Percent recovery 80–120%	95.05%	Passed
Intra-assay (Repeatability) Precision	Per Analyst (A1, A2) CV % < 25%	A1 = 1.73%, A2 = 4.31%	Passed
Inter assay (Intermediate) Precision	CV % < 25%	4.16%	Passed

**Table 3:** Sensitivity and Specificity of the ELISA.

Result	Expected	Observed	Sensitivity of Assay	Specificity of Assay
True Positive	10	10	100% (94.96–100%)	100% (87.51–100%)
True Negative	9	9		

**Table 4:** Intra-assay Precision (repeatability) was evaluated by two analysts (A1 and A2) with duplicate measurements across multiple runs. Analyst 1 showed a geometric mean CV % of 1.73%, while Analyst 2 had 4.31%. Both CV % values were well within the acceptable limit of <25%, confirming strong intra-assay precision.

**Analyst 1**				**Intra-Assay Precision/Repeatibility**			
**Sample**	**Run**	**Result 1**	**Result 2**	**MEAN (Duplicates)**	**CV%**	**Mean (Days)**	**CV%**
NV-1	Run 1	990.6		990.6	N/A	988.40	0.36
	Run 2	990.3		990.3			
	Run 3	984.3		984.3			
NV-2	Run 1	540.6	589.1	564.9	1.89	561.97	1.89
	Run 2	557.5	584.2	570.9	1.87		
	Run 3	537.9	562.5	550.2	1.94		
NV-10	Run 1	288.1	300.5	294.3	5.05	306.10	3.97
	Run 2	289.9	312.6	301.3	4.93		
	Run 3	310	335.5	322.8	4.60		
NV-4	Run 1	492.1	491.4	491.8	3.49	504.42	3.29
	Run 2	498	497	497.5	3.45		
	Run 3	523.3	524.7	524	3.28		
Acceptable range of %CV = up to 25%					Geometric mean of CV		1.73
					Minimum CV		0.36
					Maximum CV		3.97
**Analyst 2**				**Intra-Assay Precision/Repeatibility**			
**Sample**	**Run**	**Result 1**	**Result 2**	**MEAN (Duplicates)**	**CV%**	**Mean (Days)**	**CV%**
NV-1	Run 1	966.3		966.3	N/A	983.57	1.54
	Run 2	994.6		994.6			
	Run 3	989.8		989.8			
NV-2	Run 1	604.8	610.6	607.7	6.22	596.90	8.43
	Run 2	642.2	614.1	628.15	6.02		
	Run 3	542.6	567.1	554.85	6.82		
NV-10	Run 1	338.7	342.5	340.6	8.18	344.82	9.25
	Run 2	375.8	373.3	374.55	7.44		
	Run 3	312.3	326.3	319.3	8.73		
NV-4	Run 1	560.5	562.4	561.45	3.69	553.08	2.86
	Run 2	561.7	574.9	568.3	3.64		
	Run 3	533.7	525.3	529.5	3.91		
Acceptable range of %CV = up to 25%					Geometric mean of CV		4.31
					Minimum CV		1.54
					Maximum CV		9.25

**Table 5:** The inter-assay/intermediate precision was evaluated by analyzing variability across multiple days, analysts, and assay runs. Two analysts (A1 and A2) measured duplicate samples, and the geometric mean was calculated for each analyst on each day. The overall geometric mean of the coefficient of variation (GCV) was 4.16%, indicating excellent reproducibility. The % CV remained well within the acceptable range (<25%), demonstrating strong precision and minimal variability, confirming the assay’s robustness and reliability across different conditions.

Sample	Ref.Value	Run	Results								
			Analyst 1 (A1)		Average of Duplic	Analyst 2 (A2)		Average of Duplic	Geomean of A1 and A2 (Day Wise)	Geomean of A1 and A2	% CV
NV-1	1000	Run 1	990.6		990.6	966.3		966.3	978.37	985.94	1.03
		Run 2	990.3		990.3	994.6		994.6	992.45		
		Run 3	984.3		984.3	989.8		989.8	987.05		
NV-2	687 (516–913)	Run 1	540.6	589.1	564.85	604.8	610.6	607.7	585.88	578.74	5.42
		Run 2	557.5	584.2	570.85	642.2	614.1	628.15	598.82		
		Run 3	537.9	562.5	550.2	542.6	567.1	554.85	552.52		
NV-10	256 (166–396)	Run 1	288.1	300.5	294.3	338.7	342.5	340.6	316.6	324.41	8.98
		Run 2	289.9	312.6	301.25	375.8	373.3	374.55	335.91		
		Run 3	310.0	335.5	322.75	312.3	326.3	319.3	321.02		
NV-4	541 (417–701)	Run 1	492.1	491.4	491.75	560.5	562.4	561.45	525.45	527.96	5.99
		Run 2	498.0	497.0	497.5	561.7	574.9	568.3	531.72		
		Run 3	523.3	524.7	524.0	533.7	525.3	529.5	526.74		
							Geometric mean of CV				4.16
							Minimum CV				1.03
							Maximum CV				8.98
	Acceptable range of %CV = upto 25%										

**Table 6:** Relative accuracy of samples at varying dilution factors. The table presents the results from two analysts (Analyst 1 and Analyst 2), along with the geometric mean of all preparations, the expected titer, and the relative accuracy percentage (Geomean/Expected × 100) for three samples (NV-2, NV-4, NV-6).

Sample	Dilution Factor	Result Analyst 1	Result Analyst 2	Geomean of All Preparations	Expected Titer	Relative Accuracy % (Geomean/Expected × 100)
NV-2	24	20.19	19.85	20.02	20.02	100.00
	30	16.17	16.01	16.09	16.02	100.43
	60	8.04	7.96	8.00	8.01	99.89
	120	3.56	4.02	3.78	4.00	94.47
	480	1.04	0.98	1.01	1.00	100.85
	960	0.52	0.48	0.50	0.50	99.40
NV-4	24	13.42	13.43	13.42	13.42	100.00
	30	9.07	9.51	9.29	10.74	86.47
	60	4.69	4.64	4.66	5.37	86.87
	120	2.23	2.34	2.28	2.68	85.08
	480	0.64	0.69	0.66	0.67	99.00
	960	0.32	0.33	0.33	0.34	96.97
NV-6	24	3.03	3.03	3.03	3.03	100.00
	30	2.43	2.38	2.40	2.42	99.28
	60	1.04	1.21	1.12	1.21	92.75
	120	0.60	0.55	0.57	0.61	94.86
	480	0.12	0.13	0.13	0.15	82.84
	960	0.00	0.00	0.00	0.01	0.00
					GMR	95.05
					MAX	100.85
					MIN	82.84

## Data Availability

Data will be made available on request. Data are contained within the article and Supplementary Materials.

## Supplementary Materials

The following supporting information can be downloaded at https://www.mdpi.com/article/10.3390/vaccines14060534/s1.
