# Supplementary material for: Development and Qualification of a Nipah Virus Glycoprotein-Specific IgG ELISA for the Assessment of Human Antibody Responses
Source: Vaccines (Basel). 2026 Jun 16;14(6):534. doi: 10.3390/vaccines14060534 (PMC13307770; doi:10.3390/vaccines14060534)
Supplement: Supplementary file 1 [file vaccines-14-00534-s001.zip › Supplementary_ELISA Qualification Data & Graph/1. Sensitivity and Specificity_Analysist-1/4. Sensitivity and Specificity_NHP_Analyst-1_Day-1.pdf]

Intro

NIPAH_NHP_27 AUGUST_Analyst#1_Day#1

OD

	1	2	3	4	5	6	7	8	9	10	11	12
A	1.220	1.016	1.363	1.311	1.037	1.302	0.080	0.062	0.054	0.046	0.058	0.046
B	1.020	0.797	1.248	1.123	0.796	1.105	0.069	0.064	0.050	0.048	0.052	0.048
C	0.860	0.677	1.160	0.936	0.627	0.916	0.063	0.058	0.048	0.048	0.049	0.048
D	0.621	0.480	0.967	0.743	0.423	0.736	0.058	0.042	0.049	0.046	0.043	0.046
E	0.398	0.305	0.762	0.481	0.261	0.473	0.052	0.049	0.044	0.048	0.039	0.048
F	0.293	0.185	0.536	0.291	0.160	0.287	0.051	0.049	0.047	0.046	0.037	0.046
G	0.123	0.126	0.341	0.203	0.108	0.194	0.048	0.042	0.046	0.045	0.043	0.045
H	0.091	0.084	0.210	0.120	0.071	0.114	0.045	0.051	0.042	0.044	0.045	0.044

Settings Information
Endpoint
Absorbance
Lm1 450
More Settings
Shake Off
Calibrate On
Carriage Speed Normal
Column Priority

Read Information
Imported Data : 4:33 PM 9/2/2024
Imported By : anjan

Reduction Settings

Optical Density
Wavelength Combination : !Lm1

Sample Dil

Main Sample Dilution 24.0

Sample 1: NHP-1 24.0

Sample 2: NHP-3 24.0

Sample 3: NHP-5 24.0

Sample 4: NHP-6 24.0

Sample 5: NHP-7 24.0

Sample 6: NC-5 24.0

Sample 7: NC-6 24.0

Sample 8: NC-7 24.0

Sample 9: NC-8 24.0

Sample 10: CNC 24.0

Sample 11: BLANK 24.0

Standards

Sample	Wells	OD	OK OD	Dilution	Calc.Conc	Adj.Conc	GMC	N	Th.Conc	RelErr%
01	A1	1.220	1.220	24	44.011	1056.3	983.0	7	41.700	5.500
	B1	1.020	1.020	48	18.910	907.7			20.800	-9.100
	C1	0.860	0.860	96	11.069	1062.6			10.400	6.400
	D1	0.621	0.621	192	5.245	1007.0			5.200	0.900
	E1	0.398	0.398	384	2.423	930.5			2.600	-6.800
	F1	0.293	0.293	768	1.542	1184.3			1.300	18.600
	G1	0.123	0.123	1536	0.511	784.4			0.700	-27.000
	H1	0.091		3072					0.300	

Samples

Sample	Wells	ID	OD	OK OD	Dilution	Calc.Conc	Adjusted.Conc	GMC	N	CVdil
01	A2	1	1.016	1.016	24	18.641	447.381	587.0	7	21.9
	B2		0.797	0.797	48	9.081	435.871			
	C2		0.677	0.677	96	6.256	600.563			
	D2		0.480	0.480	192	3.282	630.190			
	E2		0.305	0.305	384	1.632	626.697			
	F2		0.185	0.185	768	0.837	643.198			
	G2		0.126	0.126	1536	0.525	806.780			
	H2		0.084		3072					
02	A3	2	1.363	1.363	24	117.817	2827.608	2944.6	8	8.1
	B3		1.248	1.248	48	51.213	2458.236			
	C3		1.160	1.160	96	32.976	3165.727			
	D3		0.967	0.967	192	15.715	3017.376			
	E3		0.762	0.762	384	8.145	3127.667			
	F3		0.536	0.536	768	3.977	3054.558			
	G3		0.341	0.341	1536	1.918	2945.823			
	H3		0.210	0.210	3072	0.985	3024.831			
03	A4	3	1.311	1.311	24	76.446	1834.714	1416.9	8	13.5
	B4		1.123	1.123	48	28.108	1349.185			
	C4		0.936	0.936	96	14.161	1359.443			
	D4		0.743	0.743	192	7.679	1474.401			
	E4		0.481	0.481	384	3.294	1264.824			
	F4		0.291	0.291	768	1.527	1172.916			
	G4		0.203	0.203	1536	0.943	1447.719			
	H4		0.120	0.120	3072	0.496	1524.262			
04	A5	4	1.037	1.037	24	20.112	482.679	518.6	7	13.5
	B5		0.796	0.796	48	9.052	434.516			
	C5		0.627	0.627	96	5.346	513.199			
	D5		0.423	0.423	192	2.667	512.101			
	E5		0.261	0.261	384	1.314	504.573			
	F5		0.160	0.160	768	0.699	537.052			
	G5		0.108	0.108	1536	0.440	675.101			
	H5		0.071		3072					
05	A6	5	1.302	1.302	24	71.729	1721.495	1349.4	8	12.6
	B6		1.105	1.105	48	26.108	1253.175			
	C6		0.916	0.916	96	13.257	1272.707			
	D6		0.736	0.736	192	7.514	1442.766			
	E6		0.473	0.473	384	3.202	1229.569			
	F6		0.287	0.287	768	1.498	1150.405			
	G6		0.194	0.194	1536	0.889	1366.158			
	H6		0.114	0.114	3072	0.468	1436.475			
06	A7	6	0.080		24			N/A	0	-----
	B7		0.069		48					
	C7		0.063		96					
	D7		0.058		192					
	E7		0.052		384					
	F7		0.051		768					
	G7		0.048		1536					
	H7		0.045		3072					
07	A8	7	0.062		24			N/A	0	-----
	B8		0.064		48					
	C8		0.058		96					
	D8		0.042		192					
	E8		0.049		384					
	F8		0.049		768					
	G8		0.042		1536					
	H8		0.051		3072					
08	A9	8	0.054		24			N/A	0	-----
	B9		0.050		48					
	C9		0.048		96					
	D9		0.049		192					

Samples (Contd)

Sample	Wells	ID	OD	OK OD	Dilution	Calc.Conc	Adjusted.Conc	GMC	N	CVdil
	E9		0.044		384					
	F9		0.047		768					
	G9		0.046		1536					
	H9		0.042		3072					
09	A10	9	0.046		24			N/A	0	-----
	B10		0.048		48					
	C10		0.048		96					
	D10		0.046		192					
	E10		0.048		384					
	F10		0.046		768					
	G10		0.045		1536					
	H10		0.044		3072					
10	A11	10	0.058		24			N/A	0	-----
	B11		0.052		48					
	C11		0.049		96					
	D11		0.043		192					
	E11		0.039		384					
	F11		0.037		768					
	G11		0.043		1536					
	H11		0.045		3072					
11	A12	11	0.046		24			N/A	0	-----
	B12		0.048		48					
	C12		0.048		96					
	D12		0.046		192					
	E12		0.048		384					
	F12		0.046		768					
	G12		0.045		1536					
	H12		0.044		3072					

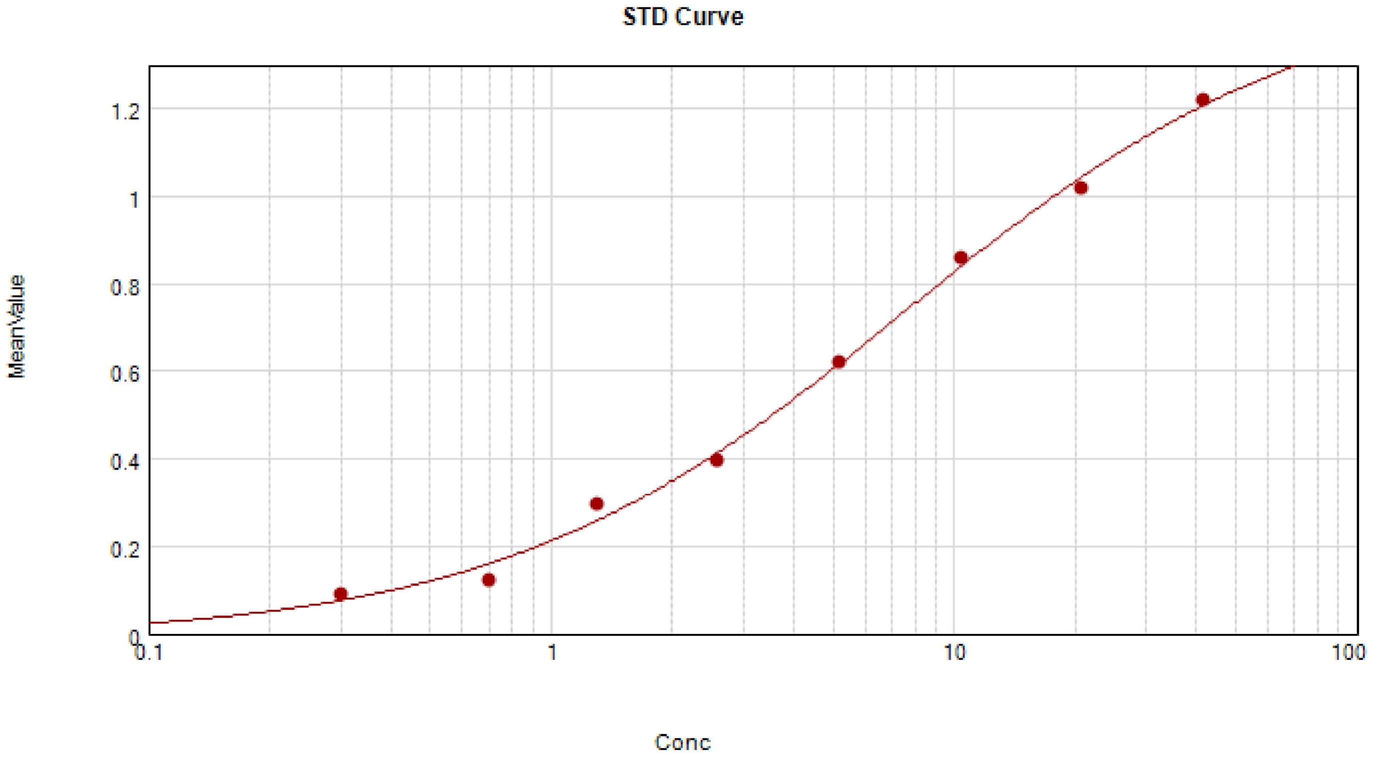

● **Std** (Standards: OD vs Th.Conc) Weighting: Fixed

Curve Fit Results ▲

Curve Fit : 4-Parameter Logistic $y = D + \frac{A - D}{1 + \left(\frac{x}{C}\right)^B}$

	Parameter	Estimated Value	Std. Error	Confidence Interval
**Std**<br>$R^2 = 0.997$<br>EC50 = 7.643	A	-0.012	0.074	[-0.217, 0.193]
	B	0.856	0.183	[0.350, 1.363]
	C	7.643	1.918	[2.318, 12.97]
	D	1.495	0.176	[1.005, 1.985]

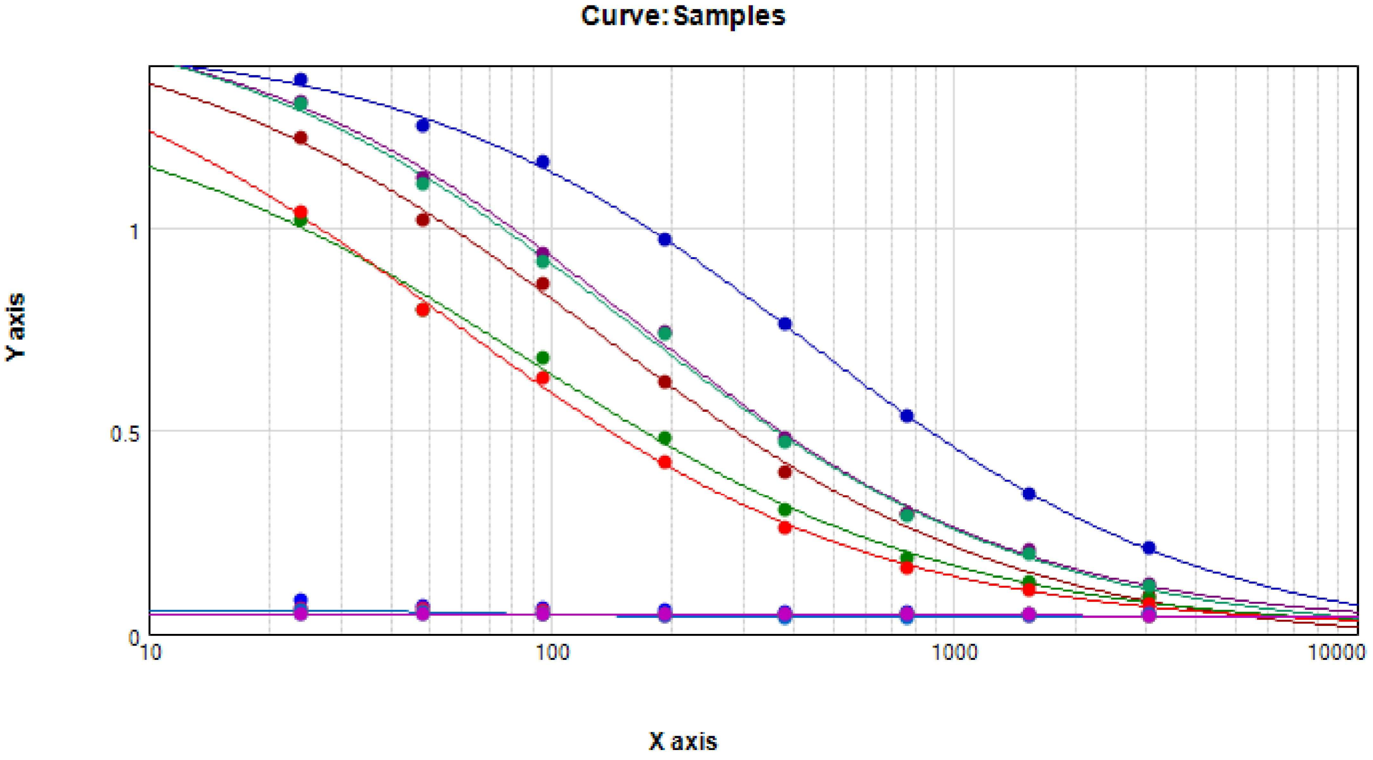

- **STD** (Standards: OD vs Dilution) Weighting: Fixed
- **S-1** (Samples: ODS1 vs DilSple1) Weighting: Fixed
- **S-2** (Samples: ODS2 vs DilSple2) Weighting: Fixed
- **S-3** (Samples: ODS3 vs DilSple3) Weighting: Fixed
- **S-4** (Samples: ODS4 vs DilSple4) Weighting: Fixed
- **S-5** (Samples: ODS5 vs DilSple5) Weighting: Fixed
- **S-6** (Samples: ODS6 vs DilSple6) Weighting: Fixed
- **S-7** (Samples: ODS7 vs DilSple7) Weighting: Fixed
- **S-8** (Samples: ODS8 vs DilSple8) Weighting: Fixed
- **S-9** (Samples: ODS9 vs DilSple9) Weighting: Fixed
- **S-10** (Samples: ODS10 vs DilSple10) Weighting: Fixed
- **S-11** (Samples: ODS11 vs DilSple11) Weighting: Fixed

Curve Fit Results ▼

Assay Parameter

Samples

Theoretical First Dilution Of Test Sample In Plate : 24.0 Sample dilution fold: 2.0

Nipha_Standard : NV-1

Concentration: 1000.0

Dilution (First dil in plate): 24.0

Dilution fold: 2.0

Others parameters

Rounding Decimal Standard Th.Conc: 1

Rounding Decimal RelErr% & CVdil: 1

Rounding Decimal GMC: 1

Average ODs of Blank: 0.046

SD of Blank: 0.002

Cutoff OD: 0.094
