# Supplementary material for: Development and Qualification of a Nipah Virus Glycoprotein-Specific IgG ELISA for the Assessment of Human Antibody Responses
Source: Vaccines (Basel). 2026 Jun 16;14(6):534. doi: 10.3390/vaccines14060534 (PMC13307770; doi:10.3390/vaccines14060534)
Supplement: Supplementary file 1 [file vaccines-14-00534-s001.zip › Supplementary_ELISA Qualification Data & Graph/1. Sensitivity and Specificity_Analysist-1/5. Sensitivity and Specificity_NHP_Analyst-1_Day-2.pdf]

Intro

NIPAH_NHP_28 AUGUST_Analyst#1_Day#2

OD

	1	2	3	4	5	6	7	8	9	10	11	12
A	1.227	1.018	1.363	1.321	1.042	1.311	0.063	0.053	0.072	0.077	0.043	0.044
B	1.018	0.787	1.256	1.128	0.803	1.112	0.055	0.056	0.065	0.062	0.047	0.045
C	0.859	0.681	1.153	0.932	0.631	0.920	0.046	0.050	0.060	0.054	0.043	0.046
D	0.628	0.493	0.958	0.738	0.425	0.740	0.045	0.042	0.050	0.047	0.040	0.043
E	0.342	0.311	0.766	0.487	0.264	0.476	0.039	0.042	0.047	0.042	0.043	0.045
F	0.299	0.191	0.542	0.294	0.172	0.282	0.046	0.046	0.044	0.038	0.039	0.048
G	0.130	0.131	0.337	0.212	0.112	0.198	0.044	0.039	0.040	0.041	0.044	0.046
H	0.092	0.086	0.204	0.132	0.075	0.108	0.046	0.048	0.039	0.045	0.041	0.047

Settings Information
Endpoint
Absorbance
Lm1 450
More Settings
Shake Off
Calibrate On
Carriage Speed Normal
Column Priority

Read Information
Imported Data : 4:35 PM 9/2/2024
Imported By : anjan

Reduction Settings

Optical Density
Wavelength Combination : !Lm1

Sample Dil

Main Sample Dilution 24.0

Sample 1: NHP-1 24.0

Sample 2: NHP-3 24.0

Sample 3: NHP-5 24.0

Sample 4: NHP-6 24.0

Sample 5: NHP-7 24.0

Sample 6: NC-5 24.0

Sample 7: NC-6 24.0

Sample 8: NC-7 24.0

Sample 9: NC-8 24.0

Sample 10: CNC 24.0

Sample 11: BLANK 24.0

Standards

Sample	Wells	OD	OK OD	Dilution	Calc.Conc	Adj.Conc	GMC	N	Th.Conc	RelErr%
01	A1	1.227	1.227	24	44.732	1073.6	991.5	7	41.700	7.300
	B1	1.018	1.018	48	18.496	887.8			20.800	-11.100
	C1	0.859	0.859	96	11.098	1065.4			10.400	6.700
	D1	0.628	0.628	192	5.580	1071.3			5.200	7.300
	E1	0.342	0.342	384	2.070	795.0			2.600	-20.400
	F1	0.299	0.299	768	1.706	1310.5			1.300	31.300
	G1	0.130	0.130	1536	0.541	831.3			0.700	-22.700
	H1	0.092		3072					0.300	

Samples

Sample	Wells	ID	OD	OK OD	Dilution	Calc.Conc	Adjusted.Conc	GMC	N	CVdil
01	A2	1	1.018	1.018	24	18.496	443.904	617.8	7	25.6
	B2		0.787	0.787	48	8.947	429.448			
	C2		0.681	0.681	96	6.540	627.809			
	D2		0.493	0.493	192	3.641	698.999			
	E2		0.311	0.311	384	1.805	693.038			
	F2		0.191	0.191	768	0.918	704.729			
	G2		0.131	0.131	1536	0.547	840.246			
	H2		0.086		3072					
02	A3	2	1.363	1.363	24	127.931	3070.334	3018.3	8	8.0
	B3		1.256	1.256	48	52.859	2537.208			
	C3		1.153	1.153	96	31.181	2993.418			
	D3		0.958	0.958	192	15.127	2904.317			
	E3		0.766	0.766	384	8.408	3228.628			
	F3		0.542	0.542	768	4.273	3281.798			
	G3		0.337	0.337	1536	2.026	3112.607			
	H3		0.204	0.204	3072	1.004	3084.072			
03	A4	3	1.321	1.321	24	84.090	2018.166	1503.2	8	15.6
	B4		1.128	1.128	48	28.018	1344.854			
	C4		0.932	0.932	96	13.915	1335.830			
	D4		0.738	0.738	192	7.741	1486.240			
	E4		0.487	0.487	384	3.568	1370.083			
	F4		0.294	0.294	768	1.666	1279.470			
	G4		0.212	0.212	1536	1.058	1625.325			
	H4		0.132	0.132	3072	0.553	1698.345			
04	A5	4	1.042	1.042	24	20.128	483.077	547.1	7	13.6
	B5		0.803	0.803	48	9.382	450.335			
	C5		0.631	0.631	96	5.630	540.517			
	D5		0.425	0.425	192	2.871	551.163			
	E5		0.264	0.264	384	1.432	549.945			
	F5		0.172	0.172	768	0.795	610.861			
	G5		0.112	0.112	1536	0.439	674.185			
	H5		0.075		3072					
05	A6	5	1.311	1.311	24	77.430	1858.319	1385.5	8	14.2
	B6		1.112	1.112	48	26.241	1259.579			
	C6		0.920	0.920	96	13.397	1286.099			
	D6		0.740	0.740	192	7.787	1495.038			
	E6		0.476	0.476	384	3.437	1319.871			
	F6		0.282	0.282	768	1.571	1206.357			
	G6		0.198	0.198	1536	0.964	1480.424			
	H6		0.108	0.108	3072	0.417	1280.173			
06	A7	6	0.063		24			N/A	0	-----
	B7		0.055		48					
	C7		0.046		96					
	D7		0.045		192					
	E7		0.039		384					
	F7		0.046		768					
	G7		0.044		1536					
	H7		0.046		3072					
07	A8	7	0.053		24			N/A	0	-----
	B8		0.056		48					
	C8		0.050		96					
	D8		0.042		192					
	E8		0.042		384					
	F8		0.046		768					
	G8		0.039		1536					
	H8		0.048		3072					
08	A9	8	0.072		24			N/A	0	-----
	B9		0.065		48					
	C9		0.060		96					
	D9		0.050		192					

Samples (Contd)

Sample	Wells	ID	OD	OK OD	Dilution	Calc.Conc	Adjusted.Conc	GMC	N	CVdil
	E9		0.047		384					
	F9		0.044		768					
	G9		0.040		1536					
	H9		0.039		3072					
09	A10	9	0.077		24			N/A	0	-----
	B10		0.062		48					
	C10		0.054		96					
	D10		0.047		192					
	E10		0.042		384					
	F10		0.038		768					
	G10		0.041		1536					
	H10		0.045		3072					
10	A11	10	0.043		24			N/A	0	-----
	B11		0.047		48					
	C11		0.043		96					
	D11		0.040		192					
	E11		0.043		384					
	F11		0.039		768					
	G11		0.044		1536					
	H11		0.041		3072					
11	A12	11	0.044		24			N/A	0	-----
	B12		0.045		48					
	C12		0.046		96					
	D12		0.043		192					
	E12		0.045		384					
	F12		0.048		768					
	G12		0.046		1536					
	H12		0.047		3072					

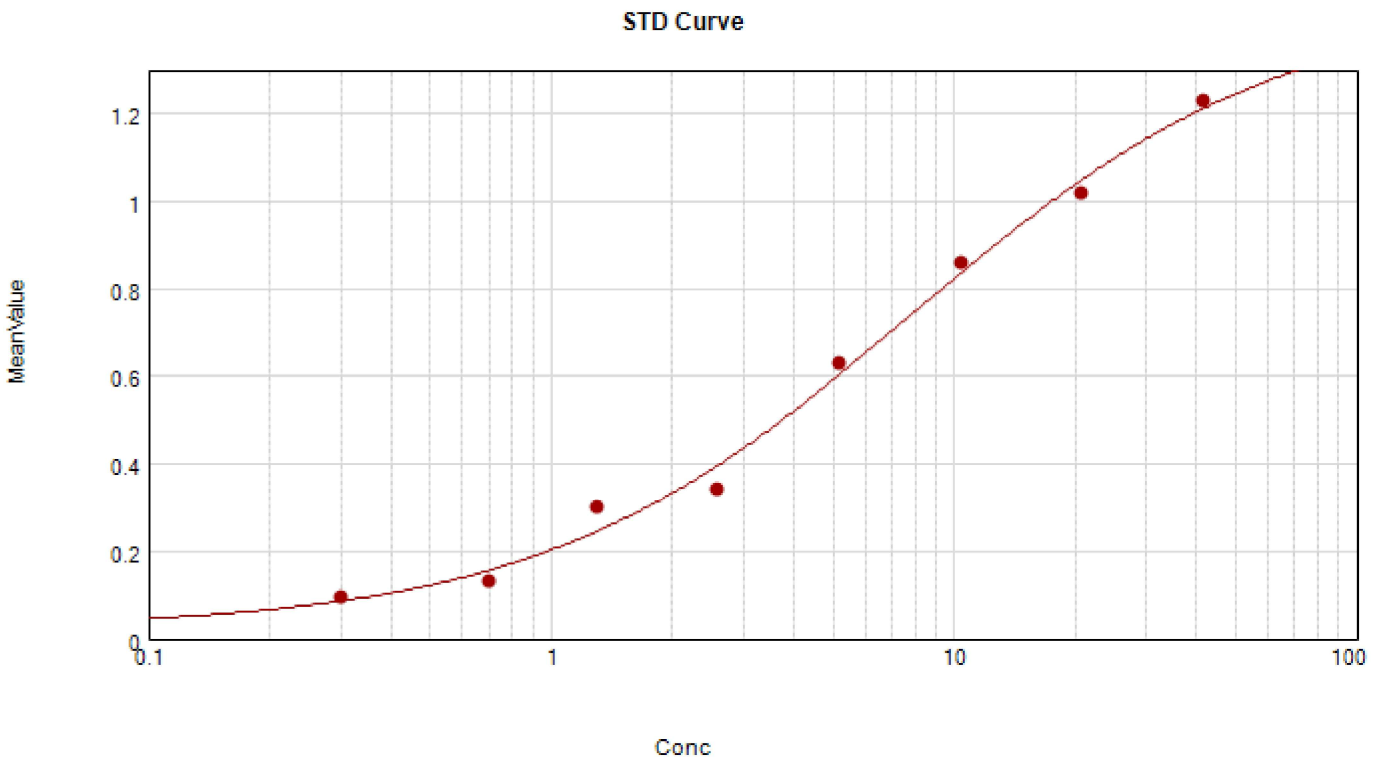

● Std (Standards: OD vs Th.Conc) Weighting: Fixed

Curve Fit Results ▲

Curve Fit : 4-Parameter Logistic $y = D + \frac{A - D}{1 + \left(\frac{x}{C}\right)^B}$

	Parameter	Estimated Value	Std. Error	Confidence Interval
**Std**<br>R^2 = 0.993<br>EC50 = 7.806	A	0.023	0.086	[-0.215, 0.262]
	B	0.944	0.263	[0.213, 1.676]
	C	7.806	2.424	[1.075, 14.54]
	D	1.459	0.214	[0.865, 2.052]

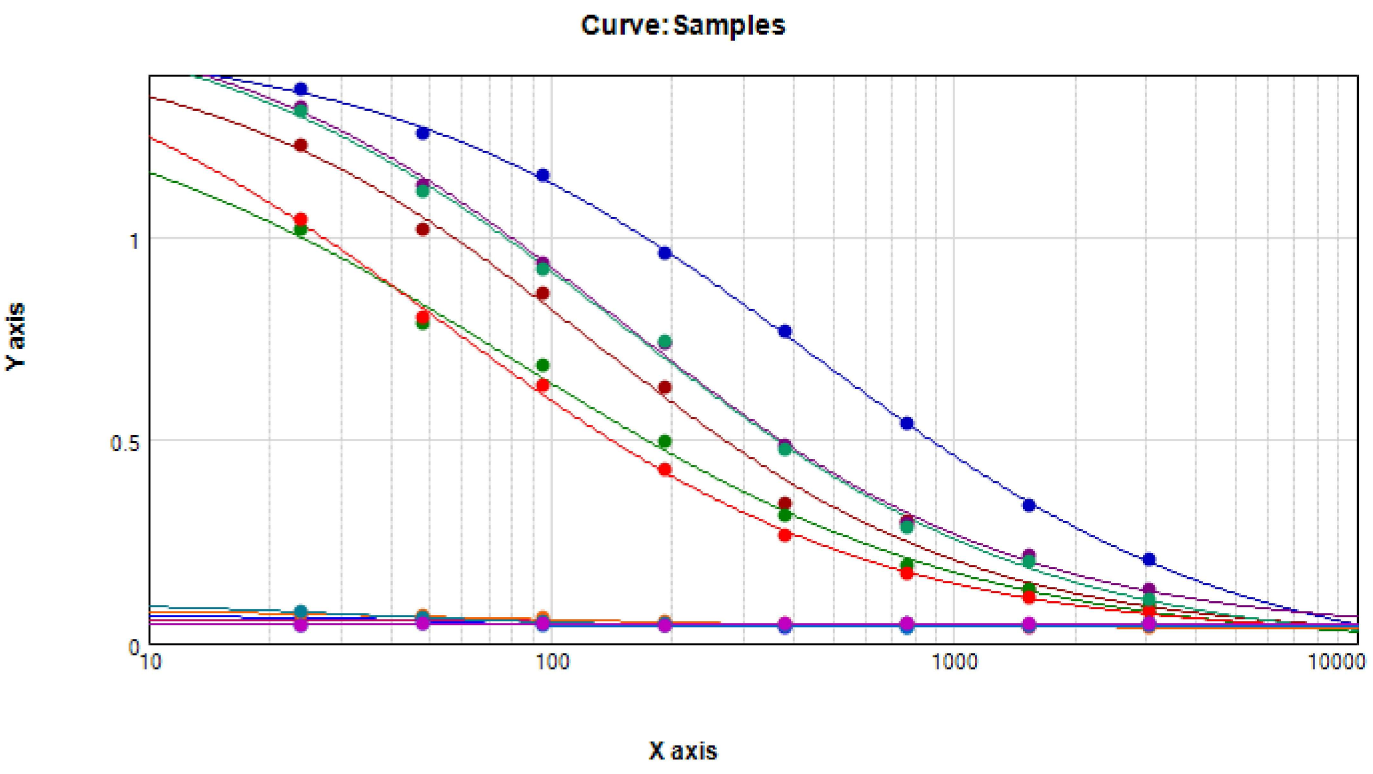

- **STD** (Standards: OD vs Dilution) Weighting: Fixed
- **S-1** (Samples: ODS1 vs DilSple1) Weighting: Fixed
- **S-2** (Samples: ODS2 vs DilSple2) Weighting: Fixed
- **S-3** (Samples: ODS3 vs DilSple3) Weighting: Fixed
- **S-4** (Samples: ODS4 vs DilSple4) Weighting: Fixed
- **S-5** (Samples: ODS5 vs DilSple5) Weighting: Fixed
- **S-6** (Samples: ODS6 vs DilSple6) Weighting: Fixed
- **S-7** (Samples: ODS7 vs DilSple7) Weighting: Fixed
- **S-8** (Samples: ODS8 vs DilSple8) Weighting: Fixed
- **S-9** (Samples: ODS9 vs DilSple9) Weighting: Fixed
- **S-10** (Samples: ODS10 vs DilSple10) Weighting: Fixed
- **S-11** (Samples: ODS11 vs DilSple11) Weighting: Fixed

Curve Fit Results ▼

Assay Parameter

Samples

Theoretical First Dilution Of Test Sample In Plate : 24.0   Sample dilution fold: 2.0

Nipha_Standard : NV-1

Concentration: 1000.0

Dilution (First dil in plate): 24.0

Dilution fold: 2.0

Others parameters

Rounding Decimal Standard Th.Conc: 1

Rounding Decimal RelErr% & CVdil: 1

Rounding Decimal GMC: 1

Average ODs of Blank: 0.045

SD of Blank: 0.002

Cutoff OD: 0.093
