# Supplementary material for: Development and Qualification of a Nipah Virus Glycoprotein-Specific IgG ELISA for the Assessment of Human Antibody Responses
Source: Vaccines (Basel). 2026 Jun 16;14(6):534. doi: 10.3390/vaccines14060534 (PMC13307770; doi:10.3390/vaccines14060534)
Supplement: Supplementary file 1 [file vaccines-14-00534-s001.zip › Supplementary_ELISA Qualification Data & Graph/1. Sensitivity and Specificity_Analysist-1/6. Sensitivity and Specificity_NHP_Analyst-1_Day-3.pdf]

Intro

NIPAH_NHP_29 AUGUST_Analyst#1_Day#3

OD

	1	2	3	4	5	6	7	8	9	10	11	12
A	1.223	1.012	1.370	1.319	1.047	1.308	0.058	0.057	0.062	0.068	0.060	0.046
B	1.023	0.790	1.263	1.124	0.810	1.109	0.043	0.048	0.062	0.057	0.046	0.048
C	0.863	0.686	1.149	0.935	0.637	0.924	0.042	0.046	0.049	0.056	0.045	0.048
D	0.630	0.497	0.960	0.733	0.426	0.746	0.039	0.044	0.042	0.045	0.043	0.046
E	0.345	0.318	0.771	0.482	0.264	0.473	0.036	0.042	0.042	0.042	0.040	0.048
F	0.289	0.187	0.539	0.296	0.176	0.287	0.040	0.040	0.045	0.041	0.043	0.046
G	0.121	0.126	0.340	0.209	0.108	0.187	0.038	0.042	0.044	0.040	0.039	0.045
H	0.090	0.089	0.210	0.138	0.085	0.111	0.038	0.037	0.037	0.037	0.037	0.044

Settings Information

Endpoint

Absorbance

Lm1 450

More Settings

Shake Off

Calibrate On

Carriage Speed Normal

Column Priority

Read Information

Imported Data : 4:37 PM 9/2/2024

Imported By : anjan

Reduction Settings

Optical Density

Wavelength Combination : !Lm1

Sample Dil

Main Sample Dilution 24.0

Sample 1: NHP-1 24.0

Sample 2: NHP-3 24.0

Sample 3: NHP-5 24.0

Sample 4: NHP-6 24.0

Sample 5: NHP-7 24.0

Sample 6: NC-5 24.0

Sample 7: NC-6 24.0

Sample 8: NC-7 24.0

Sample 9: NC-8 24.0

Sample 10: CNC 24.0

Sample 11: BLANK 24.0

Standards

Sample	Wells	OD	OK OD	Dilution	Calc.Conc	Adj.Conc	GMC	N	Th.Conc	RelErr%
01	A1	1.223	1.223	24	45.124	1083.0	987.5	7	41.700	8.200
	B1	1.023	1.023	48	18.491	887.6			20.800	-11.100
	C1	0.863	0.863	96	11.003	1056.3			10.400	5.800
	D1	0.630	0.630	192	5.557	1066.9			5.200	6.900
	E1	0.345	0.345	384	2.136	820.3			2.600	-17.800
	F1	0.289	0.289	768	1.671	1283.0			1.300	28.500
	G1	0.121	0.121	1536	0.523	803.4			0.700	-25.300
	H1	0.090		3072					0.300	

Samples

Sample	Wells	ID	OD	OK OD	Dilution	Calc.Conc	Adjusted.Conc	GMC	N	CVdil
01	A2	1	1.012	1.012	24	17.787	426.889	622.1	7	27.8
	B2		0.790	0.790	48	8.859	425.215			
	C2		0.686	0.686	96	6.547	628.496			
	D2		0.497	0.497	192	3.693	709.045			
	E2		0.318	0.318	384	1.906	731.771			
	F2		0.187	0.187	768	0.935	717.808			
	G2		0.126	0.126	1536	0.553	848.840			
	H2		0.089		3072					
02	A3	2	1.370	1.370	24	200.500	4812.000	3261.7	8	17.1
	B3		1.263	1.263	48	58.631	2814.288			
	C3		1.149	1.149	96	30.668	2944.143			
	D3		0.960	0.960	192	14.916	2863.943			
	E3		0.771	0.771	384	8.380	3218.060			
	F3		0.539	0.539	768	4.220	3240.806			
	G3		0.340	0.340	1536	2.093	3214.325			
	H3		0.210	0.210	3072	1.089	3346.323			
03	A4	3	1.319	1.319	24	95.524	2292.579	1547.0	8	21.1
	B4		1.124	1.124	48	27.423	1316.313			
	C4		0.935	0.935	96	13.757	1320.655			
	D4		0.733	0.733	192	7.504	1440.760			
	E4		0.482	0.482	384	3.516	1350.300			
	F4		0.296	0.296	768	1.726	1325.707			
	G4		0.209	0.209	1536	1.082	1662.636			
	H4		0.138	0.138	3072	0.625	1919.100			
04	A5	4	1.047	1.047	24	20.173	484.158	559.3	7	15.3
	B5		0.810	0.810	48	9.395	450.942			
	C5		0.637	0.637	96	5.673	544.580			
	D5		0.426	0.426	192	2.905	557.813			
	E5		0.264	0.264	384	1.478	567.441			
	F5		0.176	0.176	768	0.863	662.637			
	G5		0.108	0.108	1536	0.447	686.907			
	H5		0.085		3072					
05	A6	5	1.308	1.308	24	85.369	2048.854	1419.4	8	16.5
	B6		1.109	1.109	48	25.728	1234.931			
	C6		0.924	0.924	96	13.284	1275.257			
	D6		0.746	0.746	192	7.793	1496.167			
	E6		0.473	0.473	384	3.413	1310.676			
	F6		0.287	0.287	768	1.655	1270.911			
	G6		0.187	0.187	1536	0.935	1435.616			
	H6		0.111	0.111	3072	0.465	1427.112			
06	A7	6	0.058		24			N/A	0	-----
	B7		0.043		48					
	C7		0.042		96					
	D7		0.039		192					
	E7		0.036		384					
	F7		0.040		768					
	G7		0.038		1536					
	H7		0.038		3072					
07	A8	7	0.057		24			N/A	0	-----
	B8		0.048		48					
	C8		0.046		96					
	D8		0.044		192					
	E8		0.042		384					
	F8		0.040		768					
	G8		0.042		1536					
	H8		0.037		3072					
08	A9	8	0.062		24			N/A	0	-----
	B9		0.062		48					
	C9		0.049		96					
	D9		0.042		192					

Samples (Contd)

Sample	Wells	ID	OD	OK OD	Dilution	Calc.Conc	Adjusted.Conc	GMC	N	CVdil
	E9		0.042		384					
	F9		0.045		768					
	G9		0.044		1536					
	H9		0.037		3072					
09	A10	9	0.068		24			N/A	0	-----
	B10		0.057		48					
	C10		0.056		96					
	D10		0.045		192					
	E10		0.042		384					
	F10		0.041		768					
	G10		0.040		1536					
	H10		0.037		3072					
10	A11	10	0.060		24			N/A	0	-----
	B11		0.046		48					
	C11		0.045		96					
	D11		0.043		192					
	E11		0.040		384					
	F11		0.043		768					
	G11		0.039		1536					
	H11		0.037		3072					
11	A12	11	0.046		24			N/A	0	-----
	B12		0.048		48					
	C12		0.048		96					
	D12		0.046		192					
	E12		0.048		384					
	F12		0.046		768					
	G12		0.045		1536					
	H12		0.044		3072					

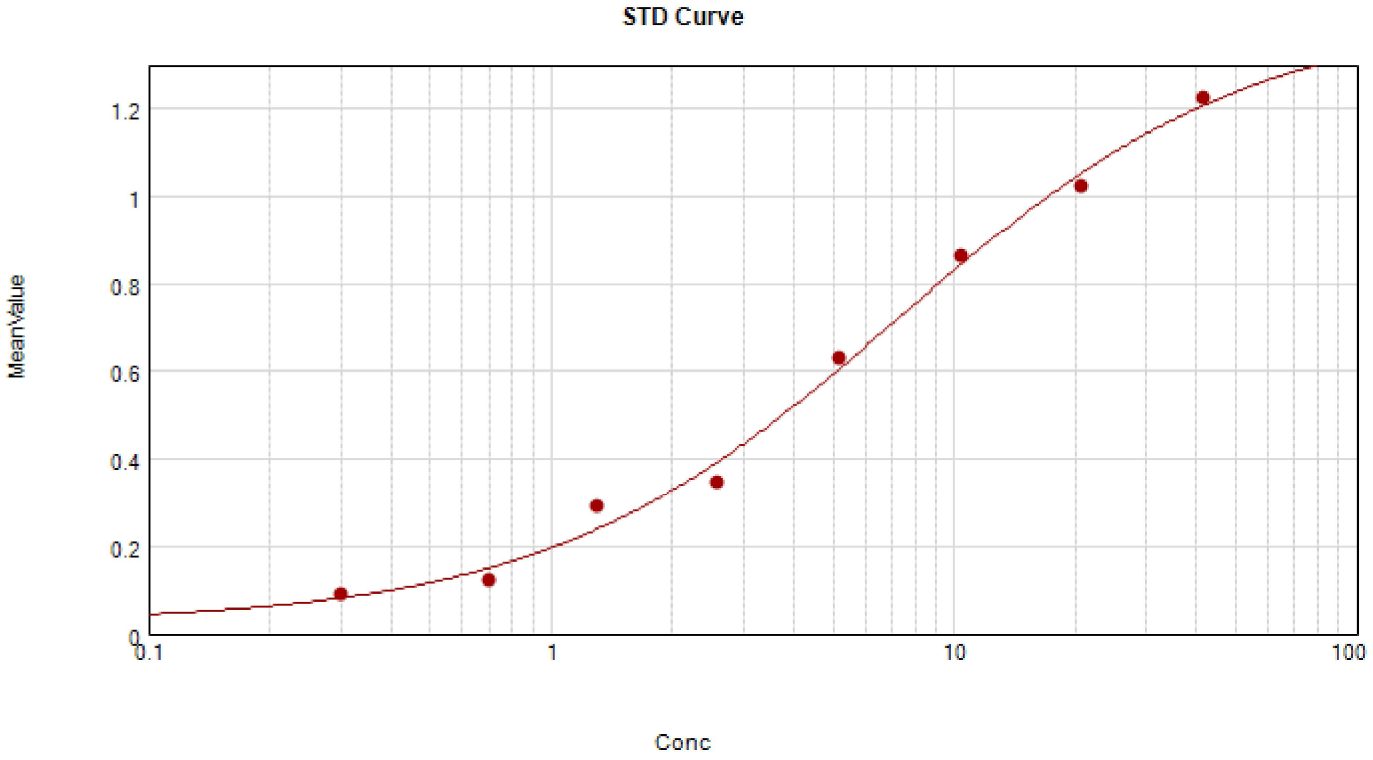

● Std (Standards: OD vs Th.Conc) Weighting: Fixed

Curve Fit Results ▲

Curve Fit : 4-Parameter Logistic $y = D + \frac{A - D}{1 + \left(\frac{x}{C}\right)^B}$

	Parameter	Estimated Value	Std. Error	Confidence Interval
**Std**<br>R^2 = 0.994<br>EC50 = 7.284	A	0.024	0.076	[-0.186, 0.235]
	B	0.987	0.246	[0.305, 1.669]
	C	7.284	1.863	[2.112, 12.46]
	D	1.421	0.174	[0.938, 1.904]

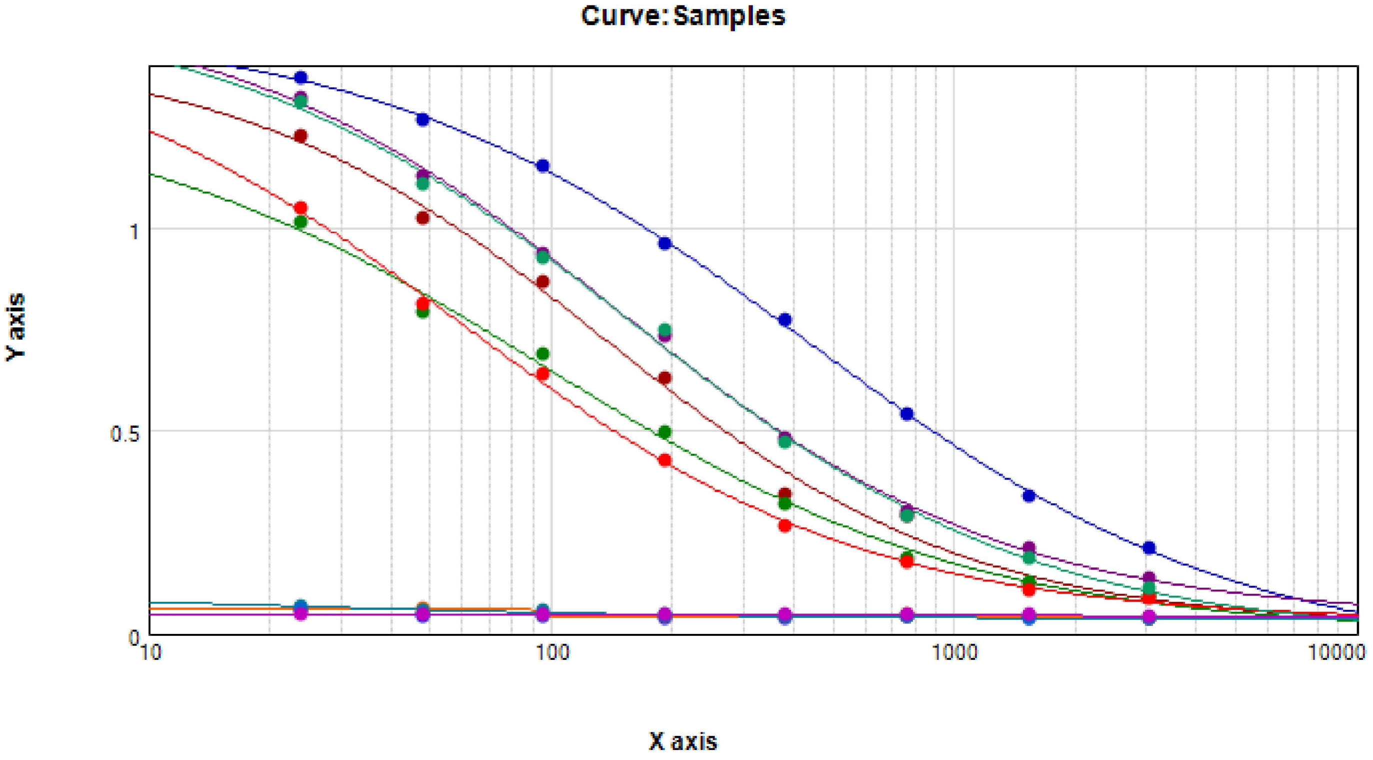

- **STD** (Standards: OD vs Dilution) Weighting: Fixed
- **S-1** (Samples: ODS1 vs DilSple1) Weighting: Fixed
- **S-2** (Samples: ODS2 vs DilSple2) Weighting: Fixed
- **S-3** (Samples: ODS3 vs DilSple3) Weighting: Fixed
- **S-4** (Samples: ODS4 vs DilSple4) Weighting: Fixed
- **S-5** (Samples: ODS5 vs DilSple5) Weighting: Fixed
- **S-6** (Samples: ODS6 vs DilSple6) Weighting: Fixed
- **S-7** (Samples: ODS7 vs DilSple7) Weighting: Fixed
- **S-8** (Samples: ODS8 vs DilSple8) Weighting: Fixed
- **S-9** (Samples: ODS9 vs DilSple9) Weighting: Fixed
- **S-10** (Samples: ODS10 vs DilSple10) Weighting: Fixed
- **S-11** (Samples: ODS11 vs DilSple11) Weighting: Fixed

Curve Fit Results ▼

Assay Parameter

Samples

Theoretical First Dilution Of Test Sample In Plate : 24.0   Sample dilution fold: 2.0

Nipha_Standard : NV-1

Concentration: 1000.0

Dilution (First dil in plate): 24.0

Dilution fold: 2.0

Others parameters

Rounding Decimal Standard Th.Conc: 1

Rounding Decimal RelErr% & CVdil: 1

Rounding Decimal GMC: 1

Average ODs of Blank: 0.046

SD of Blank: 0.002

Cutoff OD: 0.094
