# Supplementary material for: Development and Qualification of a Nipah Virus Glycoprotein-Specific IgG ELISA for the Assessment of Human Antibody Responses
Source: Vaccines (Basel). 2026 Jun 16;14(6):534. doi: 10.3390/vaccines14060534 (PMC13307770; doi:10.3390/vaccines14060534)
Supplement: Supplementary file 1 [file vaccines-14-00534-s001.zip › Supplementary_ELISA Qualification Data & Graph/1. Sensitivity and Specificity_Analysist-1/2. Sensitivity and Specificity_WHO IS_Analyst-1_Day-2.pdf]

Intro

Nipah_NIBSC_13 AUGUST_Analyst#1_Day#2

OD

	1	2	3	4	5	6	7	8	9	10	11	12
A	1.220	1.177	0.915	0.815	0.638	0.065	0.059	0.072	0.077	0.059	0.041	0.047
B	1.028	0.987	0.782	0.711	0.511	0.058	0.046	0.065	0.062	0.046	0.042	0.042
C	0.861	0.743	0.625	0.478	0.320	0.048	0.045	0.060	0.054	0.045	0.040	0.048
D	0.627	0.509	0.451	0.316	0.197	0.042	0.043	0.050	0.047	0.043	0.041	0.045
E	0.389	0.323	0.244	0.184	0.093	0.039	0.042	0.047	0.042	0.042	0.043	0.046
F	0.291	0.210	0.161	0.092	0.081	0.046	0.042	0.044	0.038	0.042	0.040	0.047
G	0.118	0.115	0.085	0.073	0.061	0.044	0.039	0.040	0.041	0.039	0.044	0.049
H	0.090	0.087	0.061	0.051	0.049	0.046	0.037	0.039	0.045	0.037	0.046	0.046

Settings Information

Endpoint
- Absorbance
  - Lm1 450
- More Settings
  - Shake Off
  - Calibrate On
  - Carriage Speed Normal
  - Column Priority

Read Information

Imported Data : 4:12 PM 9/1/2024
Imported By : anjan

Reduction Settings

Optical Density
Wavelength Combination : !Lm1

Sample Dil

Main Sample Dilution 24.0

Sample 1: NV-2 24.0

Sample 2: NV-4 24.0

Sample 3: NV-10 24.0

Sample 4: NV-6 24.0

Sample 5: NC-1 24.0

Sample 6: NC-2 24.0

Sample 7: NC-3 24.0

Sample 8: NC-4 24.0

Sample 9: CNC 24.0

Sample 10: BLANK-2 24.0

Sample 11: BLANK 24.0

Standards

Sample	Wells	OD	OK OD	Dilution	Calc.Conc	Adj.Conc	GMC	N	Th.Conc	RelErr%
01	A1	1.220	1.220	24	44.008	1056.2	981.2	7	41.700	5.500
	B1	1.028	1.028	48	19.060	914.9			20.800	-8.400
	C1	0.861	0.861	96	10.910	1047.3			10.400	4.900
	D1	0.627	0.627	192	5.327	1022.8			5.200	2.400
	E1	0.389	0.389	384	2.378	913.0			2.600	-8.600
	F1	0.291	0.291	768	1.567	1203.2			1.300	20.500
	G1	0.118	0.118	1536	0.501	770.0			0.700	-28.400
	H1	0.090		3072					0.300	

Samples

Sample	Wells	ID	OD	OK OD	Dilution	Calc.Conc	Adjusted.Conc	GMC	N	CVdil
01	A2	1	1.177	1.177	24	35.294	847.051	754.3	7	7.1
	B2		0.987	0.987	48	16.483	791.170			
	C2		0.743	0.743	96	7.592	728.841			
	D2		0.509	0.509	192	3.651	701.057			
	E2		0.323	0.323	384	1.812	695.958			
	F2		0.210	0.210	768	1.016	780.589			
	G2		0.115	0.115	1536	0.486	746.953			
	H2		0.087		3072					
02	A3	2	0.915	0.915	24	12.957	310.973	463.3	6	23.3
	B3		0.782	0.782	48	8.549	410.336			
	C3		0.625	0.625	96	5.294	508.246			
	D3		0.451	0.451	192	2.991	574.288			
	E3		0.244	0.244	384	1.236	474.440			
	F3		0.161	0.161	768	0.728	559.423			
	G3		0.085		1536					
	H3		0.061		3072					
03	A4	3	0.815	0.815	24	9.458	226.992	305.0	5	16.8
	B4		0.711	0.711	48	6.889	330.674			
	C4		0.478	0.478	96	3.287	315.563			
	D4		0.316	0.316	192	1.757	337.372			
	E4		0.184	0.184	384	0.860	330.096			
	F4		0.092		768					
	G4		0.073		1536					
	H4		0.051		3072					
04	A5	4	0.638	0.638	24	5.511	132.273	163.9	4	14.5
	B5		0.511	0.511	48	3.676	176.438			
	C5		0.320	0.320	96	1.789	171.707			
	D5		0.197	0.197	192	0.937	179.879			
	E5		0.093		384					
	F5		0.081		768					
	G5		0.061		1536					
	H5		0.049		3072					
05	A6	5	0.065		24			N/A	0	-----
	B6		0.058		48					
	C6		0.048		96					
	D6		0.042		192					
	E6		0.039		384					
	F6		0.046		768					
	G6		0.044		1536					
	H6		0.046		3072					
06	A7	6	0.059		24			N/A	0	-----
	B7		0.046		48					
	C7		0.045		96					
	D7		0.043		192					
	E7		0.042		384					
	F7		0.042		768					
	G7		0.039		1536					
	H7		0.037		3072					
07	A8	7	0.072		24			N/A	0	-----
	B8		0.065		48					
	C8		0.060		96					
	D8		0.050		192					
	E8		0.047		384					
	F8		0.044		768					
	G8		0.040		1536					
	H8		0.039		3072					
08	A9	8	0.077		24			N/A	0	-----
	B9		0.062		48					
	C9		0.054		96					
	D9		0.047		192					

Samples (Contd)

Sample	Wells	ID	OD	OK OD	Dilution	Calc.Conc	Adjusted.Conc	GMC	N	CVdil
	E9		0.042		384					
	F9		0.038		768					
	G9		0.041		1536					
	H9		0.045		3072					
09	A10	9	0.059		24			N/A	0	-----
	B10		0.046		48					
	C10		0.045		96					
	D10		0.043		192					
	E10		0.042		384					
	F10		0.042		768					
	G10		0.039		1536					
	H10		0.037		3072					
10	A11	10	0.041		24			N/A	0	-----
	B11		0.042		48					
	C11		0.040		96					
	D11		0.041		192					
	E11		0.043		384					
	F11		0.040		768					
	G11		0.044		1536					
	H11		0.046		3072					
11	A12	11	0.047		24			N/A	0	-----
	B12		0.042		48					
	C12		0.048		96					
	D12		0.045		192					
	E12		0.046		384					
	F12		0.047		768					
	G12		0.049		1536					
	H12		0.046		3072					

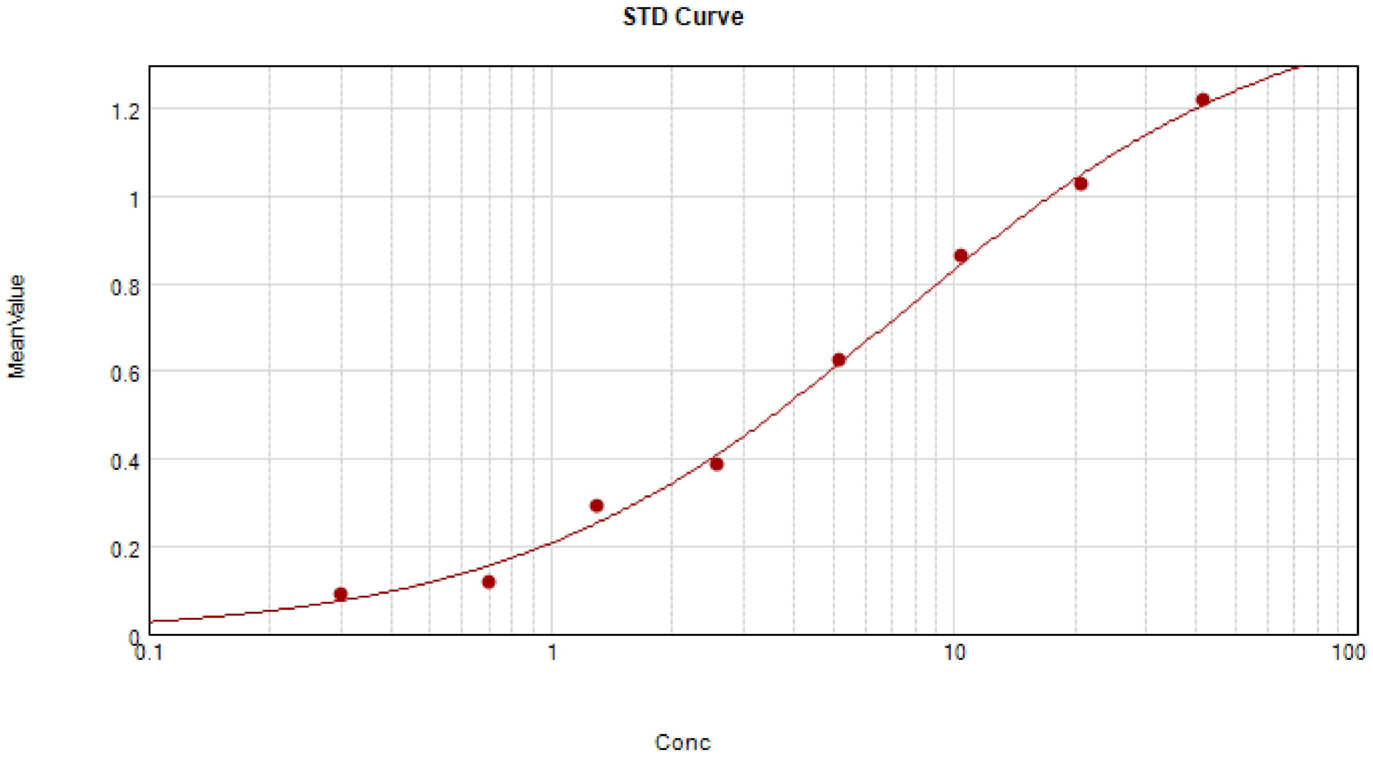

● **Std** (Standards: OD vs Th.Conc) Weighting: Fixed

Curve Fit Results ▲

Curve Fit : 4-Parameter Logistic $y = D + \frac{A-D}{1+\left(\frac{x}{C}\right)^B}$

	Parameter	Estimated Value	Std. Error	Confidence Interval
**Std**<br>R^2 = 0.996<br>EC50 = 7.304	A	-0.003	0.070	[-0.197, 0.190]
	B	0.898	0.186	[0.381, 1.415]
	C	7.304	1.671	[2.664, 11.94]
	D	1.464	0.160	[1.021, 1.907]

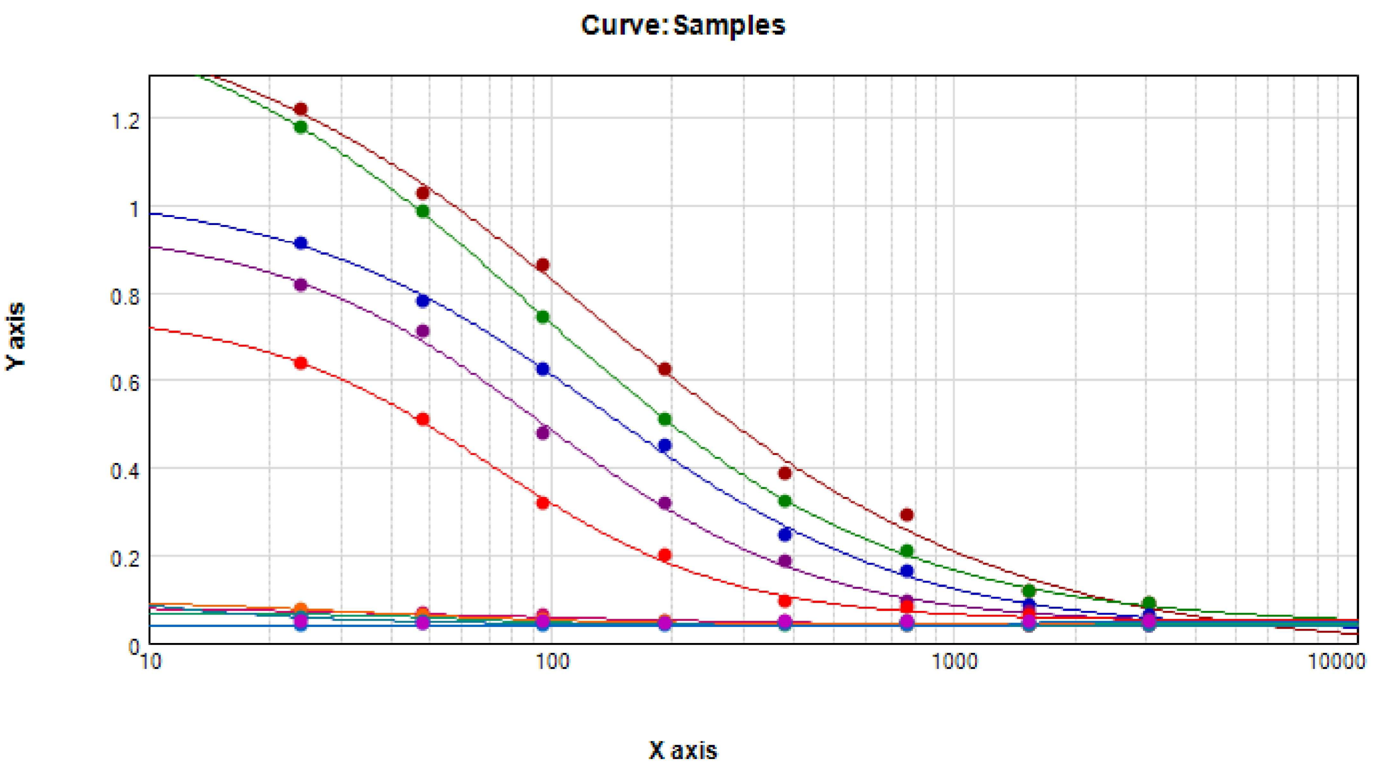

- **STD** (Standards: OD vs Dilution) Weighting: Fixed
- **S-1** (Samples: ODS1 vs DilSple1) Weighting: Fixed
- **S-2** (Samples: ODS2 vs DilSple2) Weighting: Fixed
- **S-3** (Samples: ODS3 vs DilSple3) Weighting: Fixed
- **S-4** (Samples: ODS4 vs DilSple4) Weighting: Fixed
- **S-5** (Samples: ODS5 vs DilSple5) Weighting: Fixed
- **S-6** (Samples: ODS6 vs DilSple6) Weighting: Fixed
- **S-7** (Samples: ODS7 vs DilSple7) Weighting: Fixed
- **S-8** (Samples: ODS8 vs DilSple8) Weighting: Fixed
- **S-9** (Samples: ODS9 vs DilSple9) Weighting: Fixed
- **S-10** (Samples: ODS10 vs DilSple10) Weighting: Fixed
- **S-11** (Samples: ODS11 vs DilSple11) Weighting: Fixed

Curve Fit Results ▼

Assay Parameter

Samples

Theoretical First Dilution Of Test Sample In Plate : 24.0   Sample dilution fold: 2.0

Nipha_Standard : NV-1

Concentration: 1000.0

Dilution (First dil in plate): 24.0

Dilution fold: 2.0

Others parameters

Rounding Decimal Standard Th.Conc: 1

Rounding Decimal RelErr% & CVdil: 1

Rounding Decimal GMC: 1

Average ODs of Blank: 0.046

SD of Blank: 0.002

Cutoff OD: 0.095
