# Supplementary material for: Development and Qualification of a Nipah Virus Glycoprotein-Specific IgG ELISA for the Assessment of Human Antibody Responses
Source: Vaccines (Basel). 2026 Jun 16;14(6):534. doi: 10.3390/vaccines14060534 (PMC13307770; doi:10.3390/vaccines14060534)
Supplement: Supplementary file 1 [file vaccines-14-00534-s001.zip › Supplementary_ELISA Qualification Data & Graph/1. Sensitivity and Specificity_Analysist-1/3. Sensitivity and Specificity_WHO IS_Analyst-1_Day-3.pdf]

Intro

Nipah_NIBSC_14 AUGUST_Analyst#1_Day#3

OD

	1	2	3	4	5	6	7	8	9	10	11	12
A	1.223	1.173	0.918	0.820	0.640	0.068	0.068	0.072	0.077	0.052	0.041	0.047
B	1.030	0.981	0.783	0.718	0.515	0.053	0.054	0.065	0.062	0.048	0.042	0.042
C	0.872	0.739	0.630	0.480	0.322	0.045	0.046	0.060	0.054	0.045	0.040	0.048
D	0.632	0.511	0.448	0.310	0.194	0.047	0.042	0.041	0.047	0.046	0.041	0.045
E	0.391	0.326	0.242	0.178	0.091	0.044	0.040	0.039	0.042	0.042	0.043	0.046
F	0.289	0.221	0.165	0.094	0.084	0.040	0.041	0.046	0.038	0.042	0.040	0.047
G	0.121	0.120	0.088	0.082	0.065	0.041	0.042	0.044	0.041	0.039	0.044	0.049
H	0.093	0.091	0.064	0.061	0.052	0.046	0.041	0.046	0.045	0.037	0.046	0.046

Settings Information
Endpoint
Absorbance
Lm1 450
More Settings
Shake Off
Calibrate On
Carriage Speed Normal
Column Priority

Read Information
Imported Data : 4:17 PM 9/1/2024
Imported By : anjan

Reduction Settings

Optical Density
Wavelength Combination : !Lm1

Sample Dil

Main Sample Dilution 24.0

Sample 1: NV-2 24.0

Sample 2: NV-4 24.0

Sample 3: NV-10 24.0

Sample 4: NV-6 24.0

Sample 5: NC-1 24.0

Sample 6: NC-2 24.0

Sample 7: NC-3 24.0

Sample 8: NC-4 24.0

Sample 9: CNC 24.0

Sample 10: BLANK-2 24.0

Sample 11: BLANK 24.0

Standards

Sample	Wells	OD	OK OD	Dilution	Calc.Conc	Adj.Conc	GMC	N	Th.Conc	RelErr%
01	A1	1.223	1.223	24	44.636	1071.3	982.2	7	41.700	7.000
	B1	1.030	1.030	48	18.726	898.9			20.800	-10.000
	C1	0.872	0.872	96	11.017	1057.6			10.400	5.900
	D1	0.632	0.632	192	5.320	1021.5			5.200	2.300
	E1	0.391	0.391	384	2.382	914.6			2.600	-8.400
	F1	0.289	0.289	768	1.548	1188.9			1.300	19.100
	G1	0.121	0.121	1536	0.508	779.7			0.700	-27.500
	H1	0.093		3072					0.300	

Samples

Sample	Wells	ID	OD	OK OD	Dilution	Calc.Conc	Adjusted.Conc	GMC	N	CVdil
01	A2	1	1.173	1.173	24	34.158	819.796	753.1	7	7.4
	B2		0.981	0.981	48	15.736	755.305			
	C2		0.739	0.739	96	7.345	705.074			
	D2		0.511	0.511	192	3.634	697.764			
	E2		0.326	0.326	384	1.830	702.715			
	F2		0.221	0.221	768	1.083	831.692			
	G2		0.120	0.120	1536	0.502	771.780			
	H2		0.091		3072					
02	A3	2	0.918	0.918	24	12.754	306.106	459.8	6	24.1
	B3		0.783	0.783	48	8.384	402.440			
	C3		0.630	0.630	96	5.288	507.623			
	D3		0.448	0.448	192	2.934	563.396			
	E3		0.242	0.242	384	1.220	468.363			
	F3		0.165	0.165	768	0.746	572.856			
	G3		0.088		1536					
	H3		0.064		3072					
03	A4	3	0.820	0.820	24	9.381	225.145	299.7	5	16.3
	B4		0.718	0.718	48	6.896	331.012			
	C4		0.480	0.480	96	3.277	314.596			
	D4		0.310	0.310	192	1.705	327.436			
	E4		0.178	0.178	384	0.821	315.144			
	F4		0.094		768					
	G4		0.082		1536					
	H4		0.061		3072					
04	A5	4	0.640	0.640	24	5.451	130.831	162.8	4	14.7
	B5		0.515	0.515	48	3.682	176.746			
	C5		0.322	0.322	96	1.798	172.651			
	D5		0.194	0.194	192	0.916	175.787			
	E5		0.091		384					
	F5		0.084		768					
	G5		0.065		1536					
	H5		0.052		3072					
05	A6	5	0.068		24			N/A	0	-----
	B6		0.053		48					
	C6		0.045		96					
	D6		0.047		192					
	E6		0.044		384					
	F6		0.040		768					
	G6		0.041		1536					
	H6		0.046		3072					
06	A7	6	0.068		24			N/A	0	-----
	B7		0.054		48					
	C7		0.046		96					
	D7		0.042		192					
	E7		0.040		384					
	F7		0.041		768					
	G7		0.042		1536					
	H7		0.041		3072					
07	A8	7	0.072		24			N/A	0	-----
	B8		0.065		48					
	C8		0.060		96					
	D8		0.041		192					
	E8		0.039		384					
	F8		0.046		768					
	G8		0.044		1536					
	H8		0.046		3072					
08	A9	8	0.077		24			N/A	0	-----
	B9		0.062		48					
	C9		0.054		96					
	D9		0.047		192					

Samples (Contd)

Sample	Wells	ID	OD	OK OD	Dilution	Calc.Conc	Adjusted.Conc	GMC	N	CVdil
	E9		0.042		384					
	F9		0.038		768					
	G9		0.041		1536					
	H9		0.045		3072					
09	A10	9	0.052		24			N/A	0	-----
	B10		0.048		48					
	C10		0.045		96					
	D10		0.046		192					
	E10		0.042		384					
	F10		0.042		768					
	G10		0.039		1536					
	H10		0.037		3072					
10	A11	10	0.041		24			N/A	0	-----
	B11		0.042		48					
	C11		0.040		96					
	D11		0.041		192					
	E11		0.043		384					
	F11		0.040		768					
	G11		0.044		1536					
	H11		0.046		3072					
11	A12	11	0.047		24			N/A	0	-----
	B12		0.042		48					
	C12		0.048		96					
	D12		0.045		192					
	E12		0.046		384					
	F12		0.047		768					
	G12		0.049		1536					
	H12		0.046		3072					

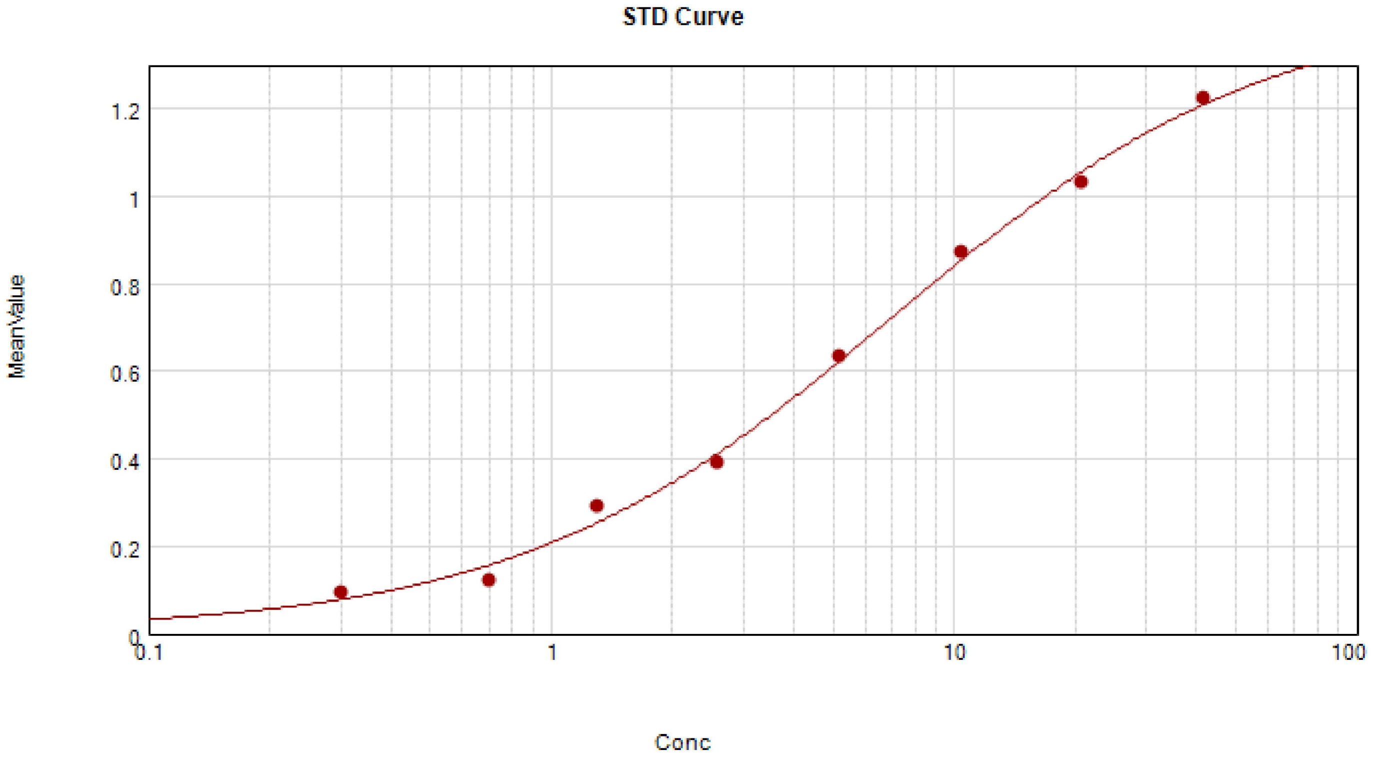

● **Std** (Standards: OD vs Th.Conc) Weighting: Fixed

Curve Fit Results ▲

Curve Fit : 4-Parameter Logistic $y = D + \frac{A - D}{1 + \left(\frac{x}{C}\right)^B}$

	Parameter	Estimated Value	Std. Error	Confidence Interval
**Std** $R^2 = 0.996$ EC50 = 7.008	A	0.006	0.066	[-0.178, 0.190]
	B	0.929	0.186	[0.413, 1.445]
	C	7.008	1.468	[2.931, 11.08]
	D	1.441	0.145	[1.039, 1.842]

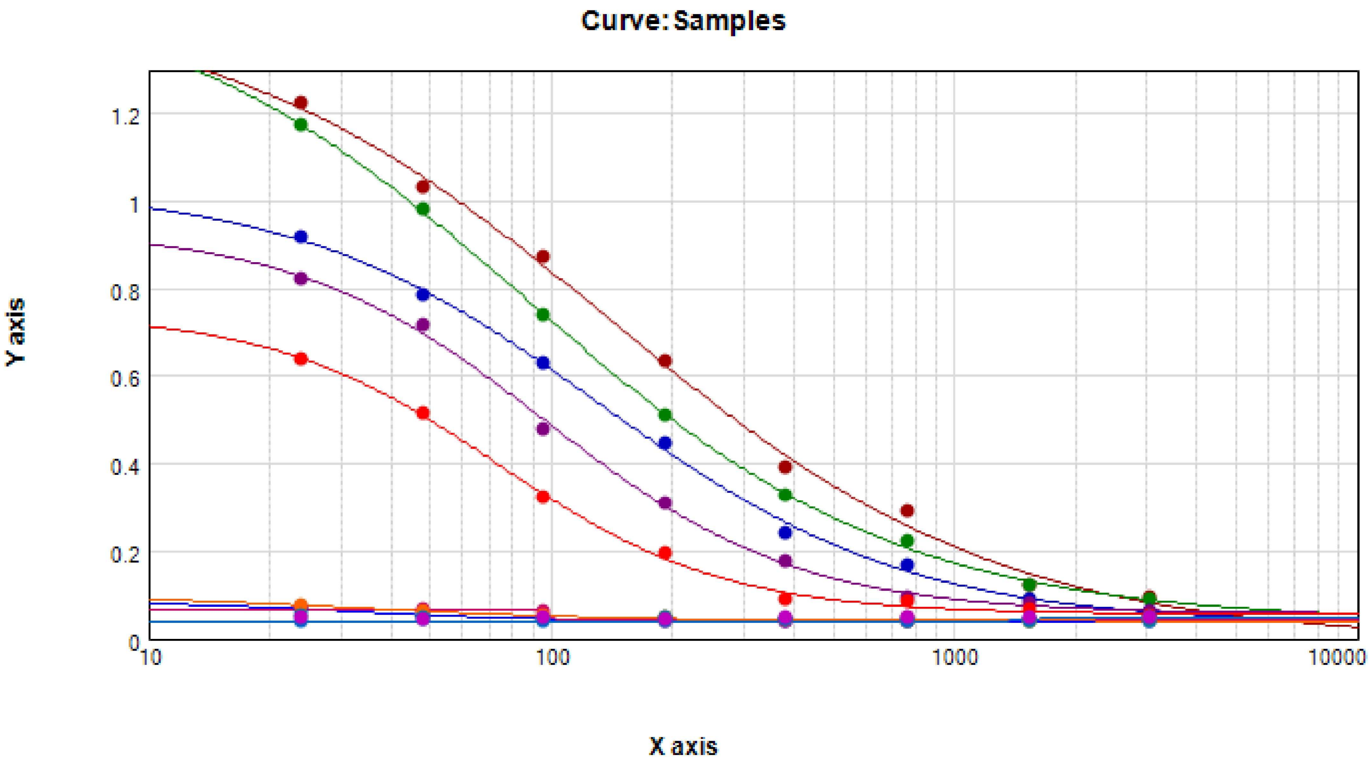

- **STD** (Standards: OD vs Dilution) Weighting: Fixed
- **S-1** (Samples: ODS1 vs DilSple1) Weighting: Fixed
- **S-2** (Samples: ODS2 vs DilSple2) Weighting: Fixed
- **S-3** (Samples: ODS3 vs DilSple3) Weighting: Fixed
- **S-4** (Samples: ODS4 vs DilSple4) Weighting: Fixed
- **S-5** (Samples: ODS5 vs DilSple5) Weighting: Fixed
- **S-6** (Samples: ODS6 vs DilSple6) Weighting: Fixed
- **S-7** (Samples: ODS7 vs DilSple7) Weighting: Fixed
- **S-8** (Samples: ODS8 vs DilSple8) Weighting: Fixed
- **S-9** (Samples: ODS9 vs DilSple9) Weighting: Fixed
- **S-10** (Samples: ODS10 vs DilSple10) Weighting: Fixed
- **S-11** (Samples: ODS11 vs DilSple11) Weighting: Fixed

Curve Fit Results ▼

Assay Parameter

Samples

Theoretical First Dilution Of Test Sample In Plate : 24.0   Sample dilution fold: 2.0

Nipha_Standard : NV-1

Concentration: 1000.0

Dilution (First dil in plate): 24.0

Dilution fold: 2.0

Others parameters

Rounding Decimal Standard Th.Conc: 1

Rounding Decimal RelErr% & CVdil: 1

Rounding Decimal GMC: 1

Average ODs of Blank: 0.046

SD of Blank: 0.002

Cutoff OD: 0.095
