# Supplementary material for: Development and Qualification of a Nipah Virus Glycoprotein-Specific IgG ELISA for the Assessment of Human Antibody Responses
Source: Vaccines (Basel). 2026 Jun 16;14(6):534. doi: 10.3390/vaccines14060534 (PMC13307770; doi:10.3390/vaccines14060534)
Supplement: Supplementary file 1 [file vaccines-14-00534-s001.zip › Supplementary_ELISA Qualification Data & Graph/1. Sensitivity and Specificity_Analysist-1/1. Sensitivity and Specificity_WHO IS_Analyst-1_Day-1.pdf]

Audit Trail

Wednesday, August 21, 2024 3:32:08 PM User ID: anjan
User created a new document.
Created from: Testproject_1/Nipah_21 August_Day#1
Created by: SoftMax Pro 7.1 GxP
Product Key: Remote

Wednesday, August 21, 2024 3:32:08 PM User ID: anjan
User saved a document using Save or Save As.
Document saved as: Testproject_1/Nipah_21 August_Day#1_NIBSC

Wednesday, August 21, 2024 3:32:29 PM User ID: anjan
User pasted data over existing data in a Plate section or Cuvette Set section.
Experiment: Expt1
Section: OD

Wednesday, August 21, 2024 3:32:29 PM User ID: anjan
User deleted data from a Plate section or a Cuvette Set section.
Deleted from Experiment: Expt1
Section: OD

Wednesday, August 21, 2024 3:32:30 PM User ID: anjan
User pasted data into a Plate section.
Experiment: Expt1
Section: OD

Wednesday, August 21, 2024 3:33:24 PM User ID: anjan
User saved a document using Save or Save As.
Document saved as: Testproject_1/Nipah_21 August_Day#1_NIBSC

Wednesday, August 21, 2024 3:33:40 PM User ID: anjan
User saved a document using Save or Save As.
Document saved as: Testproject_1/Nipah_21 August_Day#1_NIBSC

Intro

Nipah_NIBSC_21 August_Day#1

OD

	1	2	3	4	5	6	7	8	9	10	11	12
A	1.220	1.181	0.911	0.805	0.643	0.063	0.053	0.072	0.077	0.059	0.041	0.047
B	1.020	0.991	0.785	0.703	0.504	0.055	0.056	0.065	0.062	0.046	0.042	0.042
C	0.859	0.745	0.620	0.481	0.317	0.046	0.050	0.060	0.054	0.045	0.040	0.048
D	0.618	0.512	0.457	0.311	0.193	0.045	0.042	0.050	0.047	0.043	0.041	0.045
E	0.395	0.326	0.249	0.181	0.093	0.039	0.042	0.047	0.042	0.042	0.043	0.046
F	0.294	0.213	0.169	0.094	0.081	0.046	0.046	0.044	0.038	0.042	0.040	0.047
G	0.124	0.117	0.083	0.074	0.061	0.044	0.039	0.040	0.041	0.039	0.044	0.049
H	0.090	0.089	0.065	0.055	0.049	0.046	0.048	0.039	0.045	0.037	0.046	0.046

Settings Information
Endpoint
Absorbance
Lm1 450
More Settings
Shake Off
Calibrate On
Carriage Speed Normal
Column Priority

Read Information
Imported Data : 3:32 PM 8/21/2024
Imported By : anjan

Reduction Settings

Optical Density
Wavelength Combination : !Lm1

Sample Dil

Main Sample Dilution 24.0

Sample 1: NV-2 24.0

Sample 2: NV-4 24.0

Sample 3: NV-10 24.0

Sample 4: NV-6 24.0

Sample 5: NC-1 24.0

Sample 6: NC-2 24.0

Sample 7: NC-3 24.0

Sample 8: NC-4 24.0

Sample 9: CNC 24.0

Sample 10: BLANK-2 24.0

Sample 11: BLANK 24.0

Standards

Sample	Wells	OD	OK OD	Dilution	Calc.Conc	Adj.Conc	GMC	N	Th.Conc	RelErr%
01	A1	1.220	1.220	24	43.830	1051.9	984.4	7	41.700	5.100
	B1	1.020	1.020	48	18.979	911.0			20.800	-8.800
	C1	0.859	0.859	96	11.096	1065.2			10.400	6.700
	D1	0.618	0.618	192	5.228	1003.7			5.200	0.500
	E1	0.395	0.395	384	2.407	924.4			2.600	-7.400
	F1	0.294	0.294	768	1.556	1194.9			1.300	19.700
	G1	0.124	0.124	1536	0.515	791.7			0.700	-26.400
	H1	0.090		3072					0.300	

Samples

Sample	Wells	ID	OD	OK OD	Dilution	Calc.Conc	Adjusted.Conc	GMC	N	CVdil
01	A2	1	1.181	1.181	24	36.249	869.987	762.4	7	8.1
	B2		0.991	0.991	48	17.140	822.712			
	C2		0.745	0.745	96	7.775	746.388			
	D2		0.512	0.512	192	3.689	708.343			
	E2		0.326	0.326	384	1.804	692.745			
	F2		0.213	0.213	768	1.006	772.352			
	G2		0.117	0.117	1536	0.482	739.701			
	H2		0.089		3072					
02	A3	2	0.911	0.911	24	13.111	314.654	469.5	6	23.4
	B3		0.785	0.785	48	8.801	422.446			
	C3		0.620	0.620	96	5.261	505.065			
	D3		0.457	0.457	192	3.041	583.861			
	E3		0.249	0.249	384	1.237	475.050			
	F3		0.169	0.169	768	0.749	575.393			
	G3		0.083		1536					
	H3		0.065		3072					
03	A4	3	0.805	0.805	24	9.366	224.779	298.7	5	16.1
	B4		0.703	0.703	48	6.826	327.645			
	C4		0.481	0.481	96	3.313	318.050			
	D4		0.311	0.311	192	1.685	323.590			
	E4		0.181	0.181	384	0.816	313.485			
	F4		0.094		768					
	G4		0.074		1536					
	H4		0.055		3072					
04	A5	4	0.643	0.643	24	5.659	135.807	160.4	4	11.2
	B5		0.504	0.504	48	3.589	172.290			
	C5		0.317	0.317	96	1.732	166.303			
	D5		0.193	0.193	192	0.886	170.034			
	E5		0.093		384					
	F5		0.081		768					
	G5		0.061		1536					
	H5		0.049		3072					
05	A6	5	0.063		24			N/A	0	-----
	B6		0.055		48					
	C6		0.046		96					
	D6		0.045		192					
	E6		0.039		384					
	F6		0.046		768					
	G6		0.044		1536					
	H6		0.046		3072					
06	A7	6	0.053		24			N/A	0	-----
	B7		0.056		48					
	C7		0.050		96					
	D7		0.042		192					
	E7		0.042		384					
	F7		0.046		768					
	G7		0.039		1536					
	H7		0.048		3072					
07	A8	7	0.072		24			N/A	0	-----
	B8		0.065		48					
	C8		0.060		96					
	D8		0.050		192					
	E8		0.047		384					
	F8		0.044		768					
	G8		0.040		1536					
	H8		0.039		3072					
08	A9	8	0.077		24			N/A	0	-----
	B9		0.062		48					
	C9		0.054		96					
	D9		0.047		192					

Samples (Contd)

Sample	Wells	ID	OD	OK OD	Dilution	Calc.Conc	Adjusted.Conc	GMC	N	CVdil
	E9		0.042		384					
	F9		0.038		768					
	G9		0.041		1536					
	H9		0.045		3072					
09	A10	9	0.059		24			N/A	0	-----
	B10		0.046		48					
	C10		0.045		96					
	D10		0.043		192					
	E10		0.042		384					
	F10		0.042		768					
	G10		0.039		1536					
	H10		0.037		3072					
10	A11	10	0.041		24			N/A	0	-----
	B11		0.042		48					
	C11		0.040		96					
	D11		0.041		192					
	E11		0.043		384					
	F11		0.040		768					
	G11		0.044		1536					
	H11		0.046		3072					
11	A12	11	0.047		24			N/A	0	-----
	B12		0.042		48					
	C12		0.048		96					
	D12		0.045		192					
	E12		0.046		384					
	F12		0.047		768					
	G12		0.049		1536					
	H12		0.046		3072					

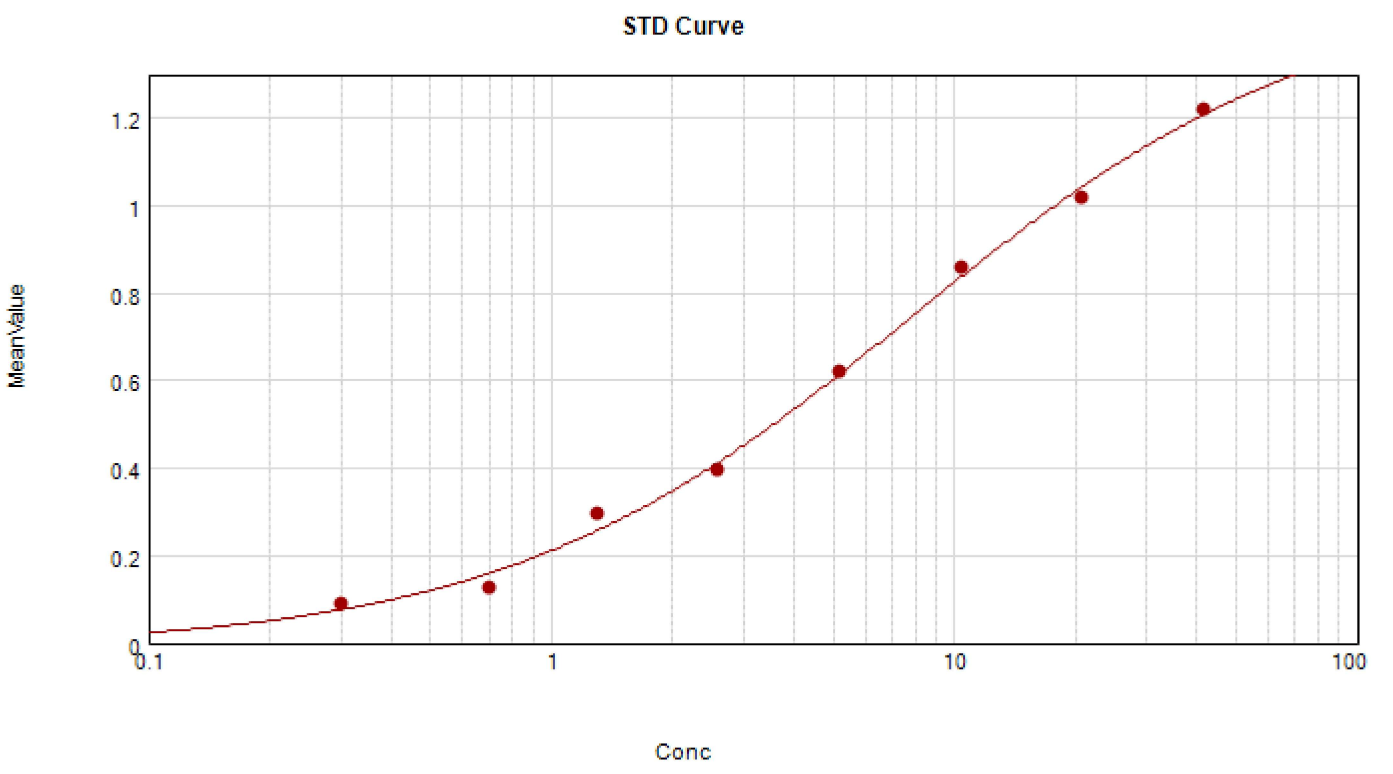

● **Std** (Standards: OD vs Th.Conc) Weighting: Fixed

Curve Fit Results ▲

Curve Fit : 4-Parameter Logistic $y = D + \frac{A - D}{1 + \left(\frac{x}{C}\right)^B}$

	Parameter	Estimated Value	Std. Error	Confidence Interval
**Std**<br>R^2 = 0.997<br>EC50 = 7.764	A	-0.012	0.074	[-0.217, 0.194]
	B	0.854	0.183	[0.345, 1.363]
	C	7.764	1.989	[2.241, 13.29]
	D	1.501	0.180	[1.000, 2.001]

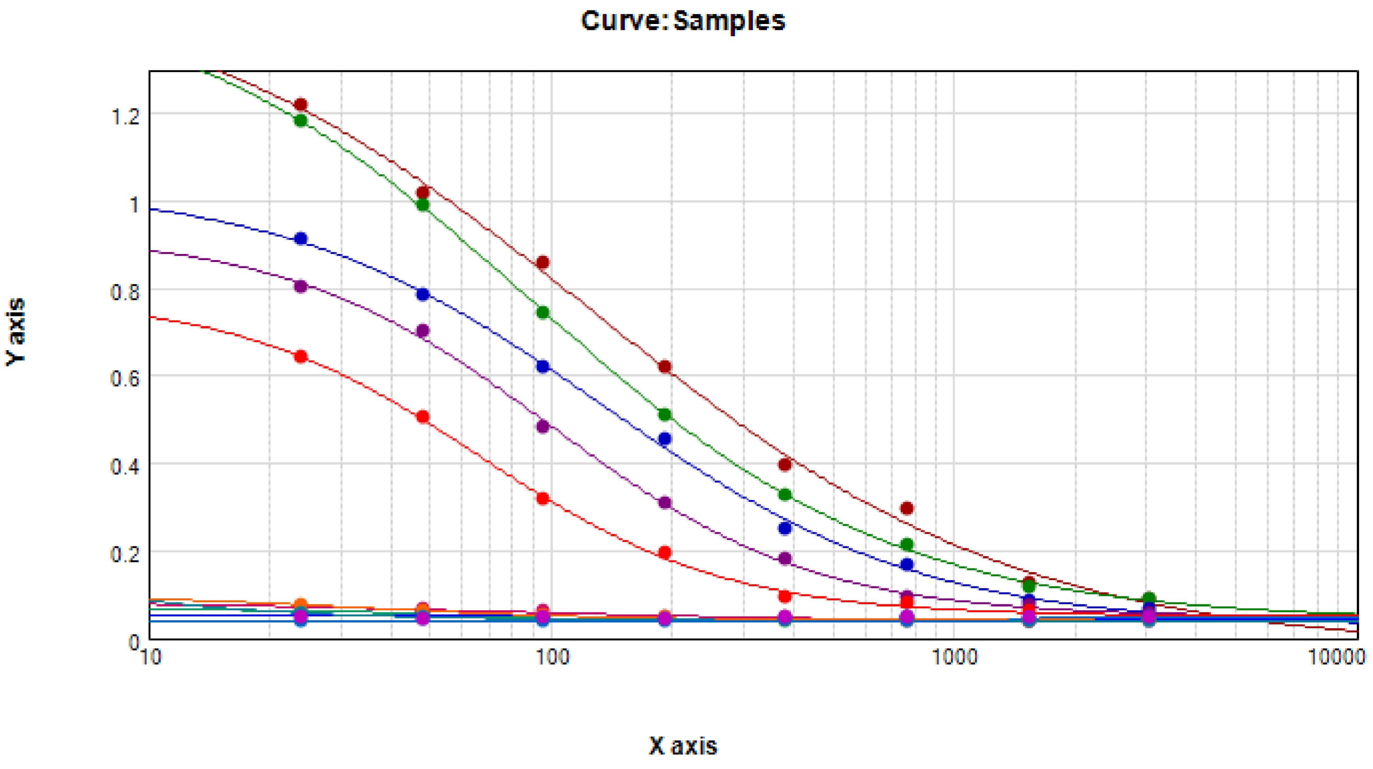

- **STD** (Standards: OD vs Dilution) Weighting: Fixed
- **S-1** (Samples: ODS1 vs DilSple1) Weighting: Fixed
- **S-2** (Samples: ODS2 vs DilSple2) Weighting: Fixed
- **S-3** (Samples: ODS3 vs DilSple3) Weighting: Fixed
- **S-4** (Samples: ODS4 vs DilSple4) Weighting: Fixed
- **S-5** (Samples: ODS5 vs DilSple5) Weighting: Fixed
- **S-6** (Samples: ODS6 vs DilSple6) Weighting: Fixed
- **S-7** (Samples: ODS7 vs DilSple7) Weighting: Fixed
- **S-8** (Samples: ODS8 vs DilSple8) Weighting: Fixed
- **S-9** (Samples: ODS9 vs DilSple9) Weighting: Fixed
- **S-10** (Samples: ODS10 vs DilSple10) Weighting: Fixed
- **S-11** (Samples: ODS11 vs DilSple11) Weighting: Fixed

Curve Fit Results ▼

Assay Parameter

Samples

Theoretical First Dilution Of Test Sample In Plate : 24.0 Sample dilution fold: 2.0

Nipha_Standard : NV-1

Concentration: 1000.0

Dilution (First dil in plate): 24.0

Dilution fold: 2.0

Others parameters

Rounding Decimal Standard Th.Conc: 1

Rounding Decimal RelErr% & CVdil: 1

Rounding Decimal GMC: 1

Average ODs of Blank: 0.046

SD of Blank: 0.002

Cutoff OD: 0.095
