# Supplementary material for: Development and Qualification of a Nipah Virus Glycoprotein-Specific IgG ELISA for the Assessment of Human Antibody Responses
Source: Vaccines (Basel). 2026 Jun 16;14(6):534. doi: 10.3390/vaccines14060534 (PMC13307770; doi:10.3390/vaccines14060534)
Supplement: Supplementary file 1 [file vaccines-14-00534-s001.zip › Supplementary_ELISA Qualification Data & Graph/2. Sensitivity and Specificity_Analysist-2/4. Sensitivity and Specificity_NHP_Analyst-2_Day-1.pdf]

Intro

NIPAH_NHP_27 AUGUST_Analyst#2_Day#1

OD

	1	2	3	4	5	6	7	8	9	10	11	12
A	1.240	1.011	1.369	1.316	1.032	1.302	0.060	0.058	0.062	0.054	0.053	0.046
B	1.021	0.787	1.238	1.127	0.794	1.101	0.058	0.045	0.055	0.047	0.048	0.048
C	0.870	0.667	1.110	0.926	0.625	0.909	0.042	0.047	0.044	0.048	0.045	0.048
D	0.627	0.482	0.960	0.733	0.421	0.726	0.047	0.042	0.043	0.046	0.042	0.046
E	0.393	0.311	0.749	0.480	0.259	0.471	0.044	0.043	0.044	0.048	0.042	0.048
F	0.291	0.172	0.531	0.282	0.140	0.283	0.043	0.044	0.043	0.046	0.042	0.046
G	0.118	0.116	0.335	0.205	0.105	0.191	0.041	0.044	0.041	0.045	0.044	0.045
H	0.091	0.083	0.190	0.120	0.073	0.110	0.041	0.042	0.044	0.044	0.040	0.044

Settings Information
Endpoint
Absorbance
Lm1 450
More Settings
Shake Off
Calibrate On
Carriage Speed Normal
Column Priority

Read Information
Imported Data : 4:42 PM 9/2/2024
Imported By : anjan

Reduction Settings

Optical Density
Wavelength Combination : !Lm1

Sample Dil

Main Sample Dilution 24.0

Sample 1: NHP-1 24.0

Sample 2: NHP-3 24.0

Sample 3: NHP-5 24.0

Sample 4: NHP-6 24.0

Sample 5: NHP-7 24.0

Sample 6: NC-5 24.0

Sample 7: NC-6 24.0

Sample 8: NC-7 24.0

Sample 9: NC-8 24.0

Sample 10: CNC 24.0

Sample 11: BLANK 24.0

Standards

Sample	Wells	OD	OK OD	Dilution	Calc.Conc	Adj.Conc	GMC	N	Th.Conc	RelErr%
01	A1	1.240	1.240	24	44.858	1076.6	979.7	7	41.700	7.600
	B1	1.021	1.021	48	18.340	880.3			20.800	-11.800
	C1	0.870	0.870	96	11.215	1076.7			10.400	7.800
	D1	0.627	0.627	192	5.322	1021.8			5.200	2.300
	E1	0.393	0.393	384	2.387	916.7			2.600	-8.200
	F1	0.291	0.291	768	1.541	1183.6			1.300	18.500
	G1	0.118	0.118	1536	0.499	765.7			0.700	-28.800
	H1	0.091		3072					0.300	

Samples

Sample	Wells	ID	OD	OK OD	Dilution	Calc.Conc	Adjusted.Conc	GMC	N	CVdil
01	A2	1	1.011	1.011	24	17.720	425.274	567.7	7	22.3
	B2		0.787	0.787	48	8.690	417.119			
	C2		0.667	0.667	96	6.025	578.408			
	D2		0.482	0.482	192	3.310	635.498			
	E2		0.311	0.311	384	1.692	649.595			
	F2		0.172	0.172	768	0.778	597.204			
	G2		0.116	0.116	1536	0.489	751.041			
	H2		0.083		3072					
02	A3	2	1.369	1.369	24	102.415	2457.960	2665.9	8	12.2
	B3		1.238	1.238	48	44.407	2131.547			
	C3		1.110	1.110	96	25.373	2435.790			
	D3		0.960	0.960	192	14.939	2868.373			
	E3		0.749	0.749	384	7.741	2972.493			
	F3		0.531	0.531	768	3.909	3002.168			
	G3		0.335	0.335	1536	1.882	2890.370			
	H3		0.190	0.190	3072	0.880	2702.053			
03	A4	3	1.316	1.316	24	69.254	1662.097	1378.8	8	12.7
	B4		1.127	1.127	48	27.123	1301.884			
	C4		0.926	0.926	96	13.382	1284.679			
	D4		0.733	0.733	192	7.373	1415.619			
	E4		0.480	0.480	384	3.287	1262.177			
	F4		0.282	0.282	768	1.476	1133.250			
	G4		0.205	0.205	1536	0.968	1486.943			
	H4		0.120	0.120	3072	0.508	1561.078			
04	A5	4	1.032	1.032	24	19.055	457.330	501.5	7	14.6
	B5		0.794	0.794	48	8.877	426.116			
	C5		0.625	0.625	96	5.289	507.707			
	D5		0.421	0.421	192	2.657	510.106			
	E5		0.259	0.259	384	1.314	504.677			
	F5		0.140	0.140	768	0.607	466.510			
	G5		0.105	0.105	1536	0.437	671.640			
	H5		0.073		3072					
05	A6	5	1.302	1.302	24	63.366	1520.791	1298.6	8	10.3
	B6		1.101	1.101	48	24.510	1176.475			
	C6		0.909	0.909	96	12.677	1216.951			
	D6		0.726	0.726	192	7.218	1385.763			
	E6		0.471	0.471	384	3.185	1223.021			
	F6		0.283	0.283	768	1.483	1138.788			
	G6		0.191	0.191	1536	0.885	1359.934			
	H6		0.110	0.110	3072	0.461	1414.837			
06	A7	6	0.060		24			N/A	0	-----
	B7		0.058		48					
	C7		0.042		96					
	D7		0.047		192					
	E7		0.044		384					
	F7		0.043		768					
	G7		0.041		1536					
	H7		0.041		3072					
07	A8	7	0.058		24			N/A	0	-----
	B8		0.045		48					
	C8		0.047		96					
	D8		0.042		192					
	E8		0.043		384					
	F8		0.044		768					
	G8		0.044		1536					
	H8		0.042		3072					
08	A9	8	0.062		24			N/A	0	-----
	B9		0.055		48					
	C9		0.044		96					
	D9		0.043		192					

Samples (Contd)

Sample	Wells	ID	OD	OK OD	Dilution	Calc.Conc	Adjusted.Conc	GMC	N	CVdil
	E9		0.044		384					
	F9		0.043		768					
	G9		0.041		1536					
	H9		0.044		3072					
09	A10	9	0.054		24			N/A	0	-----
	B10		0.047		48					
	C10		0.048		96					
	D10		0.046		192					
	E10		0.048		384					
	F10		0.046		768					
	G10		0.045		1536					
	H10		0.044		3072					
10	A11	10	0.053		24			N/A	0	-----
	B11		0.048		48					
	C11		0.045		96					
	D11		0.042		192					
	E11		0.042		384					
	F11		0.042		768					
	G11		0.044		1536					
	H11		0.040		3072					
11	A12	11	0.046		24			N/A	0	-----
	B12		0.048		48					
	C12		0.048		96					
	D12		0.046		192					
	E12		0.048		384					
	F12		0.046		768					
	G12		0.045		1536					
	H12		0.044		3072					

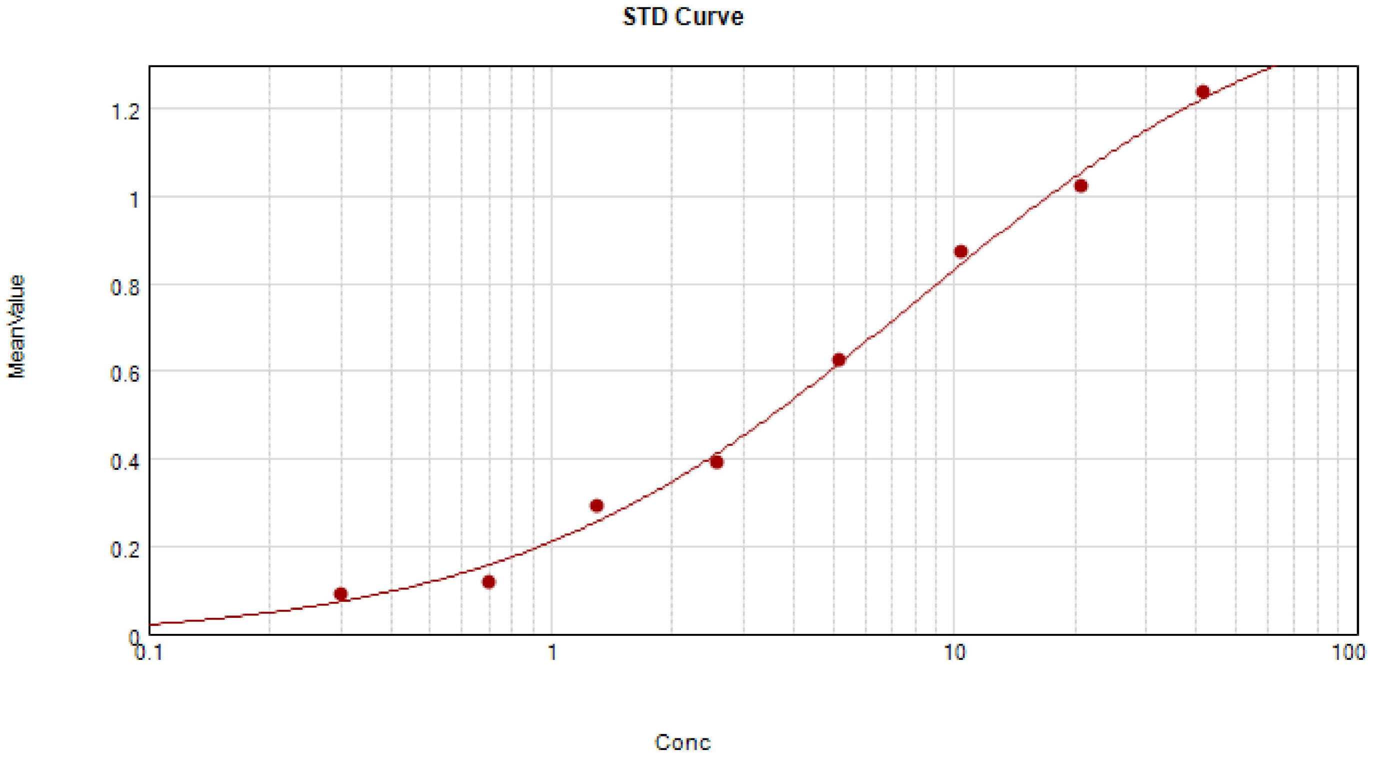

● **Std** (Standards: OD vs Th.Conc) Weighting: Fixed

Curve Fit Results ▲

Curve Fit : 4-Parameter Logistic $y = D + \frac{A - D}{1 + \left(\frac{x}{C}\right)^B}$

	Parameter	Estimated Value	Std. Error	Confidence Interval
**Std**<br>$R^2 = 0.996$<br>EC50 = 7.862	A	-0.015	0.084	[-0.249, 0.219]
	B	0.855	0.207	[0.279, 1.431]
	C	7.862	2.295	[1.490, 14.23]
	D	1.523	0.209	[0.944, 2.102]

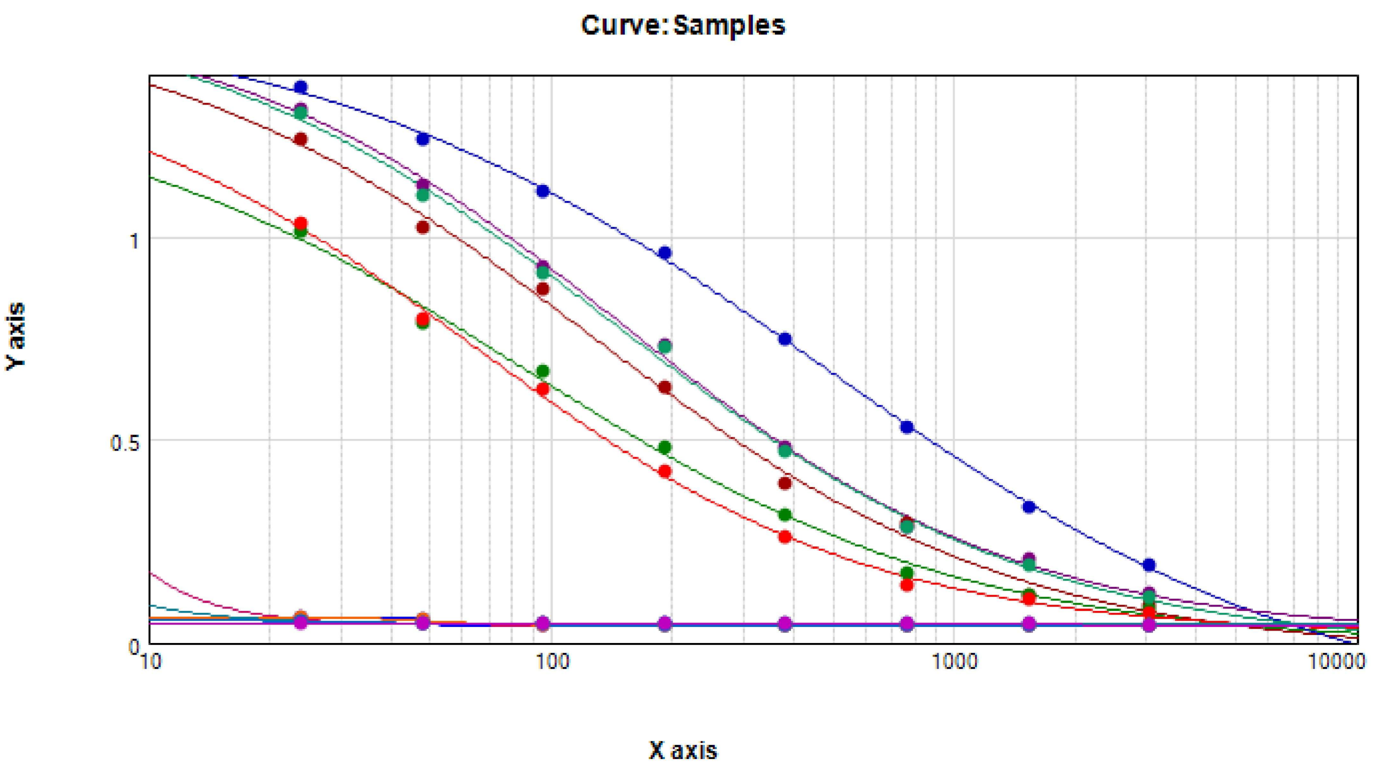

- **STD** (Standards: OD vs Dilution) Weighting: Fixed
- **S-1** (Samples: ODS1 vs DilSple1) Weighting: Fixed
- **S-2** (Samples: ODS2 vs DilSple2) Weighting: Fixed
- **S-3** (Samples: ODS3 vs DilSple3) Weighting: Fixed
- **S-4** (Samples: ODS4 vs DilSple4) Weighting: Fixed
- **S-5** (Samples: ODS5 vs DilSple5) Weighting: Fixed
- **S-6** (Samples: ODS6 vs DilSple6) Weighting: Fixed
- **S-7** (Samples: ODS7 vs DilSple7) Weighting: Fixed
- **S-8** (Samples: ODS8 vs DilSple8) Weighting: Fixed
- **S-9** (Samples: ODS9 vs DilSple9) Weighting: Fixed
- **S-10** (Samples: ODS10 vs DilSple10) Weighting: Fixed
- **S-11** (Samples: ODS11 vs DilSple11) Weighting: Fixed

Curve Fit Results ▼

Assay Parameter

Samples

Theoretical First Dilution Of Test Sample In Plate : 24.0   Sample dilution fold: 2.0

Nipha_Standard : NV-1

Concentration: 1000.0

Dilution (First dil in plate): 24.0

Dilution fold: 2.0

Others parameters

Rounding Decimal Standard Th.Conc: 1

Rounding Decimal RelErr% & CVdil: 1

Rounding Decimal GMC: 1

Average ODs of Blank: 0.046

SD of Blank: 0.002

Cutoff OD: 0.094
