# Supplementary material for: Development and Qualification of a Nipah Virus Glycoprotein-Specific IgG ELISA for the Assessment of Human Antibody Responses
Source: Vaccines (Basel). 2026 Jun 16;14(6):534. doi: 10.3390/vaccines14060534 (PMC13307770; doi:10.3390/vaccines14060534)
Supplement: Supplementary file 1 [file vaccines-14-00534-s001.zip › Supplementary_ELISA Qualification Data & Graph/2. Sensitivity and Specificity_Analysist-2/5. Sensitivity and Specificity_NHP_Analyst-2_Day-2.pdf]

Intro

Nipah_28 AUGUST_Analyst#2_Day#2

OD

	1	2	3	4	5	6	7	8	9	10	11	12
A	1.260	1.010	1.365	1.315	1.033	1.305	0.062	0.044	0.040	0.043	0.048	0.044
B	1.023	0.786	1.235	1.125	0.793	1.103	0.053	0.041	0.041	0.047	0.046	0.043
C	0.850	0.665	1.111	0.924	0.626	0.908	0.043	0.044	0.042	0.043	0.045	0.041
D	0.624	0.481	0.959	0.731	0.420	0.724	0.042	0.043	0.043	0.042	0.044	0.044
E	0.391	0.309	0.747	0.481	0.257	0.470	0.043	0.042	0.044	0.043	0.046	0.040
F	0.293	0.171	0.532	0.280	0.141	0.285	0.043	0.043	0.043	0.044	0.048	0.041
G	0.119	0.117	0.332	0.207	0.103	0.192	0.042	0.041	0.041	0.044	0.048	0.042
H	0.092	0.085	0.191	0.120	0.075	0.111	0.042	0.041	0.044	0.042	0.046	0.043

Settings Information
Endpoint
Absorbance
Lm1 450
More Settings
Shake Off
Calibrate On
Carriage Speed Normal
Column Priority

Read Information
Imported Data : 4:04 PM 9/1/2024
Imported By : anjan

Reduction Settings

Optical Density
Wavelength Combination : !Lm1

Sample Dil

Main Sample Dilution 24.0

Sample 1: NHP-1 24.0

Sample 2: NHP-3 24.0

Sample 3: NHP-5 24.0

Sample 4: NHP-6 24.0

Sample 5: NHP-7 24.0

Sample 6: CNC 24.0

Sample 7: Blank-5 24.0

Sample 8: Blank-4 24.0

Sample 9: BLANK-3 24.0

Sample 10: BLANK-2 24.0

Sample 11: BLANK 24.0

Standards

Sample	Wells	OD	OK OD	Dilution	Calc.Conc	Adj.Conc	GMC	N	Th.Conc	RelErr%
01	A1	1.260	1.260	24	43.948	1054.7	998.0	8	41.700	5.400
	B1	1.023	1.023	48	18.792	902.0			20.800	-9.700
	C1	0.850	0.850	96	10.963	1052.5			10.400	5.400
	D1	0.624	0.624	192	5.446	1045.5			5.200	4.700
	E1	0.391	0.391	384	2.376	912.3			2.600	-8.600
	F1	0.293	0.293	768	1.536	1179.5			1.300	18.100
	G1	0.119	0.119	1536	0.493	757.2			0.700	-29.600
	H1	0.092	0.092	3072	0.376	1153.9			0.300	25.200

Samples

Sample	Wells	ID	OD	OK OD	Dilution	Calc.Conc	Adjusted.Conc	GMC	N	CVdil
01	A2	1	1.010	1.010	24	18.023	432.547	570.3	7	20.7
	B2		0.786	0.786	48	9.020	432.939			
	C2		0.665	0.665	96	6.205	595.659			
	D2		0.481	0.481	192	3.353	643.684			
	E2		0.309	0.309	384	1.659	637.046			
	F2		0.171	0.171	768	0.751	577.059			
	G2		0.117	0.117	1536	0.484	743.199			
	H2		0.085		3072					
02	A3	2	1.365	1.365	24	70.924	1702.185	2523.2	8	22.8
	B3		1.235	1.235	48	39.719	1906.528			
	C3		1.111	1.111	96	25.177	2416.967			
	D3		0.959	0.959	192	15.338	2944.885			
	E3		0.747	0.747	384	8.005	3073.821			
	F3		0.532	0.532	768	4.013	3081.682			
	G3		0.332	0.332	1536	1.845	2834.214			
	H3		0.191	0.191	3072	0.862	2649.175			
03	A4	3	1.315	1.315	24	55.764	1338.335	1341.3	8	10.4
	B4		1.125	1.125	48	26.427	1268.488			
	C4		0.924	0.924	96	13.757	1320.633			
	D4		0.731	0.731	192	7.620	1463.101			
	E4		0.481	0.481	384	3.353	1287.368			
	F4		0.280	0.280	768	1.439	1105.436			
	G4		0.207	0.207	1536	0.956	1468.285			
	H4		0.120	0.120	3072	0.498	1528.335			
04	A5	4	1.033	1.033	24	19.410	465.831	502.5	7	12.9
	B5		0.793	0.793	48	9.214	442.288			
	C5		0.626	0.626	96	5.481	526.142			
	D5		0.420	0.420	192	2.667	512.007			
	E5		0.257	0.257	384	1.277	490.242			
	F5		0.141	0.141	768	0.597	458.524			
	G5		0.103	0.103	1536	0.422	648.228			
	H5		0.075		3072					
05	A6	5	1.305	1.305	24	53.304	1279.304	1277.8	8	8.1
	B6		1.103	1.103	48	24.496	1175.798			
	C6		0.908	0.908	96	13.094	1257.027			
	D6		0.724	0.724	192	7.458	1431.849			
	E6		0.470	0.470	384	3.221	1236.850			
	F6		0.285	0.285	768	1.476	1133.615			
	G6		0.192	0.192	1536	0.868	1333.376			
	H6		0.111	0.111	3072	0.457	1403.833			
06	A7	6	0.062		24			N/A	0	-----
	B7		0.053		48					
	C7		0.043		96					
	D7		0.042		192					
	E7		0.043		384					
	F7		0.043		768					
	G7		0.042		1536					
	H7		0.042		3072					
07	A8	7	0.044		24			N/A	0	-----
	B8		0.041		48					
	C8		0.044		96					
	D8		0.043		192					
	E8		0.042		384					
	F8		0.043		768					
	G8		0.041		1536					
	H8		0.041		3072					
08	A9	8	0.040		24			N/A	0	-----
	B9		0.041		48					
	C9		0.042		96					
	D9		0.043		192					

Samples (Contd)

Sample	Wells	ID	OD	OK OD	Dilution	Calc.Conc	Adjusted.Conc	GMC	N	CVdil
	E9		0.044		384					
	F9		0.043		768					
	G9		0.041		1536					
	H9		0.044		3072					
09	A10	9	0.043		24			N/A	0	-----
	B10		0.047		48					
	C10		0.043		96					
	D10		0.042		192					
	E10		0.043		384					
	F10		0.044		768					
	G10		0.044		1536					
	H10		0.042		3072					
10	A11	10	0.048		24			N/A	0	-----
	B11		0.046		48					
	C11		0.045		96					
	D11		0.044		192					
	E11		0.046		384					
	F11		0.048		768					
	G11		0.048		1536					
	H11		0.046		3072					
11	A12	11	0.044		24			N/A	0	-----
	B12		0.043		48					
	C12		0.041		96					
	D12		0.044		192					
	E12		0.040		384					
	F12		0.041		768					
	G12		0.042		1536					
	H12		0.043		3072					

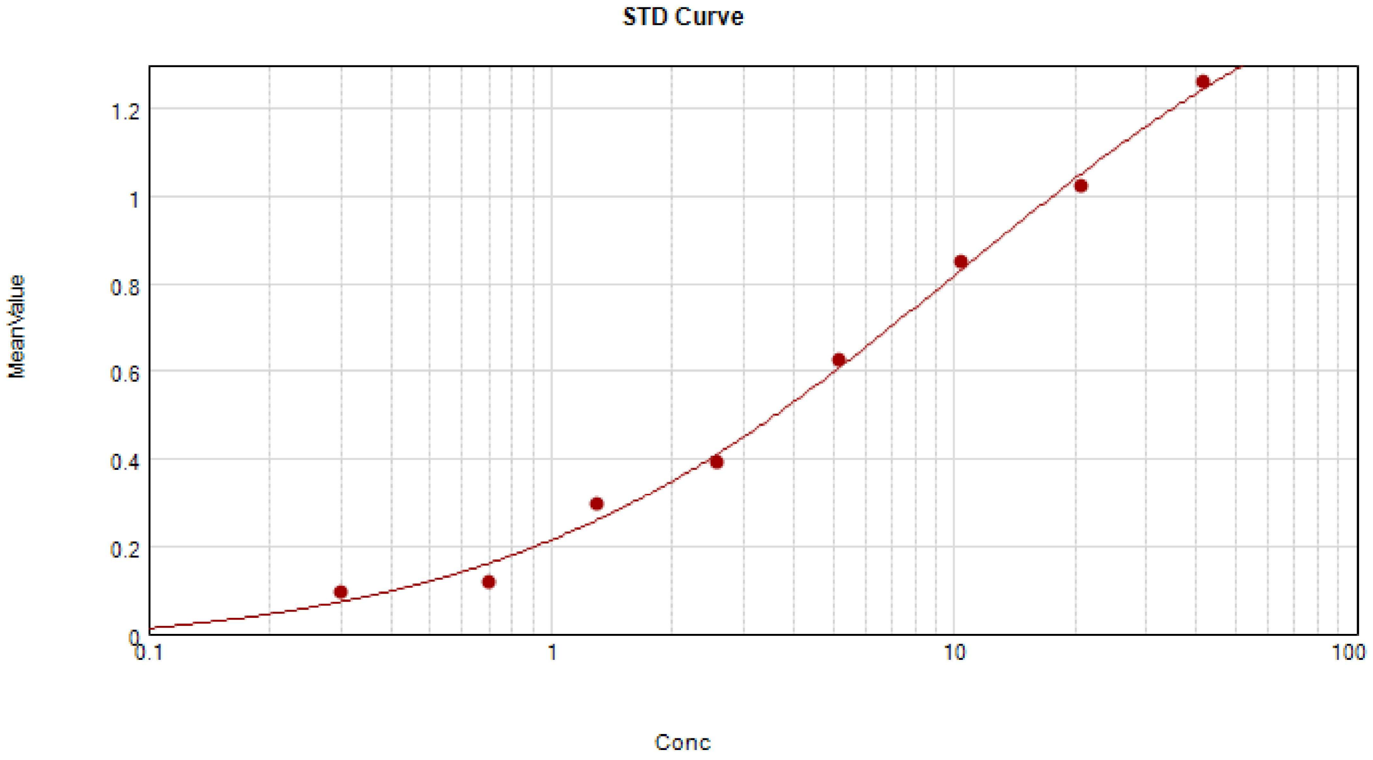

● **Std** (Standards: OD vs Th.Conc) Weighting: Fixed

Curve Fit Results ▲

Curve Fit : 4-Parameter Logistic $y = D + \frac{A - D}{1 + \left(\frac{x}{C}\right)^B}$

	Parameter	Estimated Value	Std. Error	Confidence Interval
**Std**	A	-0.036	0.098	[-0.307, 0.234]
$R^2 = 0.996$	B	0.765	0.207	[0.192, 1.338]
EC50 = 10.05	C	10.05	4.229	[-1.693, 21.79]
	D	1.679	0.309	[0.823, 2.536]

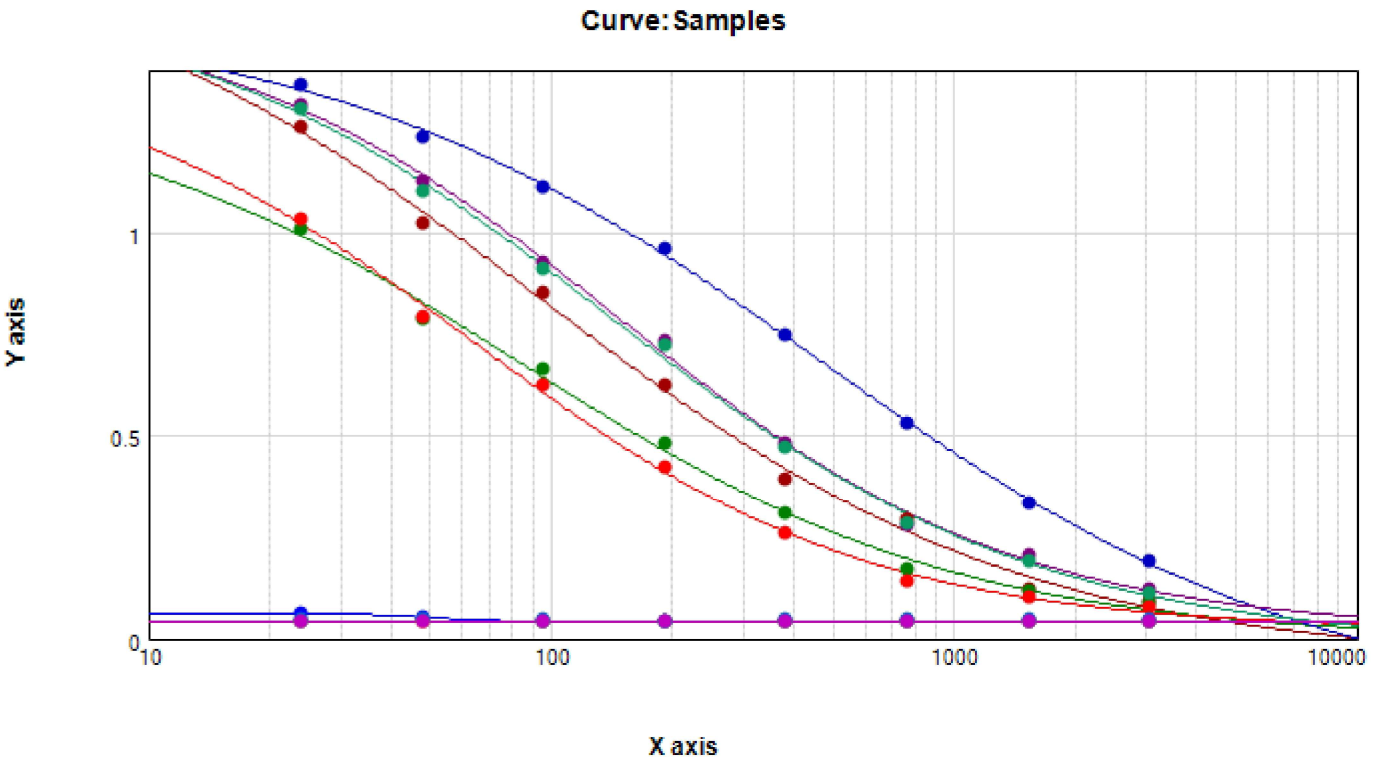

- **STD** (Standards: OD vs Dilution) Weighting: Fixed
- **S-1** (Samples: ODS1 vs DilSple1) Weighting: Fixed
- **S-2** (Samples: ODS2 vs DilSple2) Weighting: Fixed
- **S-3** (Samples: ODS3 vs DilSple3) Weighting: Fixed
- **S-4** (Samples: ODS4 vs DilSple4) Weighting: Fixed
- **S-5** (Samples: ODS5 vs DilSple5) Weighting: Fixed
- **S-6** (Samples: ODS6 vs DilSple6) Weighting: Fixed
- **S-7** (Samples: ODS7 vs DilSple7) Weighting: Fixed
- **S-8** (Samples: ODS8 vs DilSple8) Weighting: Fixed
- **S-9** (Samples: ODS9 vs DilSple9) Weighting: Fixed
- **S-10** (Samples: ODS10 vs DilSple10) Weighting: Fixed
- **S-11** (Samples: ODS11 vs DilSple11) Weighting: Fixed

Curve Fit Results ▼

Assay Parameter

Samples

Theoretical First Dilution Of Test Sample In Plate : 24.0 Sample dilution fold: 2.0

Nipha_Standard : NV-1

Concentration: 1000.0

Dilution (First dil in plate): 24.0

Dilution fold: 2.0

Others parameters

Rounding Decimal Standard Th.Conc: 1

Rounding Decimal RelErr% & CVdil: 1

Rounding Decimal GMC: 1

Average ODs of Blank: 0.042

SD of Blank: 0.001

Cutoff OD: 0.086
