# Supplementary material for: Development and Qualification of a Nipah Virus Glycoprotein-Specific IgG ELISA for the Assessment of Human Antibody Responses
Source: Vaccines (Basel). 2026 Jun 16;14(6):534. doi: 10.3390/vaccines14060534 (PMC13307770; doi:10.3390/vaccines14060534)
Supplement: Supplementary file 1 [file vaccines-14-00534-s001.zip › Supplementary_ELISA Qualification Data & Graph/2. Sensitivity and Specificity_Analysist-2/6. Sensitivity and Specificity_NHP_Analyst-2_Day-3.pdf]

Intro

NIPAH_NHP_29 AUGUST_Analyst#2_Day#3

OD

	1	2	3	4	5	6	7	8	9	10	11	12
A	1.220	1.012	1.361	1.317	1.031	1.301	0.064	0.051	0.061	0.054	0.058	0.048
B	1.020	0.789	1.237	1.128	0.791	1.103	0.054	0.045	0.055	0.043	0.052	0.046
C	0.871	0.668	1.130	0.925	0.623	0.900	0.052	0.046	0.044	0.041	0.049	0.048
D	0.629	0.480	0.961	0.733	0.420	0.727	0.053	0.049	0.043	0.044	0.043	0.046
E	0.394	0.310	0.750	0.479	0.260	0.472	0.045	0.047	0.042	0.040	0.049	0.045
F	0.290	0.172	0.532	0.281	0.140	0.282	0.044	0.041	0.043	0.041	0.047	0.044
G	0.117	0.118	0.337	0.208	0.104	0.193	0.043	0.051	0.041	0.042	0.043	0.046
H	0.092	0.082	0.191	0.121	0.077	0.112	0.048	0.048	0.044	0.043	0.045	0.048

Settings Information
Endpoint
Absorbance
Lm1 450
More Settings
Shake Off
Calibrate On
Carriage Speed Normal
Column Priority

Read Information
Imported Data : 4:45 PM 9/2/2024
Imported By : anjan

Reduction Settings

Optical Density
Wavelength Combination : !Lm1

Sample Dil

Main Sample Dilution 24.0

Sample 1: NHP-1 24.0

Sample 2: NHP-3 24.0

Sample 3: NHP-5 24.0

Sample 4: NHP-6 24.0

Sample 5: NHP-7 24.0

Sample 6: NC-5 24.0

Sample 7: NC-6 24.0

Sample 8: NC-7 24.0

Sample 9: NC-8 24.0

Sample 10: CNC 24.0

Sample 11: BLANK 24.0

Standards

Sample	Wells	OD	OK OD	Dilution	Calc.Conc	Adj.Conc	GMC	N	Th.Conc	RelErr%
01	A1	1.220	1.220	24	45.025	1080.6	979.6	7	41.700	8.000
	B1	1.020	1.020	48	18.437	885.0			20.800	-11.400
	C1	0.871	0.871	96	11.144	1069.8			10.400	7.200
	D1	0.629	0.629	192	5.295	1016.6			5.200	1.800
	E1	0.394	0.394	384	2.400	921.5			2.600	-7.700
	F1	0.290	0.290	768	1.547	1188.1			1.300	19.000
	G1	0.117	0.117	1536	0.495	760.3			0.700	-29.300
	H1	0.092		3072					0.300	

Samples

Sample	Wells	ID	OD	OK OD	Dilution	Calc.Conc	Adjusted.Conc	GMC	N	CVdil
01	A2	1	1.012	1.012	24	17.908	429.781	569.3	7	22.7
	B2		0.789	0.789	48	8.633	414.361			
	C2		0.668	0.668	96	5.969	573.041			
	D2		0.480	0.480	192	3.273	628.378			
	E2		0.310	0.310	384	1.696	651.368			
	F2		0.172	0.172	768	0.787	604.282			
	G2		0.118	0.118	1536	0.500	767.981			
	H2		0.082		3072					
02	A3	2	1.361	1.361	24	147.709	3545.016	2882.0	8	11.0
	B3		1.237	1.237	48	49.807	2390.740			
	C3		1.130	1.130	96	28.650	2750.363			
	D3		0.961	0.961	192	14.972	2874.699			
	E3		0.750	0.750	384	7.663	2942.513			
	F3		0.532	0.532	768	3.894	2990.424			
	G3		0.337	0.337	1536	1.908	2931.230			
	H3		0.191	0.191	3072	0.896	2753.749			
03	A4	3	1.317	1.317	24	90.457	2170.958	1437.3	8	20.0
	B4		1.128	1.128	48	28.396	1363.029			
	C4		0.925	0.925	96	13.270	1273.896			
	D4		0.733	0.733	192	7.276	1397.060			
	E4		0.479	0.479	384	3.262	1252.457			
	F4		0.281	0.281	768	1.482	1138.135			
	G4		0.208	0.208	1536	0.998	1533.563			
	H4		0.121	0.121	3072	0.515	1582.045			
04	A5	4	1.031	1.031	24	19.200	460.788	499.7	7	14.4
	B5		0.791	0.791	48	8.686	416.911			
	C5		0.623	0.623	96	5.197	498.938			
	D5		0.420	0.420	192	2.646	507.976			
	E5		0.260	0.260	384	1.335	512.616			
	F5		0.140	0.140	768	0.613	470.423			
	G5		0.104	0.104	1536	0.431	662.671			
	H5		0.077		3072					
05	A6	5	1.301	1.301	24	78.550	1885.193	1341.2	8	16.3
	B6		1.103	1.103	48	25.495	1223.779			
	C6		0.900	0.900	96	12.229	1173.989			
	D6		0.727	0.727	192	7.145	1371.792			
	E6		0.472	0.472	384	3.184	1222.655			
	F6		0.282	0.282	768	1.489	1143.640			
	G6		0.193	0.193	1536	0.908	1395.006			
	H6		0.112	0.112	3072	0.470	1444.803			
06	A7	6	0.064		24			N/A	0	-----
	B7		0.054		48					
	C7		0.052		96					
	D7		0.053		192					
	E7		0.045		384					
	F7		0.044		768					
	G7		0.043		1536					
	H7		0.048		3072					
07	A8	7	0.051		24			N/A	0	-----
	B8		0.045		48					
	C8		0.046		96					
	D8		0.049		192					
	E8		0.047		384					
	F8		0.041		768					
	G8		0.051		1536					
	H8		0.048		3072					
08	A9	8	0.061		24			N/A	0	-----
	B9		0.055		48					
	C9		0.044		96					
	D9		0.043		192					

Samples (Contd)

Sample	Wells	ID	OD	OK OD	Dilution	Calc.Conc	Adjusted.Conc	GMC	N	CVdil
	E9		0.042		384					
	F9		0.043		768					
	G9		0.041		1536					
	H9		0.044		3072					
09	A10	9	0.054		24			N/A	0	-----
	B10		0.043		48					
	C10		0.041		96					
	D10		0.044		192					
	E10		0.040		384					
	F10		0.041		768					
	G10		0.042		1536					
	H10		0.043		3072					
10	A11	10	0.058		24			N/A	0	-----
	B11		0.052		48					
	C11		0.049		96					
	D11		0.043		192					
	E11		0.049		384					
	F11		0.047		768					
	G11		0.043		1536					
	H11		0.045		3072					
11	A12	11	0.048		24			N/A	0	-----
	B12		0.046		48					
	C12		0.048		96					
	D12		0.046		192					
	E12		0.045		384					
	F12		0.044		768					
	G12		0.046		1536					
	H12		0.048		3072					

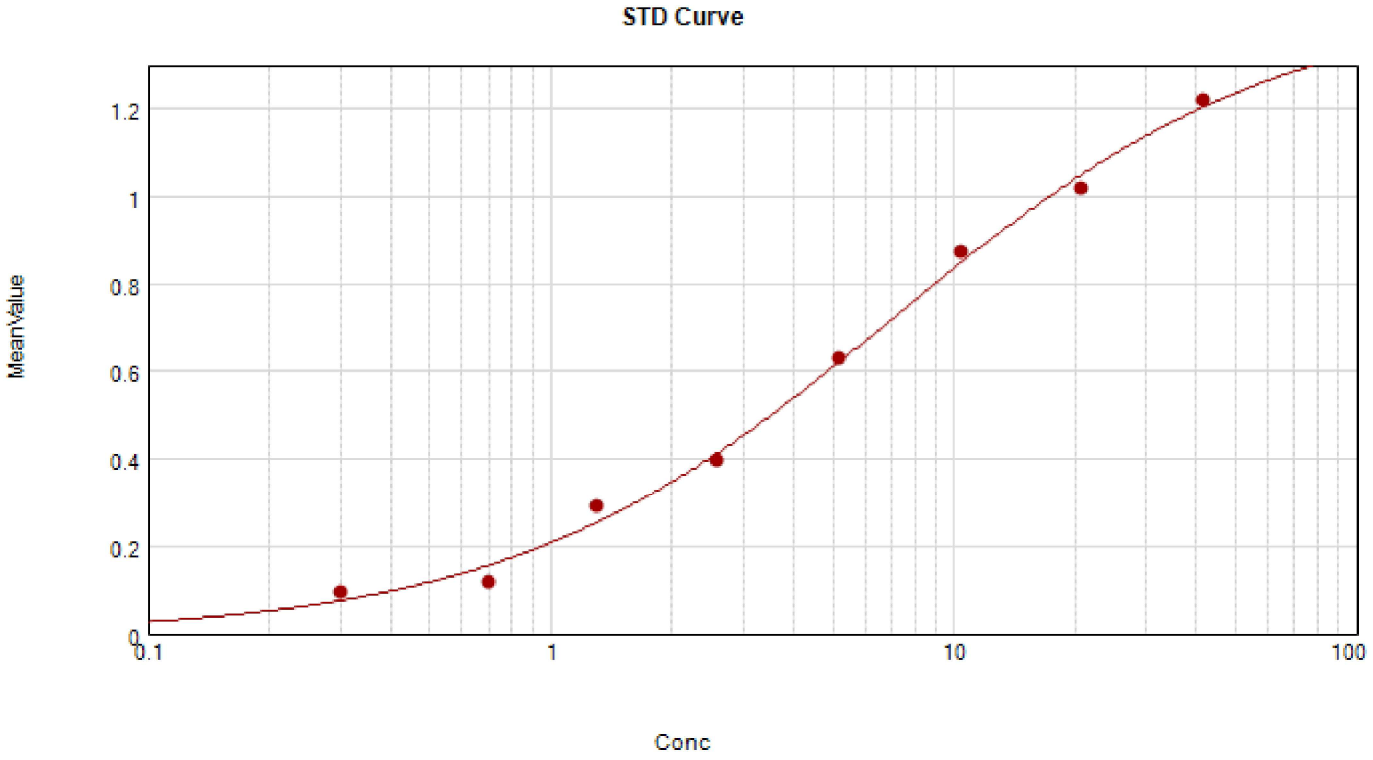

● **Std** (Standards: OD vs Th.Conc) Weighting: Fixed

Curve Fit Results ▲

Curve Fit : 4-Parameter Logistic $y = D + \frac{A - D}{1 + \left(\frac{x}{C}\right)^B}$

	Parameter	Estimated Value	Std. Error	Confidence Interval
**Std**<br>R^2 = 0.996<br>EC50 = 7.043	A	-0.003	0.074	[-0.209, 0.204]
	B	0.907	0.200	[0.353, 1.461]
	C	7.043	1.657	[2.444, 11.64]
	D	1.447	0.163	[0.995, 1.900]

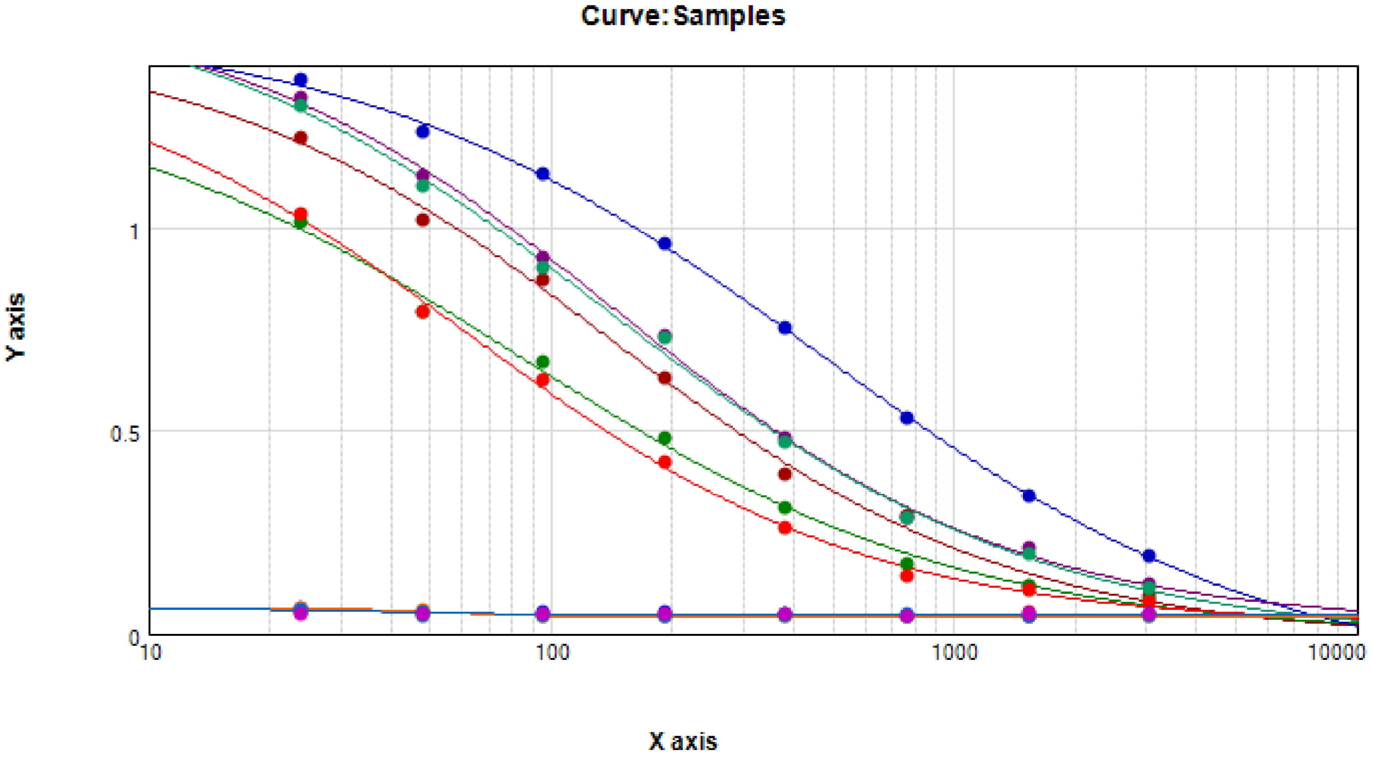

- **STD** (Standards: OD vs Dilution) Weighting: Fixed
- **S-1** (Samples: ODS1 vs DilSple1) Weighting: Fixed
- **S-2** (Samples: ODS2 vs DilSple2) Weighting: Fixed
- **S-3** (Samples: ODS3 vs DilSple3) Weighting: Fixed
- **S-4** (Samples: ODS4 vs DilSple4) Weighting: Fixed
- **S-5** (Samples: ODS5 vs DilSple5) Weighting: Fixed
- **S-6** (Samples: ODS6 vs DilSple6) Weighting: Fixed
- **S-7** (Samples: ODS7 vs DilSple7) Weighting: Fixed
- **S-8** (Samples: ODS8 vs DilSple8) Weighting: Fixed
- **S-9** (Samples: ODS9 vs DilSple9) Weighting: Fixed
- **S-10** (Samples: ODS10 vs DilSple10) Weighting: Fixed
- **S-11** (Samples: ODS11 vs DilSple11) Weighting: Fixed

Curve Fit Results ▼

Assay Parameter

Samples

Theoretical First Dilution Of Test Sample In Plate : 24.0   Sample dilution fold: 2.0

Nipha_Standard : NV-1

Concentration: 1000.0

Dilution (First dil in plate): 24.0

Dilution fold: 2.0

Others parameters

Rounding Decimal Standard Th.Conc: 1

Rounding Decimal RelErr% & CVdil: 1

Rounding Decimal GMC: 1

Average ODs of Blank: 0.046

SD of Blank: 0.002

Cutoff OD: 0.094
