# Supplementary material for: Development and Qualification of a Nipah Virus Glycoprotein-Specific IgG ELISA for the Assessment of Human Antibody Responses
Source: Vaccines (Basel). 2026 Jun 16;14(6):534. doi: 10.3390/vaccines14060534 (PMC13307770; doi:10.3390/vaccines14060534)
Supplement: Supplementary file 1 [file vaccines-14-00534-s001.zip › Supplementary_ELISA Qualification Data & Graph/2. Sensitivity and Specificity_Analysist-2/1. Sensitivity and Specificity_WHO IS_Analyst-2_Day-1.pdf]

Intro

NIPAH_NIBSC_13 AUGUST_Analyst#2_Day#1

OD

	1	2	3	4	5	6	7	8	9	10	11	12
A	1.210	1.176	0.918	0.808	0.643	0.074	0.076	0.068	0.064	0.047	0.043	0.045
B	1.016	0.991	0.790	0.701	0.501	0.062	0.059	0.061	0.053	0.041	0.046	0.046
C	0.855	0.747	0.620	0.481	0.315	0.060	0.049	0.058	0.054	0.051	0.044	0.048
D	0.601	0.515	0.451	0.304	0.198	0.049	0.043	0.051	0.052	0.048	0.047	0.043
E	0.394	0.329	0.247	0.180	0.091	0.048	0.048	0.045	0.045	0.051	0.047	0.046
F	0.297	0.207	0.167	0.092	0.084	0.043	0.046	0.044	0.048	0.045	0.042	0.046
G	0.129	0.117	0.092	0.078	0.063	0.049	0.043	0.049	0.043	0.046	0.046	0.047
H	0.092	0.090	0.076	0.064	0.052	0.047	0.044	0.047	0.044	0.049	0.043	0.045

Settings Information
Endpoint
Absorbance
Lm1 450
More Settings
Shake Off
Calibrate On
Carriage Speed Normal
Column Priority

Read Information
Imported Data : 4:49 PM 9/2/2024
Imported By : anjan

Reduction Settings

Optical Density
Wavelength Combination : !Lm1

Sample Dil

Main Sample Dilution 24.0

Sample 1: NV-2 24.0

Sample 2: NV-4 24.0

Sample 3: NV-10 24.0

Sample 4: NV-6 24.0

Sample 5: NC-1 24.0

Sample 6: NC-2 24.0

Sample 7: NC-3 24.0

Sample 8: NC-4 24.0

Sample 9: CNC 24.0

Sample 10: BLANK-2 24.0

Sample 11: BLANK 24.0

Standards

Sample	Wells	OD	OK OD	Dilution	Calc.Conc	Adj.Conc	GMC	N	Th.Conc	RelErr%
01	A1	1.210	1.210	24	43.169	1036.1	987.4	7	41.700	3.500
	B1	1.016	1.016	48	19.268	924.9			20.800	-7.400
	C1	0.855	0.855	96	11.248	1079.8			10.400	8.200
	D1	0.601	0.601	192	5.056	970.8			5.200	-2.800
	E1	0.394	0.394	384	2.429	932.7			2.600	-6.600
	F1	0.297	0.297	768	1.587	1218.6			1.300	22.100
	G1	0.129	0.129	1536	0.522	801.7			0.700	-25.400
	H1	0.092		3072					0.300	

Samples

Sample	Wells	ID	OD	OK OD	Dilution	Calc.Conc	Adjusted.Conc	GMC	N	CVdil
01	A2	1	1.176	1.176	24	36.641	879.395	766.2	7	8.7
	B2		0.991	0.991	48	17.641	846.771			
	C2		0.747	0.747	96	8.016	769.491			
	D2		0.515	0.515	192	3.796	728.911			
	E2		0.329	0.329	384	1.844	707.914			
	F2		0.207	0.207	768	0.961	738.325			
	G2		0.117	0.117	1536	0.462	710.039			
	H2		0.090		3072					
02	A3	2	0.918	0.918	24	13.786	330.872	474.4	6	20.7
	B3		0.790	0.790	48	9.166	439.968			
	C3		0.620	0.620	96	5.375	516.040			
	D3		0.451	0.451	192	3.023	580.390			
	E3		0.247	0.247	384	1.223	469.505			
	F3		0.167	0.167	768	0.725	556.651			
	G3		0.092		1536					
	H3		0.076		3072					
03	A4	3	0.808	0.808	24	9.697	232.737	299.9	5	14.6
	B4		0.701	0.701	48	6.944	333.312			
	C4		0.481	0.481	96	3.370	323.537			
	D4		0.304	0.304	192	1.641	315.127			
	E4		0.180	0.180	384	0.799	306.836			
	F4		0.092		768					
	G4		0.078		1536					
	H4		0.064		3072					
04	A5	4	0.643	0.643	24	5.785	138.837	162.4	4	10.7
	B5		0.501	0.501	48	3.616	173.591			
	C5		0.315	0.315	96	1.729	165.965			
	D5		0.198	0.198	192	0.906	173.957			
	E5		0.091		384					
	F5		0.084		768					
	G5		0.063		1536					
	H5		0.052		3072					
05	A6	5	0.074		24			N/A	0	-----
	B6		0.062		48					
	C6		0.060		96					
	D6		0.049		192					
	E6		0.048		384					
	F6		0.043		768					
	G6		0.049		1536					
	H6		0.047		3072					
06	A7	6	0.076		24			N/A	0	-----
	B7		0.059		48					
	C7		0.049		96					
	D7		0.043		192					
	E7		0.048		384					
	F7		0.046		768					
	G7		0.043		1536					
	H7		0.044		3072					
07	A8	7	0.068		24			N/A	0	-----
	B8		0.061		48					
	C8		0.058		96					
	D8		0.051		192					
	E8		0.045		384					
	F8		0.044		768					
	G8		0.049		1536					
	H8		0.047		3072					
08	A9	8	0.064		24			N/A	0	-----
	B9		0.053		48					
	C9		0.054		96					
	D9		0.052		192					

Samples (Contd)

Sample	Wells	ID	OD	OK OD	Dilution	Calc.Conc	Adjusted.Conc	GMC	N	CVdil
	E9		0.045		384					
	F9		0.048		768					
	G9		0.043		1536					
	H9		0.044		3072					
09	A10	9	0.047		24			N/A	0	-----
	B10		0.041		48					
	C10		0.051		96					
	D10		0.048		192					
	E10		0.051		384					
	F10		0.045		768					
	G10		0.046		1536					
	H10		0.049		3072					
10	A11	10	0.043		24			N/A	0	-----
	B11		0.046		48					
	C11		0.044		96					
	D11		0.047		192					
	E11		0.047		384					
	F11		0.042		768					
	G11		0.046		1536					
	H11		0.043		3072					
11	A12	11	0.045		24			N/A	0	-----
	B12		0.046		48					
	C12		0.048		96					
	D12		0.043		192					
	E12		0.046		384					
	F12		0.046		768					
	G12		0.047		1536					
	H12		0.045		3072					

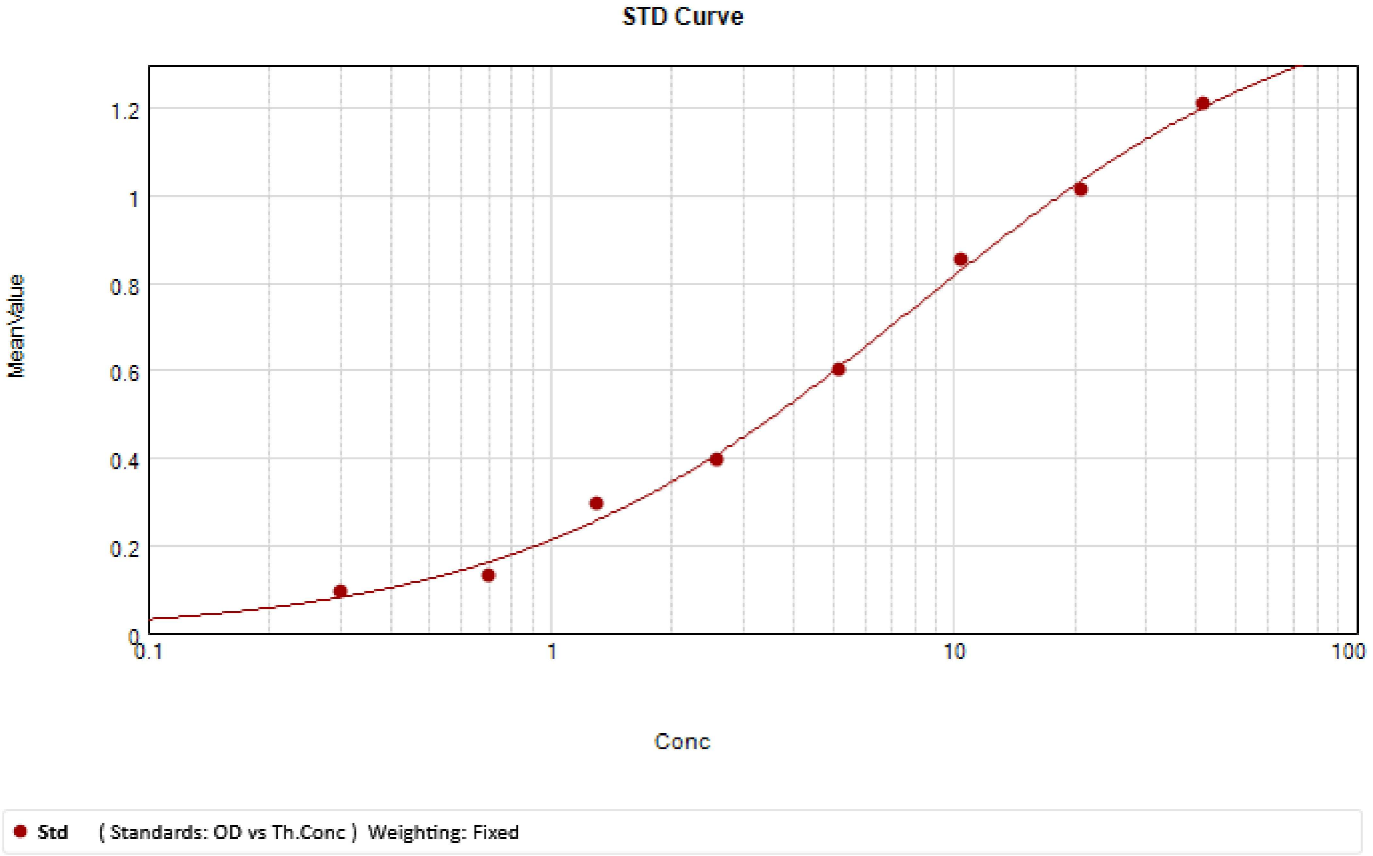

Curve Fit Results ▲

Curve Fit : 4-Parameter Logistic $y = D + \frac{A - D}{1 + \left(\frac{x}{C}\right)^B}$

	Parameter	Estimated Value	Std. Error	Confidence Interval
**Std**<br>R^2 = 0.997<br>EC50 = 8.017	A	-0.003	0.072	[-0.203, 0.197]
	B	0.856	0.184	[0.345, 1.366]
	C	8.017	2.103	[2.178, 13.86]
	D	1.497	0.182	[0.991, 2.004]

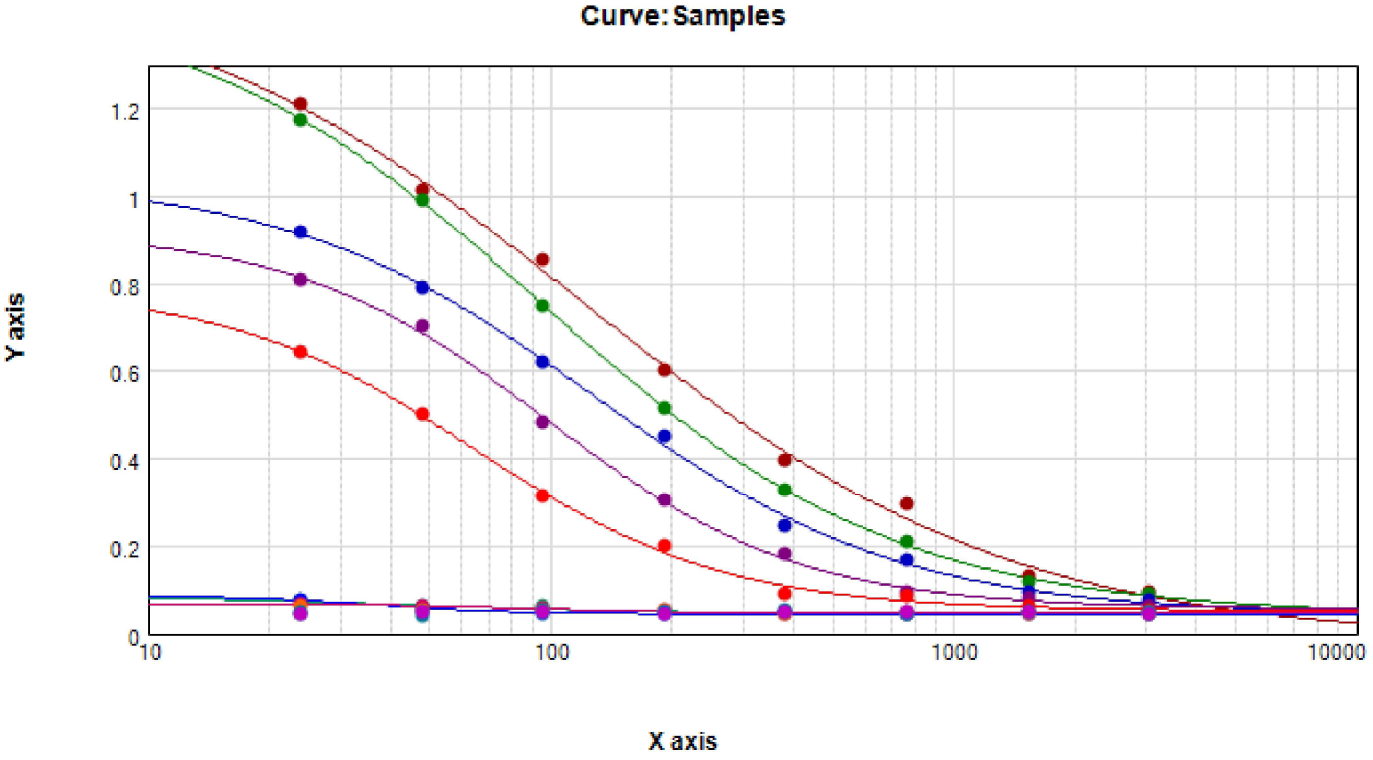

- STD (Standards: OD vs Dilution) Weighting: Fixed
- S-1 (Samples: ODS1 vs DilSple1) Weighting: Fixed
- S-2 (Samples: ODS2 vs DilSple2) Weighting: Fixed
- S-3 (Samples: ODS3 vs DilSple3) Weighting: Fixed
- S-4 (Samples: ODS4 vs DilSple4) Weighting: Fixed
- S-5 (Samples: ODS5 vs DilSple5) Weighting: Fixed
- S-6 (Samples: ODS6 vs DilSple6) Weighting: Fixed
- S-7 (Samples: ODS7 vs DilSple7) Weighting: Fixed
- S-8 (Samples: ODS8 vs DilSple8) Weighting: Fixed
- S-9 (Samples: ODS9 vs DilSple9) Weighting: Fixed
- S-10 (Samples: ODS10 vs DilSple10) Weighting: Fixed
- S-11 (Samples: ODS11 vs DilSple11) Weighting: Fixed

Curve Fit Results ▼

Assay Parameter

Samples

Theoretical First Dilution Of Test Sample In Plate : 24.0   Sample dilution fold: 2.0

Nipha_Standard : NV-1

Concentration: 1000.0

Dilution (First dil in plate): 24.0

Dilution fold: 2.0

Others parameters

Rounding Decimal Standard Th.Conc: 1

Rounding Decimal RelErr% & CVdil: 1

Rounding Decimal GMC: 1

Average ODs of Blank: 0.046

SD of Blank: 0.001

Cutoff OD: 0.093
