# Supplementary material for: Development and Qualification of a Nipah Virus Glycoprotein-Specific IgG ELISA for the Assessment of Human Antibody Responses
Source: Vaccines (Basel). 2026 Jun 16;14(6):534. doi: 10.3390/vaccines14060534 (PMC13307770; doi:10.3390/vaccines14060534)
Supplement: Supplementary file 1 [file vaccines-14-00534-s001.zip › Supplementary_ELISA Qualification Data & Graph/2. Sensitivity and Specificity_Analysist-2/2. Sensitivity and Specificity_WHO IS_Analyst-2_Day-2.pdf]

Intro

Nipah_NIBSC_14 AUGUST_Analyst#2_Day#2

OD

	1	2	3	4	5	6	7	8	9	10	11	12
A	1.230	1.180	0.914	0.810	0.647	0.072	0.077	0.069	0.064	0.051	0.047	0.047
B	1.017	0.989	0.787	0.703	0.505	0.060	0.056	0.058	0.052	0.045	0.042	0.045
C	0.853	0.743	0.618	0.482	0.313	0.062	0.043	0.060	0.054	0.046	0.046	0.048
D	0.603	0.511	0.449	0.305	0.199	0.048	0.049	0.051	0.053	0.049	0.043	0.043
E	0.395	0.327	0.245	0.181	0.091	0.049	0.043	0.049	0.045	0.047	0.043	0.046
F	0.296	0.203	0.166	0.092	0.086	0.043	0.046	0.047	0.044	0.041	0.046	0.046
G	0.127	0.115	0.091	0.076	0.064	0.047	0.048	0.045	0.043	0.051	0.044	0.045
H	0.092	0.090	0.074	0.066	0.051	0.046	0.044	0.044	0.048	0.048	0.047	0.046

Settings Information
Endpoint
Absorbance
Lm1 450
More Settings
Shake Off
Calibrate On
Carriage Speed Normal
Column Priority

Read Information
Imported Data : 4:15 PM 9/1/2024
Imported By : anjan

Reduction Settings

Optical Density
Wavelength Combination : !Lm1

Sample Dil

Main Sample Dilution 24.0

Sample 1: NV-2 24.0

Sample 2: NV-4 24.0

Sample 3: NV-10 24.0

Sample 4: NV-6 24.0

Sample 5: NC-1 24.0

Sample 6: NC-2 24.0

Sample 7: NC-3 24.0

Sample 8: NC-4 24.0

Sample 9: CNC 24.0

Sample 10: BLANK-2 24.0

Sample 11: BLANK 24.0

Standards

Sample	Wells	OD	OK OD	Dilution	Calc.Conc	Adj.Conc	GMC	N	Th.Conc	RelErr%
01	A1	1.230	1.230	24	43.394	1041.5	984.8	7	41.700	4.100
	B1	1.017	1.017	48	19.090	916.3			20.800	-8.200
	C1	0.853	0.853	96	11.231	1078.2			10.400	8.000
	D1	0.603	0.603	192	5.121	983.2			5.200	-1.500
	E1	0.395	0.395	384	2.432	933.8			2.600	-6.500
	F1	0.296	0.296	768	1.567	1203.4			1.300	20.500
	G1	0.127	0.127	1536	0.514	790.1			0.700	-26.500
	H1	0.092		3072					0.300	

Samples

Sample	Wells	ID	OD	OK OD	Dilution	Calc.Conc	Adjusted.Conc	GMC	N	CVdil
01	A2	1	1.180	1.180	24	34.955	838.927	751.2	7	8.0
	B2		0.989	0.989	48	17.375	834.019			
	C2		0.743	0.743	96	7.979	766.032			
	D2		0.511	0.511	192	3.755	720.993			
	E2		0.327	0.327	384	1.815	697.073			
	F2		0.203	0.203	768	0.929	713.364			
	G2		0.115	0.115	1536	0.457	702.216			
	H2		0.090		3072					
02	A3	2	0.914	0.914	24	13.614	326.748	470.1	6	20.8
	B3		0.787	0.787	48	9.147	439.051			
	C3		0.618	0.618	96	5.377	516.178			
	D3		0.449	0.449	192	3.000	576.073			
	E3		0.245	0.245	384	1.198	460.089			
	F3		0.166	0.166	768	0.716	549.645			
	G3		0.091		1536					
	H3		0.074		3072					
03	A4	3	0.810	0.810	24	9.824	235.765	301.7	5	14.3
	B4		0.703	0.703	48	7.044	338.101			
	C4		0.482	0.482	96	3.387	325.196			
	D4		0.305	0.305	192	1.637	314.317			
	E4		0.181	0.181	384	0.799	306.994			
	F4		0.092		768					
	G4		0.076		1536					
	H4		0.066		3072					
04	A5	4	0.647	0.647	24	5.902	141.646	163.2	4	10.0
	B5		0.505	0.505	48	3.677	176.489			
	C5		0.313	0.313	96	1.701	163.272			
	D5		0.199	0.199	192	0.905	173.712			
	E5		0.091		384					
	F5		0.086		768					
	G5		0.064		1536					
	H5		0.051		3072					
05	A6	5	0.072		24			N/A	0	-----
	B6		0.060		48					
	C6		0.062		96					
	D6		0.048		192					
	E6		0.049		384					
	F6		0.043		768					
	G6		0.047		1536					
	H6		0.046		3072					
06	A7	6	0.077		24			N/A	0	-----
	B7		0.056		48					
	C7		0.043		96					
	D7		0.049		192					
	E7		0.043		384					
	F7		0.046		768					
	G7		0.048		1536					
	H7		0.044		3072					
07	A8	7	0.069		24			N/A	0	-----
	B8		0.058		48					
	C8		0.060		96					
	D8		0.051		192					
	E8		0.049		384					
	F8		0.047		768					
	G8		0.045		1536					
	H8		0.044		3072					
08	A9	8	0.064		24			N/A	0	-----
	B9		0.052		48					
	C9		0.054		96					
	D9		0.053		192					

Samples (Contd)

Sample	Wells	ID	OD	OK OD	Dilution	Calc.Conc	Adjusted.Conc	GMC	N	CVdil
	E9		0.045		384					
	F9		0.044		768					
	G9		0.043		1536					
	H9		0.048		3072					
09	A10	9	0.051		24			N/A	0	-----
	B10		0.045		48					
	C10		0.046		96					
	D10		0.049		192					
	E10		0.047		384					
	F10		0.041		768					
	G10		0.051		1536					
	H10		0.048		3072					
10	A11	10	0.047		24			N/A	0	-----
	B11		0.042		48					
	C11		0.046		96					
	D11		0.043		192					
	E11		0.043		384					
	F11		0.046		768					
	G11		0.044		1536					
	H11		0.047		3072					
11	A12	11	0.047		24			N/A	0	-----
	B12		0.045		48					
	C12		0.048		96					
	D12		0.043		192					
	E12		0.046		384					
	F12		0.046		768					
	G12		0.045		1536					
	H12		0.046		3072					

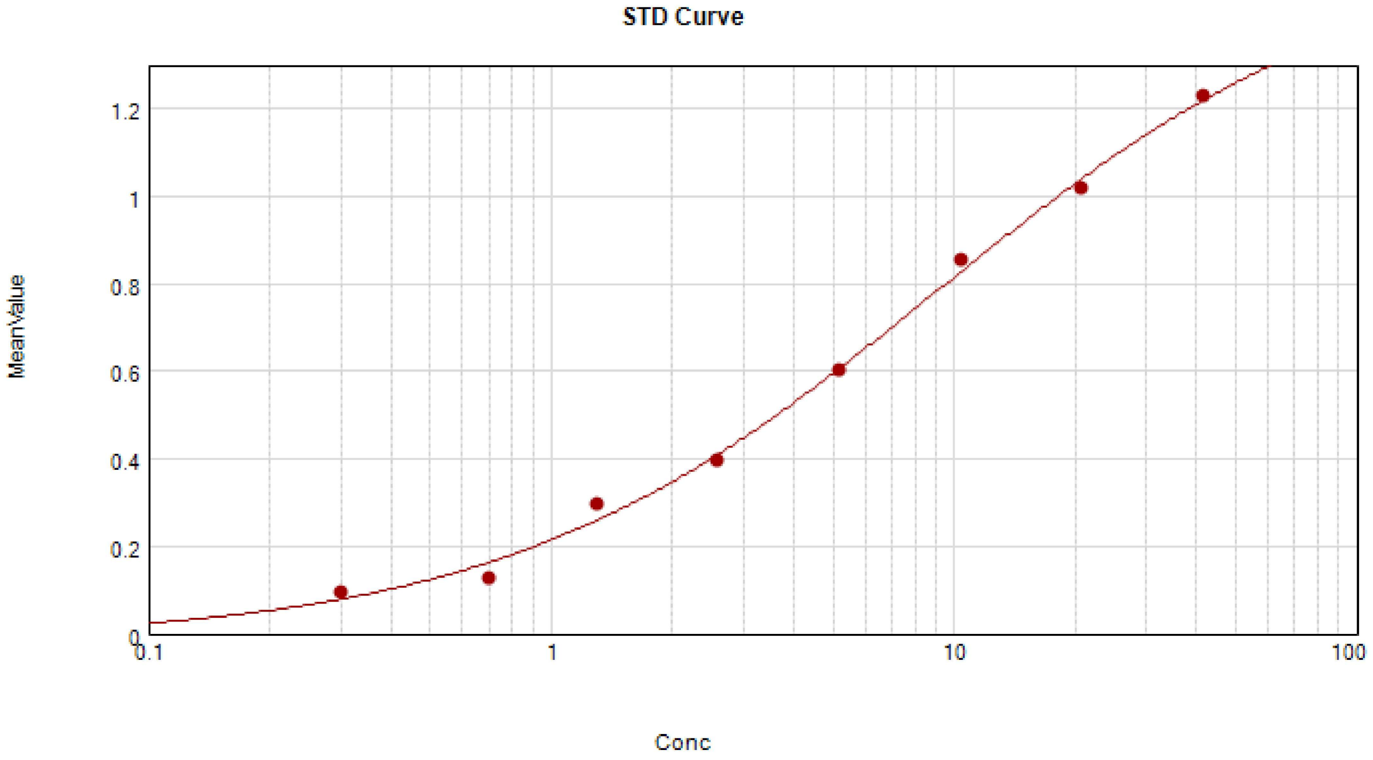

● **Std** (Standards: OD vs Th.Conc) Weighting: Fixed

Curve Fit Results ▲

Curve Fit : 4-Parameter Logistic $y = D + \frac{A - D}{1 + \left(\frac{x}{C}\right)^B}$

	Parameter	Estimated Value	Std. Error	Confidence Interval
**Std** $R^2 = 0.996$ EC50 = 8.981	A	-0.017	0.081	[-0.241, 0.207]
	B	0.808	0.188	[0.286, 1.330]
	C	8.981	2.886	[0.967, 16.99]
	D	1.579	0.229	[0.944, 2.214]

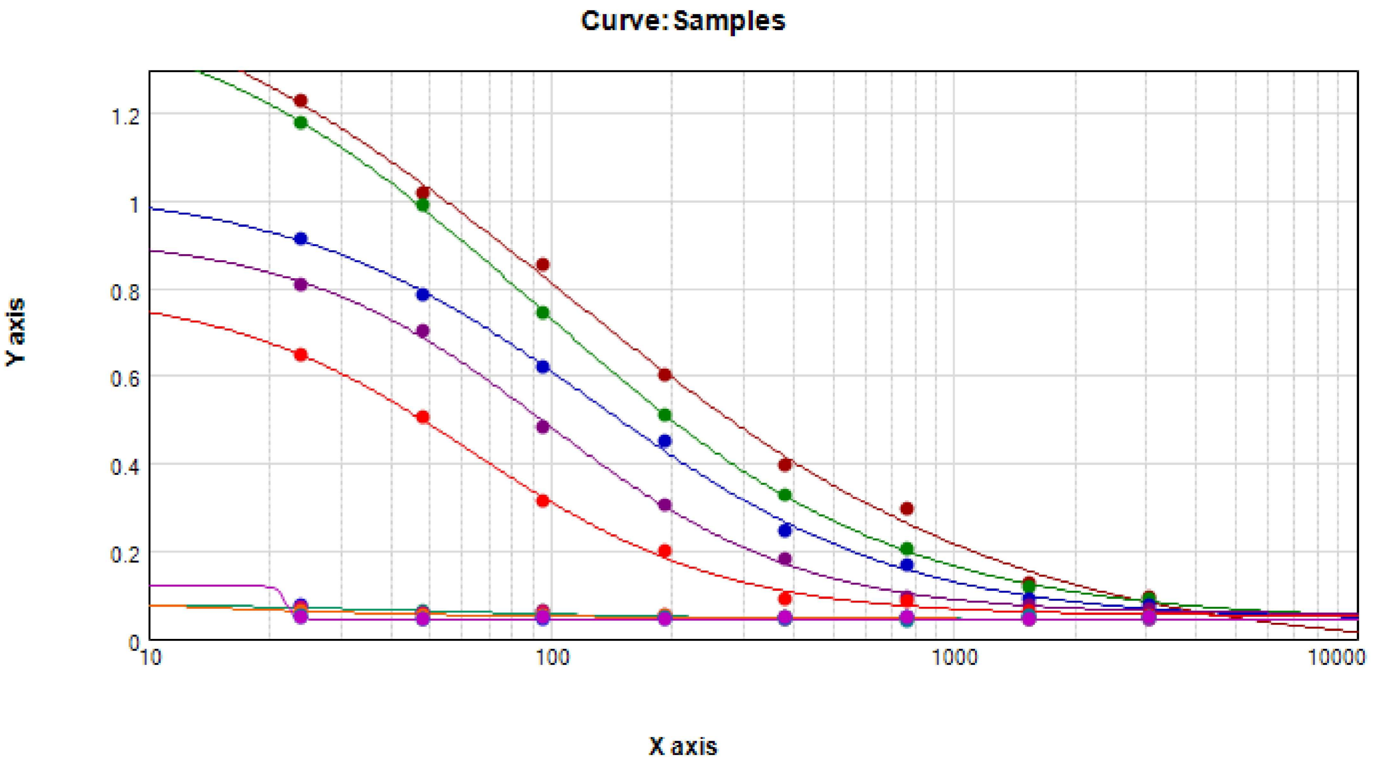

- STD (Standards: OD vs Dilution) Weighting: Fixed
- S-1 (Samples: ODS1 vs DilSple1) Weighting: Fixed
- S-2 (Samples: ODS2 vs DilSple2) Weighting: Fixed
- S-3 (Samples: ODS3 vs DilSple3) Weighting: Fixed
- S-4 (Samples: ODS4 vs DilSple4) Weighting: Fixed
- S-5 (Samples: ODS5 vs DilSple5) Weighting: Fixed
- S-6 (Samples: ODS6 vs DilSple6) Weighting: Fixed
- S-7 (Samples: ODS7 vs DilSple7) Weighting: Fixed
- S-8 (Samples: ODS8 vs DilSple8) Weighting: Fixed
- S-9 (Samples: ODS9 vs DilSple9) Weighting: Fixed
- S-10 (Samples: ODS10 vs DilSple10) Weighting: Fixed
- S-11 (Samples: ODS11 vs DilSple11) Weighting: Fixed

Curve Fit Results ▼

Assay Parameter

Samples

Theoretical First Dilution Of Test Sample In Plate : 24.0 Sample dilution fold: 2.0

Nipha_Standard : NV-1

Concentration: 1000.0

Dilution (First dil in plate): 24.0

Dilution fold: 2.0

Others parameters

Rounding Decimal Standard Th.Conc: 1

Rounding Decimal RelErr% & CVdil: 1

Rounding Decimal GMC: 1

Average ODs of Blank: 0.046

SD of Blank: 0.001

Cutoff OD: 0.093
