# Supplementary material for: Development and Qualification of a Nipah Virus Glycoprotein-Specific IgG ELISA for the Assessment of Human Antibody Responses
Source: Vaccines (Basel). 2026 Jun 16;14(6):534. doi: 10.3390/vaccines14060534 (PMC13307770; doi:10.3390/vaccines14060534)
Supplement: Supplementary file 1 [file vaccines-14-00534-s001.zip › Supplementary_ELISA Qualification Data & Graph/2. Sensitivity and Specificity_Analysist-2/3. Sensitivity and Specificity_WHO IS_Analyst-2_Day-3.pdf]

Intro

Nipah_NIBSC_15 AUGUST_Analyst#2_Day#3

OD

	1	2	3	4	5	6	7	8	9	10	11	12
A	1.200	1.178	0.917	0.807	0.645	0.075	0.077	0.078	0.064	0.043	0.045	0.044
B	1.015	0.990	0.789	0.704	0.504	0.062	0.067	0.061	0.053	0.046	0.046	0.042
C	0.851	0.744	0.621	0.480	0.314	0.060	0.061	0.061	0.054	0.044	0.048	0.044
D	0.600	0.516	0.450	0.302	0.196	0.049	0.054	0.048	0.052	0.047	0.043	0.048
E	0.393	0.327	0.244	0.182	0.090	0.047	0.045	0.047	0.045	0.047	0.046	0.042
F	0.295	0.205	0.169	0.091	0.085	0.049	0.047	0.047	0.048	0.042	0.046	0.045
G	0.125	0.116	0.092	0.075	0.061	0.048	0.044	0.047	0.043	0.046	0.047	0.046
H	0.091	0.090	0.075	0.064	0.053	0.043	0.046	0.052	0.044	0.043	0.045	0.048

Settings Information

Endpoint

Absorbance

Lm1 450

More Settings

Shake Off

Calibrate On

Carriage Speed Normal

Column Priority

Read Information

Imported Data : 4:19 PM 9/1/2024

Imported By : anjan

Reduction Settings

Optical Density

Wavelength Combination : !Lm1

Sample Dil

Main Sample Dilution 24.0

Sample 1: NV-2 24.0

Sample 2: NV-4 24.0

Sample 3: NV-10 24.0

Sample 4: NV-6 24.0

Sample 5: NC-1 24.0

Sample 6: NC-2 24.0

Sample 7: NC-3 24.0

Sample 8: NC-4 24.0

Sample 9: CNC 24.0

Sample 10: BLANK-2 24.0

Sample 11: BLANK 24.0

Standards

Sample	Wells	OD	OK OD	Dilution	Calc.Conc	Adj.Conc	GMC	N	Th.Conc	RelErr%
01	A1	1.200	1.200	24	43.001	1032.0	985.7	7	41.700	3.100
	B1	1.015	1.015	48	19.447	933.5			20.800	-6.500
	C1	0.851	0.851	96	11.155	1070.8			10.400	7.300
	D1	0.600	0.600	192	5.058	971.1			5.200	-2.700
	E1	0.393	0.393	384	2.440	937.1			2.600	-6.100
	F1	0.295	0.295	768	1.592	1222.3			1.300	22.400
	G1	0.125	0.125	1536	0.513	787.6			0.700	-26.700
	H1	0.091		3072					0.300	

Samples

Sample	Wells	ID	OD	OK OD	Dilution	Calc.Conc	Adjusted.Conc	GMC	N	CVdil
01	A2	1	1.178	1.178	24	38.458	922.988	774.5	7	9.9
	B2		0.990	0.990	48	17.771	853.019			
	C2		0.744	0.744	96	7.963	764.461			
	D2		0.516	0.516	192	3.829	735.157			
	E2		0.327	0.327	384	1.848	709.524			
	F2		0.205	0.205	768	0.966	741.600			
	G2		0.116	0.116	1536	0.468	718.108			
	H2		0.090		3072					
02	A3	2	0.917	0.917	24	13.832	331.974	478.0	6	21.3
	B3		0.789	0.789	48	9.166	439.991			
	C3		0.621	0.621	96	5.410	519.385			
	D3		0.450	0.450	192	3.032	582.219			
	E3		0.244	0.244	384	1.221	468.756			
	F3		0.169	0.169	768	0.750	576.379			
	G3		0.092		1536					
	H3		0.075		3072					
03	A4	3	0.807	0.807	24	9.700	232.807	303.0	5	15.0
	B4		0.704	0.704	48	7.028	337.368			
	C4		0.480	0.480	96	3.378	324.322			
	D4		0.302	0.302	192	1.646	316.028			
	E4		0.182	0.182	384	0.826	317.182			
	F4		0.091		768					
	G4		0.075		1536					
	H4		0.064		3072					
04	A5	4	0.645	0.645	24	5.839	140.143	163.9	4	10.7
	B5		0.504	0.504	48	3.674	176.362			
	C5		0.314	0.314	96	1.741	167.170			
	D5		0.196	0.196	192	0.910	174.731			
	E5		0.090		384					
	F5		0.085		768					
	G5		0.061		1536					
	H5		0.053		3072					
05	A6	5	0.075		24			N/A	0	-----
	B6		0.062		48					
	C6		0.060		96					
	D6		0.049		192					
	E6		0.047		384					
	F6		0.049		768					
	G6		0.048		1536					
	H6		0.043		3072					
06	A7	6	0.077		24			N/A	0	-----
	B7		0.067		48					
	C7		0.061		96					
	D7		0.054		192					
	E7		0.045		384					
	F7		0.047		768					
	G7		0.044		1536					
	H7		0.046		3072					
07	A8	7	0.078		24			N/A	0	-----
	B8		0.061		48					
	C8		0.061		96					
	D8		0.048		192					
	E8		0.047		384					
	F8		0.047		768					
	G8		0.047		1536					
	H8		0.052		3072					
08	A9	8	0.064		24			N/A	0	-----
	B9		0.053		48					
	C9		0.054		96					
	D9		0.052		192					

Samples (Contd)

Sample	Wells	ID	OD	OK OD	Dilution	Calc.Conc	Adjusted.Conc	GMC	N	CVdil
	E9		0.045		384					
	F9		0.048		768					
	G9		0.043		1536					
	H9		0.044		3072					
09	A10	9	0.043		24			N/A	0	-----
	B10		0.046		48					
	C10		0.044		96					
	D10		0.047		192					
	E10		0.047		384					
	F10		0.042		768					
	G10		0.046		1536					
	H10		0.043		3072					
10	A11	10	0.045		24			N/A	0	-----
	B11		0.046		48					
	C11		0.048		96					
	D11		0.043		192					
	E11		0.046		384					
	F11		0.046		768					
	G11		0.047		1536					
	H11		0.045		3072					
11	A12	11	0.044		24			N/A	0	-----
	B12		0.042		48					
	C12		0.044		96					
	D12		0.048		192					
	E12		0.042		384					
	F12		0.045		768					
	G12		0.046		1536					
	H12		0.048		3072					

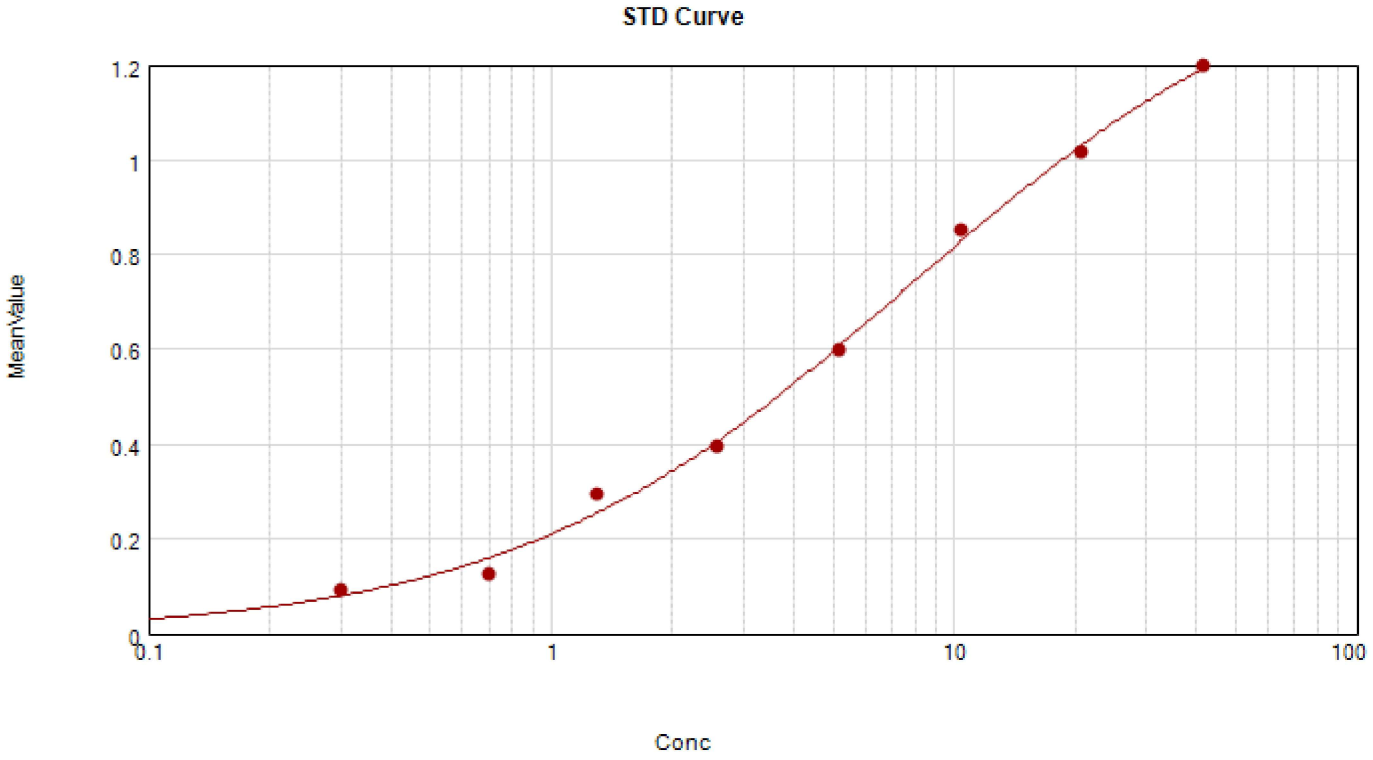

● Std (Standards: OD vs Th.Conc) Weighting: Fixed

Curve Fit Results ▲

Curve Fit : 4-Parameter Logistic $y = D + \frac{A - D}{1 + \left(\frac{x}{C}\right)^B}$

	Parameter	Estimated Value	Std. Error	Confidence Interval
**Std**<br>$R^2 = 0.997$<br>EC50 = 7.701	A	-0.001	0.069	[-0.193, 0.190]
	B	0.873	0.181	[0.370, 1.376]
	C	7.701	1.868	[2.514, 12.89]
	D	1.468	0.167	[1.004, 1.931]

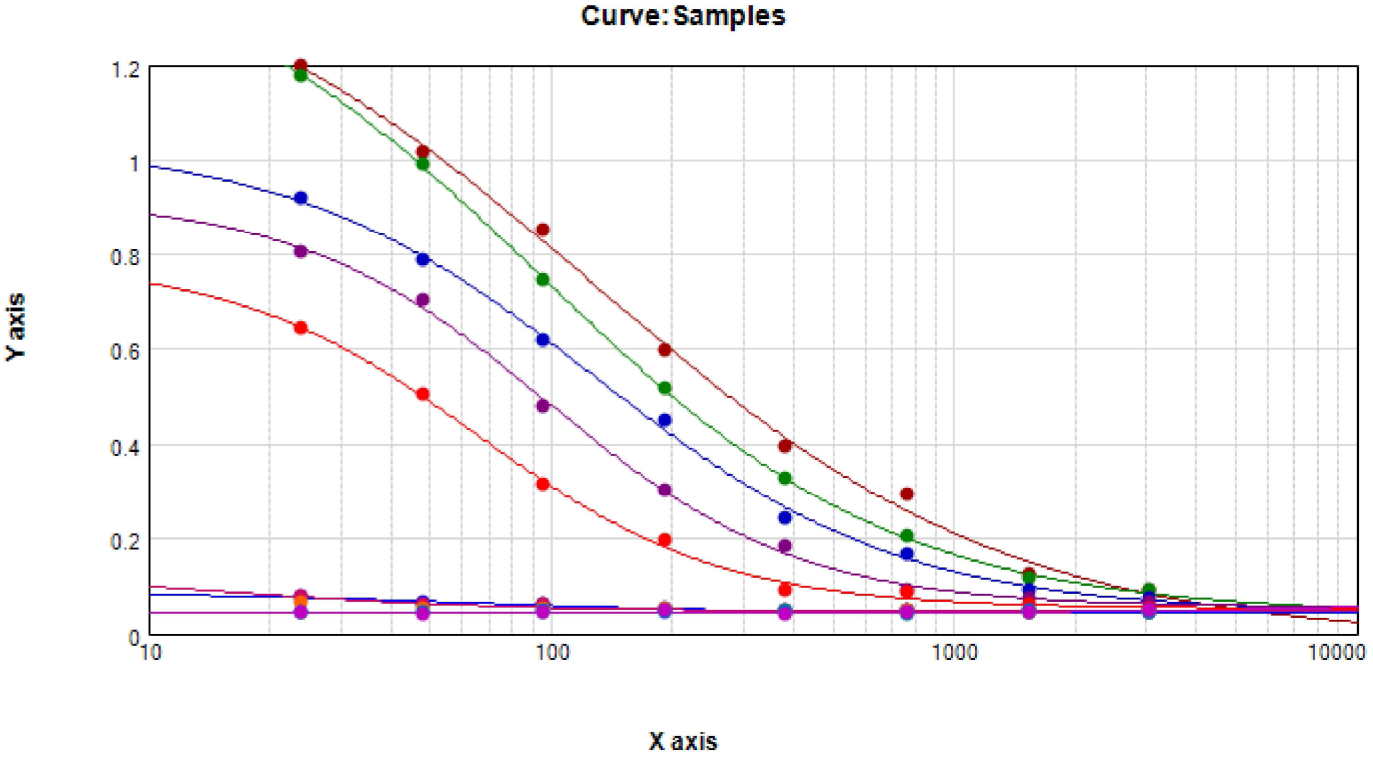

- **STD** (Standards: OD vs Dilution) Weighting: Fixed
- **S-1** (Samples: ODS1 vs DilSple1) Weighting: Fixed
- **S-2** (Samples: ODS2 vs DilSple2) Weighting: Fixed
- **S-3** (Samples: ODS3 vs DilSple3) Weighting: Fixed
- **S-4** (Samples: ODS4 vs DilSple4) Weighting: Fixed
- **S-5** (Samples: ODS5 vs DilSple5) Weighting: Fixed
- **S-6** (Samples: ODS6 vs DilSple6) Weighting: Fixed
- **S-7** (Samples: ODS7 vs DilSple7) Weighting: Fixed
- **S-8** (Samples: ODS8 vs DilSple8) Weighting: Fixed
- **S-9** (Samples: ODS9 vs DilSple9) Weighting: Fixed
- **S-10** (Samples: ODS10 vs DilSple10) Weighting: Fixed
- **S-11** (Samples: ODS11 vs DilSple11) Weighting: Fixed

Curve Fit Results ▼

Assay Parameter

Samples

Theoretical First Dilution Of Test Sample In Plate : 24.0   Sample dilution fold: 2.0

Nipha_Standard : NV-1

Concentration: 1000.0

Dilution (First dil in plate): 24.0

Dilution fold: 2.0

Others parameters

Rounding Decimal Standard Th.Conc: 1

Rounding Decimal RelErr% & CVdil: 1

Rounding Decimal GMC: 1

Average ODs of Blank: 0.045

SD of Blank: 0.002

Cutoff OD: 0.092
