# Supplementary material for: Development and Qualification of a Nipah Virus Glycoprotein-Specific IgG ELISA for the Assessment of Human Antibody Responses
Source: Vaccines (Basel). 2026 Jun 16;14(6):534. doi: 10.3390/vaccines14060534 (PMC13307770; doi:10.3390/vaccines14060534)
Supplement: Supplementary file 1 [file vaccines-14-00534-s001.zip › Supplementary_ELISA Qualification Data & Graph/6. Precision_Analysist-2/3. Precision_WHO IS_ANALYST-2_PLATE-3.pdf]

Intro

NIPAH_NIBSC_ANALYST#2_PLATE#3

OD

	1	2	3	4	5	6	7	8	9	10	11	12
A	0.969	0.557	0.562	0.384	0.387	0.501	0.507	0.052	0.055	0.047	0.042	0.047
B	0.737	0.324	0.345	0.272	0.281	0.312	0.314	0.049	0.053	0.045	0.044	0.047
C	0.596	0.239	0.248	0.174	0.185	0.276	0.275	0.047	0.045	0.046	0.047	0.043
D	0.417	0.154	0.156	0.108	0.110	0.146	0.143	0.048	0.043	0.043	0.043	0.041
E	0.251	0.113	0.115	0.081	0.083	0.117	0.110	0.042	0.044	0.043	0.046	0.046
F	0.158	0.080	0.081	0.074	0.071	0.082	0.083	0.043	0.043	0.041	0.042	0.044
G	0.089	0.058	0.058	0.058	0.062	0.060	0.066	0.042	0.047	0.042	0.042	0.043
H	0.078	0.050	0.052	0.052	0.052	0.052	0.047	0.049	0.051	0.042	0.043	0.043

Settings Information
Endpoint
Absorbance
Lm1 450
More Settings
Shake Off
Calibrate On
Carriage Speed Normal
Column Priority

Read Information
Imported Data : 4:24 PM 9/22/2024
Imported By : anjan

Reduction Settings

Optical Density
Wavelength Combination : !Lm1

Sample Dil

Main Sample Dilution 50.0

Sample 1: NV-2 120.0

Sample 2: NV-2 120.0

Sample 3: NV-10 50.0

Sample 4: NV-10 50.0

Sample 5: NV-4 120.0

Sample 6: NV-4 120.0

Sample 7: NC-1 60.0

Sample 8: NC-1 60.0

Sample 9: CNC 60.0

Sample 10: CNC 60.0

Sample 11: BLANK 50.0

Standards

Sample	Wells	OD	OK OD	Dilution	Calc.Conc	Adj.Conc	GMC	N	Th.Conc	RelErr%
01	A1	0.969	0.969	50	20.800	1040.0	989.8	6	20.000	4.000
	B1	0.737	0.737	100	8.993	899.3			10.000	-10.100
	C1	0.596	0.596	200	5.430	1086.0			5.000	8.600
	D1	0.417	0.417	400	2.676	1070.3			2.500	7.000
	E1	0.251	0.251	800	1.151	921.0			1.300	-11.400
	F1	0.158	0.158	1600	0.587	939.2			0.600	-2.200
	G1	0.089		3200					0.300	
	H1	0.078		6400					0.200	

Samples

Sample	Wells	ID	OD	OK OD	Dilution	Calc.Conc	Adjusted.Conc	GMC	N	CVdil
01	A2	1	0.557	0.557	120	4.697	563.608	542.6	5	20.0
	B2		0.324	0.324	240	1.729	415.017			
	C2		0.239	0.239	480	1.068	512.769			
	D2		0.154	0.154	960	0.567	543.975			
	E2		0.113	0.113	1920	0.375	720.771			
	F2		0.080		3840					
	G2		0.058		7680					
	H2		0.050		15360					
02	A3	2	0.562	0.562	120	4.786	574.305	567.1	5	17.0
	B3		0.345	0.345	240	1.921	460.963			
	C3		0.248	0.248	480	1.130	542.515			
	D3		0.156	0.156	960	0.577	553.717			
	E3		0.115	0.115	1920	0.384	737.266			
	F3		0.081		3840					
	G3		0.058		7680					
	H3		0.052		15360					
03	A4	3	0.384	0.384	120	2.310	277.152	312.3	4	8.7
	B4		0.272	0.272	240	1.304	313.060			
	C4		0.174	0.174	480	0.672	322.369			
	D4		0.108	0.108	960	0.354	340.083			
	E4		0.081		1920					
	F4		0.074		3840					
	G4		0.058		7680					
	H4		0.052		15360					
04	A5	4	0.387	0.387	120	2.341	280.971	326.3	4	10.4
	B5		0.281	0.281	240	1.373	329.564			
	C5		0.185	0.185	480	0.733	351.681			
	D5		0.110	0.110	960	0.363	348.150			
	E5		0.083		1920					
	F5		0.071		3840					
	G5		0.062		7680					
	H5		0.052		15360					
05	A6	5	0.501	0.501	120	3.786	454.284	533.7	5	26.9
	B6		0.312	0.312	240	1.625	390.036			
	C6		0.276	0.276	480	1.335	640.676			
	D6		0.146	0.146	960	0.527	505.745			
	E6		0.117	0.117	1920	0.393	753.906			
	F6		0.082		3840					
	G6		0.060		7680					
	H6		0.052		15360					
06	A7	6	0.507	0.507	120	3.876	465.134	525.3	5	23.7
	B7		0.314	0.314	240	1.642	394.136			
	C7		0.275	0.275	480	1.327	637.020			
	D7		0.143	0.143	960	0.512	491.712			
	E7		0.110	0.110	1920	0.363	696.299			
	F7		0.083		3840					
	G7		0.066		7680					
	H7		0.047		15360					
07	A8	7	0.052		120			N/A	0	-----
	B8		0.049		240					
	C8		0.047		480					
	D8		0.048		960					
	E8		0.042		1920					
	F8		0.043		3840					
	G8		0.042		7680					
	H8		0.049		15360					
08	A9	8	0.055		120			N/A	0	-----
	B9		0.053		240					
	C9		0.045		480					
	D9		0.043		960					

Samples (Contd)

Sample	Wells	ID	OD	OK OD	Dilution	Calc.Conc	Adjusted.Conc	GMC	N	CVdil
	E9		0.044		1920					
	F9		0.043		3840					
	G9		0.047		7680					
	H9		0.051		15360					
09	A10	9	0.047		120			N/A	0	-----
	B10		0.045		240					
	C10		0.046		480					
	D10		0.043		960					
	E10		0.043		1920					
	F10		0.041		3840					
	G10		0.042		7680					
	H10		0.042		15360					
10	A11	10	0.042		120			N/A	0	-----
	B11		0.044		240					
	C11		0.047		480					
	D11		0.043		960					
	E11		0.046		1920					
	F11		0.042		3840					
	G11		0.042		7680					
	H11		0.043		15360					
11	A12	11	0.047		120			N/A	0	-----
	B12		0.047		240					
	C12		0.043		480					
	D12		0.041		960					
	E12		0.046		1920					
	F12		0.044		3840					
	G12		0.043		7680					
	H12		0.043		15360					

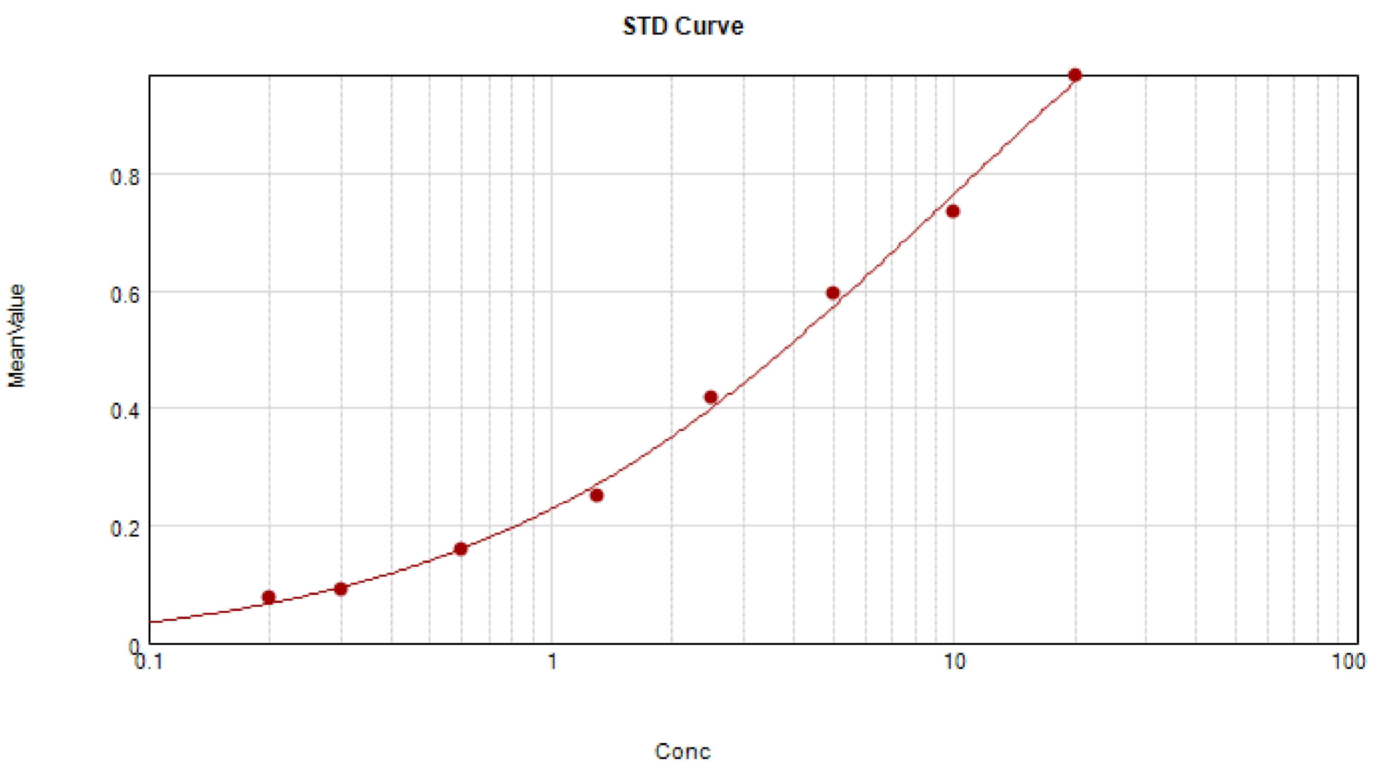

● **Std** (Standards: OD vs Th.Conc) Weighting: Fixed

Curve Fit Results ▲

Curve Fit : 4-Parameter Logistic $y = D + \frac{A - D}{1 + \left(\frac{x}{C}\right)^B}$

	Parameter	Estimated Value	Std. Error	Confidence Interval
**Std**<br>R^2 = 0.997<br>EC50 = 9.503	A	-0.017	0.067	[-0.202, 0.168]
	B	0.738	0.210	[0.154, 1.321]
	C	9.503	6.564	[-8.721, 27.73]
	D	1.522	0.422	[0.350, 2.695]

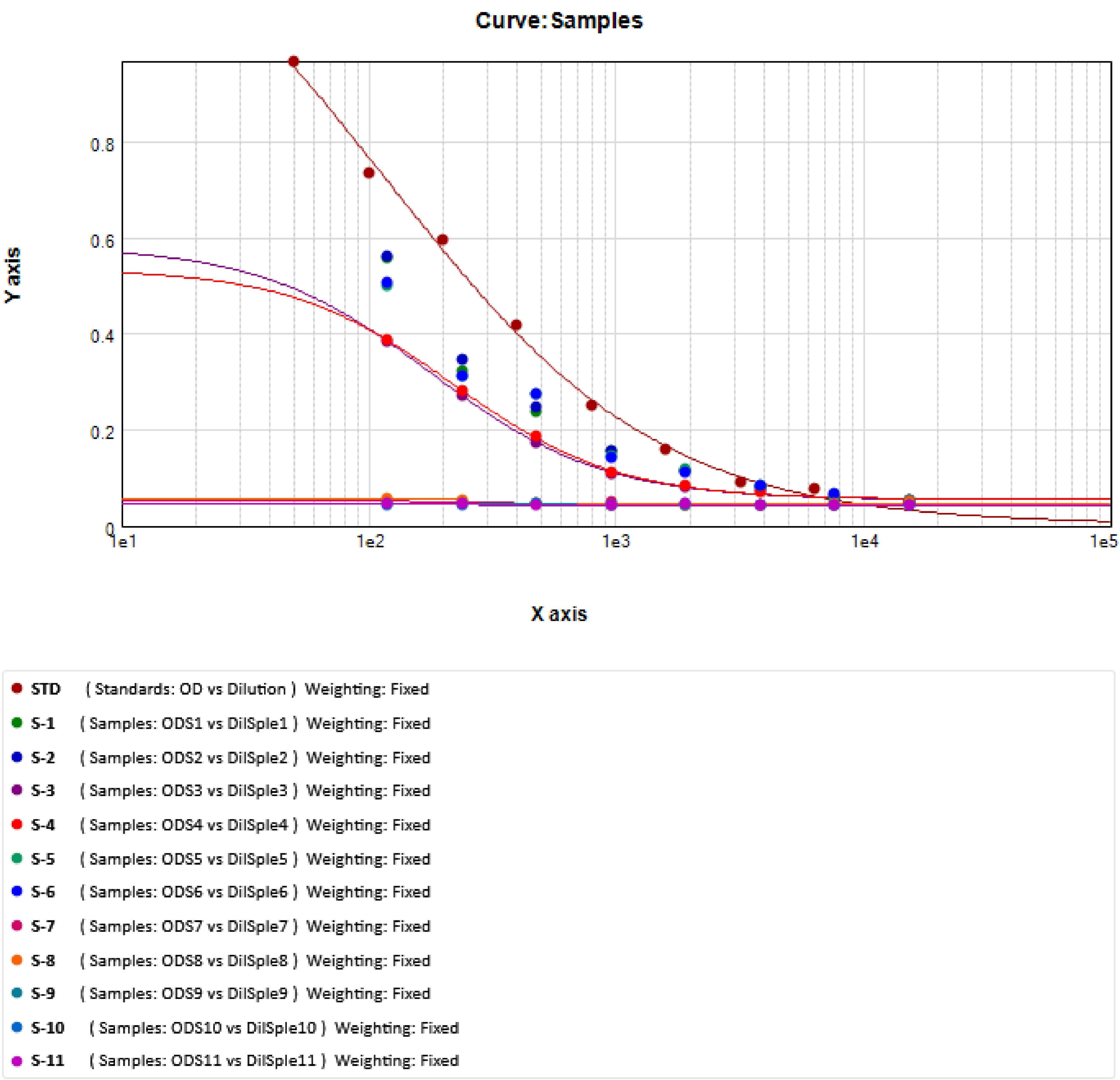

Curve Fit Results ▼

Assay Parameter

Samples

Theoretical First Dilution Of Test Sample In Plate : 50.0   Sample dilution fold: 2.0

Nipha_Standard : NV-1

Concentration: 1000.0

Dilution (First dil in plate): 50.0

Dilution fold: 2.0

Others parameters

Rounding Decimal Standard Th.Conc: 1

Rounding Decimal RelErr% & CVdil: 1

Rounding Decimal GMC: 1

Average ODs of Blank: 0.044

SD of Blank: 0.002

Cutoff OD: 0.091
