# Supplementary material for: Development and Qualification of a Nipah Virus Glycoprotein-Specific IgG ELISA for the Assessment of Human Antibody Responses
Source: Vaccines (Basel). 2026 Jun 16;14(6):534. doi: 10.3390/vaccines14060534 (PMC13307770; doi:10.3390/vaccines14060534)
Supplement: Supplementary file 1 [file vaccines-14-00534-s001.zip › Supplementary_ELISA Qualification Data & Graph/6. Precision_Analysist-2/1. Precision_WHO IS_ANALYST-2_PLATE-1.pdf]

Intro

NIPAH_NIBSC_ANALYST#2_PLATE#1

OD

	1	2	3	4	5	6	7	8	9	10	11	12
A	0.978	0.564	0.557	0.376	0.382	0.504	0.507	0.053	0.055	0.052	0.049	0.042
B	0.737	0.332	0.346	0.282	0.276	0.314	0.312	0.051	0.050	0.049	0.045	0.045
C	0.563	0.247	0.243	0.177	0.172	0.275	0.274	0.051	0.048	0.051	0.051	0.043
D	0.420	0.157	0.159	0.112	0.120	0.142	0.148	0.052	0.046	0.043	0.044	0.047
E	0.243	0.123	0.124	0.084	0.082	0.115	0.112	0.046	0.047	0.050	0.044	0.041
F	0.132	0.084	0.082	0.058	0.060	0.077	0.079	0.048	0.044	0.049	0.045	0.048
G	0.092	0.067	0.063	0.054	0.053	0.064	0.060	0.047	0.047	0.042	0.048	0.048
H	0.083	0.056	0.057	0.041	0.047	0.050	0.053	0.047	0.043	0.045	0.045	0.047

Settings Information
Endpoint
Absorbance
Lm1 450
More Settings
Shake Off
Calibrate On
Carriage Speed Normal
Column Priority

Read Information
Imported Data : 4:02 PM 9/22/2024
Imported By : anjan

Reduction Settings

Optical Density
Wavelength Combination : !Lm1

Sample Dil

Main Sample Dilution 50.0

Sample 1: NV-2 120.0

Sample 2: NV-2 120.0

Sample 3: NV-10 50.0

Sample 4: NV-10 50.0

Sample 5: NV-4 120.0

Sample 6: NV-4 120.0

Sample 7: NC-1 60.0

Sample 8: NC-1 60.0

Sample 9: CNC 60.0

Sample 10: CNC 60.0

Sample 11: BLANK 50.0

Standards

Sample	Wells	OD	OK OD	Dilution	Calc.Conc	Adj.Conc	GMC	N	Th.Conc	RelErr%
01	A1	0.978	0.978	50	20.613	1030.7	966.3	6	20.000	3.100
	B1	0.737	0.737	100	9.269	926.9			10.000	-7.300
	C1	0.563	0.563	200	5.085	1017.1			5.000	1.700
	D1	0.420	0.420	400	2.902	1160.9			2.500	16.100
	E1	0.243	0.243	800	1.171	937.1			1.300	-9.900
	F1	0.132	0.132	1600	0.481	770.1			0.600	-19.800
	G1	0.092		3200					0.300	
	H1	0.083		6400					0.200	

Samples

Sample	Wells	ID	OD	OK OD	Dilution	Calc.Conc	Adjusted.Conc	GMC	N	CVdil
01	A2	1	0.564	0.564	120	5.104	612.468	604.8	5	21.5
	B2		0.332	0.332	240	1.932	463.671			
	C2		0.247	0.247	480	1.201	576.661			
	D2		0.157	0.157	960	0.614	589.140			
	E2		0.123	0.123	1920	0.437	838.777			
	F2		0.084		3840					
	G2		0.067		7680					
	H2		0.056		15360					
02	A3	2	0.557	0.557	120	4.975	597.028	610.6	5	20.1
	B3		0.346	0.346	240	2.071	496.962			
	C3		0.243	0.243	480	1.171	562.272			
	D3		0.159	0.159	960	0.625	599.848			
	E3		0.124	0.124	1920	0.442	848.102			
	F3		0.082		3840					
	G3		0.063		7680					
	H3		0.057		15360					
03	A4	3	0.376	0.376	120	2.387	286.458	338.7	4	11.4
	B4		0.282	0.282	240	1.480	355.204			
	C4		0.177	0.177	480	0.729	349.936			
	D4		0.112	0.112	960	0.385	369.427			
	E4		0.084		1920					
	F4		0.058		3840					
	G4		0.054		7680					
	H4		0.041		15360					
04	A5	4	0.382	0.382	120	2.454	294.446	342.5	4	13.2
	B5		0.276	0.276	240	1.430	343.230			
	C5		0.172	0.172	480	0.699	335.713			
	D5		0.120	0.120	960	0.422	405.522			
	E5		0.082		1920					
	F5		0.060		3840					
	G5		0.053		7680					
	H5		0.047		15360					
05	A6	5	0.504	0.504	120	4.079	489.442	560.5	5	25.0
	B6		0.314	0.314	240	1.762	422.789			
	C6		0.275	0.275	480	1.422	682.510			
	D6		0.142	0.142	960	0.533	511.393			
	E6		0.115	0.115	1920	0.399	765.624			
	F6		0.077		3840					
	G6		0.064		7680					
	H6		0.050		15360					
06	A7	6	0.507	0.507	120	4.126	495.117	562.4	5	23.5
	B7		0.312	0.312	240	1.743	418.377			
	C7		0.274	0.274	480	1.414	678.572			
	D7		0.148	0.148	960	0.565	541.949			
	E7		0.112	0.112	1920	0.385	738.854			
	F7		0.079		3840					
	G7		0.060		7680					
	H7		0.053		15360					
07	A8	7	0.053		120			N/A	0	-----
	B8		0.051		240					
	C8		0.051		480					
	D8		0.052		960					
	E8		0.046		1920					
	F8		0.048		3840					
	G8		0.047		7680					
	H8		0.047		15360					
08	A9	8	0.055		120			N/A	0	-----
	B9		0.050		240					
	C9		0.048		480					
	D9		0.046		960					

Samples (Contd)

Sample	Wells	ID	OD	OK OD	Dilution	Calc.Conc	Adjusted.Conc	GMC	N	CVdil
	E9		0.047		1920					
	F9		0.044		3840					
	G9		0.047		7680					
	H9		0.043		15360					
09	A10	9	0.052		120			N/A	0	-----
	B10		0.049		240					
	C10		0.051		480					
	D10		0.043		960					
	E10		0.050		1920					
	F10		0.049		3840					
	G10		0.042		7680					
	H10		0.045		15360					
10	A11	10	0.049		120			N/A	0	-----
	B11		0.045		240					
	C11		0.051		480					
	D11		0.044		960					
	E11		0.044		1920					
	F11		0.045		3840					
	G11		0.048		7680					
	H11		0.045		15360					
11	A12	11	0.042		120			N/A	0	-----
	B12		0.045		240					
	C12		0.043		480					
	D12		0.047		960					
	E12		0.041		1920					
	F12		0.048		3840					
	G12		0.048		7680					
	H12		0.047		15360					

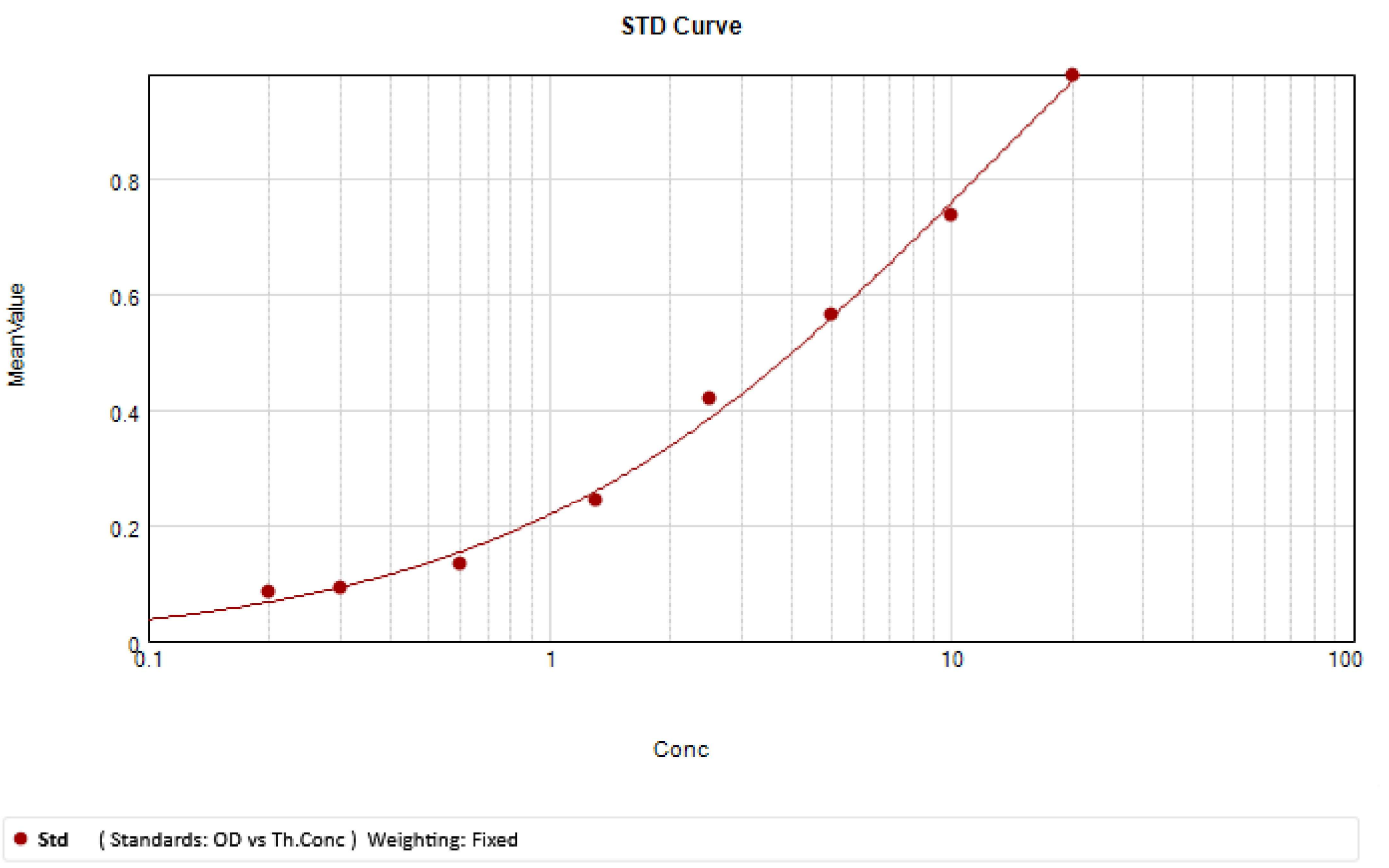

Curve Fit Results ▲

Curve Fit : 4-Parameter Logistic $y = D + \frac{A - D}{1 + \left(\frac{x}{C}\right)^B}$

	Parameter	Estimated Value	Std. Error	Confidence Interval
**Std**<br>R^2 = 0.996<br>EC50 = 12.29	A	-0.011	0.070	[-0.207, 0.184]
	B	0.730	0.233	[0.081, 1.378]
	C	12.29	11.35	[-19.21, 43.79]
	D	1.656	0.601	[-0.012, 3.324]

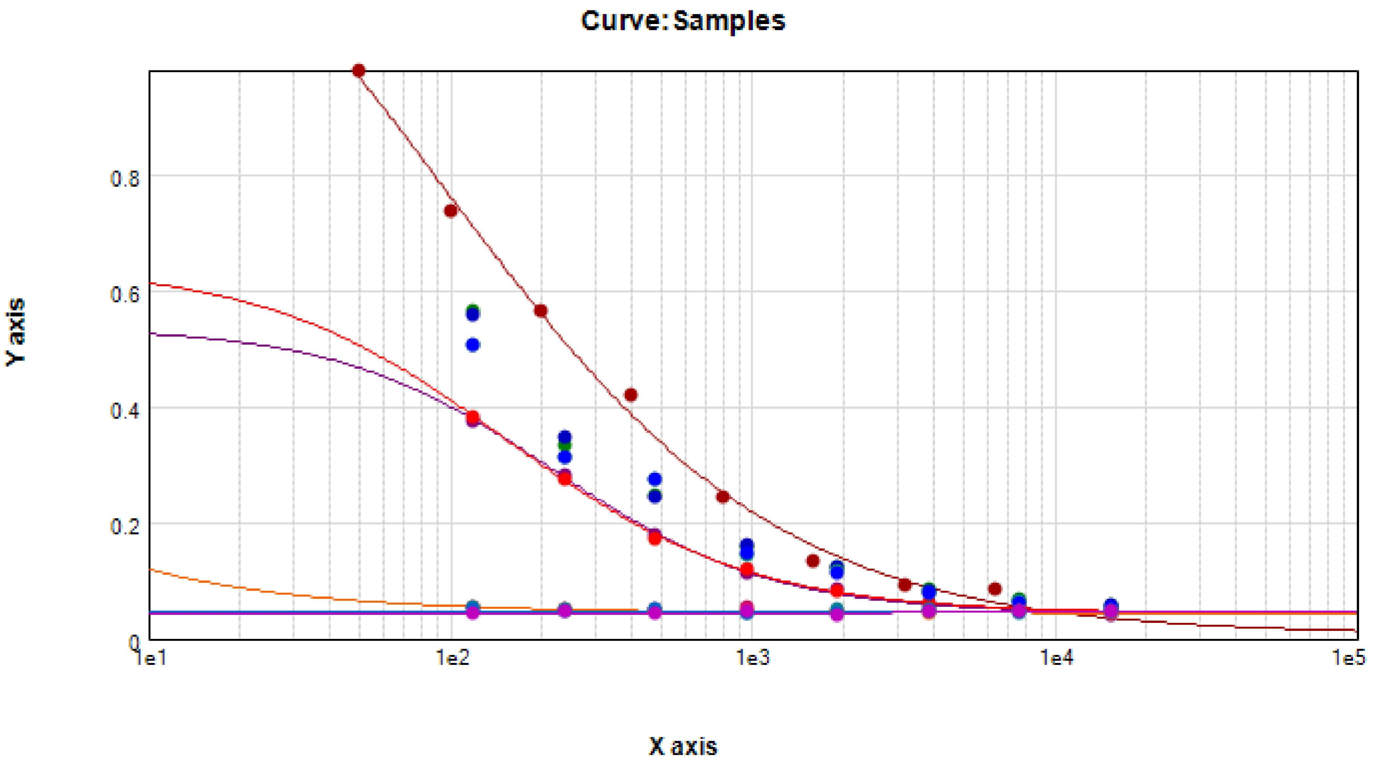

- **STD** (Standards: OD vs Dilution) Weighting: Fixed
- **S-1** (Samples: ODS1 vs DilSple1) Weighting: Fixed
- **S-2** (Samples: ODS2 vs DilSple2) Weighting: Fixed
- **S-3** (Samples: ODS3 vs DilSple3) Weighting: Fixed
- **S-4** (Samples: ODS4 vs DilSple4) Weighting: Fixed
- **S-5** (Samples: ODS5 vs DilSple5) Weighting: Fixed
- **S-6** (Samples: ODS6 vs DilSple6) Weighting: Fixed
- **S-7** (Samples: ODS7 vs DilSple7) Weighting: Fixed
- **S-8** (Samples: ODS8 vs DilSple8) Weighting: Fixed
- **S-9** (Samples: ODS9 vs DilSple9) Weighting: Fixed
- **S-10** (Samples: ODS10 vs DilSple10) Weighting: Fixed
- **S-11** (Samples: ODS11 vs DilSple11) Weighting: Fixed

Curve Fit Results ▼

Assay Parameter

Samples

Theoretical First Dilution Of Test Sample In Plate : 50.0 Sample dilution fold: 2.0

Nipha_Standard : NV-1

Concentration: 1000.0

Dilution (First dil in plate): 50.0

Dilution fold: 2.0

Others parameters

Rounding Decimal Standard Th.Conc: 1

Rounding Decimal RelErr% & CVdil: 1

Rounding Decimal GMC: 1

Average ODs of Blank: 0.045

SD of Blank: 0.003

Cutoff OD: 0.093
