# Supplementary material for: Development and Qualification of a Nipah Virus Glycoprotein-Specific IgG ELISA for the Assessment of Human Antibody Responses
Source: Vaccines (Basel). 2026 Jun 16;14(6):534. doi: 10.3390/vaccines14060534 (PMC13307770; doi:10.3390/vaccines14060534)
Supplement: Supplementary file 1 [file vaccines-14-00534-s001.zip › Supplementary_ELISA Qualification Data & Graph/6. Precision_Analysist-2/2. Precision_WHO IS_ANALYST-2_PLATE-2.pdf]

Intro

NIPAH_NIBSC_ANALYST#2_PLATE#2

OD

	1	2	3	4	5	6	7	8	9	10	11	12
A	1.010	0.502	0.495	0.325	0.320	0.463	0.435	0.050	0.056	0.050	0.044	0.049
B	0.754	0.321	0.318	0.204	0.202	0.285	0.324	0.049	0.054	0.049	0.051	0.049
C	0.529	0.199	0.192	0.146	0.153	0.171	0.172	0.048	0.048	0.041	0.044	0.047
D	0.323	0.124	0.115	0.112	0.108	0.113	0.111	0.046	0.043	0.052	0.049	0.046
E	0.194	0.099	0.098	0.082	0.085	0.098	0.101	0.041	0.049	0.043	0.049	0.049
F	0.119	0.076	0.077	0.060	0.056	0.076	0.080	0.049	0.043	0.048	0.047	0.048
G	0.081	0.058	0.050	0.050	0.054	0.056	0.053	0.042	0.047	0.043	0.041	0.040
H	0.071	0.051	0.047	0.046	0.044	0.046	0.045	0.042	0.041	0.049	0.041	0.043

Settings Information

Endpoint

Absorbance

Lm1 450

More Settings

Shake Off

Calibrate On

Carriage Speed Normal

Column Priority

Read Information

Imported Data : 4:13 PM 9/19/2024

Imported By : anjan

Reduction Settings

Optical Density

Wavelength Combination : !Lm1

Sample Dil

Main Sample Dilution 50.0

Sample 1: NV-2 120.0

Sample 2: NV-2 120.0

Sample 3: NV-10 50.0

Sample 4: NV-10 50.0

Sample 5: NV-4 120.0

Sample 6: NV-4 120.0

Sample 7: NC-1 60.0

Sample 8: NC-1 60.0

Sample 9: CNC 60.0

Sample 10: CNC 60.0

Sample 11: BLANK 50.0

Standards

Sample	Wells	OD	OK OD	Dilution	Calc.Conc	Adj.Conc	GMC	N	Th.Conc	RelErr%
01	A1	1.010	1.010	50	20.179	1009.0	994.6	6	20.000	0.900
	B1	0.754	0.754	100	9.745	974.5			10.000	-2.500
	C1	0.529	0.529	200	5.168	1033.5			5.000	3.400
	D1	0.323	0.323	400	2.498	999.1			2.500	-0.100
	E1	0.194	0.194	800	1.238	990.0			1.300	-4.800
	F1	0.119	0.119	1600	0.602	963.2			0.600	0.300
	G1	0.081		3200					0.300	
	H1	0.071		6400					0.200	

Samples

Sample	Wells	ID	OD	OK OD	Dilution	Calc.Conc	Adjusted.Conc	GMC	N	CVdil
01	A2	1	0.502	0.502	120	4.756	570.772	642.2	5	16.0
	B2		0.321	0.321	240	2.476	594.279			
	C2		0.199	0.199	480	1.282	615.416			
	D2		0.124	0.124	960	0.643	616.920			
	E2		0.099	0.099	1920	0.442	847.904			
	F2		0.076		3840					
	G2		0.058		7680					
	H2		0.051		15360					
02	A3	2	0.495	0.495	120	4.653	558.406	614.1	5	17.4
	B3		0.318	0.318	240	2.444	586.572			
	C3		0.192	0.192	480	1.220	585.498			
	D3		0.115	0.115	960	0.570	546.879			
	E3		0.098	0.098	1920	0.434	832.663			
	F3		0.077		3840					
	G3		0.050		7680					
	H3		0.047		15360					
03	A4	3	0.325	0.325	120	2.519	302.307	375.8	4	25.4
	B4		0.204	0.204	240	1.327	318.480			
	C4		0.146	0.146	480	0.824	395.569			
	D4		0.112	0.112	960	0.546	523.680			
	E4		0.082		1920					
	F4		0.060		3840					
	G4		0.050		7680					
	H4		0.046		15360					
04	A5	4	0.320	0.320	120	2.465	295.853	373.3	4	24.7
	B5		0.202	0.202	240	1.309	314.162			
	C5		0.153	0.153	480	0.883	423.764			
	D5		0.108	0.108	960	0.513	492.859			
	E5		0.085		1920					
	F5		0.056		3840					
	G5		0.054		7680					
	H5		0.044		15360					
05	A6	5	0.463	0.463	120	4.200	503.965	561.7	5	22.4
	B6		0.285	0.285	240	2.101	504.164			
	C6		0.171	0.171	480	1.036	497.400			
	D6		0.113	0.113	960	0.554	531.405			
	E6		0.098	0.098	1920	0.434	832.663			
	F6		0.076		3840					
	G6		0.056		7680					
	H6		0.046		15360					
06	A7	6	0.435	0.435	120	3.825	458.957	574.9	5	26.1
	B7		0.324	0.324	240	2.508	602.024			
	C7		0.172	0.172	480	1.045	501.541			
	D7		0.111	0.111	960	0.537	515.963			
	E7		0.101	0.101	1920	0.458	878.428			
	F7		0.080		3840					
	G7		0.053		7680					
	H7		0.045		15360					
07	A8	7	0.050		120			N/A	0	-----
	B8		0.049		240					
	C8		0.048		480					
	D8		0.046		960					
	E8		0.041		1920					
	F8		0.049		3840					
	G8		0.042		7680					
	H8		0.042		15360					
08	A9	8	0.056		120			N/A	0	-----
	B9		0.054		240					
	C9		0.048		480					
	D9		0.043		960					

Samples (Contd)

Sample	Wells	ID	OD	OK OD	Dilution	Calc.Conc	Adjusted.Conc	GMC	N	CVdil
	E9		0.049		1920					
	F9		0.043		3840					
	G9		0.047		7680					
	H9		0.041		15360					
09	A10	9	0.050		120			N/A	0	-----
	B10		0.049		240					
	C10		0.041		480					
	D10		0.052		960					
	E10		0.043		1920					
	F10		0.048		3840					
	G10		0.043		7680					
	H10		0.049		15360					
10	A11	10	0.044		120			N/A	0	-----
	B11		0.051		240					
	C11		0.044		480					
	D11		0.049		960					
	E11		0.049		1920					
	F11		0.047		3840					
	G11		0.041		7680					
	H11		0.041		15360					
11	A12	11	0.049		120			N/A	0	-----
	B12		0.049		240					
	C12		0.047		480					
	D12		0.046		960					
	E12		0.049		1920					
	F12		0.048		3840					
	G12		0.040		7680					
	H12		0.043		15360					

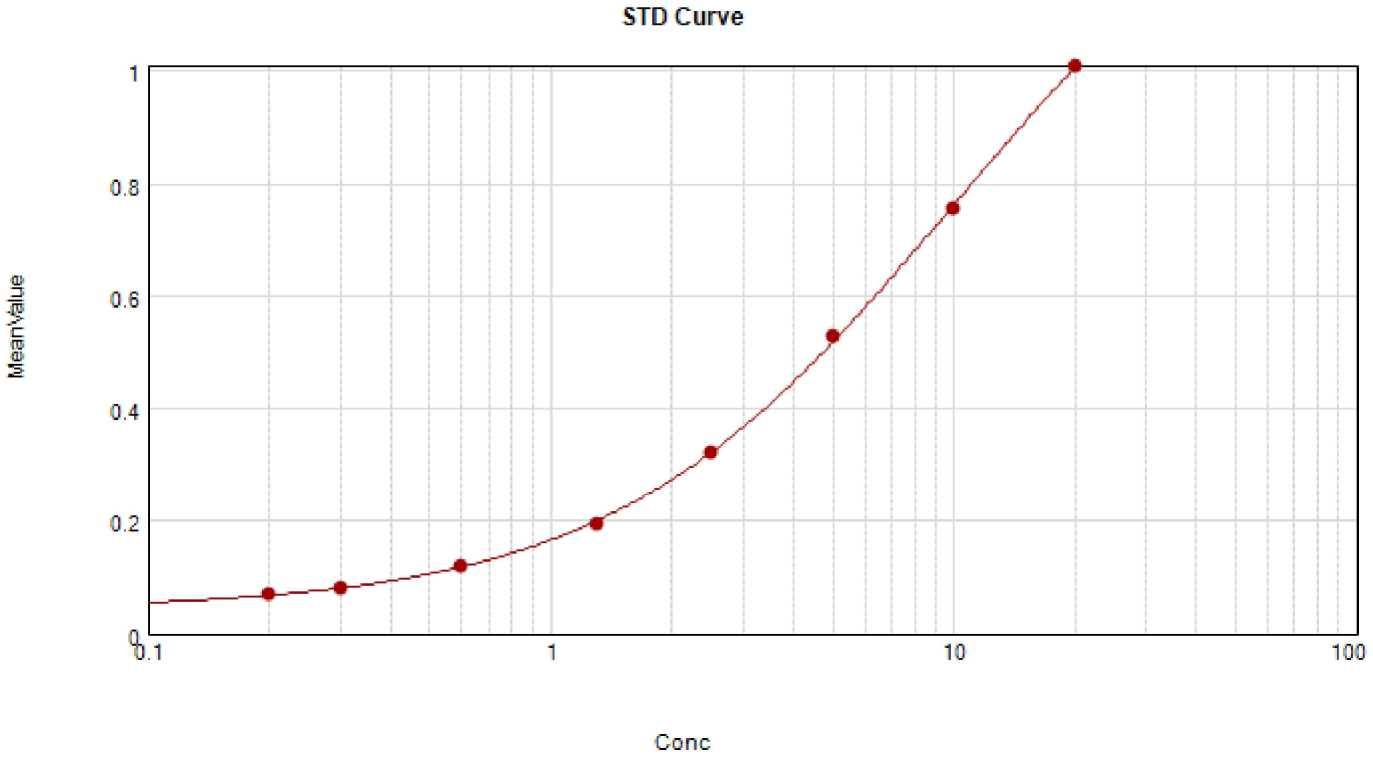

● **Std** (Standards: OD vs Th.Conc) Weighting: Fixed

Curve Fit Results ▲

Curve Fit : 4-Parameter Logistic $y = D + \frac{A - D}{1 + \left(\frac{x}{C}\right)^B}$

	Parameter	Estimated Value	Std. Error	Confidence Interval
**Std** R^2 = 1.000 EC50 = 9.809	A	0.043	0.010	[0.015, 0.071]
	B	1.030	0.066	[0.848, 1.213]
	C	9.809	1.233	[6.386, 13.23]
	D	1.470	0.090	[1.221, 1.719]

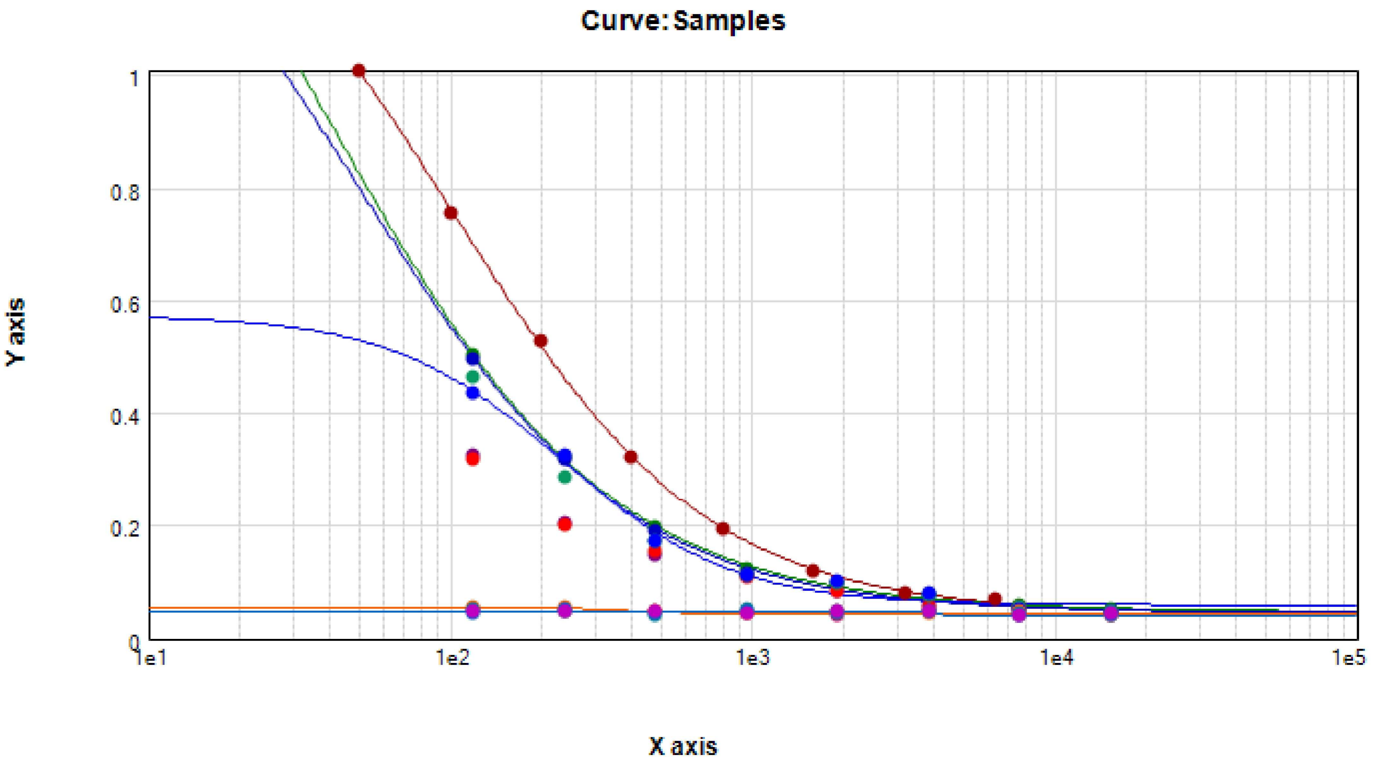

- **STD** (Standards: OD vs Dilution) Weighting: Fixed
- **S-1** (Samples: ODS1 vs DilSple1) Weighting: Fixed
- **S-2** (Samples: ODS2 vs DilSple2) Weighting: Fixed
- **S-3** (Samples: ODS3 vs DilSple3) Weighting: Fixed
- **S-4** (Samples: ODS4 vs DilSple4) Weighting: Fixed
- **S-5** (Samples: ODS5 vs DilSple5) Weighting: Fixed
- **S-6** (Samples: ODS6 vs DilSple6) Weighting: Fixed
- **S-7** (Samples: ODS7 vs DilSple7) Weighting: Fixed
- **S-8** (Samples: ODS8 vs DilSple8) Weighting: Fixed
- **S-9** (Samples: ODS9 vs DilSple9) Weighting: Fixed
- **S-10** (Samples: ODS10 vs DilSple10) Weighting: Fixed
- **S-11** (Samples: ODS11 vs DilSple11) Weighting: Fixed

Curve Fit Results ▼

Assay Parameter

Samples

Theoretical First Dilution Of Test Sample In Plate : 50.0   Sample dilution fold: 2.0

Nipha_Standard : NV-1

Concentration: 1000.0

Dilution (First dil in plate): 50.0

Dilution fold: 2.0

Others parameters

Rounding Decimal Standard Th.Conc: 1

Rounding Decimal RelErr% & CVdil: 1

Rounding Decimal GMC: 1

Average ODs of Blank: 0.046

SD of Blank: 0.003

Cutoff OD: 0.096
