# Supplementary material for: Development and Qualification of a Nipah Virus Glycoprotein-Specific IgG ELISA for the Assessment of Human Antibody Responses
Source: Vaccines (Basel). 2026 Jun 16;14(6):534. doi: 10.3390/vaccines14060534 (PMC13307770; doi:10.3390/vaccines14060534)
Supplement: Supplementary file 1 [file vaccines-14-00534-s001.zip › Supplementary_ELISA Qualification Data & Graph/5. Precision_Analysist-1/3. Precision_WHO IS_ANALYST-1_PLATE-3.pdf]

Intro

NIPAH_NIBSC_ANALYST#1_PLATE#3

OD

	1	2	3	4	5	6	7	8	9	10	11	12
A	0.968	0.553	0.562	0.386	0.394	0.503	0.517	0.052	0.054	0.050	0.049	0.041
B	0.732	0.324	0.341	0.273	0.291	0.312	0.315	0.053	0.046	0.046	0.045	0.047
C	0.592	0.235	0.245	0.171	0.184	0.278	0.274	0.047	0.043	0.049	0.041	0.047
D	0.413	0.155	0.158	0.107	0.113	0.142	0.141	0.045	0.040	0.043	0.050	0.043
E	0.256	0.112	0.113	0.071	0.073	0.110	0.108	0.041	0.040	0.047	0.042	0.047
F	0.152	0.081	0.083	0.060	0.060	0.072	0.072	0.042	0.041	0.048	0.039	0.050
G	0.091	0.066	0.058	0.053	0.055	0.062	0.056	0.044	0.045	0.042	0.041	0.042
H	0.082	0.056	0.054	0.041	0.047	0.050	0.053	0.044	0.043	0.045	0.043	0.051

Settings Information
Endpoint
Absorbance
Lm1 450
More Settings
Shake Off
Calibrate On
Carriage Speed Normal
Column Priority

Read Information
Imported Data : 4:00 PM 9/22/2024
Imported By : anjan

Reduction Settings

Optical Density
Wavelength Combination : !Lm1

Sample Dil

Main Sample Dilution 50.0

Sample 1: NV-2 120.0

Sample 2: NV-2 120.0

Sample 3: NV-10 50.0

Sample 4: NV-10 50.0

Sample 5: NV-4 120.0

Sample 6: NV-4 120.0

Sample 7: NC-1 60.0

Sample 8: NC-1 60.0

Sample 9: CNC 60.0

Sample 10: CNC 60.0

Sample 11: BLANK 50.0

Standards

Sample	Wells	OD	OK OD	Dilution	Calc.Conc	Adj.Conc	GMC	N	Th.Conc	RelErr%
01	A1	0.968	0.968	50	20.799	1040.0	984.3	6	20.000	4.000
	B1	0.732	0.732	100	8.976	897.6			10.000	-10.200
	C1	0.592	0.592	200	5.439	1087.9			5.000	8.800
	D1	0.413	0.413	400	2.663	1065.0			2.500	6.500
	E1	0.256	0.256	800	1.191	952.7			1.300	-8.400
	F1	0.152	0.152	1600	0.552	882.5			0.600	-8.100
	G1	0.091		3200					0.300	
	H1	0.082		6400					0.200	

Samples

Sample	Wells	ID	OD	OK OD	Dilution	Calc.Conc	Adjusted.Conc	GMC	N	CVdil
01	A2	1	0.553	0.553	120	4.702	564.182	537.9	5	18.9
	B2		0.324	0.324	240	1.744	418.453			
	C2		0.235	0.235	480	1.043	500.542			
	D2		0.155	0.155	960	0.567	544.147			
	E2		0.112	0.112	1920	0.365	700.464			
	F2		0.081		3840					
	G2		0.066		7680					
	H2		0.056		15360					
02	A3	2	0.562	0.562	120	4.864	583.693	562.5	5	16.0
	B3		0.341	0.341	240	1.901	456.177			
	C3		0.245	0.245	480	1.112	533.770			
	D3		0.158	0.158	960	0.582	558.989			
	E3		0.113	0.113	1920	0.369	708.689			
	F3		0.083		3840					
	G3		0.058		7680					
	H3		0.054		15360					
03	A4	3	0.386	0.386	120	2.358	282.918	310.0	4	6.5
	B4		0.273	0.273	240	1.318	316.366			
	C4		0.171	0.171	480	0.651	312.656			
	D4		0.107	0.107	960	0.344	329.951			
	E4		0.071		1920					
	F4		0.060		3840					
	G4		0.053		7680					
	H4		0.041		15360					
04	A5	4	0.394	0.394	120	2.445	293.458	335.5	4	9.0
	B5		0.291	0.291	240	1.461	350.572			
	C5		0.184	0.184	480	0.724	347.467			
	D5		0.113	0.113	960	0.369	354.345			
	E5		0.073		1920					
	F5		0.060		3840					
	G5		0.055		7680					
	H5		0.047		15360					
05	A6	5	0.503	0.503	120	3.874	464.912	523.3	5	23.9
	B6		0.312	0.312	240	1.637	392.971			
	C6		0.278	0.278	480	1.357	651.349			
	D6		0.142	0.142	960	0.502	481.824			
	E6		0.110	0.110	1920	0.356	684.125			
	F6		0.072		3840					
	G6		0.062		7680					
	H6		0.050		15360					
06	A7	6	0.517	0.517	120	4.094	491.225	524.7	5	21.7
	B7		0.315	0.315	240	1.664	399.254			
	C7		0.274	0.274	480	1.326	636.431			
	D7		0.141	0.141	960	0.497	477.164			
	E7		0.108	0.108	1920	0.348	667.938			
	F7		0.072		3840					
	G7		0.056		7680					
	H7		0.053		15360					
07	A8	7	0.052		120			N/A	0	-----
	B8		0.053		240					
	C8		0.047		480					
	D8		0.045		960					
	E8		0.041		1920					
	F8		0.042		3840					
	G8		0.044		7680					
	H8		0.044		15360					
08	A9	8	0.054		120			N/A	0	-----
	B9		0.046		240					
	C9		0.043		480					
	D9		0.040		960					

Samples (Contd)

Sample	Wells	ID	OD	OK OD	Dilution	Calc.Conc	Adjusted.Conc	GMC	N	CVdil
	E9		0.040		1920					
	F9		0.041		3840					
	G9		0.045		7680					
	H9		0.043		15360					
09	A10	9	0.050		120			N/A	0	-----
	B10		0.046		240					
	C10		0.049		480					
	D10		0.043		960					
	E10		0.047		1920					
	F10		0.048		3840					
	G10		0.042		7680					
	H10		0.045		15360					
10	A11	10	0.049		120			N/A	0	-----
	B11		0.045		240					
	C11		0.041		480					
	D11		0.050		960					
	E11		0.042		1920					
	F11		0.039		3840					
	G11		0.041		7680					
	H11		0.043		15360					
11	A12	11	0.041		120			N/A	0	-----
	B12		0.047		240					
	C12		0.047		480					
	D12		0.043		960					
	E12		0.047		1920					
	F12		0.050		3840					
	G12		0.042		7680					
	H12		0.051		15360					

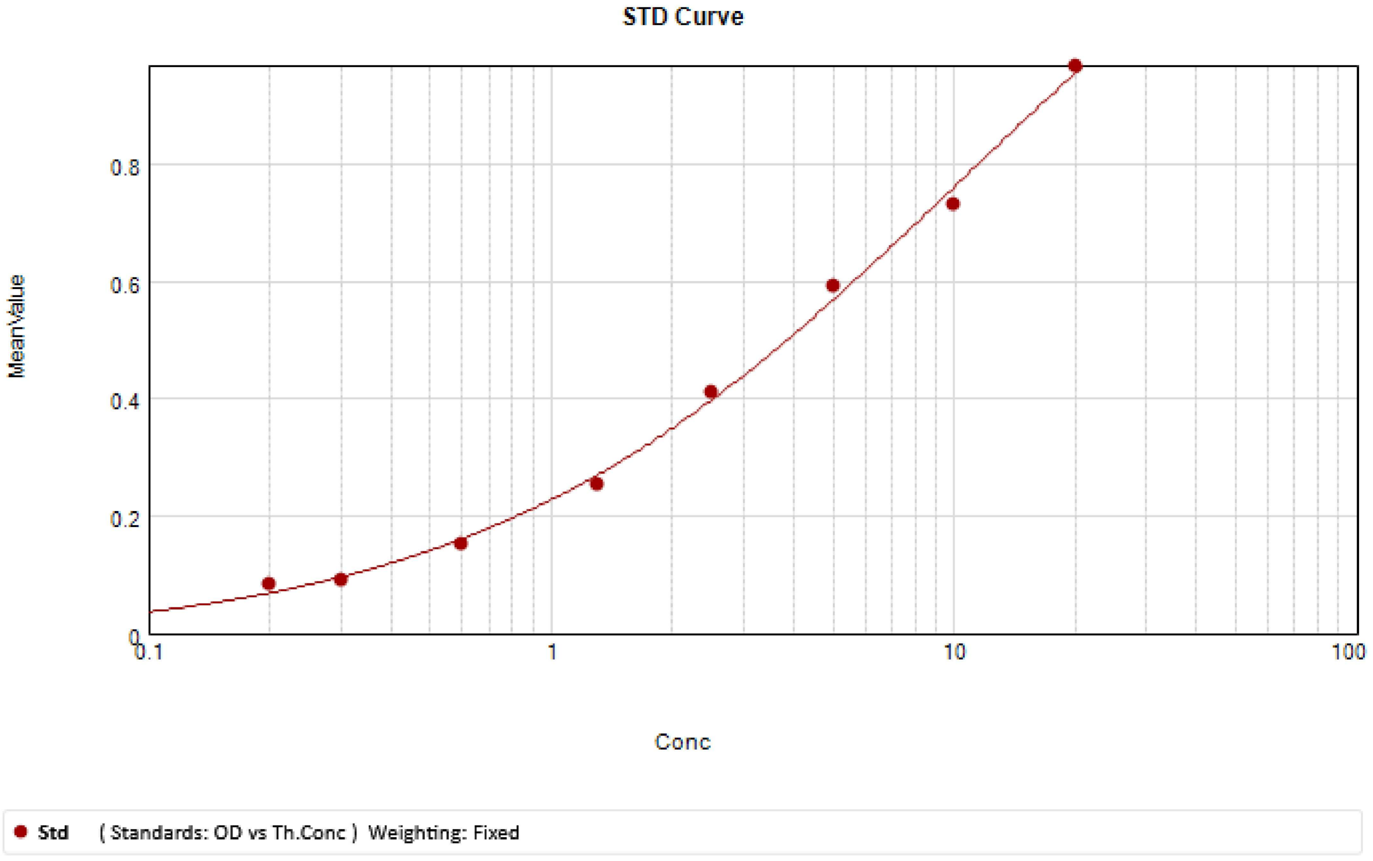

Curve Fit Results ▲

Curve Fit : 4-Parameter Logistic $y = D + \frac{A - D}{1 + \left(\frac{x}{C}\right)^B}$

	Parameter	Estimated Value	Std. Error	Confidence Interval
**Std** $R^2 = 0.997$ EC50 = 10.32	A	-0.016	0.067	[-0.203, 0.171]
	B	0.726	0.211	[0.140, 1.312]
	C	10.32	7.755	[-11.21, 31.85]
	D	1.560	0.463	[0.274, 2.845]

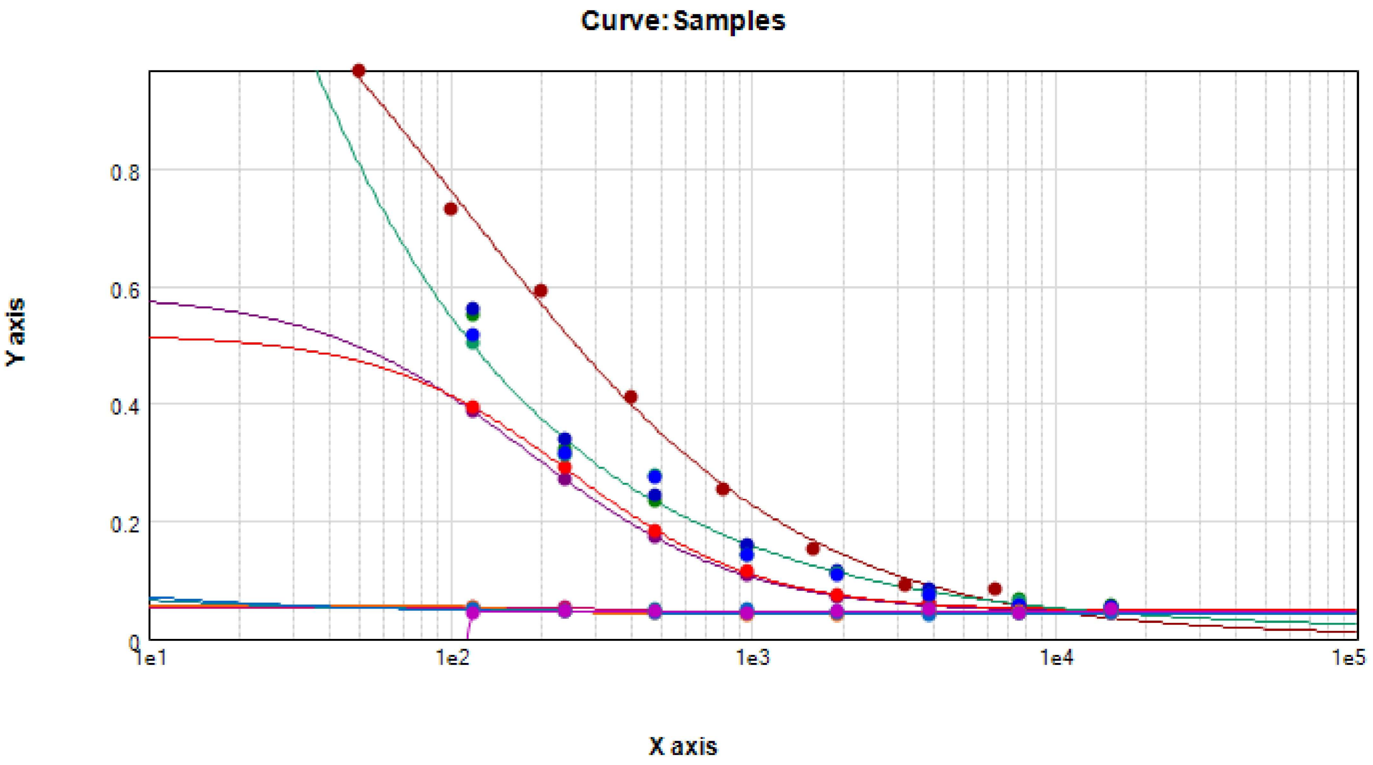

- **STD** (Standards: OD vs Dilution) Weighting: Fixed
- **S-1** (Samples: ODS1 vs DilSple1) Weighting: Fixed
- **S-2** (Samples: ODS2 vs DilSple2) Weighting: Fixed
- **S-3** (Samples: ODS3 vs DilSple3) Weighting: Fixed
- **S-4** (Samples: ODS4 vs DilSple4) Weighting: Fixed
- **S-5** (Samples: ODS5 vs DilSple5) Weighting: Fixed
- **S-6** (Samples: ODS6 vs DilSple6) Weighting: Fixed
- **S-7** (Samples: ODS7 vs DilSple7) Weighting: Fixed
- **S-8** (Samples: ODS8 vs DilSple8) Weighting: Fixed
- **S-9** (Samples: ODS9 vs DilSple9) Weighting: Fixed
- **S-10** (Samples: ODS10 vs DilSple10) Weighting: Fixed
- **S-11** (Samples: ODS11 vs DilSple11) Weighting: Fixed

Curve Fit Results ▼

Assay Parameter

Samples

Theoretical First Dilution Of Test Sample In Plate : 50.0 Sample dilution fold: 2.0

Nipha_Standard : NV-1

Concentration: 1000.0

Dilution (First dil in plate): 50.0

Dilution fold: 2.0

Others parameters

Rounding Decimal Standard Th.Conc: 1

Rounding Decimal RelErr% & CVdil: 1

Rounding Decimal GMC: 1

Average ODs of Blank: 0.046

SD of Blank: 0.004

Cutoff OD: 0.096
