# Supplementary material for: Development and Qualification of a Nipah Virus Glycoprotein-Specific IgG ELISA for the Assessment of Human Antibody Responses
Source: Vaccines (Basel). 2026 Jun 16;14(6):534. doi: 10.3390/vaccines14060534 (PMC13307770; doi:10.3390/vaccines14060534)
Supplement: Supplementary file 1 [file vaccines-14-00534-s001.zip › Supplementary_ELISA Qualification Data & Graph/5. Precision_Analysist-1/1. Precision_WHO IS_ANALYST-1_PLATE-1.pdf]

Intro

NIPAH_NIBSC_ANALYST#1_PLATE#1

OD

	1	2	3	4	5	6	7	8	9	10	11	12
A	0.969	0.556	0.591	0.380	0.402	0.508	0.497	0.058	0.055	0.046	0.050	0.041
B	0.734	0.347	0.388	0.255	0.262	0.338	0.345	0.049	0.056	0.044	0.047	0.047
C	0.594	0.234	0.251	0.160	0.160	0.227	0.230	0.044	0.048	0.044	0.046	0.047
D	0.402	0.156	0.150	0.100	0.102	0.142	0.140	0.044	0.052	0.044	0.049	0.043
E	0.258	0.101	0.105	0.074	0.069	0.096	0.096	0.047	0.049	0.046	0.046	0.047
F	0.157	0.074	0.070	0.052	0.057	0.067	0.069	0.046	0.044	0.048	0.042	0.050
G	0.092	0.060	0.059	0.053	0.051	0.052	0.058	0.041	0.046	0.043	0.045	0.042
H	0.076	0.052	0.054	0.047	0.049	0.046	0.047	0.041	0.045	0.048	0.043	0.051

Settings Information
Endpoint
Absorbance
Lm1 450
More Settings
Shake Off
Calibrate On
Carriage Speed Normal
Column Priority

Read Information
Imported Data : 4:00 PM 9/19/2024
Imported By : anjan

Reduction Settings

Optical Density
Wavelength Combination : !Lm1

Sample Dil

Main Sample Dilution 50.0

Sample 1: NV-2 120.0

Sample 2: NV-2 120.0

Sample 3: NV-10 50.0

Sample 4: NV-10 50.0

Sample 5: NV-4 120.0

Sample 6: NV-4 120.0

Sample 7: NC-1 60.0

Sample 8: NC-1 60.0

Sample 9: CNC 60.0

Sample 10: CNC 60.0

Sample 11: BLANK 50.0

Standards

Sample	Wells	OD	OK OD	Dilution	Calc.Conc	Adj.Conc	GMC	N	Th.Conc	RelErr%
01	A1	0.969	0.969	50	20.659	1033.0	990.6	6	20.000	3.300
	B1	0.734	0.734	100	9.067	906.7			10.000	-9.300
	C1	0.594	0.594	200	5.510	1102.0			5.000	10.200
	D1	0.402	0.402	400	2.548	1019.3			2.500	1.900
	E1	0.258	0.258	800	1.210	967.8			1.300	-6.900
	F1	0.157	0.157	1600	0.580	927.9			0.600	-3.300
	G1	0.092		3200					0.300	
	H1	0.076		6400					0.200	

Samples

Sample	Wells	ID	OD	OK OD	Dilution	Calc.Conc	Adjusted.Conc	GMC	N	CVdil
01	A2	1	0.556	0.556	120	4.782	573.876	540.6	5	10.9
	B2		0.347	0.347	240	1.967	472.041			
	C2		0.234	0.234	480	1.040	499.000			
	D2		0.156	0.156	960	0.575	551.797			
	E2		0.101	0.101	1920	0.323	619.259			
	F2		0.074		3840					
	G2		0.060		7680					
	H2		0.052		15360					
02	A3	2	0.591	0.591	120	5.449	653.924	589.1	5	9.9
	B3		0.388	0.388	240	2.391	573.850			
	C3		0.251	0.251	480	1.159	556.175			
	D3		0.150	0.150	960	0.544	522.547			
	E3		0.105	0.105	1920	0.339	650.575			
	F3		0.070		3840					
	G3		0.059		7680					
	H3		0.054		15360					
03	A4	3	0.380	0.380	120	2.304	276.497	288.1	4	4.2
	B4		0.255	0.255	240	1.188	285.050			
	C4		0.160	0.160	480	0.596	285.844			
	D4		0.100	0.100	960	0.319	305.763			
	E4		0.074		1920					
	F4		0.052		3840					
	G4		0.053		7680					
	H4		0.047		15360					
04	A5	4	0.402	0.402	120	2.548	305.781	300.5	4	4.0
	B5		0.262	0.262	240	1.239	297.453			
	C5		0.160	0.160	480	0.596	285.844			
	D5		0.102	0.102	960	0.327	313.515			
	E5		0.069		1920					
	F5		0.057		3840					
	G5		0.051		7680					
	H5		0.049		15360					
05	A6	5	0.508	0.508	120	3.974	476.890	492.1	5	9.7
	B6		0.338	0.338	240	1.881	451.322			
	C6		0.227	0.227	480	0.992	476.382			
	D6		0.142	0.142	960	0.505	484.634			
	E6		0.096	0.096	1920	0.303	580.976			
	F6		0.067		3840					
	G6		0.052		7680					
	H6		0.046		15360					
06	A7	6	0.497	0.497	120	3.805	456.544	491.4	5	9.7
	B7		0.345	0.345	240	1.947	467.388			
	C7		0.230	0.230	480	1.013	486.010			
	D7		0.140	0.140	960	0.495	475.350			
	E7		0.096	0.096	1920	0.303	580.976			
	F7		0.069		3840					
	G7		0.058		7680					
	H7		0.047		15360					
07	A8	7	0.058		120			N/A	0	-----
	B8		0.049		240					
	C8		0.044		480					
	D8		0.044		960					
	E8		0.047		1920					
	F8		0.046		3840					
	G8		0.041		7680					
	H8		0.041		15360					
08	A9	8	0.055		120			N/A	0	-----
	B9		0.056		240					
	C9		0.048		480					
	D9		0.052		960					

Samples (Contd)

Sample	Wells	ID	OD	OK OD	Dilution	Calc.Conc	Adjusted.Conc	GMC	N	CVdil
	E9		0.049		1920					
	F9		0.044		3840					
	G9		0.046		7680					
	H9		0.045		15360					
09	A10	9	0.046		120			N/A	0	-----
	B10		0.044		240					
	C10		0.044		480					
	D10		0.044		960					
	E10		0.046		1920					
	F10		0.048		3840					
	G10		0.043		7680					
	H10		0.048		15360					
10	A11	10	0.050		120			N/A	0	-----
	B11		0.047		240					
	C11		0.046		480					
	D11		0.049		960					
	E11		0.046		1920					
	F11		0.042		3840					
	G11		0.045		7680					
	H11		0.043		15360					
11	A12	11	0.041		120			N/A	0	-----
	B12		0.047		240					
	C12		0.047		480					
	D12		0.043		960					
	E12		0.047		1920					
	F12		0.050		3840					
	G12		0.042		7680					
	H12		0.051		15360					

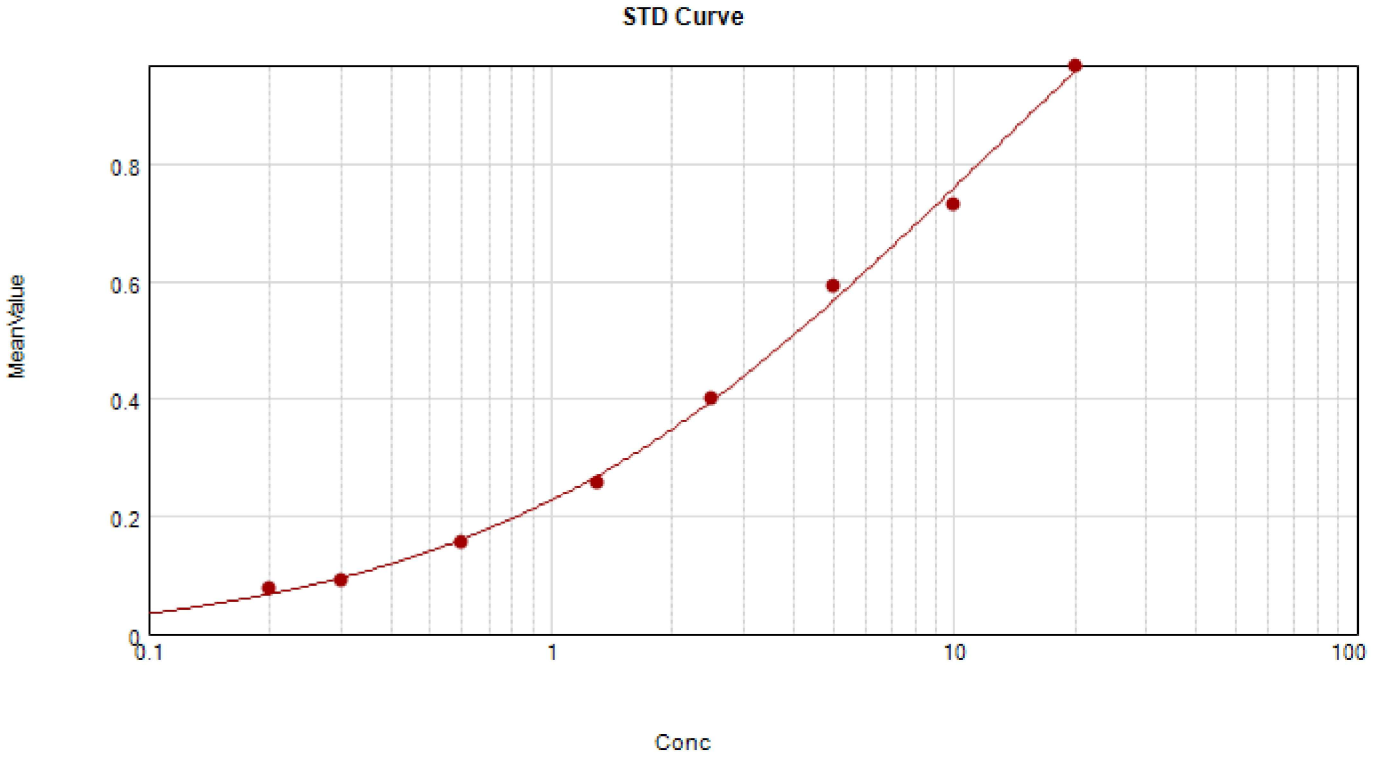

● **Std** (Standards: OD vs Th.Conc) Weighting: Fixed

Curve Fit Results ▲

Curve Fit : 4-Parameter Logistic $y = D + \frac{A - D}{1 + \left(\frac{x}{C}\right)^B}$

	Parameter	Estimated Value	Std. Error	Confidence Interval
**Std**<br>R^2 = 0.998<br>EC50 = 11.09	A	-0.020	0.062	[-0.193, 0.153]
	B	0.712	0.190	[0.184, 1.239]
	C	11.09	8.090	[-11.38, 33.55]
	D	1.604	0.456	[0.339, 2.869]

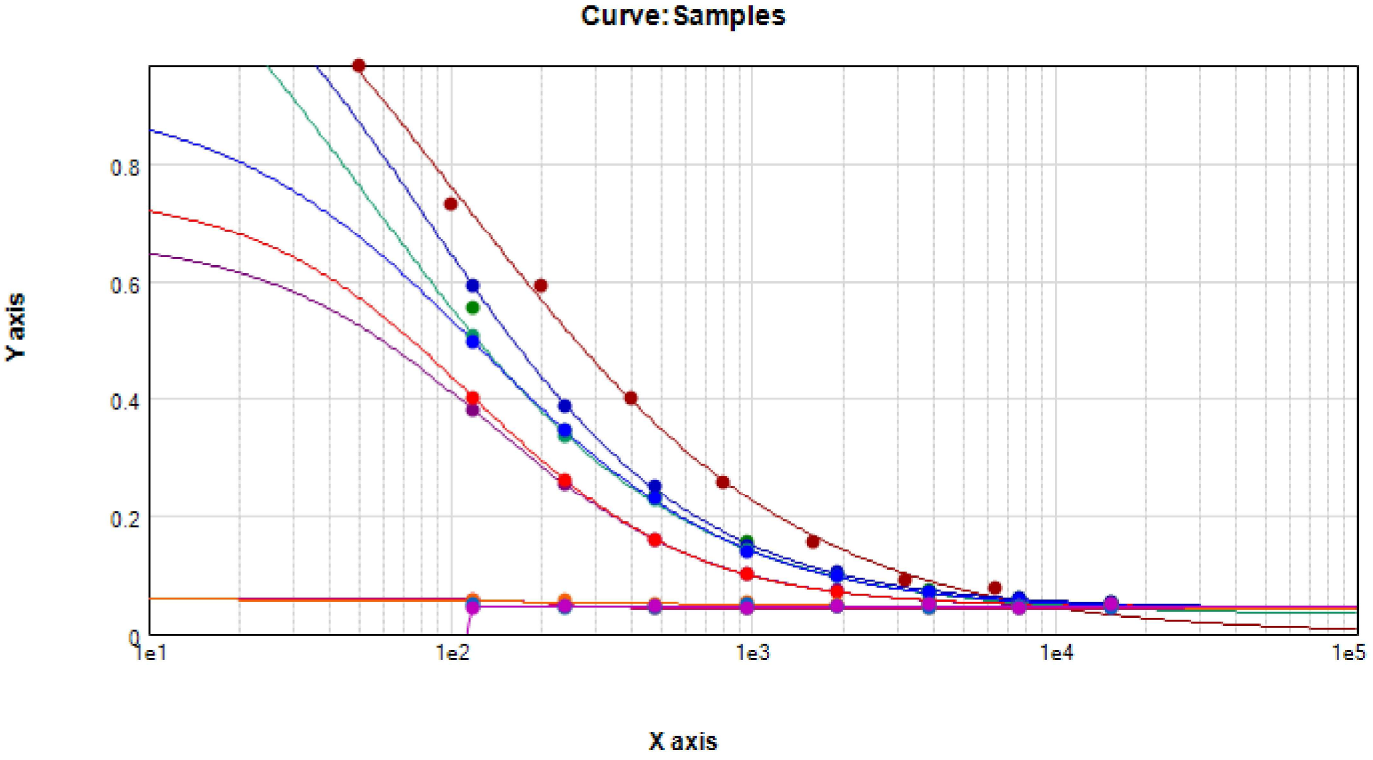

- **STD** (Standards: OD vs Dilution) Weighting: Fixed
- **S-1** (Samples: ODS1 vs DilSple1) Weighting: Fixed
- **S-2** (Samples: ODS2 vs DilSple2) Weighting: Fixed
- **S-3** (Samples: ODS3 vs DilSple3) Weighting: Fixed
- **S-4** (Samples: ODS4 vs DilSple4) Weighting: Fixed
- **S-5** (Samples: ODS5 vs DilSple5) Weighting: Fixed
- **S-6** (Samples: ODS6 vs DilSple6) Weighting: Fixed
- **S-7** (Samples: ODS7 vs DilSple7) Weighting: Fixed
- **S-8** (Samples: ODS8 vs DilSple8) Weighting: Fixed
- **S-9** (Samples: ODS9 vs DilSple9) Weighting: Fixed
- **S-10** (Samples: ODS10 vs DilSple10) Weighting: Fixed
- **S-11** (Samples: ODS11 vs DilSple11) Weighting: Fixed

Curve Fit Results ▼

Assay Parameter

Samples

Theoretical First Dilution Of Test Sample In Plate : 50.0 Sample dilution fold: 2.0

Nipha_Standard : NV-1

Concentration: 1000.0

Dilution (First dil in plate): 50.0

Dilution fold: 2.0

Others parameters

Rounding Decimal Standard Th.Conc: 1

Rounding Decimal RelErr% & CVdil: 1

Rounding Decimal GMC: 1

Average ODs of Blank: 0.046

SD of Blank: 0.004

Cutoff OD: 0.096
