# Supplementary material for: Development and Qualification of a Nipah Virus Glycoprotein-Specific IgG ELISA for the Assessment of Human Antibody Responses
Source: Vaccines (Basel). 2026 Jun 16;14(6):534. doi: 10.3390/vaccines14060534 (PMC13307770; doi:10.3390/vaccines14060534)
Supplement: Supplementary file 1 [file vaccines-14-00534-s001.zip › Supplementary_ELISA Qualification Data & Graph/5. Precision_Analysist-1/2. Precision_WHO IS_ANALYST-1_PLATE-2.pdf]

Intro

NIPAH_NIBSC_ANALYST#1_PLATE#2

OD

	1	2	3	4	5	6	7	8	9	10	11	12
A	0.981	0.558	0.582	0.379	0.403	0.506	0.498	0.053	0.051	0.043	0.049	0.047
B	0.753	0.361	0.382	0.258	0.264	0.336	0.339	0.047	0.050	0.044	0.040	0.045
C	0.597	0.236	0.249	0.150	0.160	0.224	0.223	0.050	0.046	0.045	0.044	0.045
D	0.404	0.153	0.147	0.098	0.104	0.138	0.142	0.041	0.040	0.041	0.048	0.048
E	0.255	0.100	0.102	0.073	0.069	0.097	0.095	0.042	0.039	0.041	0.044	0.044
F	0.152	0.075	0.073	0.053	0.059	0.066	0.067	0.043	0.043	0.046	0.042	0.047
G	0.093	0.059	0.056	0.042	0.046	0.055	0.055	0.046	0.040	0.046	0.044	0.048
H	0.067	0.052	0.045	0.043	0.042	0.048	0.043	0.042	0.041	0.041	0.043	0.046

Settings Information
Endpoint
Absorbance
Lm1 450
More Settings
Shake Off
Calibrate On
Carriage Speed Normal
Column Priority

Read Information
Imported Data : 4:04 PM 9/19/2024
Imported By : anjan

Reduction Settings

Optical Density
Wavelength Combination : !Lm1

Sample Dil

Main Sample Dilution 50.0

Sample 1: NV-2 120.0

Sample 2: NV-2 120.0

Sample 3: NV-10 50.0

Sample 4: NV-10 50.0

Sample 5: NV-4 120.0

Sample 6: NV-4 120.0

Sample 7: NC-1 60.0

Sample 8: NC-1 60.0

Sample 9: CNC 60.0

Sample 10: CNC 60.0

Sample 11: BLANK 50.0

Standards

Sample	Wells	OD	OK OD	Dilution	Calc.Conc	Adj.Conc	GMC	N	Th.Conc	RelErr%
01	A1	0.981	0.981	50	20.555	1027.8	990.3	6	20.000	2.800
	B1	0.753	0.753	100	9.249	924.9			10.000	-7.500
	C1	0.597	0.597	200	5.388	1077.7			5.000	7.800
	D1	0.404	0.404	400	2.551	1020.5			2.500	2.100
	E1	0.255	0.255	800	1.213	970.3			1.300	-6.700
	F1	0.152	0.152	1600	0.581	929.9			0.600	-3.100
	G1	0.093		3200					0.300	
	H1	0.067		6400					0.200	

Samples

Sample	Wells	ID	OD	OK OD	Dilution	Calc.Conc	Adjusted.Conc	GMC	N	CVdil
01	A2	1	0.558	0.558	120	4.681	561.689	557.5	5	9.9
	B2		0.361	0.361	240	2.105	505.213			
	C2		0.236	0.236	480	1.080	518.558			
	D2		0.153	0.153	960	0.586	562.866			
	E2		0.100	0.100	1920	0.339	650.368			
	F2		0.075		3840					
	G2		0.059		7680					
	H2		0.052		15360					
02	A3	2	0.582	0.582	120	5.107	612.785	584.2	5	9.0
	B3		0.382	0.382	240	2.316	555.901			
	C3		0.249	0.249	480	1.170	561.696			
	D3		0.147	0.147	960	0.556	533.473			
	E3		0.102	0.102	1920	0.347	666.510			
	F3		0.073		3840					
	G3		0.056		7680					
	H3		0.045		15360					
03	A4	3	0.379	0.379	120	2.285	274.237	289.9	4	7.0
	B4		0.258	0.258	240	1.235	296.286			
	C4		0.150	0.150	480	0.571	274.045			
	D4		0.098	0.098	960	0.330	317.183			
	E4		0.073		1920					
	F4		0.053		3840					
	G4		0.042		7680					
	H4		0.043		15360					
04	A5	4	0.403	0.403	120	2.540	304.843	312.6	4	6.0
	B5		0.264	0.264	240	1.278	306.812			
	C5		0.160	0.160	480	0.623	298.984			
	D5		0.104	0.104	960	0.356	341.397			
	E5		0.069		1920					
	F5		0.059		3840					
	G5		0.046		7680					
	H5		0.042		15360					
05	A6	5	0.506	0.506	120	3.855	462.554	498.0	5	13.3
	B6		0.336	0.336	240	1.869	448.676			
	C6		0.224	0.224	480	1.000	480.237			
	D6		0.138	0.138	960	0.511	490.578			
	E6		0.097	0.097	1920	0.326	626.420			
	F6		0.066		3840					
	G6		0.055		7680					
	H6		0.048		15360					
06	A7	6	0.498	0.498	120	3.738	448.582	497.0	5	12.6
	B7		0.339	0.339	240	1.897	455.250			
	C7		0.223	0.223	480	0.994	477.107			
	D7		0.142	0.142	960	0.531	509.466			
	E7		0.095	0.095	1920	0.318	610.632			
	F7		0.067		3840					
	G7		0.055		7680					
	H7		0.043		15360					
07	A8	7	0.053		120			N/A	0	-----
	B8		0.047		240					
	C8		0.050		480					
	D8		0.041		960					
	E8		0.042		1920					
	F8		0.043		3840					
	G8		0.046		7680					
	H8		0.042		15360					
08	A9	8	0.051		120			N/A	0	-----
	B9		0.050		240					
	C9		0.046		480					
	D9		0.040		960					

Samples (Contd)

Sample	Wells	ID	OD	OK OD	Dilution	Calc.Conc	Adjusted.Conc	GMC	N	CVdil
	E9		0.039		1920					
	F9		0.043		3840					
	G9		0.040		7680					
	H9		0.041		15360					
09	A10	9	0.043		120			N/A	0	-----
	B10		0.044		240					
	C10		0.045		480					
	D10		0.041		960					
	E10		0.041		1920					
	F10		0.046		3840					
	G10		0.046		7680					
	H10		0.041		15360					
10	A11	10	0.049		120			N/A	0	-----
	B11		0.040		240					
	C11		0.044		480					
	D11		0.048		960					
	E11		0.044		1920					
	F11		0.042		3840					
	G11		0.044		7680					
	H11		0.043		15360					
11	A12	11	0.047		120			N/A	0	-----
	B12		0.045		240					
	C12		0.045		480					
	D12		0.048		960					
	E12		0.044		1920					
	F12		0.047		3840					
	G12		0.048		7680					
	H12		0.046		15360					

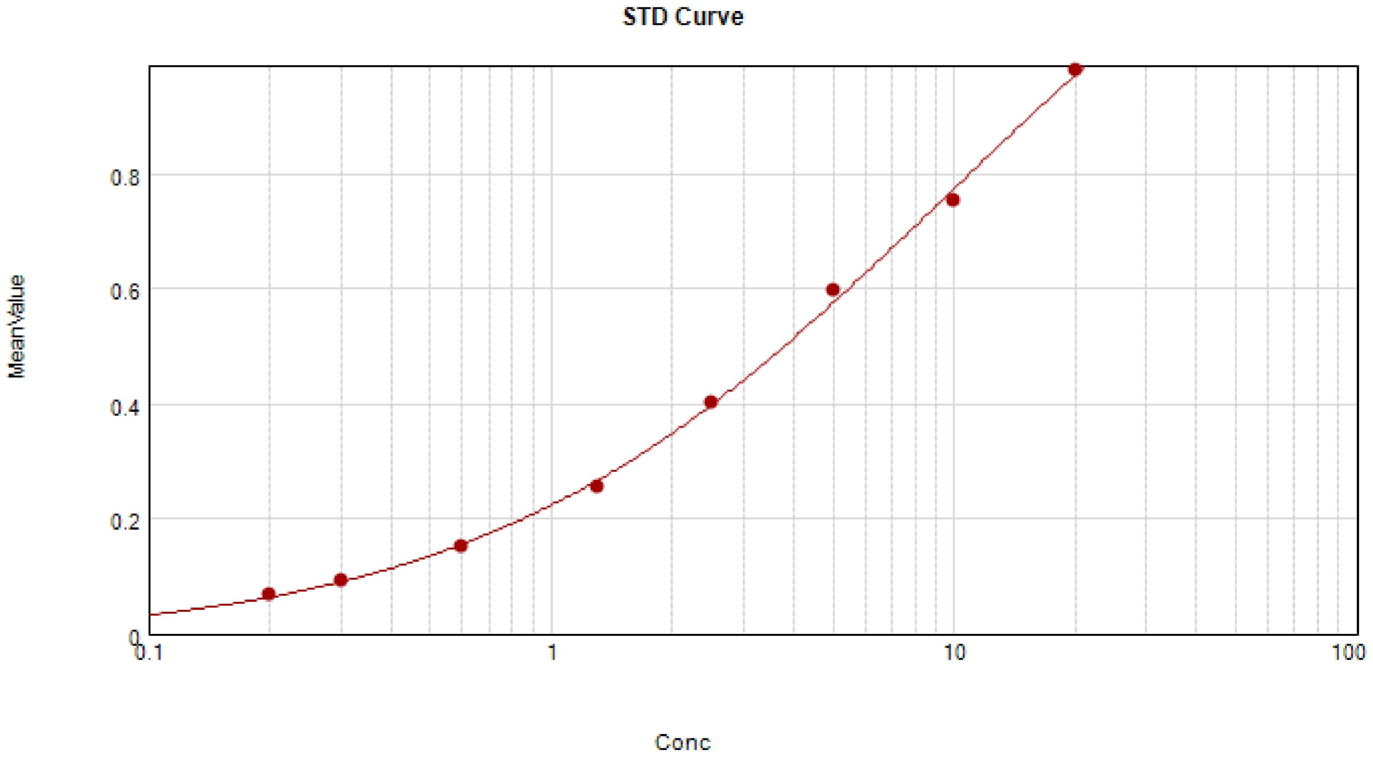

● Std (Standards: OD vs Th.Conc) Weighting: Fixed

Curve Fit Results ▲

Curve Fit : 4-Parameter Logistic $y = D + \frac{A - D}{1 + \left(\frac{x}{C}\right)^B}$

	Parameter	Estimated Value	Std. Error	Confidence Interval
**Std** $R^2 = 0.998$ EC50 = 9.484	A	-0.016	0.045	[-0.141, 0.109]
	B	0.756	0.147	[0.347, 1.165]
	C	9.484	4.417	[-2.781, 21.75]
	D	1.536	0.292	[0.726, 2.347]

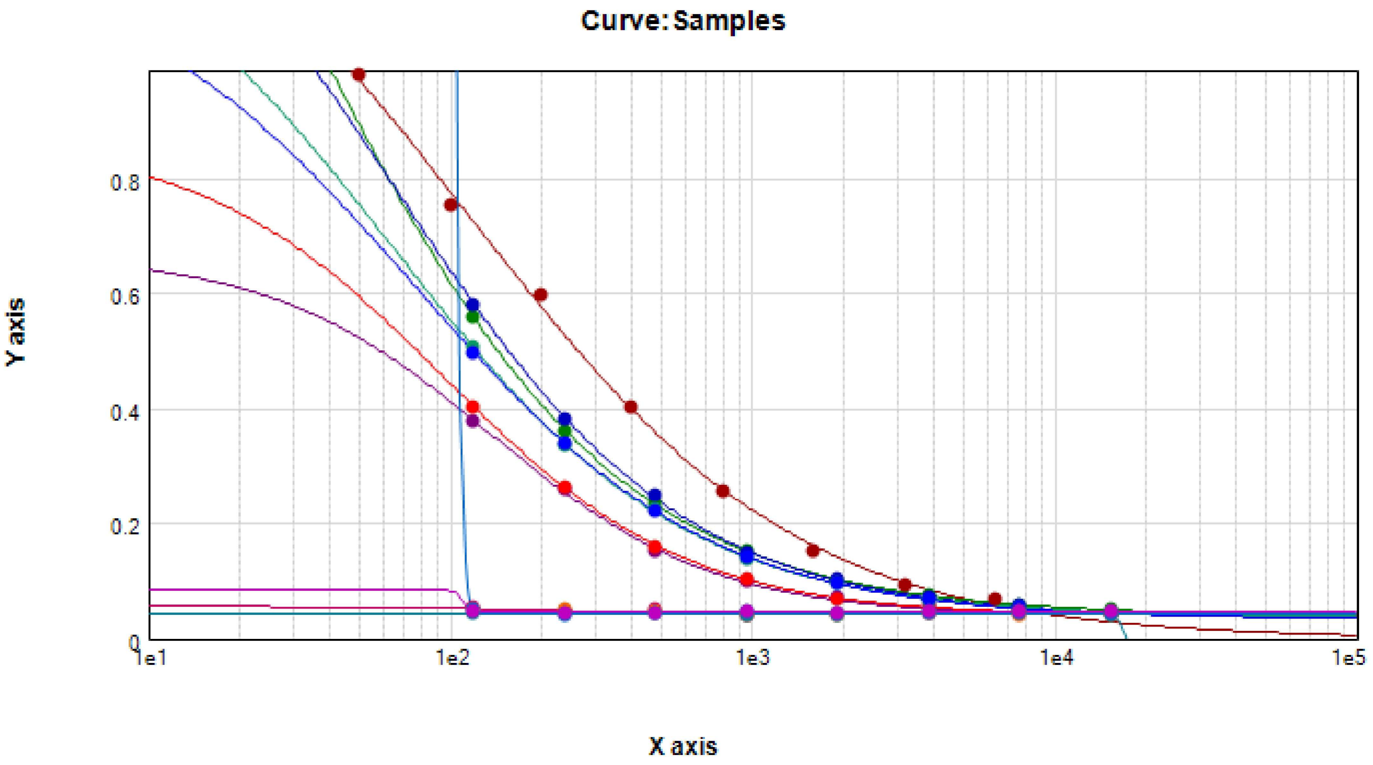

- **STD** (Standards: OD vs Dilution) Weighting: Fixed
- **S-1** (Samples: ODS1 vs DilSple1) Weighting: Fixed
- **S-2** (Samples: ODS2 vs DilSple2) Weighting: Fixed
- **S-3** (Samples: ODS3 vs DilSple3) Weighting: Fixed
- **S-4** (Samples: ODS4 vs DilSple4) Weighting: Fixed
- **S-5** (Samples: ODS5 vs DilSple5) Weighting: Fixed
- **S-6** (Samples: ODS6 vs DilSple6) Weighting: Fixed
- **S-7** (Samples: ODS7 vs DilSple7) Weighting: Fixed
- **S-8** (Samples: ODS8 vs DilSple8) Weighting: Fixed
- **S-9** (Samples: ODS9 vs DilSple9) Weighting: Fixed
- **S-10** (Samples: ODS10 vs DilSple10) Weighting: Fixed
- **S-11** (Samples: ODS11 vs DilSple11) Weighting: Fixed

Curve Fit Results ▼

Assay Parameter

Samples

Theoretical First Dilution Of Test Sample In Plate : 50.0   Sample dilution fold: 2.0

Nipha_Standard : NV-1

Concentration: 1000.0

Dilution (First dil in plate): 50.0

Dilution fold: 2.0

Others parameters

Rounding Decimal Standard Th.Conc: 1

Rounding Decimal RelErr% & CVdil: 1

Rounding Decimal GMC: 1

Average ODs of Blank: 0.046

SD of Blank: 0.001

Cutoff OD: 0.094
