# Supplementary material for: Development and Qualification of a Nipah Virus Glycoprotein-Specific IgG ELISA for the Assessment of Human Antibody Responses
Source: Vaccines (Basel). 2026 Jun 16;14(6):534. doi: 10.3390/vaccines14060534 (PMC13307770; doi:10.3390/vaccines14060534)
Supplement: Supplementary file 1 [file vaccines-14-00534-s001.zip › Supplementary_ELISA Qualification Data & Graph/3. Linearity_Analysist-1/1. Linearity_WHO IS_ANALYST-1_PLATE-1_DAY-1.pdf]

Intro

NIPAH_NIBSC_LINEARITY_ANALYST#1_PLATE#1_DAY#1

OD

	1	2	3	4	5	6	7	8	9	10	11	12
A	1.024	0.854	0.615	0.441	0.301	0.047	0.338	0.223	0.136	0.090	0.060	0.047
B	0.802	0.686	0.465	0.322	0.198	0.046	0.206	0.138	0.088	0.065	0.049	0.046
C	0.575	0.430	0.298	0.192	0.115	0.045	0.127	0.087	0.058	0.049	0.049	0.048
D	0.377	0.310	0.179	0.110	0.081	0.048	0.075	0.058	0.047	0.042	0.049	0.047
E	0.223	0.178	0.110	0.067	0.051	0.045	0.059	0.048	0.042	0.039	0.045	0.048
F	0.135	0.108	0.077	0.054	0.045	0.045	0.043	0.042	0.040	0.040	0.051	0.045
G	0.084	0.072	0.047	0.045	0.034	0.046	0.047	0.038	0.035	0.034	0.043	0.046
H	0.075	0.061	0.041	0.043	0.032	0.043	0.041	0.034	0.032	0.032	0.045	0.045

Settings Information
Endpoint
Absorbance
Lm1 450
More Settings
Shake Off
Calibrate On
Carriage Speed Normal
Column Priority

Read Information
Imported Data : 12:09 PM 10/2/2024
Imported By : anjan

Reduction Settings

Optical Density
Wavelength Combination : !Lm1

Sample Dil

Main Sample Dilution 40.0

Sample 1: NV-2 40.0

Sample 2: NV-2 (1:2) 40.0

Sample 3: NV-2 (1:4) 40.0

Sample 4: NV-2 (1:8) 40.0

Sample 5: BLANK 40.0

Sample 6: NV-6 40.0

Sample 7: NV-6 (1:2) 40.0

Sample 8: NV-6 (1:4) 40.0

Sample 9: NV-6 (1:8) 40.0

Sample 10: CNC 40.0

Sample 11: BLANK 40.0

Standards

Sample	Wells	OD	OK OD	Dilution	Calc.Conc	Adj.Conc	GMC	N	Th.Conc	RelErr%
01	A1	1.024	1.024	40	25.245	1009.8	999.5	6	25.000	1.000
	B1	0.802	0.802	80	12.288	983.1			12.500	-1.700
	C1	0.575	0.575	160	6.298	1007.6			6.300	-0.000
	D1	0.377	0.377	320	3.233	1034.7			3.100	4.300
	E1	0.223	0.223	640	1.548	990.7			1.600	-3.300
	F1	0.135	0.135	1280	0.760	972.4			0.800	-5.000
	G1	0.084		2560					0.400	
	H1	0.075		5120					0.200	

Samples

Sample	Wells	ID	OD	OK OD	Dilution	Calc.Conc	Adjusted.Conc	GMC	N	CVdil
01	A2	1	0.854	0.854	40	14.365	574.617	679.7	6	10.9
	B2		0.686	0.686	80	8.751	700.071			
	C2		0.430	0.430	160	3.933	629.263			
	D2		0.310	0.310	320	2.444	782.032			
	E2		0.178	0.178	640	1.132	724.435			
	F2		0.108	0.108	1280	0.537	687.356			
	G2		0.072		2560					
	H2		0.061		5120					
02	A3	2	0.615	0.615	40	7.102	284.080	344.1	5	10.9
	B3		0.465	0.465	80	4.437	354.995			
	C3		0.298	0.298	160	2.312	369.961			
	D3		0.179	0.179	320	1.141	365.078			
	E3		0.110	0.110	640	0.553	354.063			
	F3		0.077		1280					
	G3		0.047		2560					
	H3		0.041		5120					
03	A4	3	0.441	0.441	40	4.088	163.503	186.2	4	11.0
	B4		0.322	0.322	80	2.578	206.261			
	C4		0.192	0.192	160	1.258	201.328			
	D4		0.110	0.110	320	0.553	177.031			
	E4		0.067		640					
	F4		0.054		1280					
	G4		0.045		2560					
	H4		0.043		5120					
04	A5	4	0.301	0.301	40	2.345	93.796	97.8	3	6.2
	B5		0.198	0.198	80	1.313	105.063			
	C5		0.115	0.115	160	0.594	95.035			
	D5		0.081		320					
	E5		0.051		640					
	F5		0.045		1280					
	G5		0.034		2560					
	H5		0.032		5120					
05	A6	5	0.047		40			N/A	0	-----
	B6		0.046		80					
	C6		0.045		160					
	D6		0.048		320					
	E6		0.045		640					
	F6		0.045		1280					
	G6		0.046		2560					
	H6		0.043		5120					
06	A7	6	0.338	0.338	40	2.762	110.482	110.8	3	0.2
	B7		0.206	0.206	80	1.387	110.992			
	C7		0.127	0.127	160	0.693	110.856			
	D7		0.075		320					
	E7		0.059		640					
	F7		0.043		1280					
	G7		0.047		2560					
	H7		0.041		5120					
07	A8	7	0.223	0.223	40	1.548	61.920	62.4	2	1.0
	B8		0.138	0.138	80	0.785	62.792			
	C8		0.087		160					
	D8		0.058		320					
	E8		0.048		640					
	F8		0.042		1280					
	G8		0.038		2560					
	H8		0.034		5120					
08	A9	8	0.136	0.136	40	0.768	30.722	30.7	1	-----
	B9		0.088		80					
	C9		0.058		160					
	D9		0.047		320					

Samples (Contd)

Sample	Wells	ID	OD	OK OD	Dilution	Calc.Conc	Adjusted.Conc	GMC	N	CVdil
	E9		0.042		640					
	F9		0.040		1280					
	G9		0.035		2560					
	H9		0.032		5120					
09	A10	9	0.090		40			N/A	0	-----
	B10		0.065		80					
	C10		0.049		160					
	D10		0.042		320					
	E10		0.039		640					
	F10		0.040		1280					
	G10		0.034		2560					
	H10		0.032		5120					
10	A11	10	0.060		40			N/A	0	-----
	B11		0.049		80					
	C11		0.049		160					
	D11		0.049		320					
	E11		0.045		640					
	F11		0.051		1280					
	G11		0.043		2560					
	H11		0.045		5120					
11	A12	11	0.047		40			N/A	0	-----
	B12		0.046		80					
	C12		0.048		160					
	D12		0.047		320					
	E12		0.048		640					
	F12		0.045		1280					
	G12		0.046		2560					
	H12		0.045		5120					

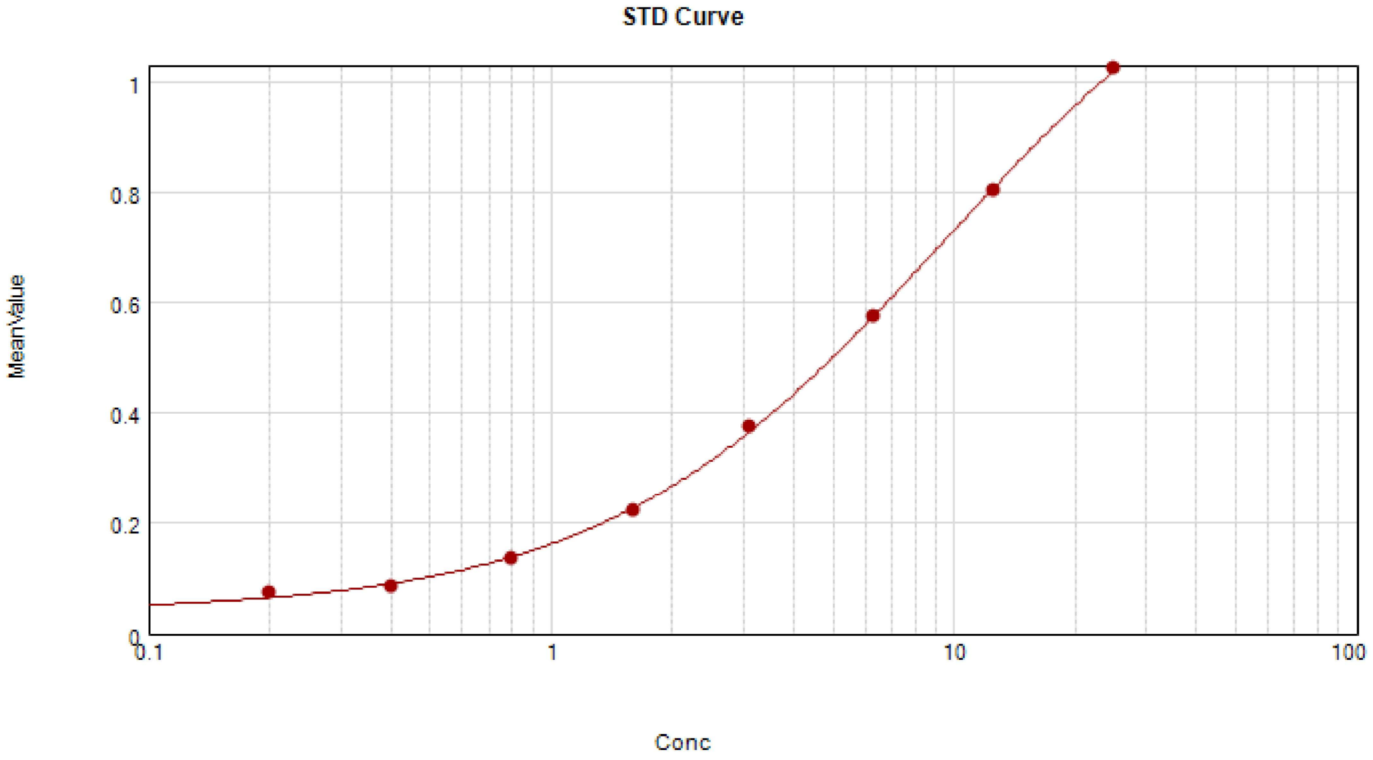

● **Std** (Standards: OD vs Th.Conc) Weighting: Fixed

Curve Fit Results ▲

Curve Fit : 4-Parameter Logistic $y = D + \frac{A - D}{1 + \left(\frac{x}{C}\right)^B}$

	Parameter	Estimated Value	Std. Error	Confidence Interval
**Std** R^2 = 1.000 EC50 = 9.449	A	0.040	0.012	[0.007, 0.072]
	B	1.021	0.068	[0.833, 1.208]
	C	9.449	1.051	[6.530, 12.37]
	D	1.385	0.073	[1.182, 1.588]

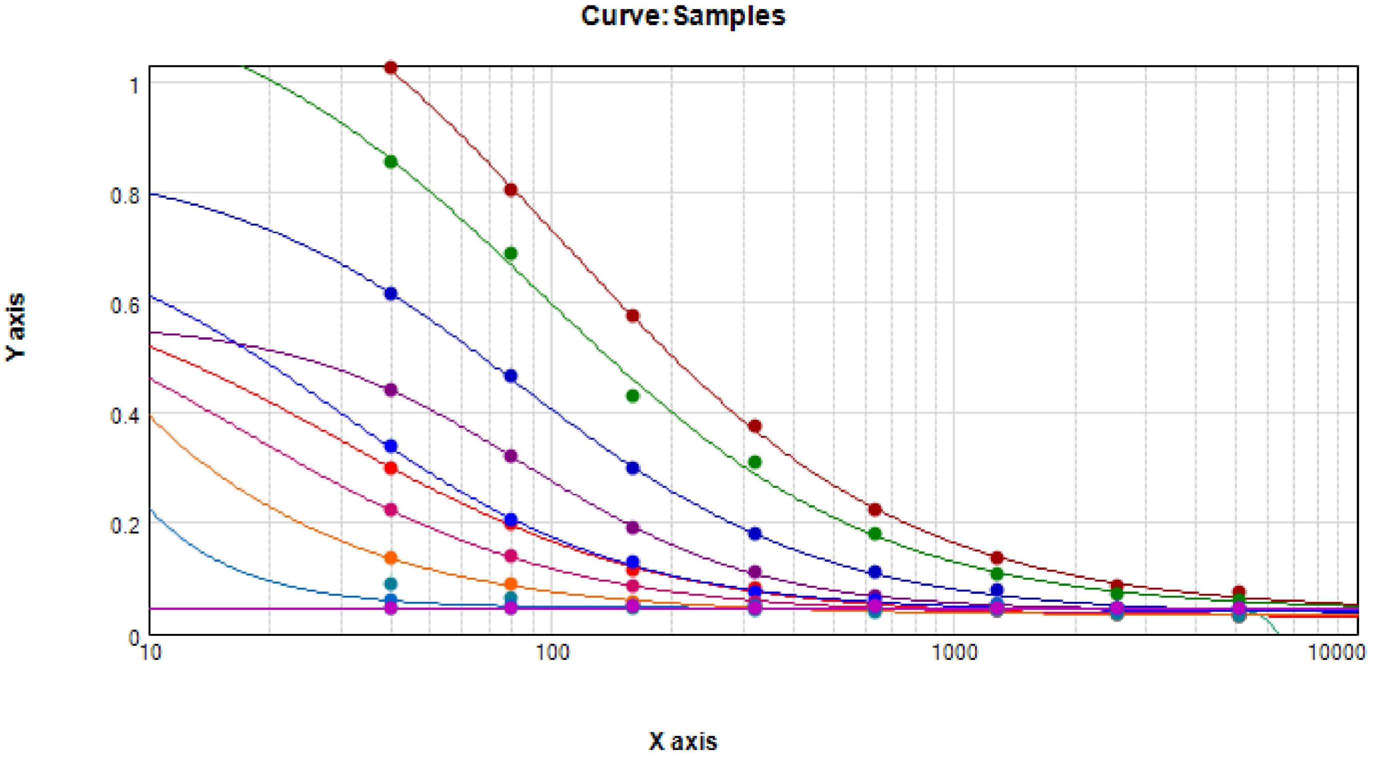

- **STD** (Standards: OD vs Dilution) Weighting: Fixed
- **S-1** (Samples: ODS1 vs DilSple1) Weighting: Fixed
- **S-2** (Samples: ODS2 vs DilSple2) Weighting: Fixed
- **S-3** (Samples: ODS3 vs DilSple3) Weighting: Fixed
- **S-4** (Samples: ODS4 vs DilSple4) Weighting: Fixed
- **S-5** (Samples: ODS5 vs DilSple5) Weighting: Fixed
- **S-6** (Samples: ODS6 vs DilSple6) Weighting: Fixed
- **S-7** (Samples: ODS7 vs DilSple7) Weighting: Fixed
- **S-8** (Samples: ODS8 vs DilSple8) Weighting: Fixed
- **S-9** (Samples: ODS9 vs DilSple9) Weighting: Fixed
- **S-10** (Samples: ODS10 vs DilSple10) Weighting: Fixed
- **S-11** (Samples: ODS11 vs DilSple11) Weighting: Fixed

Curve Fit Results ▼

Assay Parameter

Samples

Theoretical First Dilution Of Test Sample In Plate : 40.0   Sample dilution fold: 2.0

Nipha_Standard : NV-1

Concentration: 1000.0

Dilution (First dil in plate): 40.0

Dilution fold: 2.0

Others parameters

Rounding Decimal Standard Th.Conc: 1

Rounding Decimal RelErr% & CVdil: 1

Rounding Decimal GMC: 1

Average ODs of Blank: 0.046

SD of Blank: 0.001

Cutoff OD: 0.094
