# Supplementary material for: Development and Qualification of a Nipah Virus Glycoprotein-Specific IgG ELISA for the Assessment of Human Antibody Responses
Source: Vaccines (Basel). 2026 Jun 16;14(6):534. doi: 10.3390/vaccines14060534 (PMC13307770; doi:10.3390/vaccines14060534)
Supplement: Supplementary file 1 [file vaccines-14-00534-s001.zip › Supplementary_ELISA Qualification Data & Graph/3. Linearity_Analysist-1/2. Linearity_WHO IS_ANALYST-1_PLATE-1_DAY-2.pdf]

Intro

NIPAH_NIBSC_LINEARITY_ANALYST#1_PLATE#1_DAY#2

OD

	1	2	3	4	5	6	7	8	9	10	11	12
A	1.040	0.888	0.663	0.462	0.322	0.045	0.355	0.234	0.144	0.096	0.046	0.048
B	0.822	0.688	0.474	0.329	0.208	0.044	0.220	0.138	0.094	0.067	0.045	0.049
C	0.591	0.447	0.309	0.199	0.125	0.043	0.135	0.095	0.070	0.047	0.045	0.042
D	0.386	0.318	0.183	0.115	0.081	0.045	0.085	0.066	0.052	0.045	0.039	0.049
E	0.228	0.181	0.112	0.074	0.053	0.040	0.059	0.049	0.044	0.047	0.046	0.049
F	0.142	0.111	0.076	0.051	0.042	0.046	0.047	0.047	0.044	0.034	0.040	0.045
G	0.092	0.076	0.059	0.047	0.048	0.047	0.041	0.034	0.038	0.041	0.041	0.049
H	0.087	0.062	0.045	0.042	0.041	0.042	0.037	0.032	0.035	0.040	0.042	0.048

Settings Information
Endpoint
Absorbance
Lm1 450
More Settings
Shake Off
Calibrate On
Carriage Speed Normal
Column Priority

Read Information
Imported Data : 4:39 PM 10/2/2024
Imported By : anjan

Reduction Settings

Optical Density
Wavelength Combination : !Lm1

Sample Dil

Main Sample Dilution 40.0

Sample 1: NV-2 40.0

Sample 2: NV-2 (1:2) 40.0

Sample 3: NV-2 (1:4) 40.0

Sample 4: NV-2 (1:8) 40.0

Sample 5: BLANK 40.0

Sample 6: NV-6 40.0

Sample 7: NV-6 (1:2) 40.0

Sample 8: NV-6 (1:4) 40.0

Sample 9: NV-6 (1:8) 40.0

Sample 10: CNC 40.0

Sample 11: BLANK 40.0

Standards

Sample	Wells	OD	OK OD	Dilution	Calc.Conc	Adj.Conc	GMC	N	Th.Conc	RelErr%
01	A1	1.040	1.040	40	25.276	1011.0	999.3	6	25.000	1.100
	B1	0.822	0.822	80	12.278	982.2			12.500	-1.800
	C1	0.591	0.591	160	6.301	1008.2			6.300	0.000
	D1	0.386	0.386	320	3.232	1034.3			3.100	4.300
	E1	0.228	0.228	640	1.540	985.8			1.600	-3.700
	F1	0.142	0.142	1280	0.762	975.3			0.800	-4.800
	G1	0.092		2560					0.400	
	H1	0.087		5120					0.200	

Samples

Sample	Wells	ID	OD	OK OD	Dilution	Calc.Conc	Adjusted.Conc	GMC	N	CVdil
01	A2	1	0.888	0.888	40	14.990	599.615	670.2	6	9.5
	B2		0.688	0.688	80	8.341	667.260			
	C2		0.447	0.447	160	4.016	642.560			
	D2		0.318	0.318	320	2.453	784.818			
	E2		0.181	0.181	640	1.106	707.857			
	F2		0.111	0.111	1280	0.496	634.453			
	G2		0.076		2560					
	H2		0.062		5120					
02	A3	2	0.663	0.663	40	7.765	310.586	343.4	5	8.0
	B3		0.474	0.474	80	4.393	351.457			
	C3		0.309	0.309	160	2.356	376.919			
	D3		0.183	0.183	320	1.124	359.690			
	E3		0.112	0.112	640	0.504	322.685			
	F3		0.076		1280					
	G3		0.059		2560					
	H3		0.045		5120					
03	A4	3	0.462	0.462	40	4.223	168.923	186.0	4	11.0
	B4		0.329	0.329	80	2.573	205.823			
	C4		0.199	0.199	160	1.270	203.123			
	D4		0.115	0.115	320	0.530	169.535			
	E4		0.074		640					
	F4		0.051		1280					
	G4		0.047		2560					
	H4		0.042		5120					
04	A5	4	0.322	0.322	40	2.496	99.841	102.1	3	5.1
	B5		0.208	0.208	80	1.353	108.203			
	C5		0.125	0.125	160	0.615	98.457			
	D5		0.081		320					
	E5		0.053		640					
	F5		0.042		1280					
	G5		0.048		2560					
	H5		0.041		5120					
05	A6	5	0.045		40			N/A	0	-----
	B6		0.044		80					
	C6		0.043		160					
	D6		0.045		320					
	E6		0.040		640					
	F6		0.046		1280					
	G6		0.047		2560					
	H6		0.042		5120					
06	A7	6	0.355	0.355	40	2.866	114.627	114.7	3	2.2
	B7		0.220	0.220	80	1.465	117.173			
	C7		0.135	0.135	160	0.701	112.220			
	D7		0.085		320					
	E7		0.059		640					
	F7		0.047		1280					
	G7		0.041		2560					
	H7		0.037		5120					
07	A8	7	0.234	0.234	40	1.598	63.906	61.0	2	6.6
	B8		0.138	0.138	80	0.727	58.183			
	C8		0.095		160					
	D8		0.066		320					
	E8		0.049		640					
	F8		0.047		1280					
	G8		0.034		2560					
	H8		0.032		5120					
08	A9	8	0.144	0.144	40	0.779	31.171	31.2	1	-----
	B9		0.094		80					
	C9		0.070		160					
	D9		0.052		320					

Samples (Contd)

Sample	Wells	ID	OD	OK OD	Dilution	Calc.Conc	Adjusted.Conc	GMC	N	CVdil
	E9		0.044		640					
	F9		0.044		1280					
	G9		0.038		2560					
	H9		0.035		5120					
09	A10	9	0.096		40			N/A	0	-----
	B10		0.067		80					
	C10		0.047		160					
	D10		0.045		320					
	E10		0.047		640					
	F10		0.034		1280					
	G10		0.041		2560					
	H10		0.040		5120					
10	A11	10	0.046		40			N/A	0	-----
	B11		0.045		80					
	C11		0.045		160					
	D11		0.039		320					
	E11		0.046		640					
	F11		0.040		1280					
	G11		0.041		2560					
	H11		0.042		5120					
11	A12	11	0.048		40			N/A	0	-----
	B12		0.049		80					
	C12		0.042		160					
	D12		0.049		320					
	E12		0.049		640					
	F12		0.045		1280					
	G12		0.049		2560					
	H12		0.048		5120					

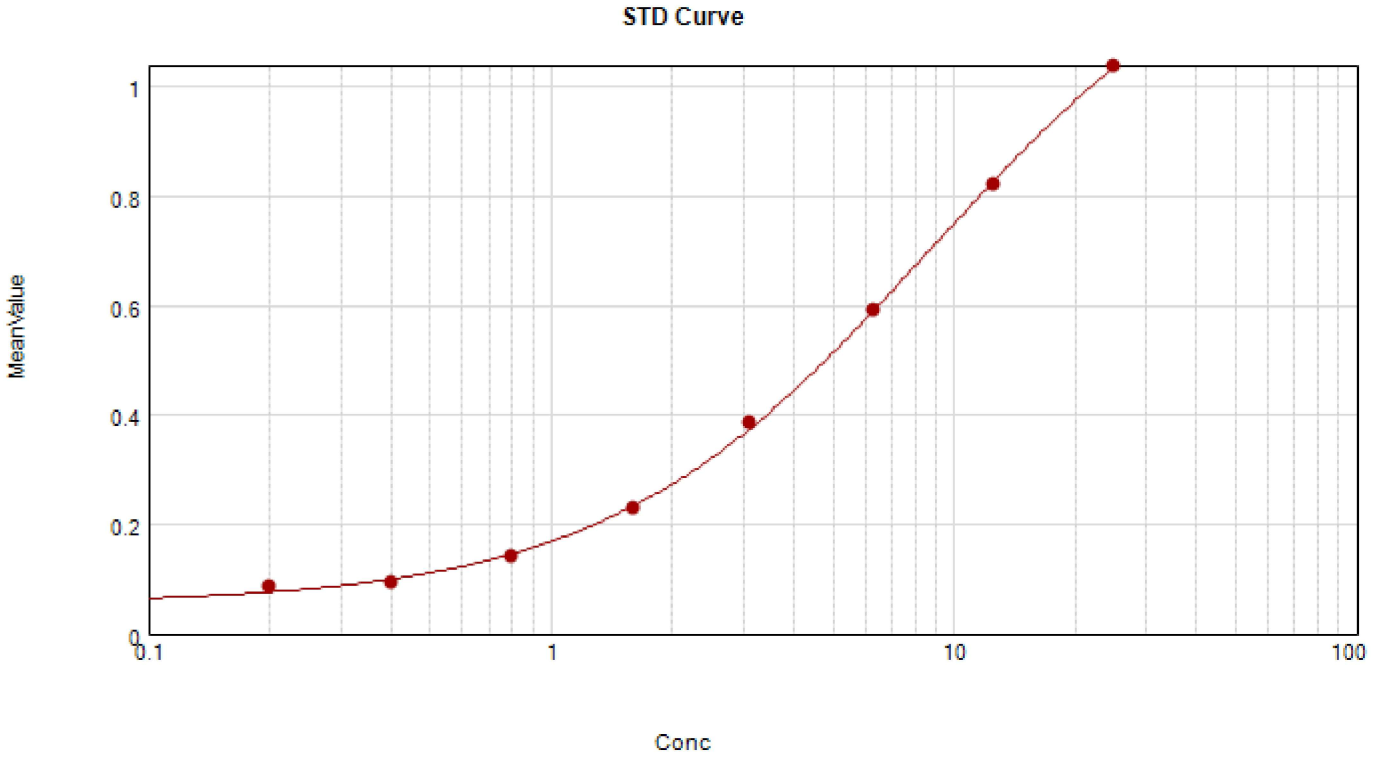

● Std (Standards: OD vs Th.Conc) Weighting: Fixed

Curve Fit Results ▲

Curve Fit : 4-Parameter Logistic $y = D + \frac{A - D}{1 + \left(\frac{x}{C}\right)^B}$

	Parameter	Estimated Value	Std. Error	Confidence Interval
**Std**<br>R^2 = 1.000<br>EC50 = 8.727	A	0.055	0.012	[0.022, 0.087]
	B	1.078	0.071	[0.880, 1.276]
	C	8.727	0.870	[6.310, 11.14]
	D	1.353	0.065	[1.172, 1.534]

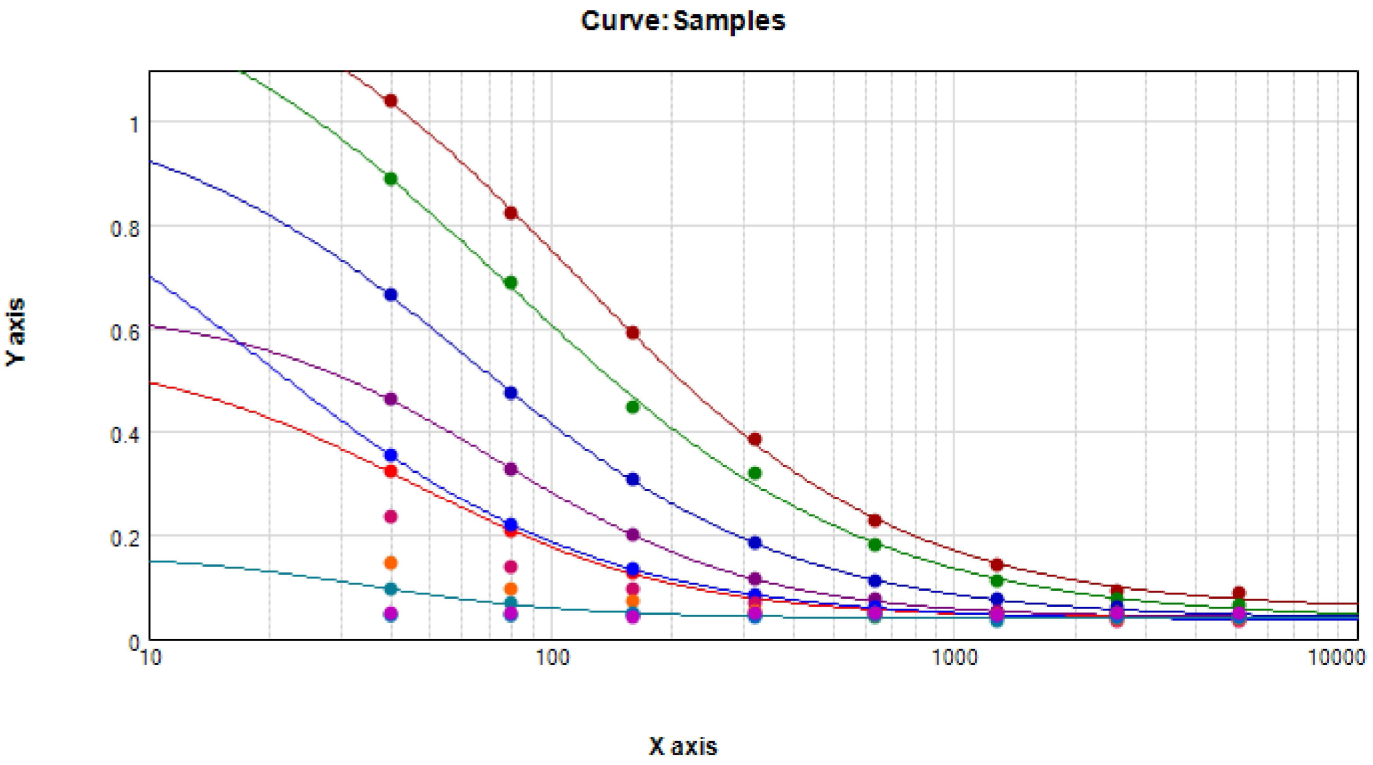

- **STD** (Standards: OD vs Dilution) Weighting: Fixed
- **S-1** (Samples: ODS1 vs DilSple1) Weighting: Fixed
- **S-2** (Samples: ODS2 vs DilSple2) Weighting: Fixed
- **S-3** (Samples: ODS3 vs DilSple3) Weighting: Fixed
- **S-4** (Samples: ODS4 vs DilSple4) Weighting: Fixed
- **S-5** (Samples: ODS5 vs DilSple5) Weighting: Fixed
- **S-6** (Samples: ODS6 vs DilSple6) Weighting: Fixed
- **S-7** (Samples: ODS7 vs DilSple7) Weighting: Fixed
- **S-8** (Samples: ODS8 vs DilSple8) Weighting: Fixed
- **S-9** (Samples: ODS9 vs DilSple9) Weighting: Fixed
- **S-10** (Samples: ODS10 vs DilSple10) Weighting: Fixed
- **S-11** (Samples: ODS11 vs DilSple11) Weighting: Fixed

Curve Fit Results ▼

Assay Parameter

Samples

Theoretical First Dilution Of Test Sample In Plate : 40.0   Sample dilution fold: 2.0

Nipha_Standard : NV-1

Concentration: 1000.0

Dilution (First dil in plate): 40.0

Dilution fold: 2.0

Others parameters

Rounding Decimal Standard Th.Conc: 1

Rounding Decimal RelErr% & CVdil: 1

Rounding Decimal GMC: 1

Average ODs of Blank: 0.047

SD of Blank: 0.003

Cutoff OD: 0.097
