# Supplementary material for: Development and Qualification of a Nipah Virus Glycoprotein-Specific IgG ELISA for the Assessment of Human Antibody Responses
Source: Vaccines (Basel). 2026 Jun 16;14(6):534. doi: 10.3390/vaccines14060534 (PMC13307770; doi:10.3390/vaccines14060534)
Supplement: Supplementary file 1 [file vaccines-14-00534-s001.zip › Supplementary_ELISA Qualification Data & Graph/3. Linearity_Analysist-1/3. Linearity_WHO IS_ANALYST-1_PLATE-1_DAY-3.pdf]

Intro

NIPAH_NIBSC_LINEARITY_ANALYST#1_PLATE#1_DAY#3

OD

	1	2	3	4	5	6	7	8	9	10	11	12
A	1.042	0.889	0.659	0.468	0.318	0.045	0.358	0.240	0.151	0.093	0.049	0.043
B	0.832	0.696	0.485	0.340	0.209	0.040	0.225	0.144	0.103	0.073	0.049	0.052
C	0.598	0.451	0.314	0.207	0.125	0.048	0.141	0.096	0.075	0.063	0.049	0.051
D	0.386	0.317	0.188	0.125	0.089	0.049	0.088	0.073	0.055	0.052	0.045	0.046
E	0.230	0.191	0.122	0.083	0.064	0.047	0.069	0.058	0.047	0.050	0.051	0.043
F	0.148	0.116	0.086	0.063	0.050	0.047	0.049	0.054	0.051	0.044	0.043	0.046
G	0.091	0.086	0.069	0.054	0.045	0.042	0.055	0.047	0.046	0.047	0.045	0.042
H	0.087	0.076	0.051	0.045	0.042	0.043	0.041	0.038	0.041	0.041	0.040	0.047

Settings Information
Endpoint
Absorbance
Lm1 450
More Settings
Shake Off
Calibrate On
Carriage Speed Normal
Column Priority

Read Information
Imported Data : 4:45 PM 10/2/2024
Imported By : anjan

Reduction Settings

Optical Density
Wavelength Combination : !Lm1

Sample Dil

Main Sample Dilution 40.0

Sample 1: NV-2 40.0

Sample 2: NV-2 (1:2) 40.0

Sample 3: NV-2 (1:4) 40.0

Sample 4: NV-2 (1:8) 40.0

Sample 5: BLANK 40.0

Sample 6: NV-6 40.0

Sample 7: NV-6 (1:2) 40.0

Sample 8: NV-6 (1:4) 40.0

Sample 9: NV-6 (1:8) 40.0

Sample 10: CNC 40.0

Sample 11: BLANK 40.0

Standards

Sample	Wells	OD	OK OD	Dilution	Calc.Conc	Adj.Conc	GMC	N	Th.Conc	RelErr%
01	A1	1.042	1.042	40	25.186	1007.5	1008.1	6	25.000	0.700
	B1	0.832	0.832	80	12.358	988.6			12.500	-1.100
	C1	0.598	0.598	160	6.298	1007.6			6.300	-0.000
	D1	0.386	0.386	320	3.187	1019.7			3.100	2.800
	E1	0.230	0.230	640	1.548	990.9			1.600	-3.200
	F1	0.148	0.148	1280	0.809	1035.2			0.800	1.100
	G1	0.091		2560					0.400	
	H1	0.087		5120					0.200	

Samples

Sample	Wells	ID	OD	OK OD	Dilution	Calc.Conc	Adjusted.Conc	GMC	N	CVdil
01	A2	1	0.889	0.889	40	14.710	588.383	682.0	6	10.4
	B2		0.696	0.696	80	8.340	667.221			
	C2		0.451	0.451	160	4.003	640.501			
	D2		0.317	0.317	320	2.415	772.698			
	E2		0.191	0.191	640	1.189	761.180			
	F2		0.116	0.116	1280	0.532	680.564			
	G2		0.086		2560					
	H2		0.076		5120					
02	A3	2	0.659	0.659	40	7.506	300.246	355.6	5	9.8
	B3		0.485	0.485	80	4.472	357.760			
	C3		0.314	0.314	160	2.383	381.279			
	D3		0.188	0.188	320	1.162	371.948			
	E3		0.122	0.122	640	0.583	373.420			
	F3		0.086		1280					
	G3		0.069		2560					
	H3		0.051		5120					
03	A4	3	0.468	0.468	40	4.234	169.345	196.9	4	10.9
	B4		0.340	0.340	80	2.663	213.002			
	C4		0.207	0.207	160	1.335	213.559			
	D4		0.125	0.125	320	0.609	194.998			
	E4		0.083		640					
	F4		0.063		1280					
	G4		0.054		2560					
	H4		0.045		5120					
04	A5	4	0.318	0.318	40	2.425	97.011	100.8	3	6.2
	B5		0.209	0.209	80	1.353	108.247			
	C5		0.125	0.125	160	0.609	97.499			
	D5		0.089		320					
	E5		0.064		640					
	F5		0.050		1280					
	G5		0.045		2560					
	H5		0.042		5120					
05	A6	5	0.045		40			N/A	0	-----
	B6		0.040		80					
	C6		0.048		160					
	D6		0.049		320					
	E6		0.047		640					
	F6		0.047		1280					
	G6		0.042		2560					
	H6		0.043		5120					
06	A7	6	0.358	0.358	40	2.863	114.516	118.1	3	2.7
	B7		0.225	0.225	80	1.501	120.106			
	C7		0.141	0.141	160	0.748	119.656			
	D7		0.088		320					
	E7		0.069		640					
	F7		0.049		1280					
	G7		0.055		2560					
	H7		0.041		5120					
07	A8	7	0.240	0.240	40	1.643	65.712	63.8	2	4.2
	B8		0.144	0.144	80	0.774	61.913			
	C8		0.096		160					
	D8		0.073		320					
	E8		0.058		640					
	F8		0.054		1280					
	G8		0.047		2560					
	H8		0.038		5120					
08	A9	8	0.151	0.151	40	0.835	33.396	33.5	2	0.3
	B9		0.103	0.103	80	0.419	33.550			
	C9		0.075		160					
	D9		0.055		320					

Samples (Contd)

Sample	Wells	ID	OD	OK OD	Dilution	Calc.Conc	Adjusted.Conc	GMC	N	CVdil
	E9		0.047		640					
	F9		0.051		1280					
	G9		0.046		2560					
	H9		0.041		5120					
09	A10	9	0.093		40			N/A	0	-----
	B10		0.073		80					
	C10		0.063		160					
	D10		0.052		320					
	E10		0.050		640					
	F10		0.044		1280					
	G10		0.047		2560					
	H10		0.041		5120					
10	A11	10	0.049		40			N/A	0	-----
	B11		0.049		80					
	C11		0.049		160					
	D11		0.045		320					
	E11		0.051		640					
	F11		0.043		1280					
	G11		0.045		2560					
	H11		0.040		5120					
11	A12	11	0.043		40			N/A	0	-----
	B12		0.052		80					
	C12		0.051		160					
	D12		0.046		320					
	E12		0.043		640					
	F12		0.046		1280					
	G12		0.042		2560					
	H12		0.047		5120					

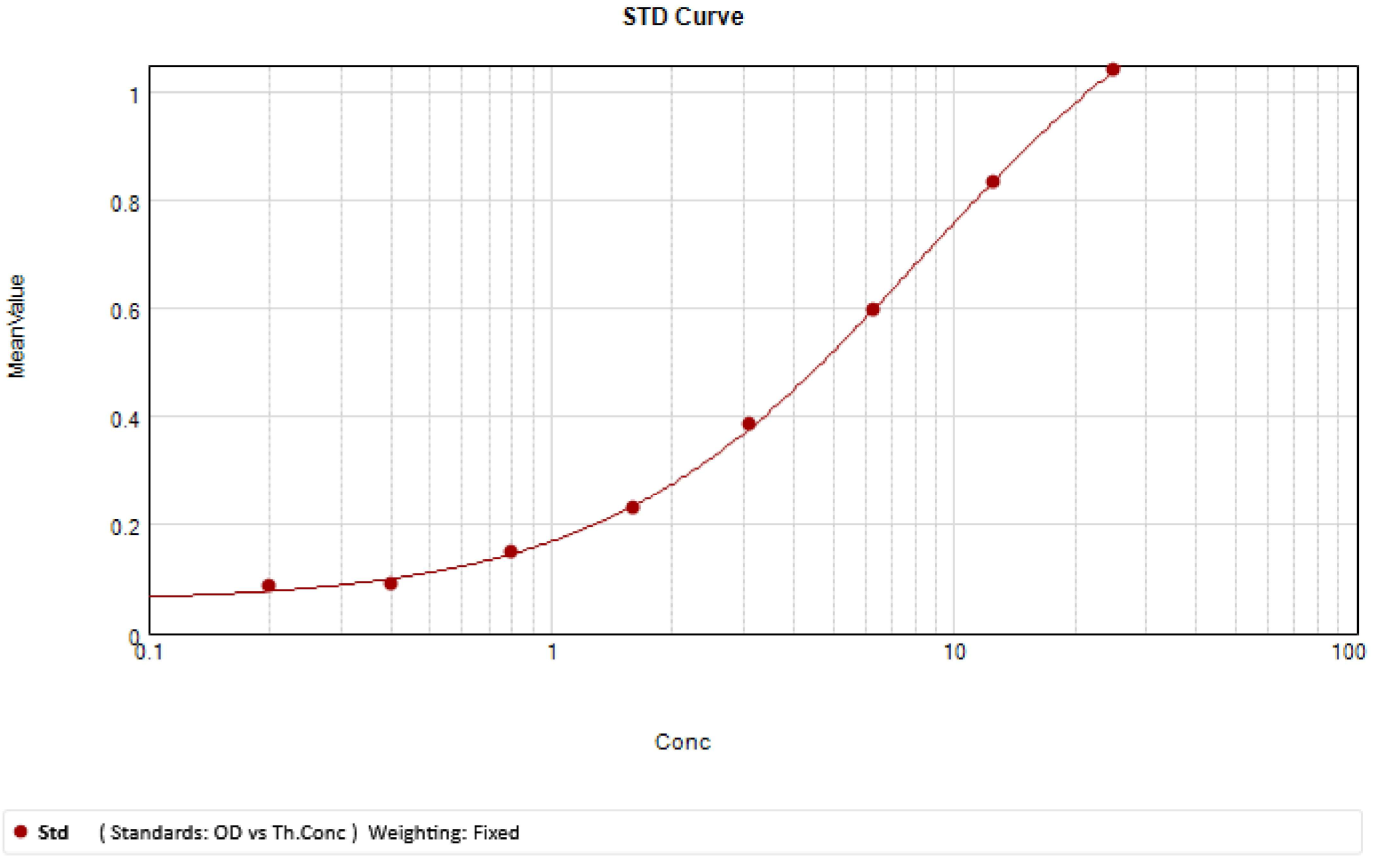

Curve Fit Results ▲

Curve Fit : 4-Parameter Logistic $y = D + \frac{A - D}{1 + \left(\frac{x}{C}\right)^B}$

	Parameter	Estimated Value	Std. Error	Confidence Interval
**Std** $R^2 = 1.000$ EC50 = 8.300	A	0.057	0.009	[0.031, 0.084]
	B	1.104	0.060	[0.938, 1.270]
	C	8.300	0.637	[6.530, 10.07]
	D	1.331	0.050	[1.192, 1.470]

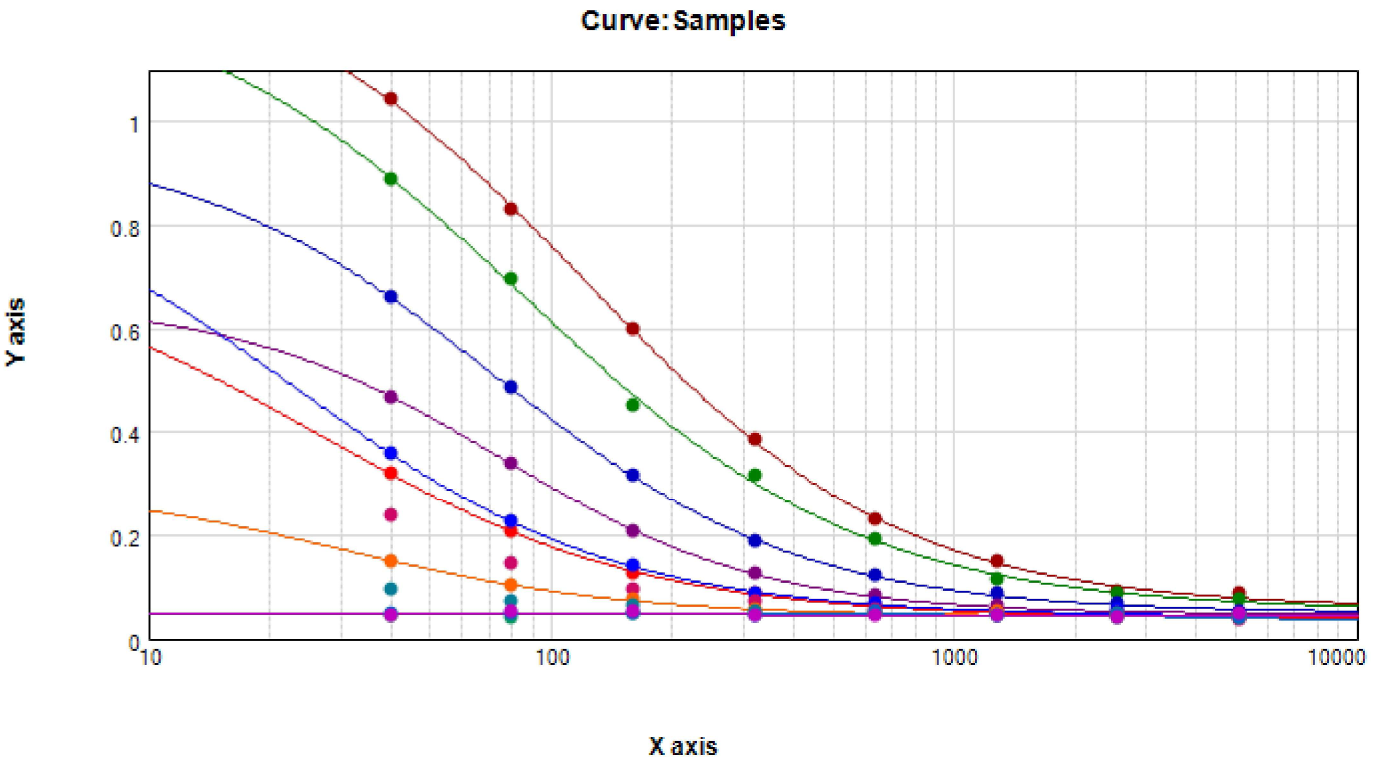

- **STD** (Standards: OD vs Dilution) Weighting: Fixed
- **S-1** (Samples: ODS1 vs DilSple1) Weighting: Fixed
- **S-2** (Samples: ODS2 vs DilSple2) Weighting: Fixed
- **S-3** (Samples: ODS3 vs DilSple3) Weighting: Fixed
- **S-4** (Samples: ODS4 vs DilSple4) Weighting: Fixed
- **S-5** (Samples: ODS5 vs DilSple5) Weighting: Fixed
- **S-6** (Samples: ODS6 vs DilSple6) Weighting: Fixed
- **S-7** (Samples: ODS7 vs DilSple7) Weighting: Fixed
- **S-8** (Samples: ODS8 vs DilSple8) Weighting: Fixed
- **S-9** (Samples: ODS9 vs DilSple9) Weighting: Fixed
- **S-10** (Samples: ODS10 vs DilSple10) Weighting: Fixed
- **S-11** (Samples: ODS11 vs DilSple11) Weighting: Fixed

Curve Fit Results ▼

Assay Parameter

Samples

Theoretical First Dilution Of Test Sample In Plate : 40.0   Sample dilution fold: 2.0

Nipha_Standard : NV-1

Concentration: 1000.0

Dilution (First dil in plate): 40.0

Dilution fold: 2.0

Others parameters

Rounding Decimal Standard Th.Conc: 1

Rounding Decimal RelErr% & CVdil: 1

Rounding Decimal GMC: 1

Average ODs of Blank: 0.046

SD of Blank: 0.004

Cutoff OD: 0.096
