# Supplementary material for: Development and Qualification of a Nipah Virus Glycoprotein-Specific IgG ELISA for the Assessment of Human Antibody Responses
Source: Vaccines (Basel). 2026 Jun 16;14(6):534. doi: 10.3390/vaccines14060534 (PMC13307770; doi:10.3390/vaccines14060534)
Supplement: Supplementary file 1 [file vaccines-14-00534-s001.zip › Supplementary_ELISA Qualification Data & Graph/3. Linearity_Analysist-1/4. Linearity_WHO IS_ANALYST-1_PLATE-2_DAY-1.pdf]

Intro

NIPAH_NIBSC_LINEARITY_ANALYST#1_PLATE#2_DAY#1

OD

	1	2	3	4	5	6	7	8	9	10	11	12
A	1.010	0.686	0.471	0.290	0.173	0.047	0.045	0.045	0.045	0.045	0.045	0.045
B	0.721	0.495	0.311	0.177	0.114	0.048	0.045	0.045	0.045	0.045	0.045	0.045
C	0.467	0.302	0.177	0.116	0.075	0.048	0.045	0.045	0.045	0.045	0.045	0.045
D	0.285	0.185	0.112	0.075	0.056	0.044	0.048	0.048	0.048	0.048	0.048	0.048
E	0.166	0.109	0.078	0.055	0.050	0.040	0.046	0.046	0.046	0.046	0.046	0.046
F	0.105	0.077	0.061	0.052	0.044	0.045	0.047	0.047	0.047	0.047	0.047	0.047
G	0.090	0.055	0.047	0.044	0.046	0.048	0.047	0.047	0.047	0.047	0.047	0.047
H	0.082	0.047	0.035	0.038	0.041	0.044	0.045	0.045	0.045	0.045	0.045	0.045

Settings Information

Endpoint

Absorbance

Lm1 450

More Settings

Shake Off

Calibrate On

Carriage Speed Normal

Column Priority

Read Information

Imported Data : 12:15 PM 10/2/2024

Imported By : anjan

Reduction Settings

Optical Density

Wavelength Combination : !Lm1

Sample Dil

Main Sample Dilution 40.0

Sample 1: NV-4 40.0

Sample 2: NV-4 (1:2) 40.0

Sample 3: NV-4 (1:4) 40.0

Sample 4: NV-4 (1:8) 40.0

Sample 5: CNC 40.0

Sample 6: BLANK 40.0

Sample 7: BLANK 40.0

Sample 8: BLANK 40.0

Sample 9: BLANK 40.0

Sample 10: BLANK 40.0

Sample 11: BLANK 40.0

Standards

Sample	Wells	OD	OK OD	Dilution	Calc.Conc	Adj.Conc	GMC	N	Th.Conc	RelErr%
01	A1	1.010	1.010	40	25.096	1003.8	971.1	6	25.000	0.400
	B1	0.721	0.721	80	12.372	989.8			12.500	-1.000
	C1	0.467	0.467	160	6.327	1012.3			6.300	0.400
	D1	0.285	0.285	320	3.240	1036.8			3.100	4.500
	E1	0.166	0.166	640	1.511	966.7			1.600	-5.600
	F1	0.105	0.105	1280	0.650	831.7			0.800	-18.800
	G1	0.090		2560					0.400	
	H1	0.082		5120					0.200	

Samples

Sample	Wells	ID	OD	OK OD	Dilution	Calc.Conc	Adjusted.Conc	GMC	N	CVdil
01	A2	1	0.686	0.686	40	11.360	454.385	514.5	5	11.6
	B2		0.495	0.495	80	6.872	549.783			
	C2		0.302	0.302	160	3.501	560.177			
	D2		0.185	0.185	320	1.778	569.064			
	E2		0.109	0.109	640	0.707	452.616			
	F2		0.077		1280					
	G2		0.055		2560					
	H2		0.047		5120					
02	A3	2	0.471	0.471	40	6.403	256.130	262.8	4	8.1
	B3		0.311	0.311	80	3.641	291.297			
	C3		0.177	0.177	160	1.665	266.458			
	D3		0.112	0.112	320	0.750	240.033			
	E3		0.078		640					
	F3		0.061		1280					
	G3		0.047		2560					
	H3		0.035		5120					
03	A4	3	0.290	0.290	40	3.316	132.652	131.7	3	1.7
	B4		0.177	0.177	80	1.665	133.229			
	C4		0.116	0.116	160	0.807	129.131			
	D4		0.075		320					
	E4		0.055		640					
	F4		0.052		1280					
	G4		0.044		2560					
	H4		0.038		5120					
04	A5	4	0.173	0.173	40	1.609	64.360	63.3	2	2.3
	B5		0.114	0.114	80	0.779	62.289			
	C5		0.075		160					
	D5		0.056		320					
	E5		0.050		640					
	F5		0.044		1280					
	G5		0.046		2560					
	H5		0.041		5120					
05	A6	5	0.047		40			N/A	0	-----
	B6		0.048		80					
	C6		0.048		160					
	D6		0.044		320					
	E6		0.040		640					
	F6		0.045		1280					
	G6		0.048		2560					
	H6		0.044		5120					
06	A7	6	0.045		40			N/A	0	-----
	B7		0.045		80					
	C7		0.045		160					
	D7		0.048		320					
	E7		0.046		640					
	F7		0.047		1280					
	G7		0.047		2560					
	H7		0.045		5120					
07	A8	7	0.045		40			N/A	0	-----
	B8		0.045		80					
	C8		0.045		160					
	D8		0.048		320					
	E8		0.046		640					
	F8		0.047		1280					
	G8		0.047		2560					
	H8		0.045		5120					
08	A9	8	0.045		40			N/A	0	-----
	B9		0.045		80					
	C9		0.045		160					
	D9		0.048		320					

Samples (Contd)

Sample	Wells	ID	OD	OK OD	Dilution	Calc.Conc	Adjusted.Conc	GMC	N	CVdil
	E9		0.046		640					
	F9		0.047		1280					
	G9		0.047		2560					
	H9		0.045		5120					
09	A10	9	0.045		40			N/A	0	-----
	B10		0.045		80					
	C10		0.045		160					
	D10		0.048		320					
	E10		0.046		640					
	F10		0.047		1280					
	G10		0.047		2560					
	H10		0.045		5120					
10	A11	10	0.045		40			N/A	0	-----
	B11		0.045		80					
	C11		0.045		160					
	D11		0.048		320					
	E11		0.046		640					
	F11		0.047		1280					
	G11		0.047		2560					
	H11		0.045		5120					
11	A12	11	0.045		40			N/A	0	-----
	B12		0.045		80					
	C12		0.045		160					
	D12		0.048		320					
	E12		0.046		640					
	F12		0.047		1280					
	G12		0.047		2560					
	H12		0.045		5120					

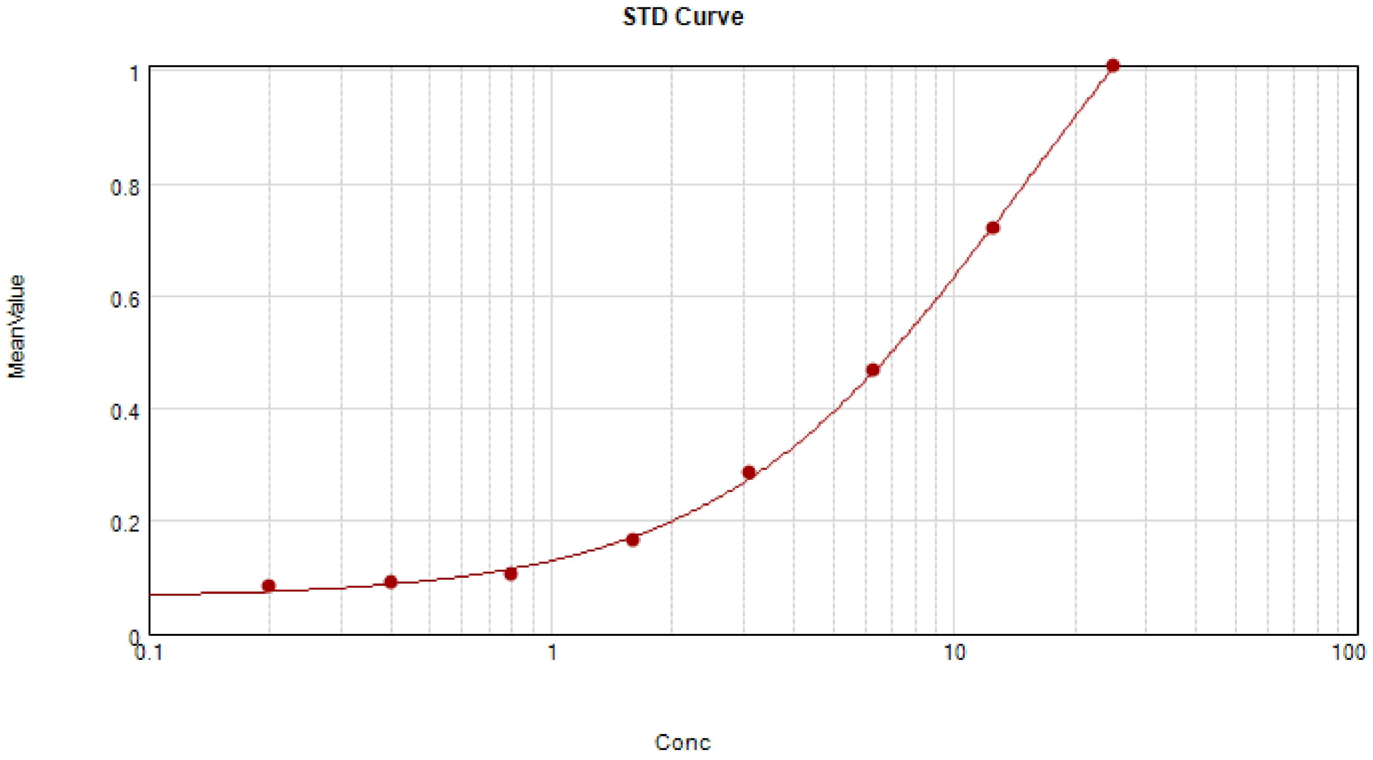

● **Std** (Standards: OD vs Th.Conc) Weighting: Fixed

Curve Fit Results ▲

Curve Fit : 4-Parameter Logistic $y = D + \frac{A - D}{1 + \left(\frac{x}{C}\right)^B}$

	Parameter	Estimated Value	Std. Error	Confidence Interval
**Std** $R^2 = 1.000$ EC50 = 15.01	A	0.064	0.008	[0.041, 0.087]
	B	1.132	0.073	[0.928, 1.336]
	C	15.01	2.053	[9.306, 20.71]
	D	1.539	0.110	[1.233, 1.844]

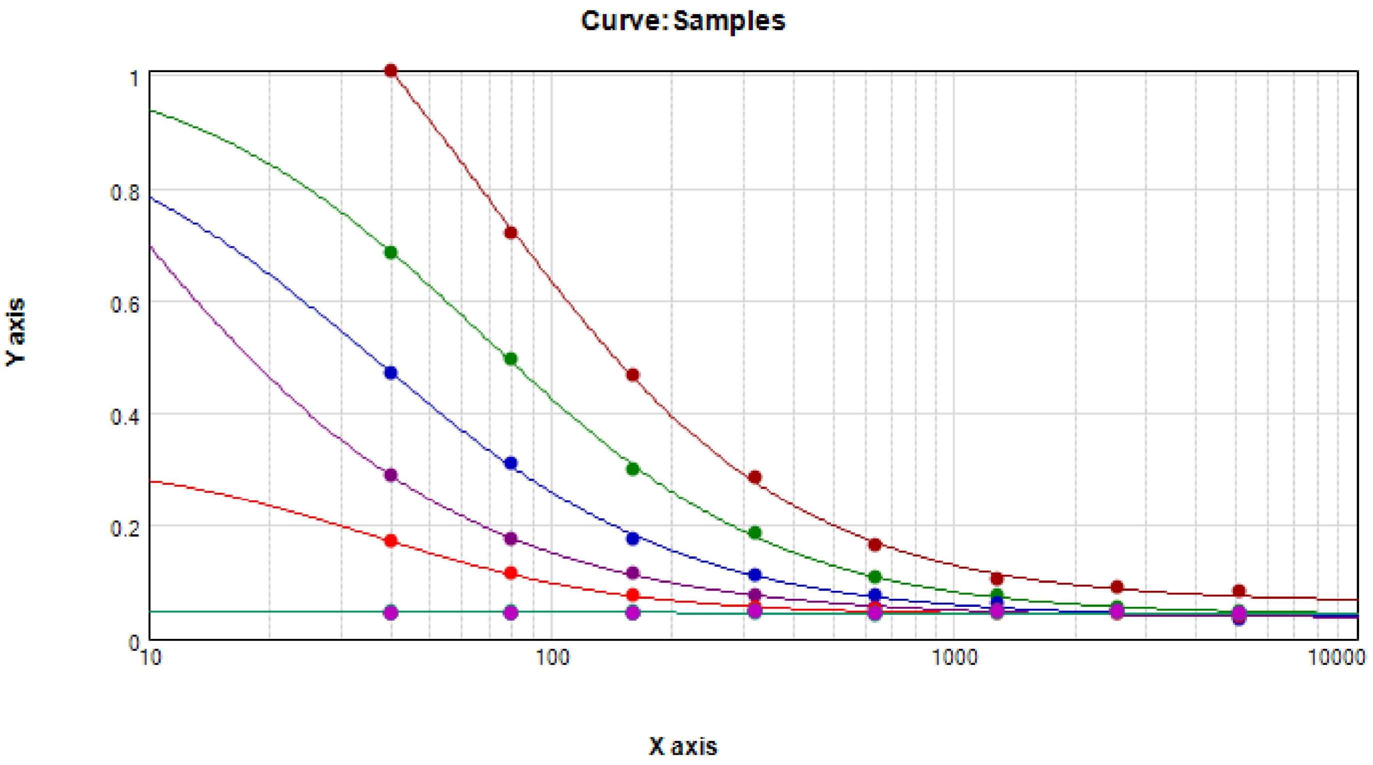

- **STD** (Standards: OD vs Dilution) Weighting: Fixed
- **S-1** (Samples: ODS1 vs DilSple1) Weighting: Fixed
- **S-2** (Samples: ODS2 vs DilSple2) Weighting: Fixed
- **S-3** (Samples: ODS3 vs DilSple3) Weighting: Fixed
- **S-4** (Samples: ODS4 vs DilSple4) Weighting: Fixed
- **S-5** (Samples: ODS5 vs DilSple5) Weighting: Fixed
- **S-6** (Samples: ODS6 vs DilSple6) Weighting: Fixed
- **S-7** (Samples: ODS7 vs DilSple7) Weighting: Fixed
- **S-8** (Samples: ODS8 vs DilSple8) Weighting: Fixed
- **S-9** (Samples: ODS9 vs DilSple9) Weighting: Fixed
- **S-10** (Samples: ODS10 vs DilSple10) Weighting: Fixed
- **S-11** (Samples: ODS11 vs DilSple11) Weighting: Fixed

Curve Fit Results ▼

Assay Parameter

Samples

Theoretical First Dilution Of Test Sample In Plate : 40.0   Sample dilution fold: 2.0

Nipha_Standard : NV-1

Concentration: 1000.0

Dilution (First dil in plate): 40.0

Dilution fold: 2.0

Others parameters

Rounding Decimal Standard Th.Conc: 1

Rounding Decimal RelErr% & CVdil: 1

Rounding Decimal GMC: 1

Average ODs of Blank: 0.046

SD of Blank: 0.001

Cutoff OD: 0.093
