# Supplementary material for: Development and Qualification of a Nipah Virus Glycoprotein-Specific IgG ELISA for the Assessment of Human Antibody Responses
Source: Vaccines (Basel). 2026 Jun 16;14(6):534. doi: 10.3390/vaccines14060534 (PMC13307770; doi:10.3390/vaccines14060534)
Supplement: Supplementary file 1 [file vaccines-14-00534-s001.zip › Supplementary_ELISA Qualification Data & Graph/3. Linearity_Analysist-1/5. Linearity_WHO IS_ANALYST-1_PLATE-2_DAY-2.pdf]

Intro

NIPAH_NIBSC_LINEARITY_ANALYST#1_PLATE#2_DAY#2

OD

	1	2	3	4	5	6	7	8	9	10	11	12
A	1.012	0.679	0.477	0.292	0.176	0.040	0.044	0.044	0.044	0.044	0.044	0.044
B	0.724	0.487	0.315	0.179	0.109	0.045	0.045	0.045	0.045	0.045	0.045	0.045
C	0.483	0.299	0.176	0.113	0.086	0.048	0.045	0.045	0.045	0.045	0.045	0.045
D	0.278	0.188	0.123	0.085	0.064	0.044	0.047	0.047	0.047	0.047	0.047	0.047
E	0.167	0.112	0.089	0.064	0.053	0.043	0.045	0.045	0.045	0.045	0.045	0.045
F	0.110	0.087	0.065	0.055	0.047	0.048	0.047	0.047	0.047	0.047	0.047	0.047
G	0.091	0.065	0.048	0.042	0.041	0.048	0.048	0.048	0.048	0.048	0.048	0.048
H	0.085	0.043	0.039	0.034	0.039	0.044	0.045	0.045	0.045	0.045	0.045	0.045

Settings Information
Endpoint
Absorbance
Lm1 450
More Settings
Shake Off
Calibrate On
Carriage Speed Normal
Column Priority

Read Information
Imported Data : 4:49 PM 10/2/2024
Imported By : anjan

Reduction Settings

Optical Density
Wavelength Combination : !Lm1

Sample Dil

Main Sample Dilution 40.0

Sample 1: NV-4 40.0

Sample 2: NV-4 (1:2) 40.0

Sample 3: NV-4 (1:4) 40.0

Sample 4: NV-4 (1:8) 40.0

Sample 5: CNC 40.0

Sample 6: BLANK 40.0

Sample 7: BLANK 40.0

Sample 8: BLANK 40.0

Sample 9: BLANK 40.0

Sample 10: BLANK 40.0

Sample 11: BLANK 40.0

Standards

Sample	Wells	OD	OK OD	Dilution	Calc.Conc	Adj.Conc	GMC	N	Th.Conc	RelErr%
01	A1	1.012	1.012	40	25.197	1007.9	980.5	6	25.000	0.800
	B1	0.724	0.724	80	12.207	976.6			12.500	-2.300
	C1	0.483	0.483	160	6.514	1042.2			6.300	3.400
	D1	0.278	0.278	320	3.105	993.6			3.100	0.200
	E1	0.167	0.167	640	1.516	970.0			1.600	-5.300
	F1	0.110	0.110	1280	0.702	898.8			0.800	-12.200
	G1	0.091		2560					0.400	
	H1	0.085		5120					0.200	

Samples

Sample	Wells	ID	OD	OK OD	Dilution	Calc.Conc	Adjusted.Conc	GMC	N	CVdil
01	A2	1	0.679	0.679	40	10.932	437.287	509.2	5	11.6
	B2		0.487	0.487	80	6.590	527.208			
	C2		0.299	0.299	160	3.419	547.031			
	D2		0.188	0.188	320	1.811	579.498			
	E2		0.112	0.112	640	0.732	468.259			
	F2		0.087		1280					
	G2		0.065		2560					
	H2		0.043		5120					
02	A3	2	0.477	0.477	40	6.401	256.021	273.9	4	6.5
	B3		0.315	0.315	80	3.662	292.990			
	C3		0.176	0.176	160	1.642	262.746			
	D3		0.123	0.123	320	0.892	285.361			
	E3		0.089		640					
	F3		0.065		1280					
	G3		0.048		2560					
	H3		0.039		5120					
03	A4	3	0.292	0.292	40	3.314	132.544	128.7	3	6.6
	B4		0.179	0.179	80	1.684	134.747			
	C4		0.113	0.113	160	0.746	119.414			
	D4		0.085		320					
	E4		0.064		640					
	F4		0.055		1280					
	G4		0.042		2560					
	H4		0.034		5120					
04	A5	4	0.176	0.176	40	1.642	65.687	60.1	2	12.6
	B5		0.109	0.109	80	0.687	54.994			
	C5		0.086		160					
	D5		0.064		320					
	E5		0.053		640					
	F5		0.047		1280					
	G5		0.041		2560					
	H5		0.039		5120					
05	A6	5	0.040		40			N/A	0	-----
	B6		0.045		80					
	C6		0.048		160					
	D6		0.044		320					
	E6		0.043		640					
	F6		0.048		1280					
	G6		0.048		2560					
	H6		0.044		5120					
06	A7	6	0.044		40			N/A	0	-----
	B7		0.045		80					
	C7		0.045		160					
	D7		0.047		320					
	E7		0.045		640					
	F7		0.047		1280					
	G7		0.048		2560					
	H7		0.045		5120					
07	A8	7	0.044		40			N/A	0	-----
	B8		0.045		80					
	C8		0.045		160					
	D8		0.047		320					
	E8		0.045		640					
	F8		0.047		1280					
	G8		0.048		2560					
	H8		0.045		5120					
08	A9	8	0.044		40			N/A	0	-----
	B9		0.045		80					
	C9		0.045		160					
	D9		0.047		320					

Samples (Contd)

Sample	Wells	ID	OD	OK OD	Dilution	Calc.Conc	Adjusted.Conc	GMC	N	CVdil
	E9		0.045		640					
	F9		0.047		1280					
	G9		0.048		2560					
	H9		0.045		5120					
09	A10	9	0.044		40			N/A	0	-----
	B10		0.045		80					
	C10		0.045		160					
	D10		0.047		320					
	E10		0.045		640					
	F10		0.047		1280					
	G10		0.048		2560					
	H10		0.045		5120					
10	A11	10	0.044		40			N/A	0	-----
	B11		0.045		80					
	C11		0.045		160					
	D11		0.047		320					
	E11		0.045		640					
	F11		0.047		1280					
	G11		0.048		2560					
	H11		0.045		5120					
11	A12	11	0.044		40			N/A	0	-----
	B12		0.045		80					
	C12		0.045		160					
	D12		0.047		320					
	E12		0.045		640					
	F12		0.047		1280					
	G12		0.048		2560					
	H12		0.045		5120					

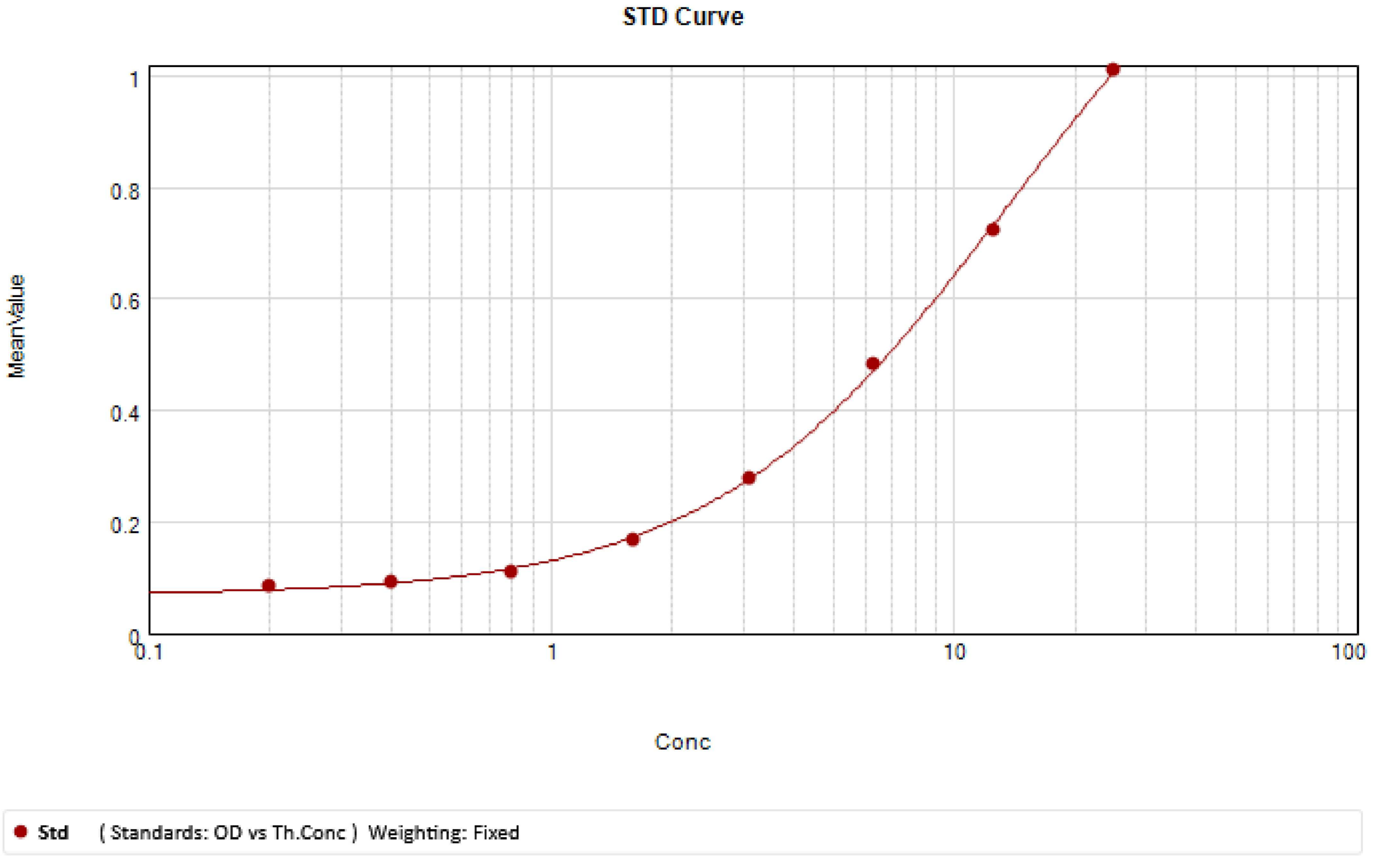

Curve Fit Results ▲

Curve Fit : 4-Parameter Logistic $y = D + \frac{A - D}{1 + \left(\frac{x}{C}\right)^B}$

	Parameter	Estimated Value	Std. Error	Confidence Interval
**Std**<br>R^2 = 1.000<br>EC50 = 13.64	A	0.068	0.009	[0.044, 0.093]
	B	1.175	0.079	[0.955, 1.394]
	C	13.64	1.739	[8.810, 18.47]
	D	1.471	0.099	[1.196, 1.746]

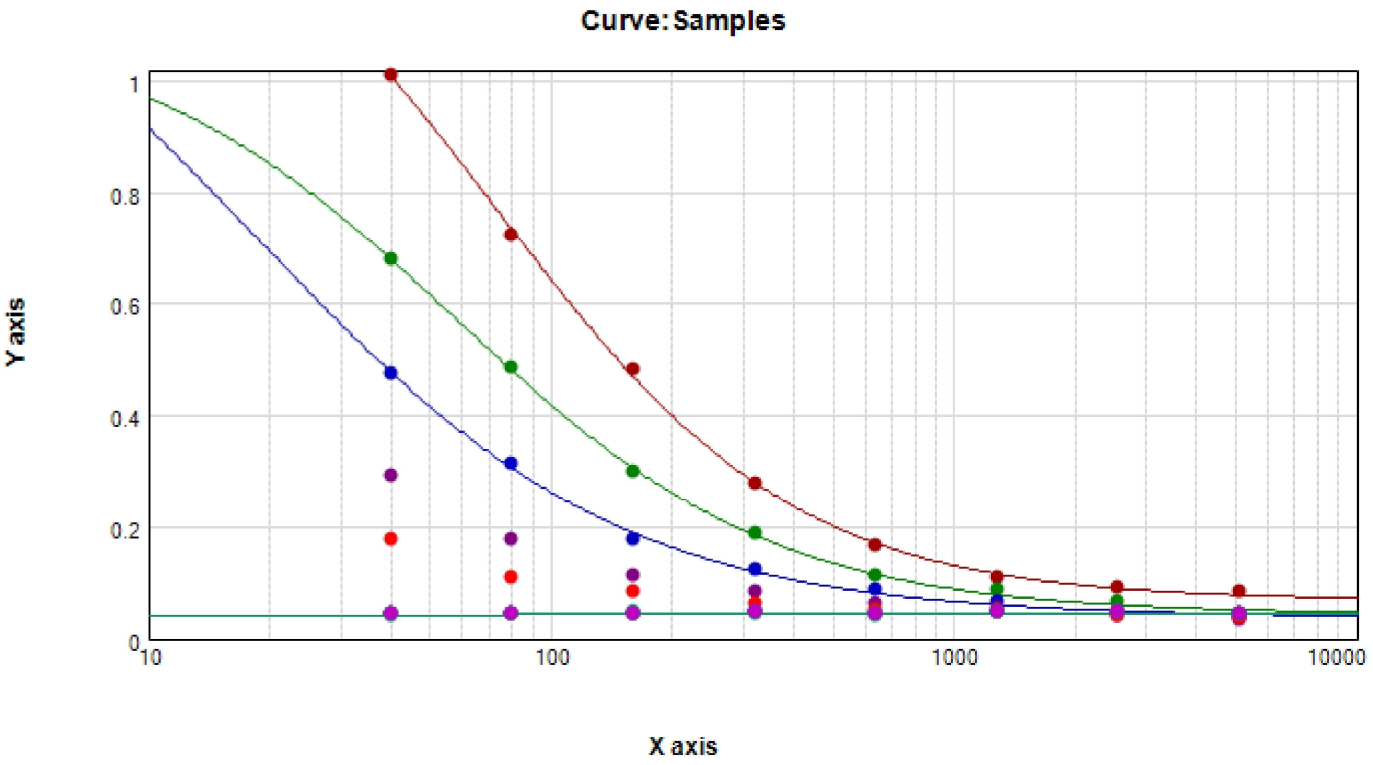

- **STD** (Standards: OD vs Dilution) Weighting: Fixed
- **S-1** (Samples: ODS1 vs DilSple1) Weighting: Fixed
- **S-2** (Samples: ODS2 vs DilSple2) Weighting: Fixed
- **S-3** (Samples: ODS3 vs DilSple3) Weighting: Fixed
- **S-4** (Samples: ODS4 vs DilSple4) Weighting: Fixed
- **S-5** (Samples: ODS5 vs DilSple5) Weighting: Fixed
- **S-6** (Samples: ODS6 vs DilSple6) Weighting: Fixed
- **S-7** (Samples: ODS7 vs DilSple7) Weighting: Fixed
- **S-8** (Samples: ODS8 vs DilSple8) Weighting: Fixed
- **S-9** (Samples: ODS9 vs DilSple9) Weighting: Fixed
- **S-10** (Samples: ODS10 vs DilSple10) Weighting: Fixed
- **S-11** (Samples: ODS11 vs DilSple11) Weighting: Fixed

Curve Fit Results ▼

Assay Parameter

Samples

Theoretical First Dilution Of Test Sample In Plate : 40.0 Sample dilution fold: 2.0

Nipha_Standard : NV-1

Concentration: 1000.0

Dilution (First dil in plate): 40.0

Dilution fold: 2.0

Others parameters

Rounding Decimal Standard Th.Conc: 1

Rounding Decimal RelErr% & CVdil: 1

Rounding Decimal GMC: 1

Average ODs of Blank: 0.046

SD of Blank: 0.001

Cutoff OD: 0.093
