# Supplementary material for: Development and Qualification of a Nipah Virus Glycoprotein-Specific IgG ELISA for the Assessment of Human Antibody Responses
Source: Vaccines (Basel). 2026 Jun 16;14(6):534. doi: 10.3390/vaccines14060534 (PMC13307770; doi:10.3390/vaccines14060534)
Supplement: Supplementary file 1 [file vaccines-14-00534-s001.zip › Supplementary_ELISA Qualification Data & Graph/3. Linearity_Analysist-1/6. Linearity_WHO IS_ANALYST-1_PLATE-2_DAY-3.pdf]

Intro

NIPAH_NIBSC_LINEARITY_ANALYST#1_PLATE#2_DAY#3

OD

	1	2	3	4	5	6	7	8	9	10	11	12
A	1.022	0.687	0.471	0.284	0.175	0.043	0.046	0.044	0.044	0.044	0.044	0.044
B	0.726	0.488	0.313	0.172	0.111	0.046	0.044	0.045	0.045	0.045	0.045	0.045
C	0.432	0.294	0.178	0.115	0.073	0.037	0.048	0.045	0.045	0.045	0.045	0.045
D	0.246	0.191	0.119	0.076	0.059	0.038	0.045	0.047	0.047	0.047	0.047	0.047
E	0.168	0.116	0.074	0.056	0.053	0.040	0.047	0.045	0.045	0.045	0.045	0.045
F	0.122	0.078	0.061	0.055	0.042	0.043	0.048	0.047	0.047	0.047	0.047	0.047
G	0.092	0.059	0.045	0.041	0.040	0.040	0.045	0.048	0.048	0.048	0.048	0.048
H	0.085	0.045	0.041	0.035	0.037	0.038	0.046	0.045	0.045	0.045	0.045	0.045

Settings Information
Endpoint
Absorbance
Lm1 450
More Settings
Shake Off
Calibrate On
Carriage Speed Normal
Column Priority

Read Information
Imported Data : 4:51 PM 10/2/2024
Imported By : anjan

Reduction Settings

Optical Density
Wavelength Combination : !Lm1

Sample Dil

Main Sample Dilution 40.0

Sample 1: NV-4 40.0

Sample 2: NV-4 (1:2) 40.0

Sample 3: NV-4 (1:4) 40.0

Sample 4: NV-4 (1:8) 40.0

Sample 5: CNC 40.0

Sample 6: BLANK 40.0

Sample 7: BLANK 40.0

Sample 8: BLANK 40.0

Sample 9: BLANK 40.0

Sample 10: BLANK 40.0

Sample 11: BLANK 40.0

Standards

Sample	Wells	OD	OK OD	Dilution	Calc.Conc	Adj.Conc	GMC	N	Th.Conc	RelErr%
01	A1	1.022	1.022	40	24.891	995.6	1045.5	6	25.000	-0.400
	B1	0.726	0.726	80	12.668	1013.4			12.500	1.300
	C1	0.432	0.432	160	6.175	988.0			6.300	-2.000
	D1	0.246	0.246	320	3.046	974.6			3.100	-1.800
	E1	0.168	0.168	640	1.760	1126.7			1.600	10.000
	F1	0.122	0.122	1280	0.932	1192.8			0.800	16.500
	G1	0.092		2560					0.400	
	H1	0.085		5120					0.200	

Samples

Sample	Wells	ID	OD	OK OD	Dilution	Calc.Conc	Adjusted.Conc	GMC	N	CVdil
01	A2	1	0.687	0.687	40	11.618	464.719	567.1	5	15.1
	B2		0.488	0.488	80	7.214	577.104			
	C2		0.294	0.294	160	3.825	612.018			
	D2		0.191	0.191	320	2.147	687.026			
	E2		0.116	0.116	640	0.813	520.268			
	F2		0.078		1280					
	G2		0.059		2560					
	H2		0.045		5120					
02	A3	2	0.471	0.471	40	6.891	275.658	297.8	4	8.7
	B3		0.313	0.313	80	4.136	330.920			
	C3		0.178	0.178	160	1.930	308.773			
	D3		0.119	0.119	320	0.873	279.334			
	E3		0.074		640					
	F3		0.061		1280					
	G3		0.045		2560					
	H3		0.041		5120					
03	A4	3	0.284	0.284	40	3.662	146.483	139.5	3	8.3
	B4		0.172	0.172	80	1.829	146.280			
	C4		0.115	0.115	160	0.793	126.826			
	D4		0.076		320					
	E4		0.056		640					
	F4		0.055		1280					
	G4		0.041		2560					
	H4		0.035		5120					
04	A5	4	0.175	0.175	40	1.879	75.171	65.4	2	20.0
	B5		0.111	0.111	80	0.710	56.817			
	C5		0.073		160					
	D5		0.059		320					
	E5		0.053		640					
	F5		0.042		1280					
	G5		0.040		2560					
	H5		0.037		5120					
05	A6	5	0.043		40			N/A	0	-----
	B6		0.046		80					
	C6		0.037		160					
	D6		0.038		320					
	E6		0.040		640					
	F6		0.043		1280					
	G6		0.040		2560					
	H6		0.038		5120					
06	A7	6	0.046		40			N/A	0	-----
	B7		0.044		80					
	C7		0.048		160					
	D7		0.045		320					
	E7		0.047		640					
	F7		0.048		1280					
	G7		0.045		2560					
	H7		0.046		5120					
07	A8	7	0.044		40			N/A	0	-----
	B8		0.045		80					
	C8		0.045		160					
	D8		0.047		320					
	E8		0.045		640					
	F8		0.047		1280					
	G8		0.048		2560					
	H8		0.045		5120					
08	A9	8	0.044		40			N/A	0	-----
	B9		0.045		80					
	C9		0.045		160					
	D9		0.047		320					

Samples (Contd)

Sample	Wells	ID	OD	OK OD	Dilution	Calc.Conc	Adjusted.Conc	GMC	N	CVdil
	E9		0.045		640					
	F9		0.047		1280					
	G9		0.048		2560					
	H9		0.045		5120					
09	A10	9	0.044		40			N/A	0	-----
	B10		0.045		80					
	C10		0.045		160					
	D10		0.047		320					
	E10		0.045		640					
	F10		0.047		1280					
	G10		0.048		2560					
	H10		0.045		5120					
10	A11	10	0.044		40			N/A	0	-----
	B11		0.045		80					
	C11		0.045		160					
	D11		0.047		320					
	E11		0.045		640					
	F11		0.047		1280					
	G11		0.048		2560					
	H11		0.045		5120					
11	A12	11	0.044		40			N/A	0	-----
	B12		0.045		80					
	C12		0.045		160					
	D12		0.047		320					
	E12		0.045		640					
	F12		0.047		1280					
	G12		0.048		2560					
	H12		0.045		5120					

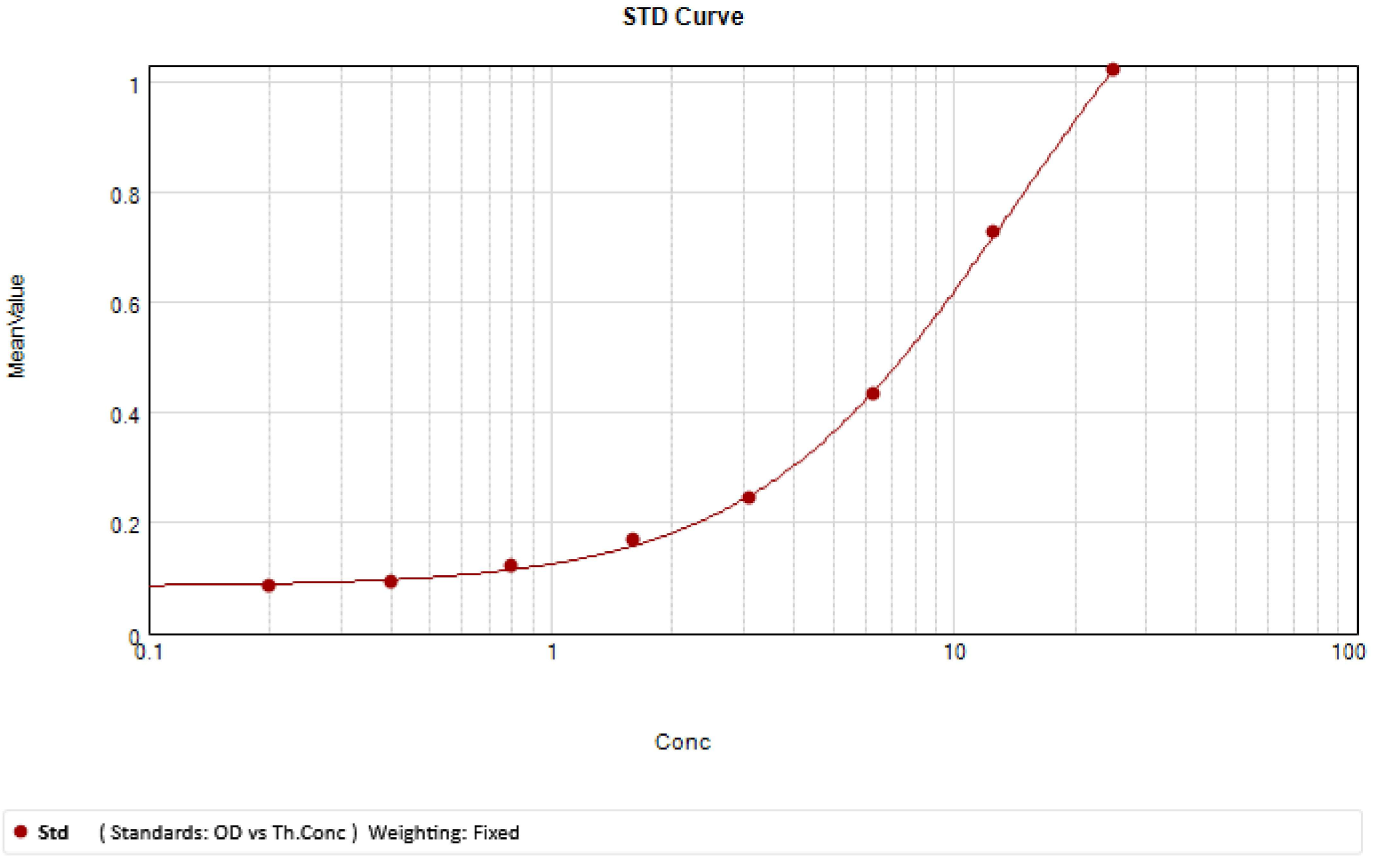

Curve Fit Results ▲

Curve Fit : 4-Parameter Logistic $y = D + \frac{A - D}{1 + \left(\frac{x}{C}\right)^B}$

	Parameter	Estimated Value	Std. Error	Confidence Interval
**Std** R^2 = 1.000 EC50 = 14.10	A	0.085	0.007	[0.067, 0.103]
	B	1.320	0.075	[1.113, 1.527]
	C	14.10	1.365	[10.32, 17.89]
	D	1.465	0.081	[1.240, 1.690]

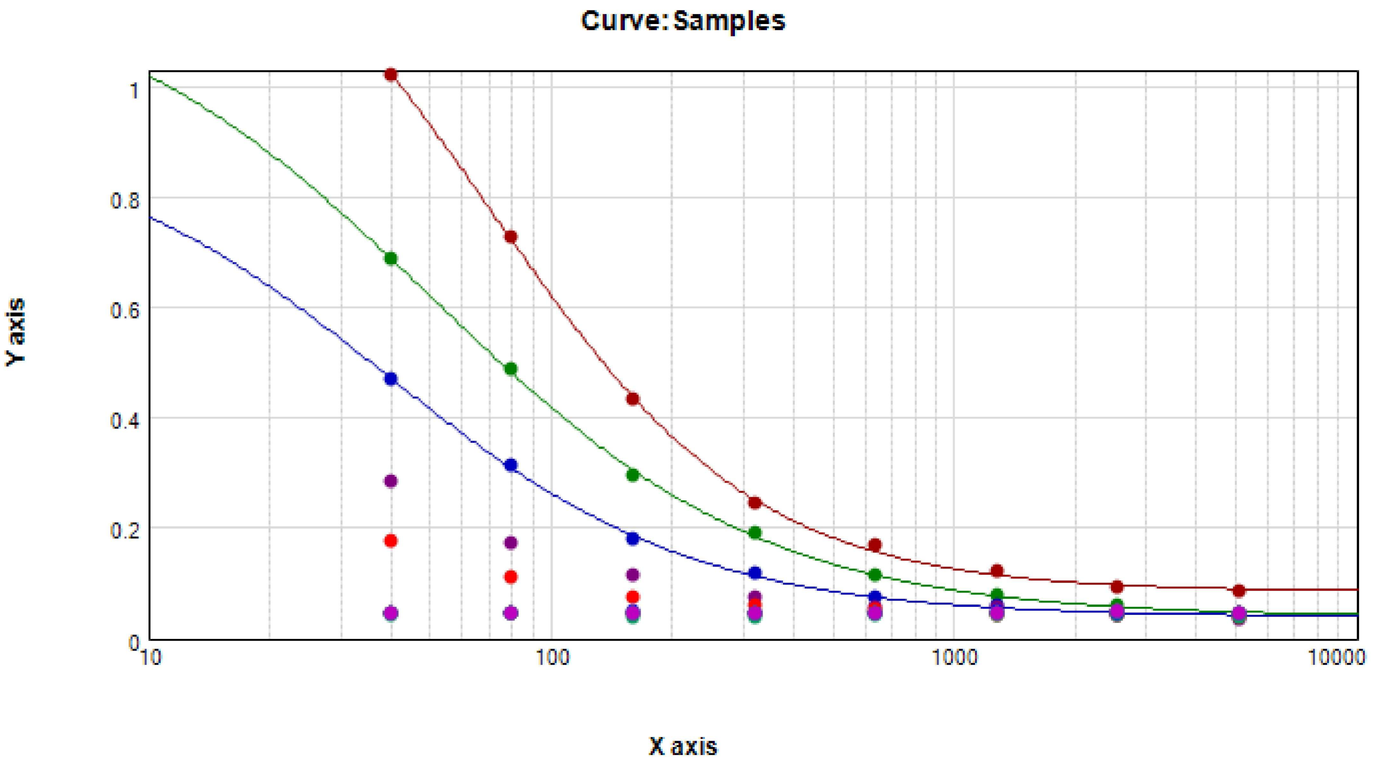

- **STD** (Standards: OD vs Dilution) Weighting: Fixed
- **S-1** (Samples: ODS1 vs DilSple1) Weighting: Fixed
- **S-2** (Samples: ODS2 vs DilSple2) Weighting: Fixed
- **S-3** (Samples: ODS3 vs DilSple3) Weighting: Fixed
- **S-4** (Samples: ODS4 vs DilSple4) Weighting: Fixed
- **S-5** (Samples: ODS5 vs DilSple5) Weighting: Fixed
- **S-6** (Samples: ODS6 vs DilSple6) Weighting: Fixed
- **S-7** (Samples: ODS7 vs DilSple7) Weighting: Fixed
- **S-8** (Samples: ODS8 vs DilSple8) Weighting: Fixed
- **S-9** (Samples: ODS9 vs DilSple9) Weighting: Fixed
- **S-10** (Samples: ODS10 vs DilSple10) Weighting: Fixed
- **S-11** (Samples: ODS11 vs DilSple11) Weighting: Fixed

Curve Fit Results ▼

Assay Parameter

Samples

Theoretical First Dilution Of Test Sample In Plate : 40.0   Sample dilution fold: 2.0

Nipha_Standard : NV-1

Concentration: 1000.0

Dilution (First dil in plate): 40.0

Dilution fold: 2.0

Others parameters

Rounding Decimal Standard Th.Conc: 1

Rounding Decimal RelErr% & CVdil: 1

Rounding Decimal GMC: 1

Average ODs of Blank: 0.046

SD of Blank: 0.001

Cutoff OD: 0.093
