# Supplementary material for: Development and Qualification of a Nipah Virus Glycoprotein-Specific IgG ELISA for the Assessment of Human Antibody Responses
Source: Vaccines (Basel). 2026 Jun 16;14(6):534. doi: 10.3390/vaccines14060534 (PMC13307770; doi:10.3390/vaccines14060534)
Supplement: Supplementary file 1 [file vaccines-14-00534-s001.zip › Supplementary_ELISA Qualification Data & Graph/4. Linearity_Analysist-2/6. Linearity_WHO IS_ANALYST-2_PLATE-2_DAY-3.pdf]

Intro

NIPAH_NIBSC_LINEARITY_ANALYST#2_PLATE#2_DAY#3

OD

	1	2	3	4	5	6	7	8	9	10	11	12
A	1.023	0.686	0.473	0.286	0.175	0.043	0.047	0.047	0.047	0.047	0.047	0.047
B	0.724	0.486	0.315	0.174	0.110	0.046	0.044	0.044	0.044	0.044	0.044	0.044
C	0.431	0.297	0.177	0.117	0.079	0.042	0.048	0.048	0.048	0.048	0.048	0.048
D	0.247	0.196	0.118	0.077	0.061	0.044	0.045	0.045	0.045	0.045	0.045	0.045
E	0.169	0.114	0.073	0.056	0.053	0.040	0.047	0.047	0.047	0.047	0.047	0.047
F	0.120	0.079	0.061	0.053	0.042	0.043	0.048	0.048	0.048	0.048	0.048	0.048
G	0.092	0.060	0.045	0.041	0.041	0.044	0.045	0.045	0.045	0.045	0.045	0.045
H	0.087	0.045	0.042	0.039	0.035	0.040	0.047	0.047	0.047	0.047	0.047	0.047

Settings Information
Endpoint
Absorbance
Lm1 450
More Settings
Shake Off
Calibrate On
Carriage Speed Normal
Column Priority

Read Information
Imported Data : 12:05 PM 10/3/2024
Imported By : anjan

Reduction Settings

Optical Density
Wavelength Combination : !Lm1

Sample Dil

Main Sample Dilution 40.0

Sample 1: NV-4 40.0

Sample 2: NV-4 (1:2) 40.0

Sample 3: NV-4 (1:4) 40.0

Sample 4: NV-4 (1:8) 40.0

Sample 5: CNC 40.0

Sample 6: BLANK 40.0

Sample 7: BLANK 40.0

Sample 8: BLANK 40.0

Sample 9: BLANK 40.0

Sample 10: BLANK 40.0

Sample 11: BLANK 40.0

Standards

Sample	Wells	OD	OK OD	Dilution	Calc.Conc	Adj.Conc	GMC	N	Th.Conc	RelErr%
01	A1	1.023	1.023	40	24.899	996.0	1037.3	6	25.000	-0.400
	B1	0.724	0.724	80	12.661	1012.9			12.500	1.300
	C1	0.431	0.431	160	6.173	987.6			6.300	-2.000
	D1	0.247	0.247	320	3.057	978.1			3.100	-1.400
	E1	0.169	0.169	640	1.767	1131.1			1.600	10.500
	F1	0.120	0.120	1280	0.883	1129.9			0.800	10.300
	G1	0.092		2560					0.400	
	H1	0.087		5120					0.200	

Samples

Sample	Wells	ID	OD	OK OD	Dilution	Calc.Conc	Adjusted.Conc	GMC	N	CVdil
01	A2	1	0.686	0.686	40	11.638	465.531	565.2	5	17.5
	B2		0.486	0.486	80	7.199	575.893			
	C2		0.297	0.297	160	3.874	619.841			
	D2		0.196	0.196	320	2.221	710.685			
	E2		0.114	0.114	640	0.763	488.189			
	F2		0.079		1280					
	G2		0.060		2560					
	H2		0.045		5120					
02	A3	2	0.473	0.473	40	6.950	278.019	295.5	4	9.6
	B3		0.315	0.315	80	4.171	333.700			
	C3		0.177	0.177	160	1.903	304.506			
	D3		0.118	0.118	320	0.843	269.834			
	E3		0.073		640					
	F3		0.061		1280					
	G3		0.045		2560					
	H3		0.042		5120					
03	A4	3	0.286	0.286	40	3.693	147.735	142.3	3	6.7
	B4		0.174	0.174	80	1.852	148.192			
	C4		0.117	0.117	160	0.823	131.729			
	D4		0.077		320					
	E4		0.056		640					
	F4		0.053		1280					
	G4		0.041		2560					
	H4		0.039		5120					
04	A5	4	0.175	0.175	40	1.869	74.774	63.8	2	22.8
	B5		0.110	0.110	80	0.680	54.412			
	C5		0.079		160					
	D5		0.061		320					
	E5		0.053		640					
	F5		0.042		1280					
	G5		0.041		2560					
	H5		0.035		5120					
05	A6	5	0.043		40			N/A	0	-----
	B6		0.046		80					
	C6		0.042		160					
	D6		0.044		320					
	E6		0.040		640					
	F6		0.043		1280					
	G6		0.044		2560					
	H6		0.040		5120					
06	A7	6	0.047		40			N/A	0	-----
	B7		0.044		80					
	C7		0.048		160					
	D7		0.045		320					
	E7		0.047		640					
	F7		0.048		1280					
	G7		0.045		2560					
	H7		0.047		5120					
07	A8	7	0.047		40			N/A	0	-----
	B8		0.044		80					
	C8		0.048		160					
	D8		0.045		320					
	E8		0.047		640					
	F8		0.048		1280					
	G8		0.045		2560					
	H8		0.047		5120					
08	A9	8	0.047		40			N/A	0	-----
	B9		0.044		80					
	C9		0.048		160					
	D9		0.045		320					

Samples (Contd)

Sample	Wells	ID	OD	OK OD	Dilution	Calc.Conc	Adjusted.Conc	GMC	N	CVdil
	E9		0.047		640					
	F9		0.048		1280					
	G9		0.045		2560					
	H9		0.047		5120					
09	A10	9	0.047		40			N/A	0	-----
	B10		0.044		80					
	C10		0.048		160					
	D10		0.045		320					
	E10		0.047		640					
	F10		0.048		1280					
	G10		0.045		2560					
	H10		0.047		5120					
10	A11	10	0.047		40			N/A	0	-----
	B11		0.044		80					
	C11		0.048		160					
	D11		0.045		320					
	E11		0.047		640					
	F11		0.048		1280					
	G11		0.045		2560					
	H11		0.047		5120					
11	A12	11	0.047		40			N/A	0	-----
	B12		0.044		80					
	C12		0.048		160					
	D12		0.045		320					
	E12		0.047		640					
	F12		0.048		1280					
	G12		0.045		2560					
	H12		0.047		5120					

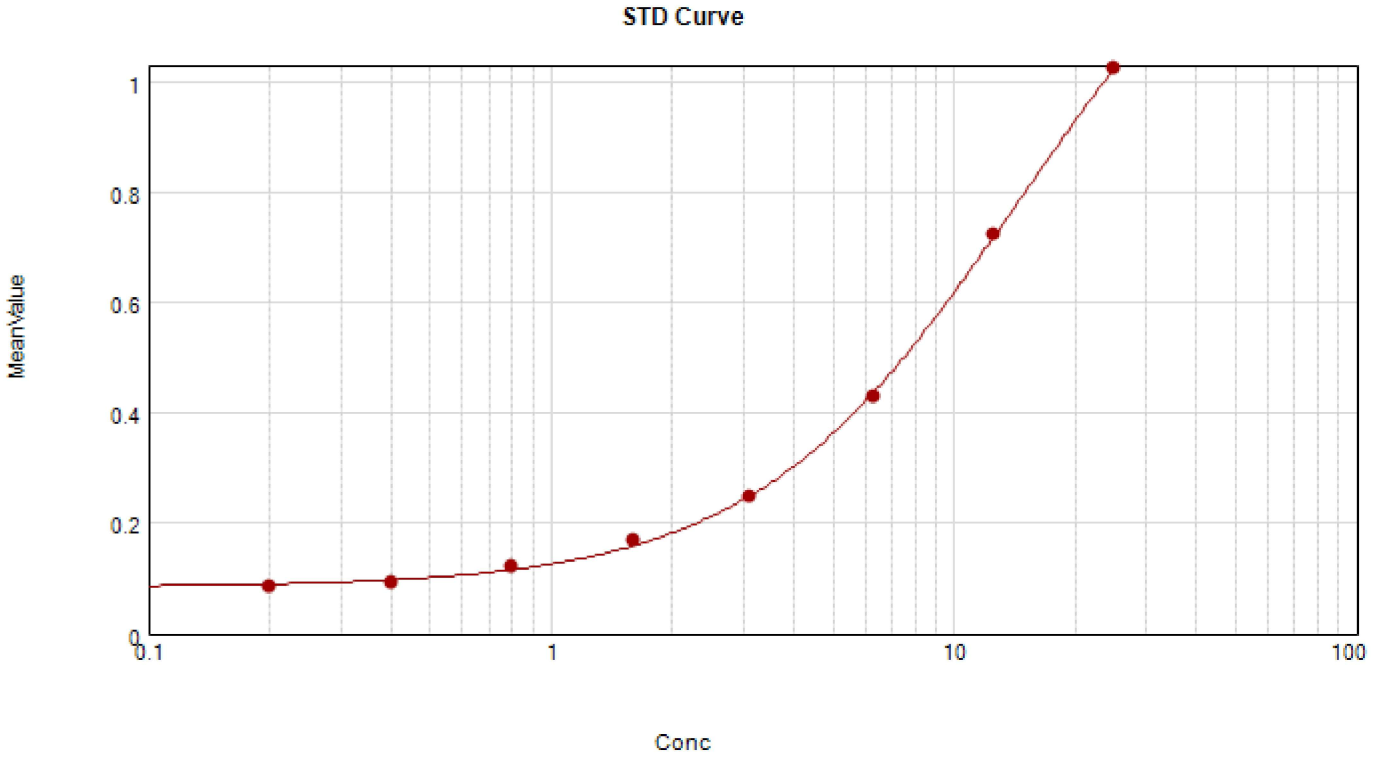

● **Std** (Standards: OD vs Th.Conc) Weighting: Fixed

Curve Fit Results ▲

Curve Fit : 4-Parameter Logistic $y = D + \frac{A - D}{1 + \left(\frac{x}{C}\right)^B}$

	Parameter	Estimated Value	Std. Error	Confidence Interval
**Std**<br>$R^2 = 1.000$<br>EC50 = 14.43	A	0.085	0.006	[0.068, 0.102]
	B	1.308	0.070	[1.115, 1.501]
	C	14.43	1.351	[10.68, 18.18]
	D	1.483	0.079	[1.263, 1.703]

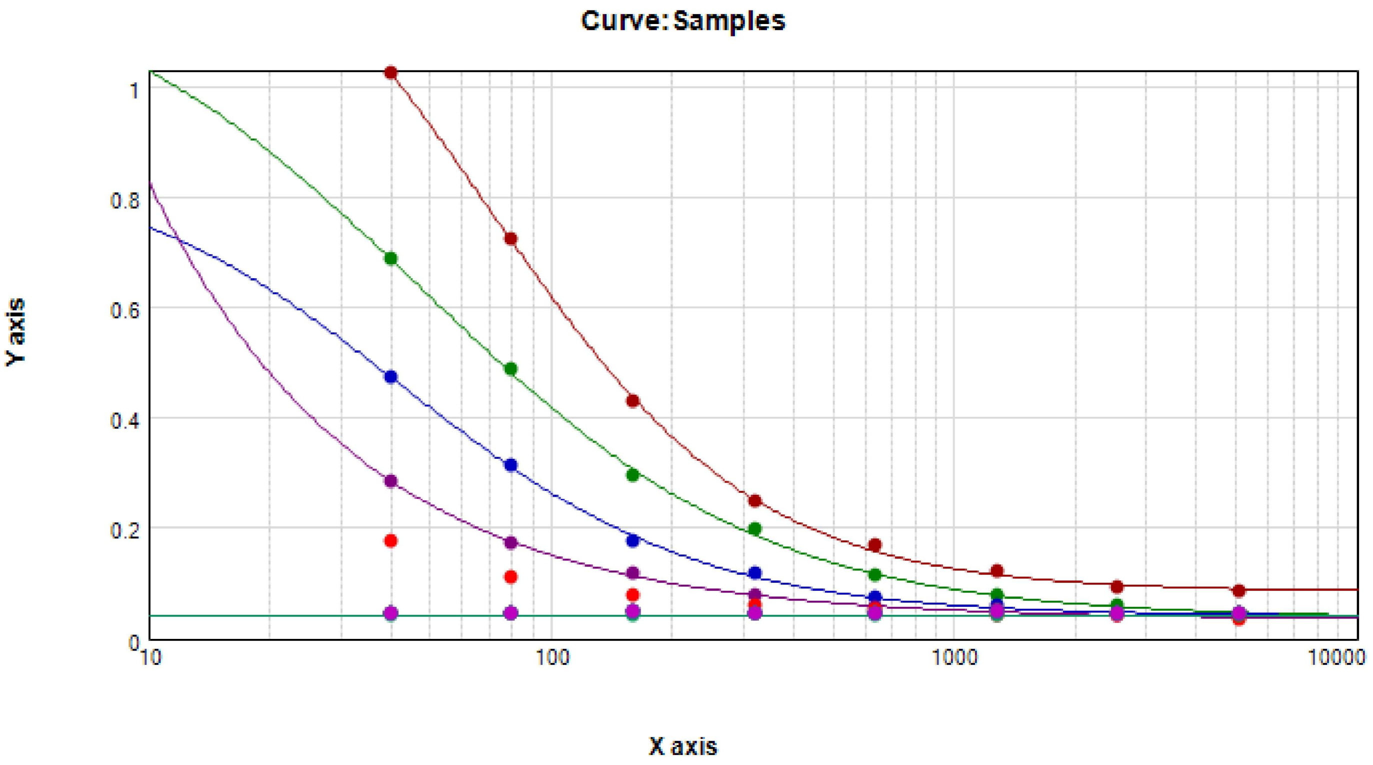

- **STD** (Standards: OD vs Dilution) Weighting: Fixed
- **S-1** (Samples: ODS1 vs DilSple1) Weighting: Fixed
- **S-2** (Samples: ODS2 vs DilSple2) Weighting: Fixed
- **S-3** (Samples: ODS3 vs DilSple3) Weighting: Fixed
- **S-4** (Samples: ODS4 vs DilSple4) Weighting: Fixed
- **S-5** (Samples: ODS5 vs DilSple5) Weighting: Fixed
- **S-6** (Samples: ODS6 vs DilSple6) Weighting: Fixed
- **S-7** (Samples: ODS7 vs DilSple7) Weighting: Fixed
- **S-8** (Samples: ODS8 vs DilSple8) Weighting: Fixed
- **S-9** (Samples: ODS9 vs DilSple9) Weighting: Fixed
- **S-10** (Samples: ODS10 vs DilSple10) Weighting: Fixed
- **S-11** (Samples: ODS11 vs DilSple11) Weighting: Fixed

Curve Fit Results ▼

Assay Parameter

Samples

Theoretical First Dilution Of Test Sample In Plate : 40.0 Sample dilution fold: 2.0

Nipha_Standard : NV-1

Concentration: 1000.0

Dilution (First dil in plate): 40.0

Dilution fold: 2.0

Others parameters

Rounding Decimal Standard Th.Conc: 1

Rounding Decimal RelErr% & CVdil: 1

Rounding Decimal GMC: 1

Average ODs of Blank: 0.046

SD of Blank: 0.002

Cutoff OD: 0.094
