# Supplementary material for: Development and Qualification of a Nipah Virus Glycoprotein-Specific IgG ELISA for the Assessment of Human Antibody Responses
Source: Vaccines (Basel). 2026 Jun 16;14(6):534. doi: 10.3390/vaccines14060534 (PMC13307770; doi:10.3390/vaccines14060534)
Supplement: Supplementary file 1 [file vaccines-14-00534-s001.zip › Supplementary_ELISA Qualification Data & Graph/4. Linearity_Analysist-2/1. Linearity_WHO IS_ANALYST-2_PLATE-1_DAY-1.pdf]

Intro

NIPAH_NIBSC_LINEARITY_ANALYST#2_PLATE#1_DAY#1

OD

	1	2	3	4	5	6	7	8	9	10	11	12
A	1.022	0.851	0.617	0.443	0.301	0.047	0.339	0.220	0.137	0.091	0.059	0.047
B	0.801	0.683	0.467	0.320	0.196	0.045	0.205	0.136	0.088	0.067	0.048	0.046
C	0.576	0.431	0.299	0.191	0.117	0.045	0.129	0.089	0.059	0.049	0.047	0.048
D	0.375	0.311	0.177	0.111	0.079	0.046	0.077	0.056	0.049	0.042	0.049	0.047
E	0.221	0.177	0.109	0.070	0.051	0.043	0.059	0.048	0.043	0.041	0.045	0.045
F	0.133	0.106	0.075	0.054	0.043	0.046	0.045	0.042	0.042	0.040	0.051	0.048
G	0.086	0.070	0.048	0.044	0.033	0.045	0.041	0.040	0.035	0.034	0.043	0.045
H	0.073	0.061	0.042	0.043	0.031	0.048	0.041	0.036	0.033	0.032	0.045	0.046

Settings Information
Endpoint
Absorbance
Lm1 450
More Settings
Shake Off
Calibrate On
Carriage Speed Normal
Column Priority

Read Information
Imported Data : 11:56 AM 10/3/2024
Imported By : anjan

Reduction Settings

Optical Density
Wavelength Combination : !Lm1

Sample Dil

Main Sample Dilution 40.0

Sample 1: NV-2 40.0

Sample 2: NV-2 (1:2) 40.0

Sample 3: NV-2 (1:4) 40.0

Sample 4: NV-2 (1:8) 40.0

Sample 5: BLANK 40.0

Sample 6: NV-6 40.0

Sample 7: NV-6 (1:2) 40.0

Sample 8: NV-6 (1:4) 40.0

Sample 9: NV-6 (1:8) 40.0

Sample 10: CNC 40.0

Sample 11: BLANK 40.0

Standards

Sample	Wells	OD	OK OD	Dilution	Calc.Conc	Adj.Conc	GMC	N	Th.Conc	RelErr%
01	A1	1.022	1.022	40	25.269	1010.8	997.7	6	25.000	1.100
	B1	0.801	0.801	80	12.259	980.7			12.500	-1.900
	C1	0.576	0.576	160	6.322	1011.6			6.300	0.400
	D1	0.375	0.375	320	3.223	1031.4			3.100	4.000
	E1	0.221	0.221	640	1.544	988.3			1.600	-3.500
	F1	0.133	0.133	1280	0.754	964.8			0.800	-5.800
	G1	0.086		2560					0.400	
	H1	0.073		5120					0.200	

Samples

Sample	Wells	ID	OD	OK OD	Dilution	Calc.Conc	Adjusted.Conc	GMC	N	CVdil
01	A2	1	0.851	0.851	40	14.253	570.126	678.6	6	11.3
	B2		0.683	0.683	80	8.676	694.064			
	C2		0.431	0.431	160	3.960	633.532			
	D2		0.311	0.311	320	2.471	790.673			
	E2		0.177	0.177	640	1.137	727.473			
	F2		0.106	0.106	1280	0.529	677.317			
	G2		0.070		2560					
	H2		0.061		5120					
02	A3	2	0.617	0.617	40	7.148	285.913	345.8	5	10.9
	B3		0.467	0.467	80	4.479	358.285			
	C3		0.299	0.299	160	2.339	374.259			
	D3		0.177	0.177	320	1.137	363.736			
	E3		0.109	0.109	640	0.554	354.412			
	F3		0.075		1280					
	G3		0.048		2560					
	H3		0.042		5120					
03	A4	3	0.443	0.443	40	4.128	165.133	188.1	4	10.2
	B4		0.320	0.320	80	2.571	205.718			
	C4		0.191	0.191	160	1.263	202.156			
	D4		0.111	0.111	320	0.570	182.472			
	E4		0.070		640					
	F4		0.054		1280					
	G4		0.044		2560					
	H4		0.043		5120					
04	A5	4	0.301	0.301	40	2.361	94.435	99.4	3	5.2
	B5		0.196	0.196	80	1.309	104.751			
	C5		0.117	0.117	160	0.620	99.170			
	D5		0.079		320					
	E5		0.051		640					
	F5		0.043		1280					
	G5		0.033		2560					
	H5		0.031		5120					
05	A6	5	0.047		40			N/A	0	-----
	B6		0.045		80					
	C6		0.045		160					
	D6		0.046		320					
	E6		0.043		640					
	F6		0.046		1280					
	G6		0.045		2560					
	H6		0.048		5120					
06	A7	6	0.339	0.339	40	2.789	111.569	112.7	3	1.9
	B7		0.205	0.205	80	1.393	111.431			
	C7		0.129	0.129	160	0.720	115.205			
	D7		0.077		320					
	E7		0.059		640					
	F7		0.045		1280					
	G7		0.041		2560					
	H7		0.041		5120					
07	A8	7	0.220	0.220	40	1.535	61.384	61.9	2	1.1
	B8		0.136	0.136	80	0.779	62.334			
	C8		0.089		160					
	D8		0.056		320					
	E8		0.048		640					
	F8		0.042		1280					
	G8		0.040		2560					
	H8		0.036		5120					
08	A9	8	0.137	0.137	40	0.788	31.507	31.5	1	-----
	B9		0.088		80					
	C9		0.059		160					
	D9		0.049		320					

Samples (Contd)

Sample	Wells	ID	OD	OK OD	Dilution	Calc.Conc	Adjusted.Conc	GMC	N	CVdil
	E9		0.043		640					
	F9		0.042		1280					
	G9		0.035		2560					
	H9		0.033		5120					
09	A10	9	0.091		40			N/A	0	-----
	B10		0.067		80					
	C10		0.049		160					
	D10		0.042		320					
	E10		0.041		640					
	F10		0.040		1280					
	G10		0.034		2560					
	H10		0.032		5120					
10	A11	10	0.059		40			N/A	0	-----
	B11		0.048		80					
	C11		0.047		160					
	D11		0.049		320					
	E11		0.045		640					
	F11		0.051		1280					
	G11		0.043		2560					
	H11		0.045		5120					
11	A12	11	0.047		40			N/A	0	-----
	B12		0.046		80					
	C12		0.048		160					
	D12		0.047		320					
	E12		0.045		640					
	F12		0.048		1280					
	G12		0.045		2560					
	H12		0.046		5120					

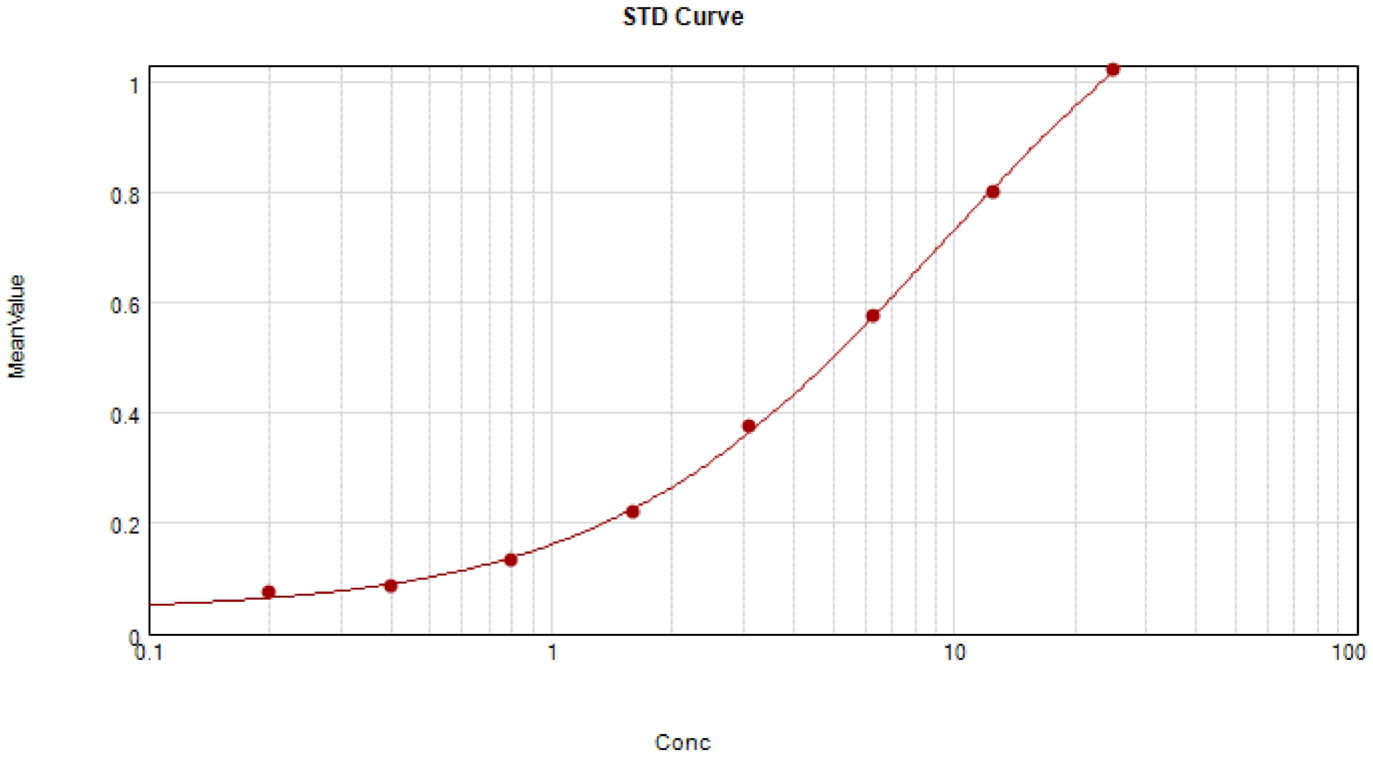

● **Std** (Standards: OD vs Th.Conc) Weighting: Fixed

Curve Fit Results ▲

Curve Fit : 4-Parameter Logistic $y = D + \frac{A - D}{1 + \left(\frac{x}{C}\right)^B}$

	Parameter	Estimated Value	Std. Error	Confidence Interval
**Std**<br>R^2 = 1.000<br>EC50 = 9.247	A	0.040	0.011	[0.011, 0.070]
	B	1.033	0.062	[0.860, 1.206]
	C	9.247	0.911	[6.718, 11.78]
	D	1.370	0.064	[1.191, 1.549]

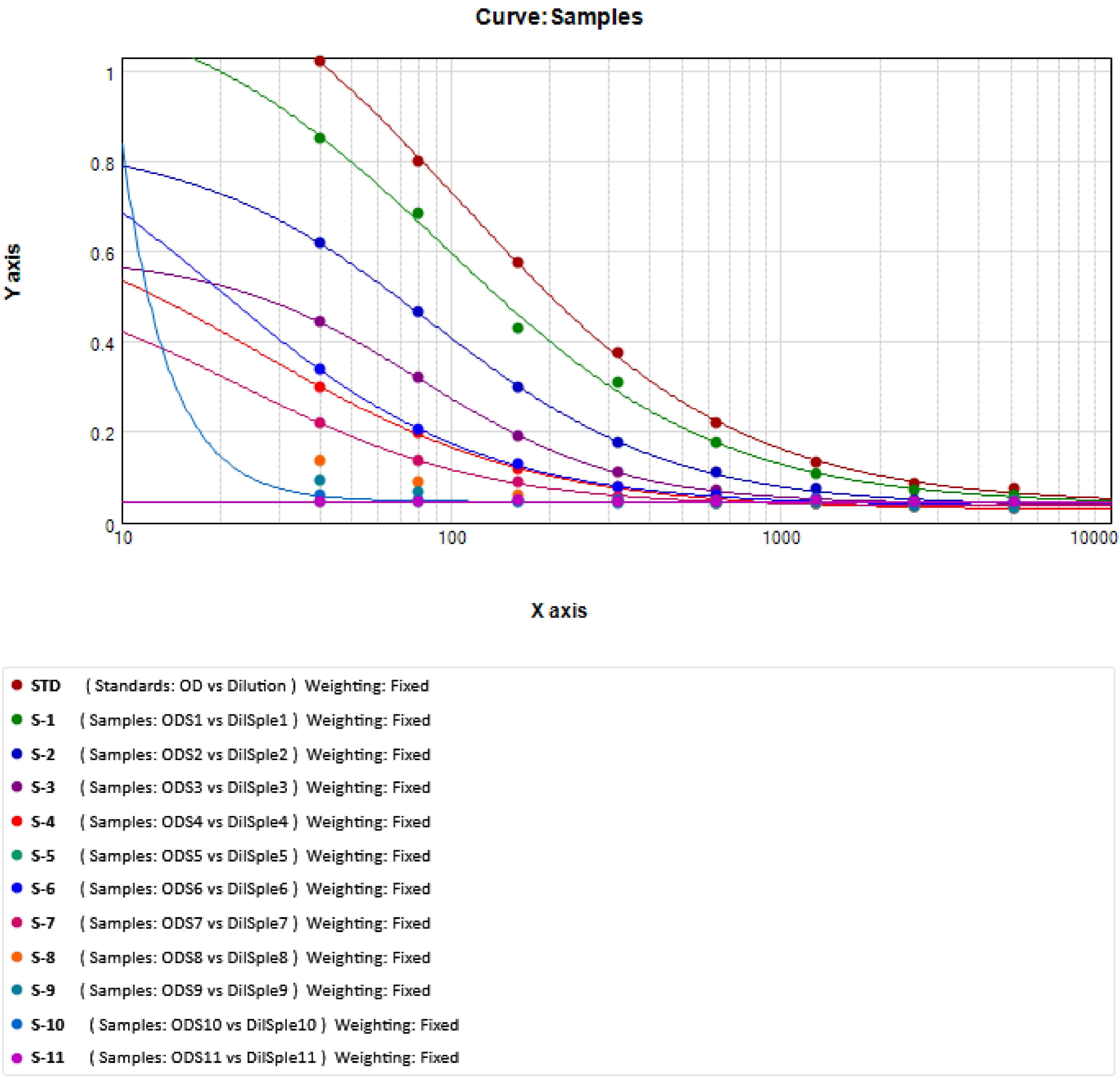

Curve Fit Results ▼

Assay Parameter

Samples

Theoretical First Dilution Of Test Sample In Plate : 40.0   Sample dilution fold: 2.0

Nipha_Standard : NV-1

Concentration: 1000.0

Dilution (First dil in plate): 40.0

Dilution fold: 2.0

Others parameters

Rounding Decimal Standard Th.Conc: 1

Rounding Decimal RelErr% & CVdil: 1

Rounding Decimal GMC: 1

Average ODs of Blank: 0.046

SD of Blank: 0.001

Cutoff OD: 0.094
