# Supplementary material for: Development and Qualification of a Nipah Virus Glycoprotein-Specific IgG ELISA for the Assessment of Human Antibody Responses
Source: Vaccines (Basel). 2026 Jun 16;14(6):534. doi: 10.3390/vaccines14060534 (PMC13307770; doi:10.3390/vaccines14060534)
Supplement: Supplementary file 1 [file vaccines-14-00534-s001.zip › Supplementary_ELISA Qualification Data & Graph/4. Linearity_Analysist-2/2. Linearity_WHO IS_ANALYST-2_PLATE-1_DAY-2.pdf]

Intro

NIPAH_NIBSC_LINEARITY_ANALYST#2_PLATE#1_DAY#2

OD

	1	2	3	4	5	6	7	8	9	10	11	12
A	1.035	0.883	0.665	0.461	0.324	0.047	0.353	0.232	0.143	0.094	0.046	0.048
B	0.824	0.687	0.476	0.328	0.210	0.044	0.221	0.136	0.093	0.065	0.045	0.049
C	0.589	0.449	0.309	0.197	0.126	0.043	0.133	0.095	0.072	0.049	0.045	0.045
D	0.384	0.318	0.185	0.117	0.083	0.045	0.087	0.064	0.052	0.047	0.047	0.048
E	0.228	0.181	0.111	0.072	0.055	0.040	0.059	0.049	0.045	0.045	0.046	0.049
F	0.141	0.110	0.074	0.053	0.048	0.046	0.047	0.045	0.044	0.043	0.040	0.045
G	0.094	0.078	0.057	0.047	0.042	0.045	0.043	0.036	0.038	0.041	0.041	0.049
H	0.087	0.064	0.045	0.044	0.041	0.042	0.039	0.034	0.036	0.040	0.042	0.048

Settings Information
Endpoint
Absorbance
Lm1 450
More Settings
Shake Off
Calibrate On
Carriage Speed Normal
Column Priority

Read Information
Imported Data : 11:58 AM 10/3/2024
Imported By : anjan

Reduction Settings

Optical Density
Wavelength Combination : !Lm1

Sample Dil

Main Sample Dilution 40.0

Sample 1: NV-2 40.0

Sample 2: NV-2 (1:2) 40.0

Sample 3: NV-2 (1:4) 40.0

Sample 4: NV-2 (1:8) 40.0

Sample 5: BLANK 40.0

Sample 6: NV-6 40.0

Sample 7: NV-6 (1:2) 40.0

Sample 8: NV-6 (1:4) 40.0

Sample 9: NV-6 (1:8) 40.0

Sample 10: CNC 40.0

Sample 11: BLANK 40.0

Standards

Sample	Wells	OD	OK OD	Dilution	Calc.Conc	Adj.Conc	GMC	N	Th.Conc	RelErr%
01	A1	1.035	1.035	40	25.200	1008.0	999.1	6	25.000	0.800
	B1	0.824	0.824	80	12.362	989.0			12.500	-1.100
	C1	0.589	0.589	160	6.265	1002.5			6.300	-0.500
	D1	0.384	0.384	320	3.223	1031.3			3.100	4.000
	E1	0.228	0.228	640	1.554	994.8			1.600	-2.900
	F1	0.141	0.141	1280	0.758	970.2			0.800	-5.300
	G1	0.094		2560					0.400	
	H1	0.087		5120					0.200	

Samples

Sample	Wells	ID	OD	OK OD	Dilution	Calc.Conc	Adjusted.Conc	GMC	N	CVdil
01	A2	1	0.883	0.883	40	14.803	592.139	668.9	6	10.4
	B2		0.687	0.687	80	8.313	665.024			
	C2		0.449	0.449	160	4.055	648.785			
	D2		0.318	0.318	320	2.469	790.017			
	E2		0.181	0.181	640	1.116	714.364			
	F2		0.110	0.110	1280	0.485	621.284			
	G2		0.078		2560					
	H2		0.064		5120					
02	A3	2	0.665	0.665	40	7.806	312.236	345.2	5	8.9
	B3		0.476	0.476	80	4.432	354.550			
	C3		0.309	0.309	160	2.372	379.520			
	D3		0.185	0.185	320	1.153	368.853			
	E3		0.111	0.111	640	0.494	316.261			
	F3		0.074		1280					
	G3		0.057		2560					
	H3		0.045		5120					
03	A4	3	0.461	0.461	40	4.220	168.799	187.3	4	10.1
	B4		0.328	0.328	80	2.578	206.235			
	C4		0.197	0.197	160	1.263	202.068			
	D4		0.117	0.117	320	0.547	174.980			
	E4		0.072		640					
	F4		0.053		1280					
	G4		0.047		2560					
	H4		0.044		5120					
04	A5	4	0.324	0.324	40	2.534	101.363	104.0	3	5.5
	B5		0.210	0.210	80	1.384	110.713			
	C5		0.126	0.126	160	0.626	100.132			
	D5		0.083		320					
	E5		0.055		640					
	F5		0.048		1280					
	G5		0.042		2560					
	H5		0.041		5120					
05	A6	5	0.047		40			N/A	0	-----
	B6		0.044		80					
	C6		0.043		160					
	D6		0.045		320					
	E6		0.040		640					
	F6		0.046		1280					
	G6		0.045		2560					
	H6		0.042		5120					
06	A7	6	0.353	0.353	40	2.858	114.339	114.4	3	3.9
	B7		0.221	0.221	80	1.488	119.012			
	C7		0.133	0.133	160	0.687	109.983			
	D7		0.087		320					
	E7		0.059		640					
	F7		0.047		1280					
	G7		0.043		2560					
	H7		0.039		5120					
07	A8	7	0.232	0.232	40	1.593	63.709	60.3	2	7.7
	B8		0.136	0.136	80	0.714	57.106			
	C8		0.095		160					
	D8		0.064		320					
	E8		0.049		640					
	F8		0.045		1280					
	G8		0.036		2560					
	H8		0.034		5120					
08	A9	8	0.143	0.143	40	0.776	31.026	31.0	1	-----
	B9		0.093		80					
	C9		0.072		160					
	D9		0.052		320					

Samples (Contd)

Sample	Wells	ID	OD	OK OD	Dilution	Calc.Conc	Adjusted.Conc	GMC	N	CVdil
	E9		0.045		640					
	F9		0.044		1280					
	G9		0.038		2560					
	H9		0.036		5120					
09	A10	9	0.094		40			N/A	0	-----
	B10		0.065		80					
	C10		0.049		160					
	D10		0.047		320					
	E10		0.045		640					
	F10		0.043		1280					
	G10		0.041		2560					
	H10		0.040		5120					
10	A11	10	0.046		40			N/A	0	-----
	B11		0.045		80					
	C11		0.045		160					
	D11		0.047		320					
	E11		0.046		640					
	F11		0.040		1280					
	G11		0.041		2560					
	H11		0.042		5120					
11	A12	11	0.048		40			N/A	0	-----
	B12		0.049		80					
	C12		0.045		160					
	D12		0.048		320					
	E12		0.049		640					
	F12		0.045		1280					
	G12		0.049		2560					
	H12		0.048		5120					

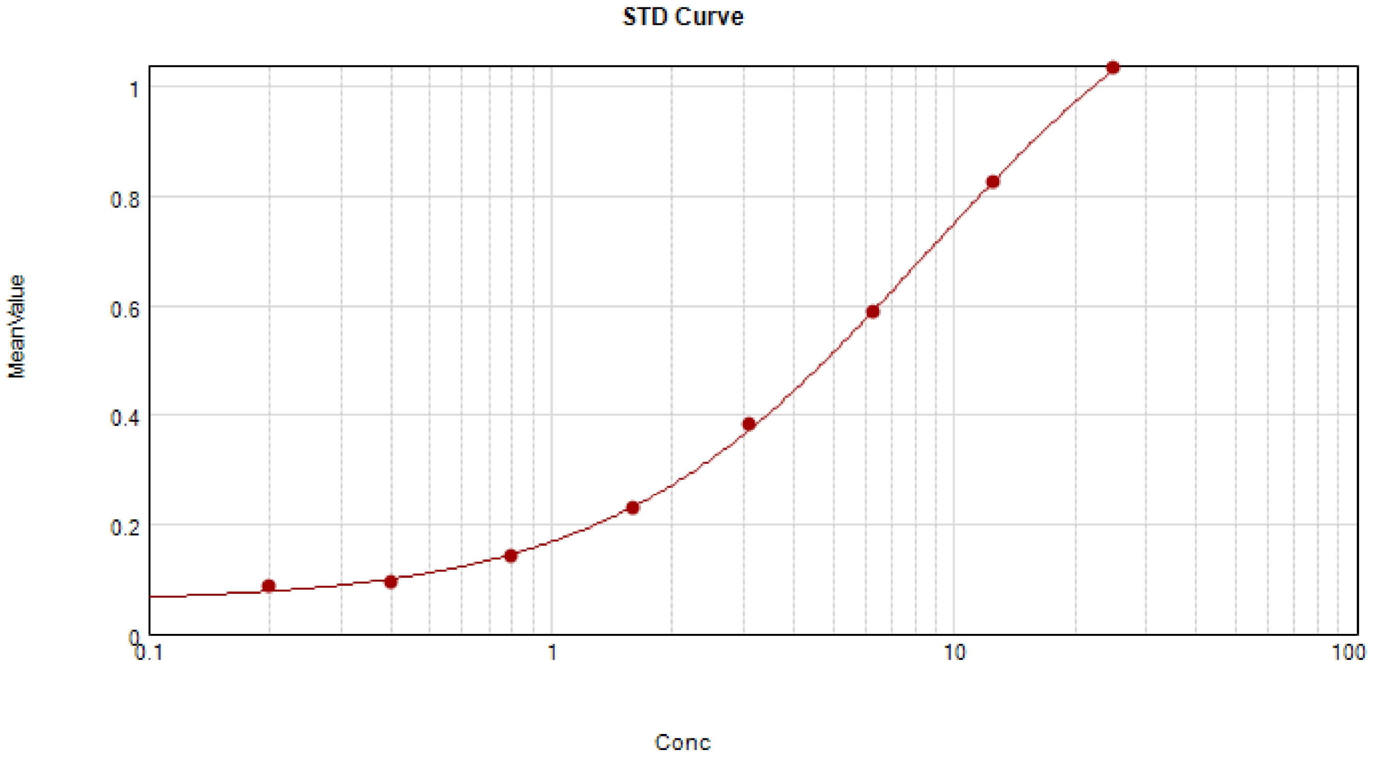

● **Std** (Standards: OD vs Th.Conc) Weighting: Fixed

Curve Fit Results ▲

Curve Fit : 4-Parameter Logistic $y = D + \frac{A - D}{1 + \left(\frac{x}{C}\right)^B}$

	Parameter	Estimated Value	Std. Error	Confidence Interval
**Std**<br>R^2 = 1.000<br>EC50 = 8.465	A	0.058	0.010	[0.031, 0.085]
	B	1.101	0.062	[0.929, 1.273]
	C	8.465	0.687	[6.558, 10.37]
	D	1.329	0.053	[1.183, 1.475]

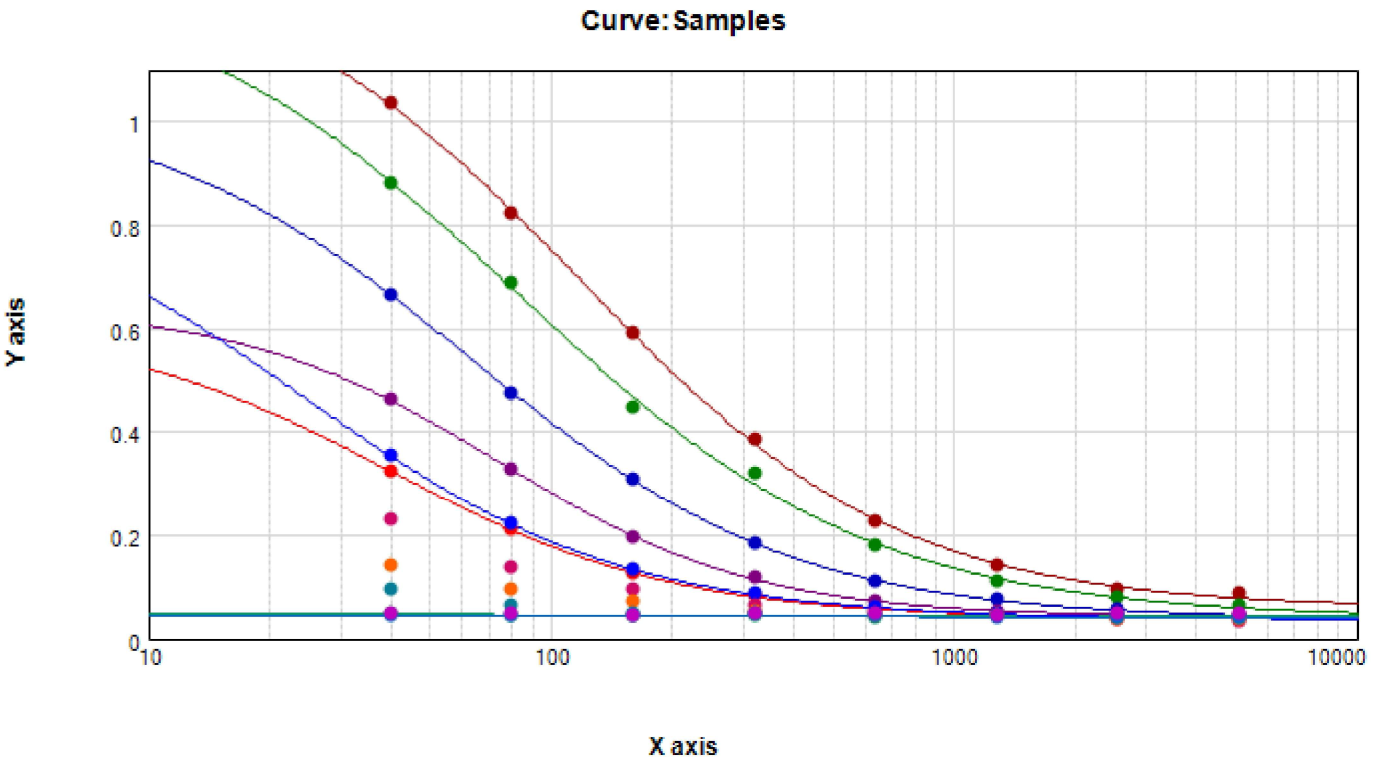

- STD (Standards: OD vs Dilution) Weighting: Fixed
- S-1 (Samples: ODS1 vs DilSple1) Weighting: Fixed
- S-2 (Samples: ODS2 vs DilSple2) Weighting: Fixed
- S-3 (Samples: ODS3 vs DilSple3) Weighting: Fixed
- S-4 (Samples: ODS4 vs DilSple4) Weighting: Fixed
- S-5 (Samples: ODS5 vs DilSple5) Weighting: Fixed
- S-6 (Samples: ODS6 vs DilSple6) Weighting: Fixed
- S-7 (Samples: ODS7 vs DilSple7) Weighting: Fixed
- S-8 (Samples: ODS8 vs DilSple8) Weighting: Fixed
- S-9 (Samples: ODS9 vs DilSple9) Weighting: Fixed
- S-10 (Samples: ODS10 vs DilSple10) Weighting: Fixed
- S-11 (Samples: ODS11 vs DilSple11) Weighting: Fixed

Curve Fit Results ▼

Assay Parameter

Samples

Theoretical First Dilution Of Test Sample In Plate : 40.0 Sample dilution fold: 2.0

Nipha_Standard : NV-1

Concentration: 1000.0

Dilution (First dil in plate): 40.0

Dilution fold: 2.0

Others parameters

Rounding Decimal Standard Th.Conc: 1

Rounding Decimal RelErr% & CVdil: 1

Rounding Decimal GMC: 1

Average ODs of Blank: 0.048

SD of Blank: 0.002

Cutoff OD: 0.097
