# Supplementary material for: Development and Qualification of a Nipah Virus Glycoprotein-Specific IgG ELISA for the Assessment of Human Antibody Responses
Source: Vaccines (Basel). 2026 Jun 16;14(6):534. doi: 10.3390/vaccines14060534 (PMC13307770; doi:10.3390/vaccines14060534)
Supplement: Supplementary file 1 [file vaccines-14-00534-s001.zip › Supplementary_ELISA Qualification Data & Graph/4. Linearity_Analysist-2/3. Linearity_WHO IS_ANALYST-2_PLATE-1_DAY-3.pdf]

Intro

NIPAH_NIBSC_LINEARITY_ANALYST#2_PLATE#1_DAY#3

OD

	1	2	3	4	5	6	7	8	9	10	11	12
A	1.037	0.879	0.657	0.465	0.316	0.045	0.356	0.239	0.148	0.093	0.051	0.043
B	0.827	0.691	0.484	0.341	0.210	0.043	0.224	0.144	0.101	0.075	0.049	0.051
C	0.589	0.449	0.315	0.205	0.123	0.048	0.143	0.094	0.076	0.064	0.049	0.049
D	0.380	0.313	0.187	0.123	0.088	0.049	0.088	0.075	0.056	0.054	0.045	0.046
E	0.227	0.189	0.120	0.085	0.064	0.047	0.067	0.058	0.052	0.052	0.057	0.043
F	0.139	0.110	0.087	0.063	0.052	0.047	0.055	0.052	0.048	0.047	0.043	0.046
G	0.093	0.085	0.069	0.055	0.045	0.042	0.047	0.045	0.046	0.044	0.045	0.042
H	0.087	0.076	0.053	0.045	0.043	0.043	0.041	0.040	0.041	0.039	0.044	0.047

Settings Information
Endpoint
Absorbance
Lm1 450
More Settings
Shake Off
Calibrate On
Carriage Speed Normal
Column Priority

Read Information
Imported Data : 11:59 AM 10/3/2024
Imported By : anjan

Reduction Settings

Optical Density
Wavelength Combination : !Lm1

Sample Dil

Main Sample Dilution 40.0

Sample 1: NV-2 40.0

Sample 2: NV-2 (1:2) 40.0

Sample 3: NV-2 (1:4) 40.0

Sample 4: NV-2 (1:8) 40.0

Sample 5: BLANK 40.0

Sample 6: NV-6 40.0

Sample 7: NV-6 (1:2) 40.0

Sample 8: NV-6 (1:4) 40.0

Sample 9: NV-6 (1:8) 40.0

Sample 10: CNC 40.0

Sample 11: BLANK 40.0

Standards

Sample	Wells	OD	OK OD	Dilution	Calc.Conc	Adj.Conc	GMC	N	Th.Conc	RelErr%
01	A1	1.037	1.037	40	25.170	1006.8	999.5	6	25.000	0.700
	B1	0.827	0.827	80	12.389	991.1			12.500	-0.900
	C1	0.589	0.589	160	6.267	1002.6			6.300	-0.500
	D1	0.380	0.380	320	3.202	1024.6			3.100	3.300
	E1	0.227	0.227	640	1.571	1005.2			1.600	-1.800
	F1	0.139	0.139	1280	0.756	967.6			0.800	-5.500
	G1	0.093		2560					0.400	
	H1	0.087		5120					0.200	

Samples

Sample	Wells	ID	OD	OK OD	Dilution	Calc.Conc	Adjusted.Conc	GMC	N	CVdil
01	A2	1	0.879	0.879	40	14.504	580.142	678.4	6	11.8
	B2		0.691	0.691	80	8.382	670.567			
	C2		0.449	0.449	160	4.077	652.356			
	D2		0.313	0.313	320	2.444	781.933			
	E2		0.189	0.189	640	1.212	775.586			
	F2		0.110	0.110	1280	0.495	633.547			
	G2		0.085		2560					
	H2		0.076		5120					
02	A3	2	0.657	0.657	40	7.613	304.528	362.7	5	10.2
	B3		0.484	0.484	80	4.566	365.284			
	C3		0.315	0.315	160	2.465	394.411			
	D3		0.187	0.187	320	1.193	381.862			
	E3		0.120	0.120	640	0.585	374.438			
	F3		0.087		1280					
	G3		0.069		2560					
	H3		0.053		5120					
03	A4	3	0.465	0.465	40	4.297	171.862	200.4	4	11.6
	B4		0.341	0.341	80	2.751	220.067			
	C4		0.205	0.205	160	1.361	217.810			
	D4		0.123	0.123	320	0.612	195.853			
	E4		0.085		640					
	F4		0.063		1280					
	G4		0.055		2560					
	H4		0.045		5120					
04	A5	4	0.316	0.316	40	2.476	99.034	103.0	3	7.8
	B5		0.210	0.210	80	1.408	112.678			
	C5		0.123	0.123	160	0.612	97.927			
	D5		0.088		320					
	E5		0.064		640					
	F5		0.052		1280					
	G5		0.045		2560					
	H5		0.043		5120					
05	A6	5	0.045		40			N/A	0	-----
	B6		0.043		80					
	C6		0.048		160					
	D6		0.049		320					
	E6		0.047		640					
	F6		0.047		1280					
	G6		0.042		2560					
	H6		0.043		5120					
06	A7	6	0.356	0.356	40	2.921	116.839	122.2	3	4.1
	B7		0.224	0.224	80	1.542	123.345			
	C7		0.143	0.143	160	0.792	126.720			
	D7		0.088		320					
	E7		0.067		640					
	F7		0.055		1280					
	G7		0.047		2560					
	H7		0.041		5120					
07	A8	7	0.239	0.239	40	1.687	67.476	65.8	2	3.7
	B8		0.144	0.144	80	0.801	64.082			
	C8		0.094		160					
	D8		0.075		320					
	E8		0.058		640					
	F8		0.052		1280					
	G8		0.045		2560					
	H8		0.040		5120					
08	A9	8	0.148	0.148	40	0.837	33.485	33.3	2	0.9
	B9		0.101	0.101	80	0.413	33.076			
	C9		0.076		160					
	D9		0.056		320					

Samples (Contd)

Sample	Wells	ID	OD	OK OD	Dilution	Calc.Conc	Adjusted.Conc	GMC	N	CVdil
	E9		0.052		640					
	F9		0.048		1280					
	G9		0.046		2560					
	H9		0.041		5120					
09	A10	9	0.093		40			N/A	0	-----
	B10		0.075		80					
	C10		0.064		160					
	D10		0.054		320					
	E10		0.052		640					
	F10		0.047		1280					
	G10		0.044		2560					
	H10		0.039		5120					
10	A11	10	0.051		40			N/A	0	-----
	B11		0.049		80					
	C11		0.049		160					
	D11		0.045		320					
	E11		0.057		640					
	F11		0.043		1280					
	G11		0.045		2560					
	H11		0.044		5120					
11	A12	11	0.043		40			N/A	0	-----
	B12		0.051		80					
	C12		0.049		160					
	D12		0.046		320					
	E12		0.043		640					
	F12		0.046		1280					
	G12		0.042		2560					
	H12		0.047		5120					

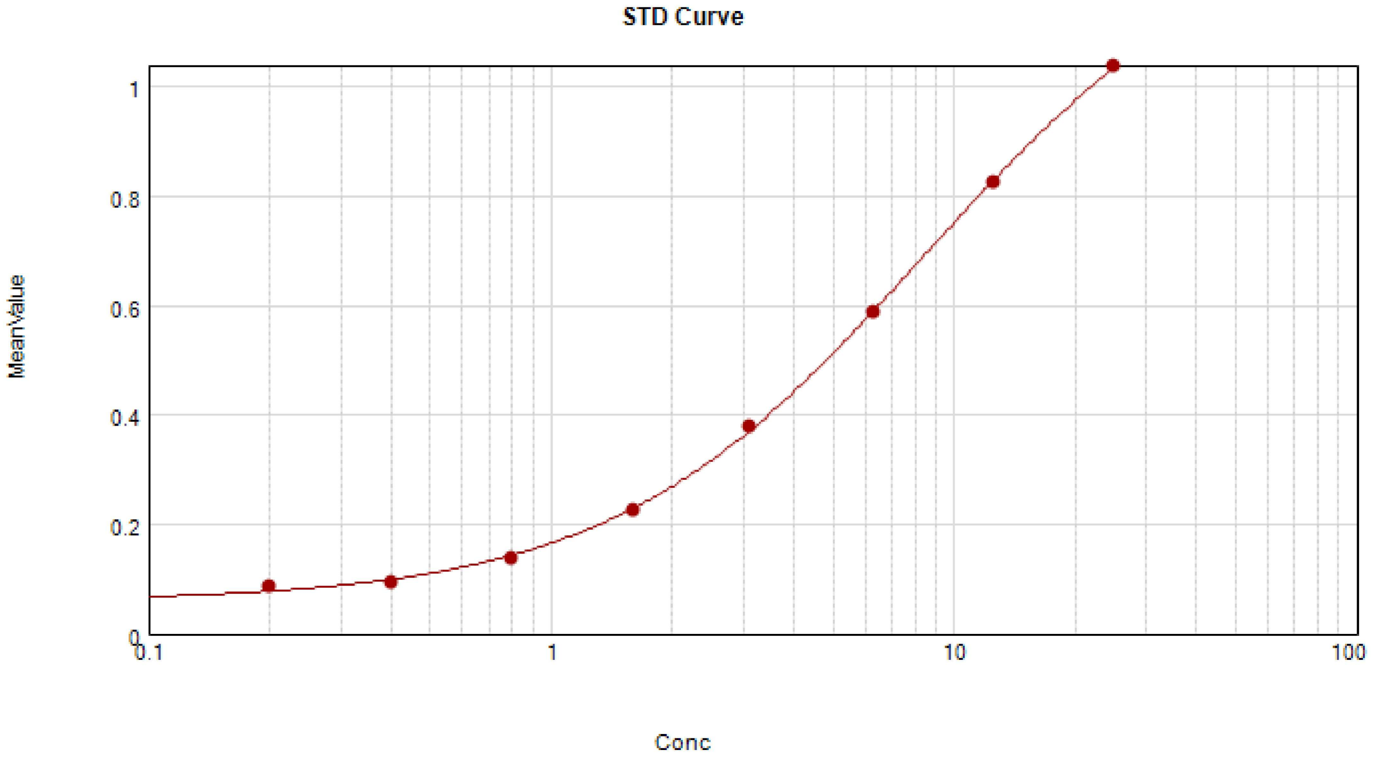

● **Std** (Standards: OD vs Th.Conc) Weighting: Fixed

Curve Fit Results ▲

Curve Fit : 4-Parameter Logistic $y = D + \frac{A - D}{1 + \left(\frac{x}{C}\right)^B}$

	Parameter	Estimated Value	Std. Error	Confidence Interval
**Std**<br>R^2 = 1.000<br>EC50 = 8.367	A	0.059	0.009	[0.035, 0.083]
	B	1.121	0.056	[0.964, 1.277]
	C	8.367	0.593	[6.719, 10.01]
	D	1.322	0.046	[1.193, 1.450]

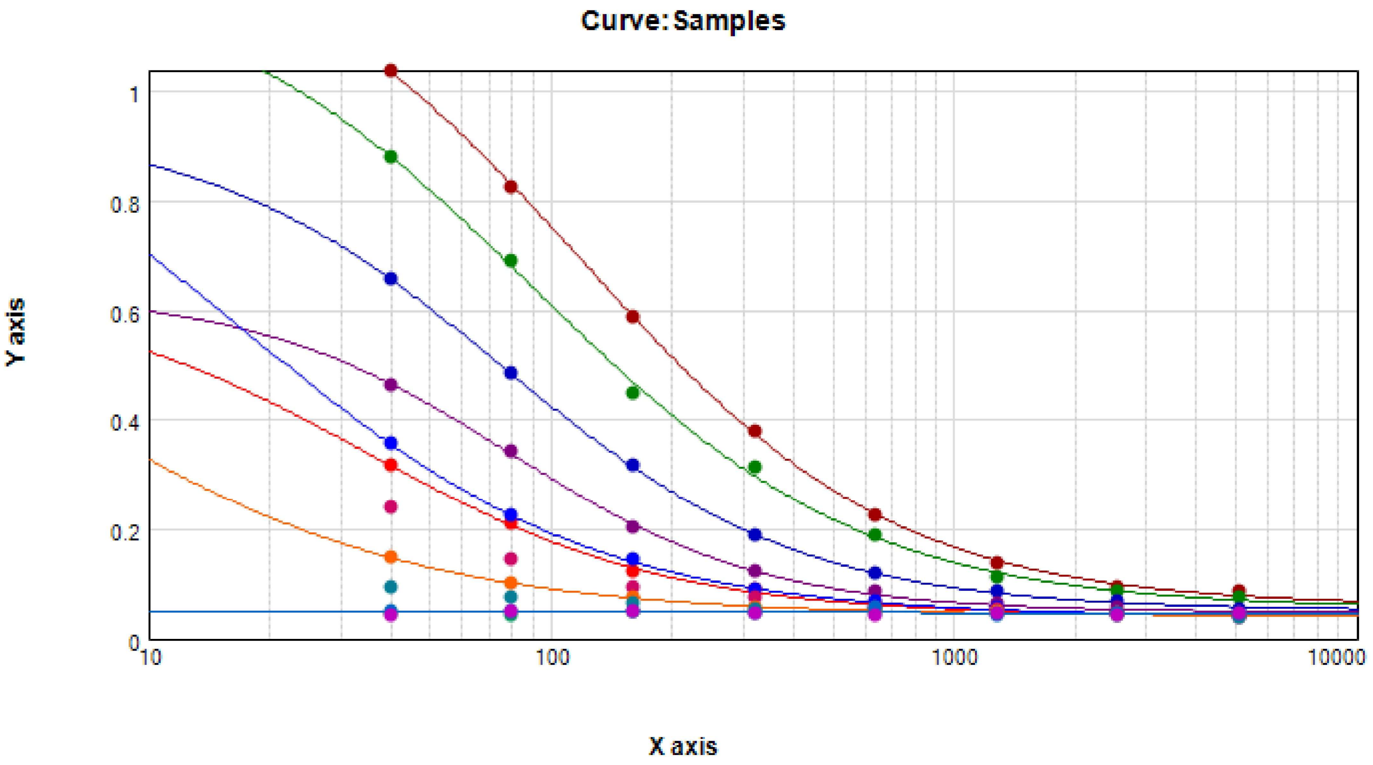

- STD (Standards: OD vs Dilution) Weighting: Fixed
- S-1 (Samples: ODS1 vs DilSple1) Weighting: Fixed
- S-2 (Samples: ODS2 vs DilSple2) Weighting: Fixed
- S-3 (Samples: ODS3 vs DilSple3) Weighting: Fixed
- S-4 (Samples: ODS4 vs DilSple4) Weighting: Fixed
- S-5 (Samples: ODS5 vs DilSple5) Weighting: Fixed
- S-6 (Samples: ODS6 vs DilSple6) Weighting: Fixed
- S-7 (Samples: ODS7 vs DilSple7) Weighting: Fixed
- S-8 (Samples: ODS8 vs DilSple8) Weighting: Fixed
- S-9 (Samples: ODS9 vs DilSple9) Weighting: Fixed
- S-10 (Samples: ODS10 vs DilSple10) Weighting: Fixed
- S-11 (Samples: ODS11 vs DilSple11) Weighting: Fixed

Curve Fit Results ▼

Assay Parameter

Samples

Theoretical First Dilution Of Test Sample In Plate : 40.0   Sample dilution fold: 2.0

Nipha_Standard : NV-1

Concentration: 1000.0

Dilution (First dil in plate): 40.0

Dilution fold: 2.0

Others parameters

Rounding Decimal Standard Th.Conc: 1

Rounding Decimal RelErr% & CVdil: 1

Rounding Decimal GMC: 1

Average ODs of Blank: 0.046

SD of Blank: 0.003

Cutoff OD: 0.095
