# Supplementary material for: Development and Qualification of a Nipah Virus Glycoprotein-Specific IgG ELISA for the Assessment of Human Antibody Responses
Source: Vaccines (Basel). 2026 Jun 16;14(6):534. doi: 10.3390/vaccines14060534 (PMC13307770; doi:10.3390/vaccines14060534)
Supplement: Supplementary file 1 [file vaccines-14-00534-s001.zip › Supplementary_ELISA Qualification Data & Graph/4. Linearity_Analysist-2/4. Linearity_WHO IS_ANALYST-2_PLATE-2_DAY-1.pdf]

Intro

NIPAH_NIBSC_LINEARITY_ANALYST#2_PLATE#2_DAY#1

OD

	1	2	3	4	5	6	7	8	9	10	11	12
A	1.011	0.688	0.473	0.292	0.173	0.048	0.048	0.048	0.048	0.048	0.048	0.048
B	0.725	0.494	0.309	0.176	0.140	0.048	0.045	0.045	0.045	0.045	0.045	0.045
C	0.469	0.303	0.179	0.116	0.074	0.047	0.046	0.046	0.046	0.046	0.046	0.046
D	0.287	0.185	0.109	0.077	0.058	0.044	0.048	0.048	0.048	0.048	0.048	0.048
E	0.168	0.109	0.078	0.055	0.052	0.045	0.045	0.045	0.045	0.045	0.045	0.045
F	0.108	0.079	0.063	0.054	0.046	0.042	0.047	0.047	0.047	0.047	0.047	0.047
G	0.091	0.055	0.047	0.044	0.044	0.048	0.047	0.047	0.047	0.047	0.047	0.047
H	0.082	0.046	0.036	0.039	0.041	0.044	0.045	0.045	0.045	0.045	0.045	0.045

Settings Information

Endpoint

Absorbance

Lm1 450

More Settings

Shake Off

Calibrate On

Carriage Speed Normal

Column Priority

Read Information

Imported Data : 12:02 PM 10/3/2024

Imported By : anjan

Reduction Settings

Optical Density

Wavelength Combination : !Lm1

Sample Dil

Main Sample Dilution 40.0

Sample 1: NV-4 40.0

Sample 2: NV-4 (1:2) 40.0

Sample 3: NV-4 (1:4) 40.0

Sample 4: NV-4 (1:8) 40.0

Sample 5: CNC 40.0

Sample 6: BLANK 40.0

Sample 7: BLANK 40.0

Sample 8: BLANK 40.0

Sample 9: BLANK 40.0

Sample 10: BLANK 40.0

Sample 11: BLANK 40.0

Standards

Sample	Wells	OD	OK OD	Dilution	Calc.Conc	Adj.Conc	GMC	N	Th.Conc	RelErr%
01	A1	1.011	1.011	40	25.078	1003.1	977.1	6	25.000	0.300
	B1	0.725	0.725	80	12.402	992.2			12.500	-0.800
	C1	0.469	0.469	160	6.307	1009.1			6.300	0.100
	D1	0.287	0.287	320	3.234	1035.0			3.100	4.300
	E1	0.168	0.168	640	1.515	969.7			1.600	-5.300
	F1	0.108	0.108	1280	0.674	863.2			0.800	-15.700
	G1	0.091		2560					0.400	
	H1	0.082		5120					0.200	

Samples

Sample	Wells	ID	OD	OK OD	Dilution	Calc.Conc	Adjusted.Conc	GMC	N	CVdil
01	A2	1	0.688	0.688	40	11.329	453.166	508.2	5	11.9
	B2		0.494	0.494	80	6.791	543.268			
	C2		0.303	0.303	160	3.479	556.596			
	D2		0.185	0.185	320	1.753	561.028			
	E2		0.109	0.109	640	0.689	440.724			
	F2		0.079		1280					
	G2		0.055		2560					
	H2		0.046		5120					
02	A3	2	0.473	0.473	40	6.383	255.314	256.0	4	11.0
	B3		0.309	0.309	80	3.571	285.711			
	C3		0.179	0.179	160	1.669	267.047			
	D3		0.109	0.109	320	0.689	220.362			
	E3		0.078		640					
	F3		0.063		1280					
	G3		0.047		2560					
	H3		0.036		5120					
03	A4	3	0.292	0.292	40	3.310	132.413	129.5	3	2.5
	B4		0.176	0.176	80	1.627	130.162			
	C4		0.116	0.116	160	0.788	126.083			
	D4		0.077		320					
	E4		0.055		640					
	F4		0.054		1280					
	G4		0.044		2560					
	H4		0.039		5120					
04	A5	4	0.173	0.173	40	1.585	63.402	75.5	2	25.1
	B5		0.140	0.140	80	1.125	89.975			
	C5		0.074		160					
	D5		0.058		320					
	E5		0.052		640					
	F5		0.046		1280					
	G5		0.044		2560					
	H5		0.041		5120					
05	A6	5	0.048		40			N/A	0	-----
	B6		0.048		80					
	C6		0.047		160					
	D6		0.044		320					
	E6		0.045		640					
	F6		0.042		1280					
	G6		0.048		2560					
	H6		0.044		5120					
06	A7	6	0.048		40			N/A	0	-----
	B7		0.045		80					
	C7		0.046		160					
	D7		0.048		320					
	E7		0.045		640					
	F7		0.047		1280					
	G7		0.047		2560					
	H7		0.045		5120					
07	A8	7	0.048		40			N/A	0	-----
	B8		0.045		80					
	C8		0.046		160					
	D8		0.048		320					
	E8		0.045		640					
	F8		0.047		1280					
	G8		0.047		2560					
	H8		0.045		5120					
08	A9	8	0.048		40			N/A	0	-----
	B9		0.045		80					
	C9		0.046		160					
	D9		0.048		320					

Samples (Contd)

Sample	Wells	ID	OD	OK OD	Dilution	Calc.Conc	Adjusted.Conc	GMC	N	CVdil
	E9		0.045		640					
	F9		0.047		1280					
	G9		0.047		2560					
	H9		0.045		5120					
09	A10	9	0.048		40			N/A	0	-----
	B10		0.045		80					
	C10		0.046		160					
	D10		0.048		320					
	E10		0.045		640					
	F10		0.047		1280					
	G10		0.047		2560					
	H10		0.045		5120					
10	A11	10	0.048		40			N/A	0	-----
	B11		0.045		80					
	C11		0.046		160					
	D11		0.048		320					
	E11		0.045		640					
	F11		0.047		1280					
	G11		0.047		2560					
	H11		0.045		5120					
11	A12	11	0.048		40			N/A	0	-----
	B12		0.045		80					
	C12		0.046		160					
	D12		0.048		320					
	E12		0.045		640					
	F12		0.047		1280					
	G12		0.047		2560					
	H12		0.045		5120					

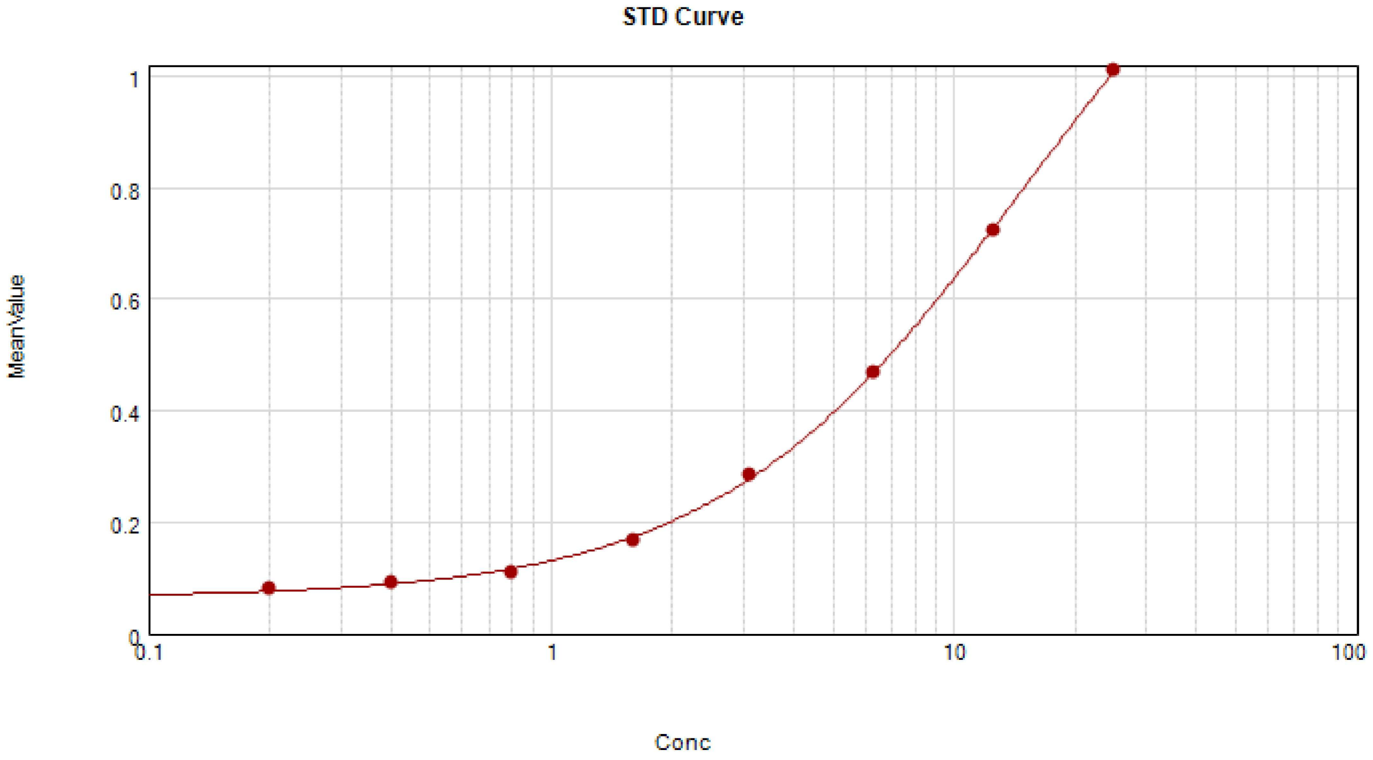

● **Std** (Standards: OD vs Th.Conc) Weighting: Fixed

Curve Fit Results ▲

Curve Fit : 4-Parameter Logistic $y = D + \frac{A - D}{1 + \left(\frac{x}{C}\right)^{B}}$

	Parameter	Estimated Value	Std. Error	Confidence Interval
**Std** $R^2 = 1.000$ EC50 = 14.79	A	0.065	0.007	[0.044, 0.086]
	B	1.134	0.065	[0.953, 1.314]
	C	14.79	1.766	[9.890, 19.70]
	D	1.531	0.095	[1.266, 1.795]

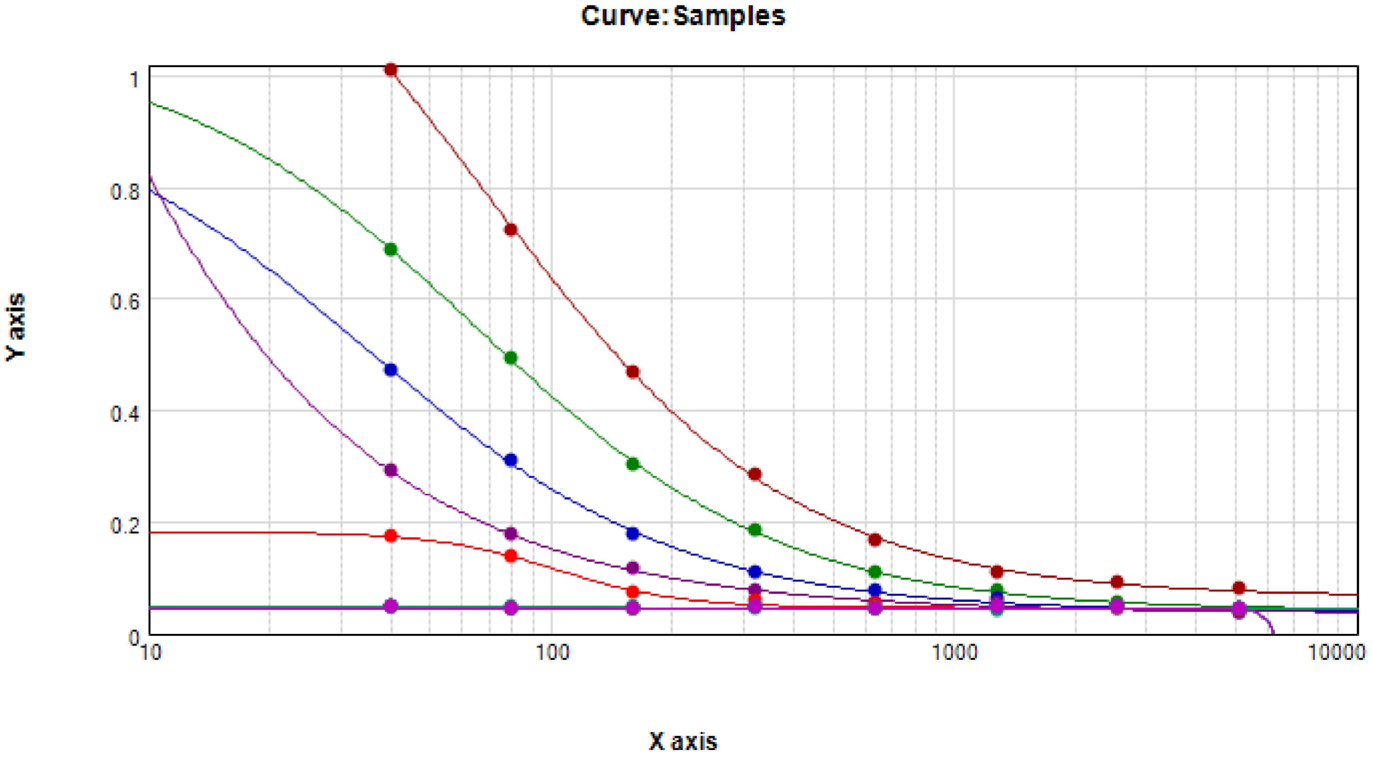

- **STD** (Standards: OD vs Dilution) Weighting: Fixed
- **S-1** (Samples: ODS1 vs DilSple1) Weighting: Fixed
- **S-2** (Samples: ODS2 vs DilSple2) Weighting: Fixed
- **S-3** (Samples: ODS3 vs DilSple3) Weighting: Fixed
- **S-4** (Samples: ODS4 vs DilSple4) Weighting: Fixed
- **S-5** (Samples: ODS5 vs DilSple5) Weighting: Fixed
- **S-6** (Samples: ODS6 vs DilSple6) Weighting: Fixed
- **S-7** (Samples: ODS7 vs DilSple7) Weighting: Fixed
- **S-8** (Samples: ODS8 vs DilSple8) Weighting: Fixed
- **S-9** (Samples: ODS9 vs DilSple9) Weighting: Fixed
- **S-10** (Samples: ODS10 vs DilSple10) Weighting: Fixed
- **S-11** (Samples: ODS11 vs DilSple11) Weighting: Fixed

Curve Fit Results ▼

Assay Parameter

Samples

Theoretical First Dilution Of Test Sample In Plate : 40.0   Sample dilution fold: 2.0

Nipha_Standard : NV-1

Concentration: 1000.0

Dilution (First dil in plate): 40.0

Dilution fold: 2.0

Others parameters

Rounding Decimal Standard Th.Conc: 1

Rounding Decimal RelErr% & CVdil: 1

Rounding Decimal GMC: 1

Average ODs of Blank: 0.046

SD of Blank: 0.001

Cutoff OD: 0.094
