# Supplementary material for: Development and Qualification of a Nipah Virus Glycoprotein-Specific IgG ELISA for the Assessment of Human Antibody Responses
Source: Vaccines (Basel). 2026 Jun 16;14(6):534. doi: 10.3390/vaccines14060534 (PMC13307770; doi:10.3390/vaccines14060534)
Supplement: Supplementary file 1 [file vaccines-14-00534-s001.zip › Supplementary_ELISA Qualification Data & Graph/4. Linearity_Analysist-2/5. Linearity_WHO IS_ANALYST-2_PLATE-2_DAY-2.pdf]

Intro

NIPAH_NIBSC_LINEARITY_ANALYST#2_PLATE#2_DAY#2

OD

	1	2	3	4	5	6	7	8	9	10	11	12
A	1.013	0.682	0.475	0.294	0.175	0.048	0.047	0.047	0.047	0.047	0.047	0.047
B	0.724	0.485	0.313	0.180	0.109	0.045	0.045	0.045	0.045	0.045	0.045	0.045
C	0.479	0.301	0.175	0.115	0.088	0.048	0.045	0.045	0.045	0.045	0.045	0.045
D	0.278	0.183	0.126	0.087	0.063	0.044	0.047	0.047	0.047	0.047	0.047	0.047
E	0.169	0.111	0.090	0.063	0.053	0.043	0.045	0.045	0.045	0.045	0.045	0.045
F	0.111	0.085	0.064	0.055	0.047	0.048	0.044	0.044	0.044	0.044	0.044	0.044
G	0.092	0.067	0.048	0.042	0.043	0.044	0.048	0.048	0.048	0.048	0.048	0.048
H	0.085	0.045	0.040	0.036	0.039	0.044	0.045	0.045	0.045	0.045	0.045	0.045

Settings Information
Endpoint
Absorbance
Lm1 450
More Settings
Shake Off
Calibrate On
Carriage Speed Normal
Column Priority

Read Information
Imported Data : 12:04 PM 10/3/2024
Imported By : anjan

Reduction Settings

Optical Density
Wavelength Combination : !Lm1

Sample Dil

Main Sample Dilution 40.0

Sample 1: NV-4 40.0

Sample 2: NV-4 (1:2) 40.0

Sample 3: NV-4 (1:4) 40.0

Sample 4: NV-4 (1:8) 40.0

Sample 5: CNC 40.0

Sample 6: BLANK 40.0

Sample 7: BLANK 40.0

Sample 8: BLANK 40.0

Sample 9: BLANK 40.0

Sample 10: BLANK 40.0

Sample 11: BLANK 40.0

Standards

Sample	Wells	OD	OK OD	Dilution	Calc.Conc	Adj.Conc	GMC	N	Th.Conc	RelErr%
01	A1	1.013	1.013	40	25.155	1006.2	982.5	6	25.000	0.600
	B1	0.724	0.724	80	12.264	981.1			12.500	-1.900
	C1	0.479	0.479	160	6.472	1035.5			6.300	2.700
	D1	0.278	0.278	320	3.109	995.0			3.100	0.300
	E1	0.169	0.169	640	1.535	982.5			1.600	-4.100
	F1	0.111	0.111	1280	0.703	900.2			0.800	-12.100
	G1	0.092		2560					0.400	
	H1	0.085		5120					0.200	

Samples

Sample	Wells	ID	OD	OK OD	Dilution	Calc.Conc	Adjusted.Conc	GMC	N	CVdil
01	A2	1	0.682	0.682	40	11.069	442.752	503.1	5	11.1
	B2		0.485	0.485	80	6.586	526.903			
	C2		0.301	0.301	160	3.457	553.101			
	D2		0.183	0.183	320	1.733	554.703			
	E2		0.111	0.111	640	0.703	450.096			
	F2		0.085		1280					
	G2		0.067		2560					
	H2		0.045		5120					
02	A3	2	0.475	0.475	40	6.396	255.834	274.8	4	7.5
	B3		0.313	0.313	80	3.641	291.290			
	C3		0.175	0.175	160	1.620	259.219			
	D3		0.126	0.126	320	0.922	295.167			
	E3		0.090		640					
	F3		0.064		1280					
	G3		0.048		2560					
	H3		0.040		5120					
03	A4	3	0.294	0.294	40	3.350	134.015	130.3	3	5.7
	B4		0.180	0.180	80	1.691	135.275			
	C4		0.115	0.115	160	0.762	121.956			
	D4		0.087		320					
	E4		0.063		640					
	F4		0.055		1280					
	G4		0.042		2560					
	H4		0.036		5120					
04	A5	4	0.175	0.175	40	1.620	64.805	59.1	2	13.1
	B5		0.109	0.109	80	0.674	53.890			
	C5		0.088		160					
	D5		0.063		320					
	E5		0.053		640					
	F5		0.047		1280					
	G5		0.043		2560					
	H5		0.039		5120					
05	A6	5	0.048		40			N/A	0	-----
	B6		0.045		80					
	C6		0.048		160					
	D6		0.044		320					
	E6		0.043		640					
	F6		0.048		1280					
	G6		0.044		2560					
	H6		0.044		5120					
06	A7	6	0.047		40			N/A	0	-----
	B7		0.045		80					
	C7		0.045		160					
	D7		0.047		320					
	E7		0.045		640					
	F7		0.044		1280					
	G7		0.048		2560					
	H7		0.045		5120					
07	A8	7	0.047		40			N/A	0	-----
	B8		0.045		80					
	C8		0.045		160					
	D8		0.047		320					
	E8		0.045		640					
	F8		0.044		1280					
	G8		0.048		2560					
	H8		0.045		5120					
08	A9	8	0.047		40			N/A	0	-----
	B9		0.045		80					
	C9		0.045		160					
	D9		0.047		320					

Samples (Contd)

Sample	Wells	ID	OD	OK OD	Dilution	Calc.Conc	Adjusted.Conc	GMC	N	CVdil
	E9		0.045		640					
	F9		0.044		1280					
	G9		0.048		2560					
	H9		0.045		5120					
09	A10	9	0.047		40			N/A	0	-----
	B10		0.045		80					
	C10		0.045		160					
	D10		0.047		320					
	E10		0.045		640					
	F10		0.044		1280					
	G10		0.048		2560					
	H10		0.045		5120					
10	A11	10	0.047		40			N/A	0	-----
	B11		0.045		80					
	C11		0.045		160					
	D11		0.047		320					
	E11		0.045		640					
	F11		0.044		1280					
	G11		0.048		2560					
	H11		0.045		5120					
11	A12	11	0.047		40			N/A	0	-----
	B12		0.045		80					
	C12		0.045		160					
	D12		0.047		320					
	E12		0.045		640					
	F12		0.044		1280					
	G12		0.048		2560					
	H12		0.045		5120					

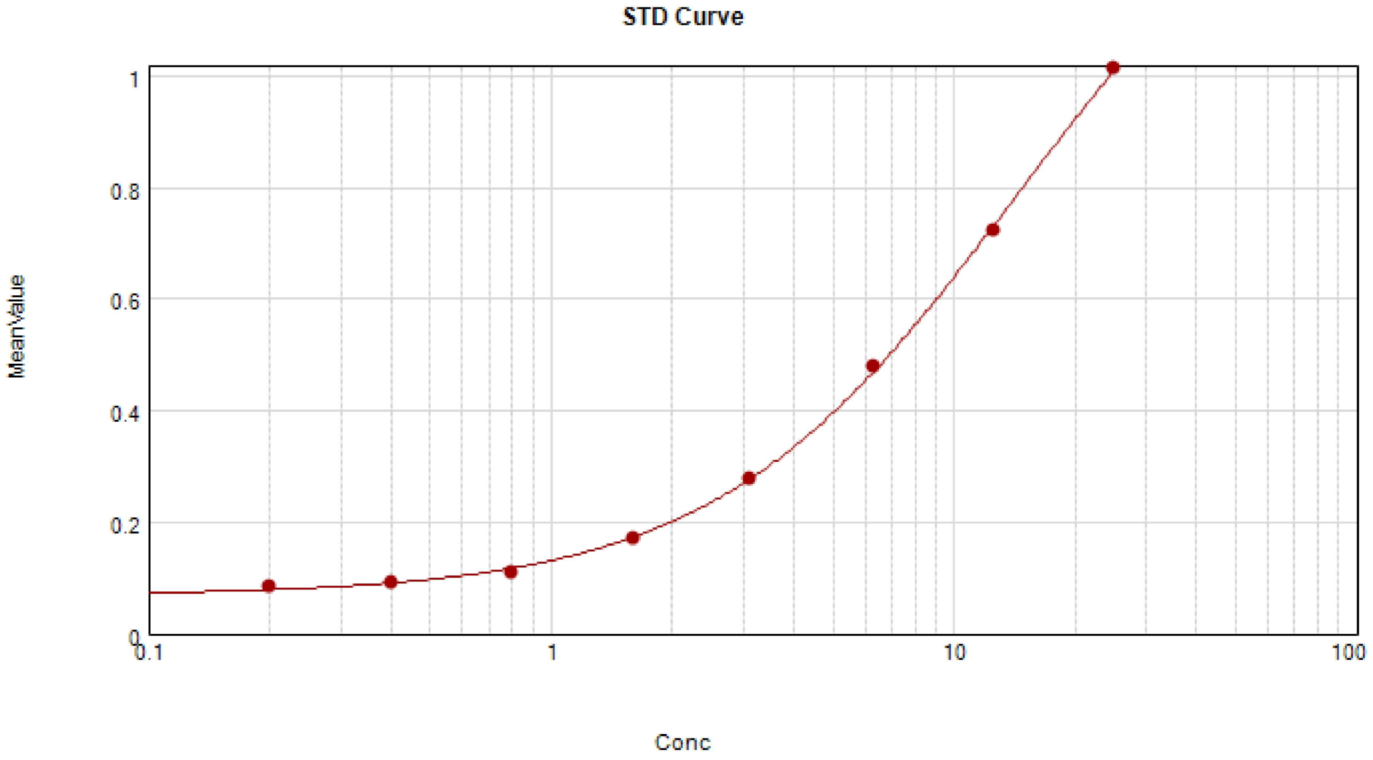

● **Std** (Standards: OD vs Th.Conc) Weighting: Fixed

Curve Fit Results ▲

Curve Fit : 4-Parameter Logistic $y = D + \frac{A - D}{1 + \left(\frac{x}{C}\right)^B}$

	Parameter	Estimated Value	Std. Error	Confidence Interval
**Std** R^2 = 1.000 EC50 = 14.05	A	0.069	0.007	[0.049, 0.089]
	B	1.166	0.066	[0.982, 1.350]
	C	14.05	1.556	[9.728, 18.37]
	D	1.492	0.087	[1.250, 1.733]

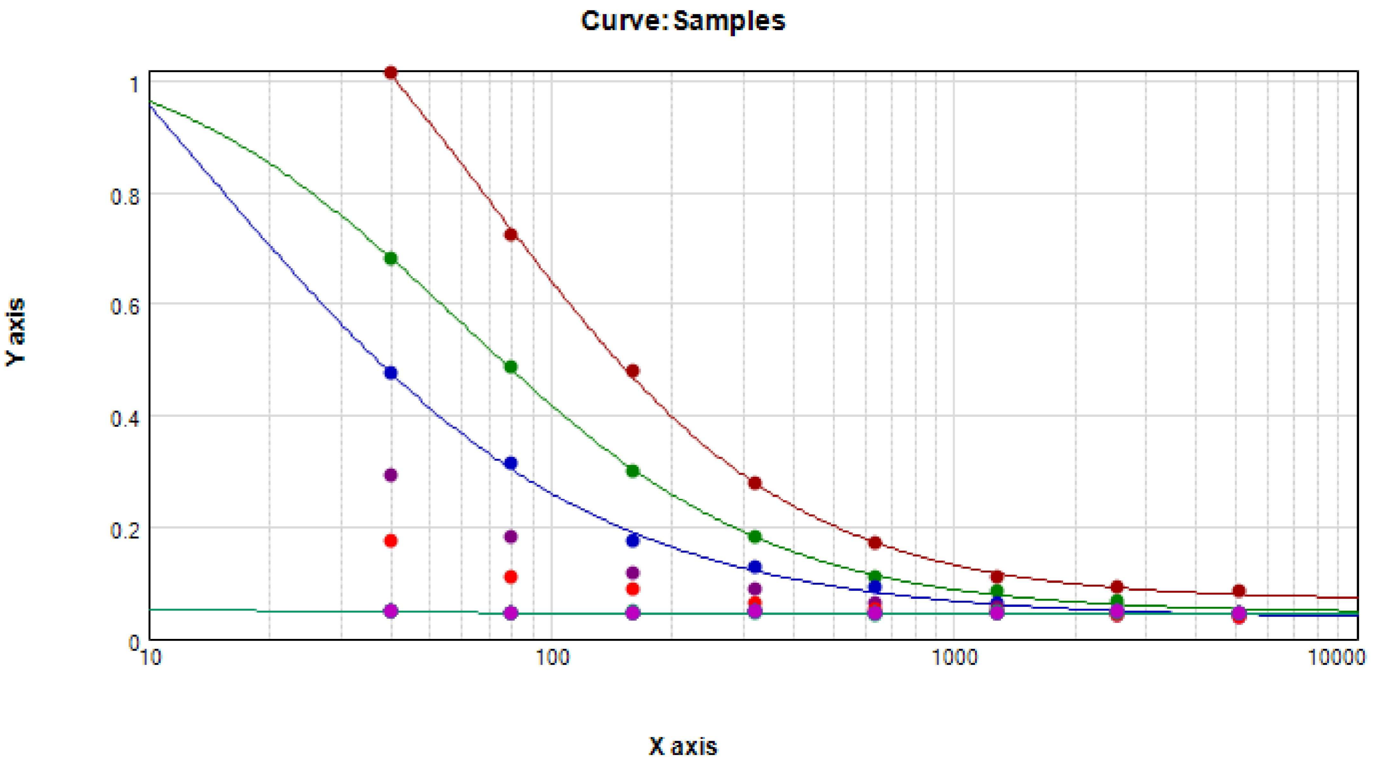

- **STD** (Standards: OD vs Dilution) Weighting: Fixed
- **S-1** (Samples: ODS1 vs DilSple1) Weighting: Fixed
- **S-2** (Samples: ODS2 vs DilSple2) Weighting: Fixed
- **S-3** (Samples: ODS3 vs DilSple3) Weighting: Fixed
- **S-4** (Samples: ODS4 vs DilSple4) Weighting: Fixed
- **S-5** (Samples: ODS5 vs DilSple5) Weighting: Fixed
- **S-6** (Samples: ODS6 vs DilSple6) Weighting: Fixed
- **S-7** (Samples: ODS7 vs DilSple7) Weighting: Fixed
- **S-8** (Samples: ODS8 vs DilSple8) Weighting: Fixed
- **S-9** (Samples: ODS9 vs DilSple9) Weighting: Fixed
- **S-10** (Samples: ODS10 vs DilSple10) Weighting: Fixed
- **S-11** (Samples: ODS11 vs DilSple11) Weighting: Fixed

Curve Fit Results ▼

Assay Parameter

Samples

Theoretical First Dilution Of Test Sample In Plate : 40.0   Sample dilution fold: 2.0

Nipha_Standard : NV-1

Concentration: 1000.0

Dilution (First dil in plate): 40.0

Dilution fold: 2.0

Others parameters

Rounding Decimal Standard Th.Conc: 1

Rounding Decimal RelErr% & CVdil: 1

Rounding Decimal GMC: 1

Average ODs of Blank: 0.046

SD of Blank: 0.001

Cutoff OD: 0.093
